# A minimal Fanconi Anemia complex in early diverging fungi

**DOI:** 10.1038/s41598-024-60318-w

**Published:** 2024-04-30

**Authors:** Drishtee Barua, Magdalena Płecha, Anna Muszewska

**Affiliations:** Institute of Biochemistry and Biophysics, Polish Academy of Sciences, Pawińskiego 5A, 02-106 Warsaw, Poland

**Keywords:** Interstrand crosslinks, Fanconi Anemia, Early diverging fungi, Ancestral trait, Fungal evolution, Opisthokonta, Evolutionary biology, Fungal evolution, Fungal genomics, Mechanisms of disease

## Abstract

Fanconi Anemia (FA) pathway resolves DNA interstrand cross links (ICL). The FA pathway was initially recognized in vertebrates, but was later confirmed in other animals and speculated in fungi. FA proteins FANCM, FANCL and FANCJ are present in S*accharomyces cerevisiae* but, their mechanism of interaction to resolve ICL is still unclear. Unlike *Dikarya*, early diverging fungi (EDF) possess more traits shared with animals. We traced the evolutionary history of the FA pathway across *Opisthokonta*. We scanned complete proteomes for FA-related homologs to establish their taxonomic distribution and analyzed their phylogenetic trees. We checked transcription profiles of FA genes to test if they respond to environmental conditions and their genomic localizations for potential co-localization. We identified fungal homologs of the activation and ID complexes, 5 out of 8 core proteins, all of the endonucleases, and deubiquitination proteins. All fungi lack FANCC, FANCF and FANCG proteins responsible for post-replication repair and chromosome stability in animals. The observed taxonomic distribution can be attributed to a gradual degradation of the FA pathway from EDF to *Dikarya*. One of the key differences is that EDF have the ID complex recruiting endonucleases to the site of ICL. Moreover, 21 out of 32 identified FA genes are upregulated in response to different growth conditions. Several FA genes are co-localized in fungal genomes which also could facilitate co-expression. Our results indicate that a minimal FA pathway might still be functional in *Mucoromycota* with a gradual loss of components in *Dikarya* ancestors.

## Introduction

Interstrand cross-links (ICL) are a lethal type of DNA damage where the two strands of DNA get cross-linked by covalent bonds, thereby halting the progress of DNA replication and transcription^1^. ICL may arise endogenously through nitrous acids and aldehydes, or exogenously through chemotherapeutic drugs such as Cisplatin and Mitomycin C^2^. Studies show that ICL are the major cytotoxic lesions produced by these drugs^3^. If left unrepaired, they stall DNA replication leading to mutations, chromosome breakage and cell death. ICL is repaired by several pathways: Nucleotide Excision Repair (NER), Homologous Recombination (HR) and Translesion DNA Synthesis (TLS) working in coordination^4^. The Fanconi Anemia (FA) pathway recognizes ICL and coordinates the ICL repair process^5^.

Fanconi Anemia is one of the rare genetic disorders characterized by a progressive bone marrow failure, congenital abnormalities along with cancer predisposition^6,7^. FA disease is linked to the failure of ICL repair^8^. Mutations in FANC genes are the most frequent cause of FA^9^. The FA pathway is activated by ICL during the S phase of the cell cycle in mammals^10^ and possibly other eukaryotes^4^. Recent studies confirmed 22 genes to be FANC genes (Table 1). The remaining FA genes encode downstream effectors of the FA pathway and mutations in them do not cause classical FA symptoms^6^. It has been reported that the FA pathway is coupled with Double-strand Break (DSB) repair and NER^11^.

**Table 1:** Composition of Fanconi Anemia pathway genes, gene name aliases and protein domains in the protein products of FA genes (subcomplex nomenclature after Niraj and co-workers^7^).

Gene	Alias	Pfam/Interpro accession	FA subcomplex
MHF1	CENPS/ FAAP16	PF15630/IPR029003	Activation
MHF2	CENPX/ FAAP10	PF09415/IPR018552	
FANCA		PF03511/IPR003516	Core complex
FANCB		IPR033333	
FANCC		PF02106/IPR000686	
FANCE		PF11510/IPR039685	
FANCF		PF11107/IPR035428	
FANCG	XRCC9	IPR039684	
FANCJ	BRIP1	PF06733/IPR010614	
FANCL		PF11793/IPR026848	
FANCM		PF16783/IPR031879	
FAAP20		PF15750/IPR031490	
FAAP24		PF17949/IPR026985	
FAAP100		PF15146/IPR029251	
FANCT	UBE2T	PF00179/IPR000608	Monoubiquitination
ATR		PF00454/IPR000403	
UHRF1		PF02182/IPR047406	
UHRF2		PF02182/IPR047468	
FANCD2		PF14631/IPR029448	ID complex
FANCI		PF14674-80/IPR026171	
SLX1		PF21202/IPR048749	Endonuclease
FANCP	SLX4	PF09494/IPR018574	
FAN1		PF08774/IPR014883	
EME1		PF21292/IPR033310	
MUS81		PF21136/IPR033309	
FANCQ	XPF	PF02732/IPR006167	
ERCC1		PF03834/IPR047260	
FANCS	BRCA1	PF00533/IPR011364	HR and DNA repair
FANCD1	BRCA2	PF09103-4/IPR015525	
FANCN	PALB2	PF16756/IPR042417	
FANCO	RAD51C	PF08423/IPR013632	
FANCR	RAD51	PF08423/IPR011941	
FANCU	XRCC2	PF08423/IPR030547	
FANCV	REV7	PF02301/IPR003511	
FANCW	RFWD3	PF13639/IPR037381	
REV1		PF16727/IPR031991	
REV3		IPR030559	
DPOLN		PF00476/IPR001098	
USP1	UBP1	PF00443/IPR033815	Deubiquitination
UAF1	WDR48	PF11816/IPR021772	

An activation complex of FANCM-FAAP24-MHF1-MHF2 recognizes ICL when a break in the replication process occurs. MHF1 (FAAP16) and MHF2 (FAAP10) form a (MHF1-MHF2) × 2 tetramer, FANCM forms a heterodimer with FAAP24 and together, they bind to MHF1/2 tetramer through a dual V-shaped structure^12^. This anchor complex recruits the FA core complex to sites of DNA damage.

The FA core complex, formed by eight FANC proteins (FANCA, FANCB, FANCC, FANCE, FANCF, FANCG, FANCL, FANCM) and three FA associated proteins (FAAP20, FAAP24, FAAP100), provides the essential E3 ubiquitin ligase function for FANCD2 ubiquitination. FANCC regulates meiotic crossover along with FANCE and FANCF^13^. The FANCA-FANCG-FAAP20 aids in the process of strand annealing and strand exchange^14^. FANCM, a multi-domain protein, binds to the core complex by the interaction of its MM1 domain with FANCF. Its MM2 domain on the other hand, interacts with the BTR complex and participates in homologous recombination^15^. The molecular functions for the remaining core proteins remain elusive, apart from binding to DNA.

The monoubiquitination of FANCD2 and FANCI, forming the ID complex, is the hallmark of the FA pathway. This ID complex facilitates recruitment of the downstream effector proteins to ICL^8^. The process involves attachment of a single ubiquitin molecule to a specific site in FANCD2 and FANCI^16^ and is carried out by FA core complex together with UBE2T. The E3 ligase FANCL acts as a catalyst, with its C-terminal RING domain binding to UBE2T and a central double RWD domain that binds to FANCD2^8^ to carry out the ubiquitination. The RWD domain is also said to stimulate the activity of UBE2T^17^. Along with UBE2T, ATR phosphorylates FANCD2 and FANCI via its effector kinase Chk1 to stabilize its association with DNA and FANCD2^18^. A recent study identified a novel ICL sensor protein—E3 ubiquitin ligase UHRF2, which along with its paralogue UHRF1, interacts with FANCD2 after its recruitment in the DNA and facilitates the retention of FANCD2 to the site of ICL^19^.

The downstream effector proteins comprises endonucleases and repair proteins that contribute to ICL repair. Coordination of nuclease activity begins with SLX4 (FANCP) that functions as a scaffold, modulator and a docking platform for SLX1, MUS81-EME1 and XPF-ERCC1 structure-specific nucleases^5,20^. The FAN1 nuclease contains a UBZ domain in its N-terminus that acts as a platform for binding to FANCD2. The monoubiquitinated ID complex recruits this endonuclease complex to the site of ICL that carries out incisions to unhook the crosslinked bases^16^. Studies identified that XPF-ERCC1 is the most important for resistance to ICL^5^. This process of unhooking is necessary for initiation of TLS. The FANCJ helicase binds to FANCD2 and regulates chromatin localization^21^.

The TLS polymerase consisting of REV1, REV3-REV7 or Pol ζ complex and DPOLN (DNA polymerase ν) is subsequently recruited to carry out lesion bypass. While REV1 inserts the first nucleotide opposite a damaged base, REV3 performs the extension step. The polymerase REV3 is particularly specialized in extending distorted base pairs (such as mismatches due to inaccurate base insertion by another TLS polymerase). REV7, a newly identified FA gene, acts as an adaptor between REV1 and REV3^22^, and together they perform lesion bypass and are considered the main players of post-replication repair^23^. DPOLN (DNA polymerase ν), a Y-family polymerase protein, is able to carry out insertions as well extensions in a diverse set of minor and major groove ICL with no stalling of replication forks^24^. In the case of TLS with A-family polymerase proteins (such as polη), a monoubiquitinated PCNA is required for recruiting polη to the site of break and ensure accurate replicative bypass TLS activation^25^. TLS involving DPOLN is controlled by the FA core complex and is independent of the PCNA ubiquitination event in mammals^26^. Proteins FANCO, FANCU and RAD51 are components of the RAD51 paralog complex BCDX2^27^. The BCDX2 complex contains RAD51B, RAD51C, RAD51D, and XRCC2; it binds to single-stranded DNA, nicks in duplex DNA and to single-stranded regions in duplex DNA^28^. FANCO forms RAD51 foci in response to the damage^29^. It facilitates phosphorylation of the checkpoint kinase CHEK2 and thereby transduction of the damage signal, leading to cell cycle arrest and HR activation^26^. XRCC2, classified as an FA gene (FANCU), stabilizes RAD51^27^. The FA components, BRCA1, BRCA2 and PALB2 take part in the subsequent steps of DNA repair involving HR^5^. BRCA1 functions upstream of BRCA2, and recruits PALB2^30^. BRCA2 helps load RAD51 to the DNA by interaction of its BRC4 repeat with the RecA domain of RAD51^31^. PALB2 binds directly to both BRCA1 and BRCA2, and together they remove the CMG helicase from stalled replication forks^7^.

The end of the FA pathway is marked with the deubiquitination of FANCD2/ FANCI heterodimer by the USP1-UAF1 complex^32^. It is carried out by the interaction of deubiquitylating enzymes USP1 and UAF1 with FANCD2 in the chromatin. The ID complex is deubiquitylated by USP1 whose activity is enhanced by the interaction with UAF1^33^.

**Figure 1:**
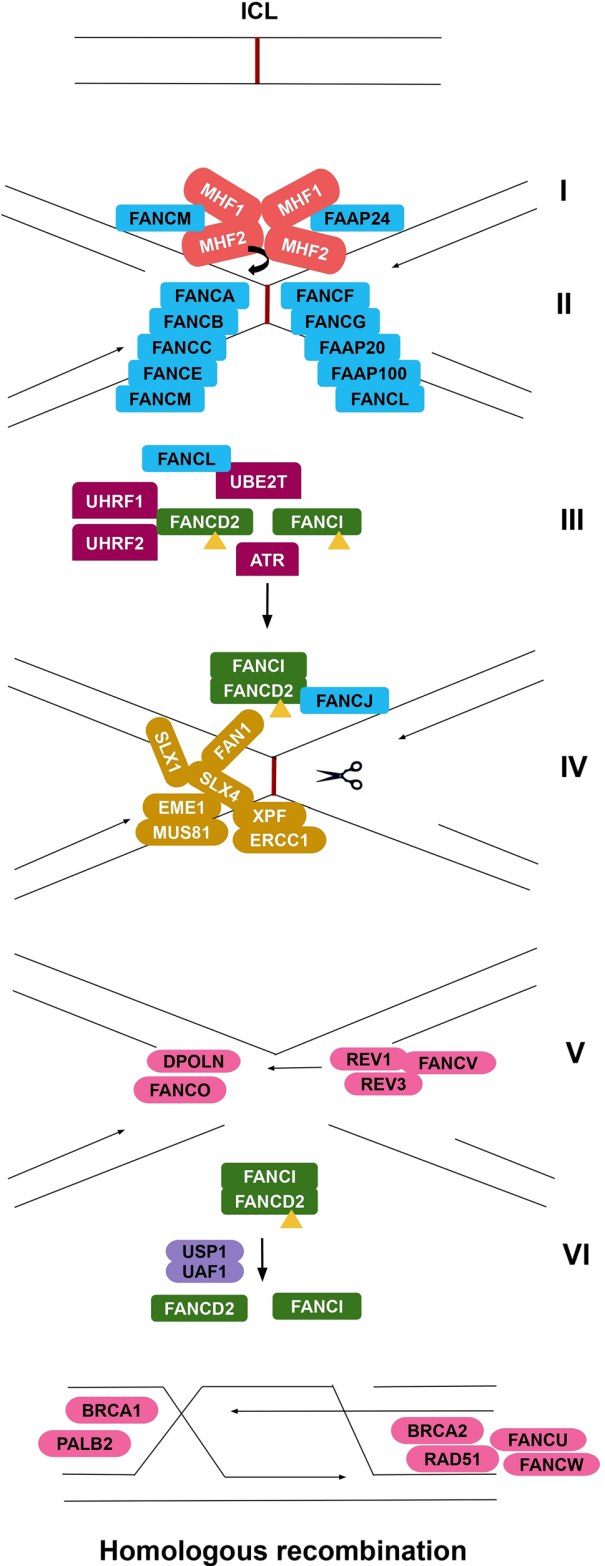
Schematic representation of the Fanconi Anemia pathway in humans, derived from^34^. [I—Activation of FA pathway; II—Binding of FA core complex to the ICL site; III—Monoubiquitination of FANCD2 & FANCI to form the ID complex; IV—Formation of endonuclease complex and subsequent ICL unhooking; V—Translesion synthesis or lesion bypass; VI—Deubiquitination of ID complex and closure of FA pathway.]

ICL repair by FA pathway is best studied in humans^2^ and mice^35^ (Fig. 1). However, studies show that FA core binding proteins are present in all classes of sponges, pointing to the ancient origin of the FA pathway in animal evolution^36^. The nematode *Caenorhabditis elegans* has a functional ortholog of the DEAD-box helicase FANCJ, termed as DOG-1 (Deletions of G-rich DNA) that functions alongside FANCD2, FANCM, FANCO, FANCI proteins and maintains the stability of its G-rich DNA^37^. The function of FA proteins was also recently studied in *Drosophila melanogaster*, where the monoubiquitination of FANCD2 was found to be linked to a mitosis-specific DNA DSB response^38^. Proteins MHF1 and MHF2, were previously identified as anti-crossover factors during meiosis in *Arabidopsis thaliana*^13^. Singh and colleagues identified FANCC as another anti-crossover gene and showed that FANCC, FANCE and FANCF subcomplex was conserved from vertebrates to plants and it regulates meiotic recombination^13^. There have also been attempts to study the FA pathway in non-animal organisms. For instance, studies in *Saccharomyces cerevisiae* report the presence of putative homologs of FA activation proteins MHF1 and MHF2 (CENPS and CENPX respectively) as well as FA core binding proteins FANCM, FANCJ and FANCP (MPH1, CHL1 and SLX4 respectively)^4,39^. However, yeast cells deficient in aforementioned protein-coding genes displayed no significant sensitivity to ICL. Instead, they require a combination of different DNA repair systems: NER, HR and post-replication repair to mitigate ICL^4^.

In light of these studies, there is substantial evidence of the FA pathway beyond mammals, with some missing proteins. The discoveries in amoebozoa, non-mammalian animals and fungi led us to ask questions about the existence of FA pathway in ancestral Opisthokonts and early diverging fungal (EDF) lineages. Here, we conduct a genomic survey of FA proteins across the fungal tree of life. We propose a hypothetical model of FA pathway for distinct evolutionary lineages and recapitulate the evolutionary history of FA complexes across taxa.

## Results

### Distribution of proteins

**Figure 2:**
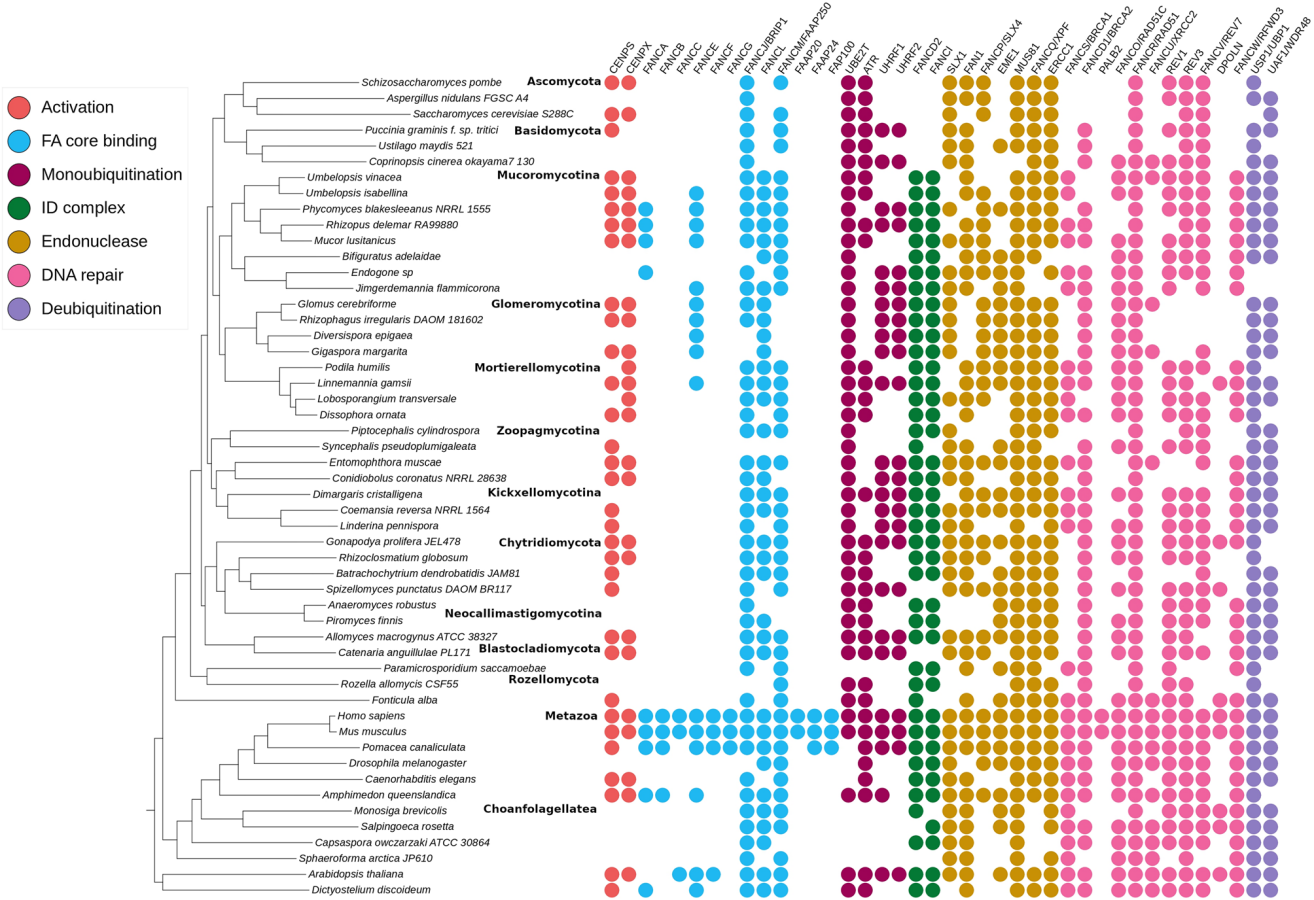
Distribution of 40 FA and FA associated components among selected eukaryotes.

Genes encoding proteins from the FA pathway are conserved in evolution because of their involvement in cellular homeostasis. To recover the distribution of individual components of FA pathway, we mapped 40 reference proteins on a collection of 183 fungal and five early diverging *Opisthokonta* proteomes (protein sets derived from whole genome sequencing projects) (10.5281/zenodo.10911400, Supplementary Table S1). Out of the aforementioned 40 FA selected reference sequences, 32 have homologs in fungal proteomes (Fig. 2, Supplementary Results).

### Transcriptomics of FA proteins

#### FA proteins expressed in pure culture transcriptomic studies of EDF

Since most of the identified homologs of FA components are annotated as hypothetical unknown proteins without experimental characterization, we looked for their gene expression levels in the available EDF whole transcriptomes. The number of transcripts found serves as an intermediate evidence that the predicted FA homologs in fungi originate from active genes in normal conditions. The analyzed transcriptomes corroborate the expression of 27 out of 32 identified FA homologs in representatives of *Mucorales* (*M. lusitanicus, J. flammicorona*), *Endogonales* (*Endogone* sp.), *Umbelopsidales* (*U. isabellina*) and *Mortierellomycotina* (*L. transversale*). For instance, *M. lusitanicus* expressed four out of eight core binding genes (FANCA, FANCE, FANCL, FANCM) and one ID complex gene FANCD2 predicted for *Mucoromycotina*. The activation genes MHF1 and MHF2 and the monoubiquitination gene UBE2T were also expressed, along with endonucleases SLX1, MUS81, FAN1 and repair genes REV1, REV3, FANCV, BRCA2 and RAD51. The *Mortierellomycotina L. transversale* expressed copies of MHF2, UBE2T, ATR, FANCD2 and FANCI, FANCL, FANCM, FANCO, SLX1, MUS81, FAN1, REV1, REV3, FANCV, DPOLN, FANCW and BRCA1 genes. The transcriptomes of *Endogone* sp*.* expressed UHRF1, MUS81, ERCC1 and RAD51 and *J. flammicorona* expressed FANCJ, ATR, DPOLN and FANCW, while *U. isabellina* expressed FANCD2, UBP1, REV1 and two copies of UBE2T. (10.5281/zenodo.10911400, Supplementary Table S1).

#### FA proteins expressed in condition-specific transcriptomic studies of EDF

In order to test the functionality of the FA candidate genes, if they are expressed and regulated depending on environmental conditions, we analyzed expression profiles of genes corresponding to the proteins with available transcriptomic data obtained from various environmental conditions. A total of 26 out of 32 genes encoding homologs of FA pathway components were expressed under multiple conditions. This result suggests that the genes are actively regulated and may be involved in stress responses.

**Figure 3:**
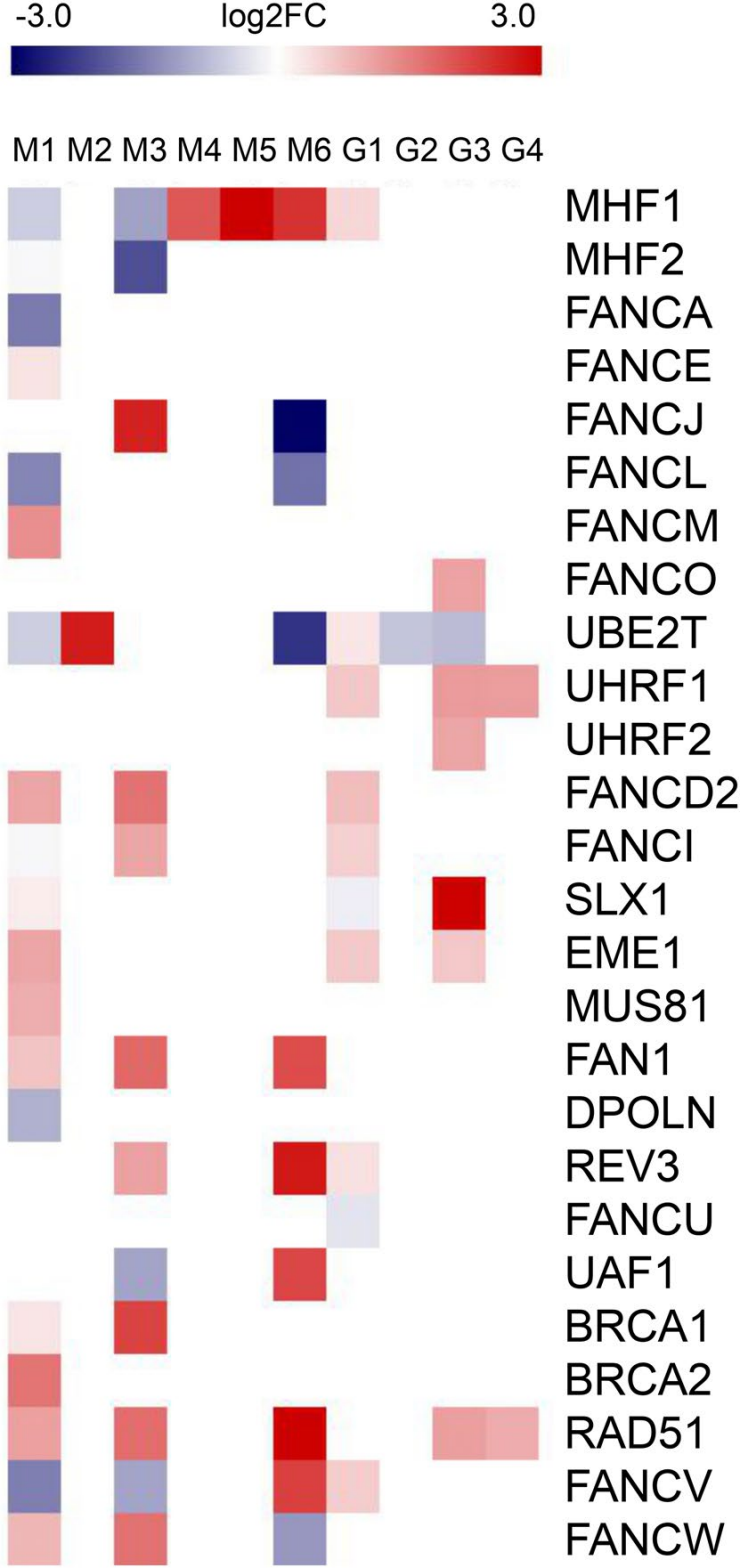
Differential expression profiling of 26 out of 32 FA pathway genes expressed in different environmental conditions in members of *Mucoromycotina* and *Glomeromycotina*. The empty cells signify the absence of gene expression from the organism in the given environmental condition. M1: *Mucor lusitanicus* growth in anaerobic vs aerobic conditions; M2: *Rhizopus microsporus* growth in presence vs absence of murine macrophage; M3: *Rhizopus delemar* growth in presence vs absence of murine macrophage; M4: *Rhizopus delemar* host-pathogen interaction (6 h) using human airway epithelial cells (A549); M5: *Rhizopus delemar* host-pathogen interaction (16 h) using human airway epithelial cells (A549); M6: *Rhizopus delemar* host-pathogen interaction (18 h) using mouse bone marrow-derived macrophages (BM); G1: *Gigaspora rosea* response to plant signals in the switch from asymbiotic to presymbiotic growth; G2: *Rhizophagus irregularis* growth in *Lotus japonicus* roots exposed to different concentrations of phosphate (20 μM, 100 μM, 300 μM, 500 μM); G3: *Rhizophagus irregularis* association with *Medicago truncatula* and treatment with strigolactone for 24 h; G4: *Rhizophagus irregularis* association with *Medicago truncatula* and treatment with strigolactone for 1 week.

Our analysis of *M. lusitanicus* (M1) transcripts expressed under anaerobic vs aerobic growth conditions^40^ identified 19 out of 26 predicted FA genes*.* Differential expression analysis showed upregulation in the FA core binding and ID complex genes: FANCD2, FANCE and FANCM, along with endonucleases SLX1, EME1, MUS81, FAN1 and repair genes BRCA1, BRCA2, RAD51 and FANCW. The activation complex genes MHF1 and MHF2, ubiquitylating gene UBE2T, 3 FA core genes FANCA, FANCI and FANCL along with DPOLN and FANCV repair genes were downregulated (Fig. 3). Transcriptomics of *R. delemar* (M3) during exposure to murine macrophages^41^ showed upregulation of FANCD2, FANCI, FANCJ, FAN1, REV3, BRCA1, RAD51 and FANCW and downregulation of MHF1 and MHF2, UAF1 and FANCV (Fig. 3). On the other hand, a similar study conducted on *R. microsporus* (M2) displayed upregulation of only UBE2T. Transcriptomic studies on *R. delemar* were also found centering human host–pathogen interaction against mucormycosis. In one of the studies, A549 airway epithelial cells were subjected to in vitro infection with *R. delemar* for 6 h (M4) and 16 h (M5)^42^. The MHF1 gene was found to be upregulated at both timepoints. In another study, mouse bone-marrow derived macrophages (BMDMs) were infected with *R. delemar* for 1 h, 4 h and 18 h^43^, and we found the expression of FA genes from the samples infected for 18 h (M6). The genes MHF1, FAN1, REV3, RAD51, UAF1 and FANCV were upregulated while FANCJ, FANCL, UBE2T and FANCW were downregulated (Fig. 3).

The study involving *G. rosea* treated with G24 (G1), a synthetic analog of strigolactone, addressed its gene regulation in response to plant signals in the switch from asymbiotic growth to presymbiotic growth^44^. Our data analysis displayed upregulation of MHF1, UBE2T, UHRF1, FANCD2, FANCI, EME1, REV3 and FANCV respectively with multiple copies of UBE2T. SLX1 and FANCU were downregulated (Fig. 3).

Two different transcriptomic studies were obtained for *R. irregularis.* One study concerned the transcriptomic profiling of AM roots exposed to varying concentrations of phosphate (20 μM, 100 μM, 300 μM, 500 μM) (G2)^45^, where multiple copies of UBE2T were downregulated. Another study focused on the differential expression of *R. irregularis* during AM symbiosis by harvesting strigolactone-treated spores a day (G3) and a week (G4) after inoculation respectively^46^. Our analysis revealed that spores harvested a day post inoculation had upregulation of FANCO, UHRF1, UHRF2, SLX1,EME1 and RAD51, and downregulation of UBE2T, while on the other hand, UHRF1 and RAD51 were upregulated in the spores harvested a week post inoculation (Fig. 3).

We also checked the expression profiling of genes involved in the NER, MMR and DSB repair pathways in order to find their correlation with the down- or upregulation of FA genes. All of the NER, MMR and DSB genes were upregulated in *R. delemar*, *M. lusitanicus* and *R. microsporus*, regardless of the environmental conditions in contrast to the results found for FA genes. However, in the case of *G. rosea* and *R. irregularis*, genes involved in DSB repair were particularly downregulated, while those involved in NER and MMR were upregulated.

#### FA genes not expressed in transcriptomic analysis

Generally, at least one gene from every FA pathway subcomplex was detected in the transcriptomes of pure culture and treated fungal species. The transcriptomes obtained from axenic cultures of *Mucoro-* and *Glomeromycotina* members did not detect the genes encoding endonucleases: EME1, SLX4 and XPF (10.5281/zenodo.10911400, Supplementary Table S1). While on the other hand, transcriptomes obtained from organisms subjected to condition-specific expression did not detect genes encoding ATR, XPF, ERCC1 and SLX4, REV1, and UBP1 (not shown in Fig. 3).

### Genomic co-occurrence of FA genes

**Figure 4:**
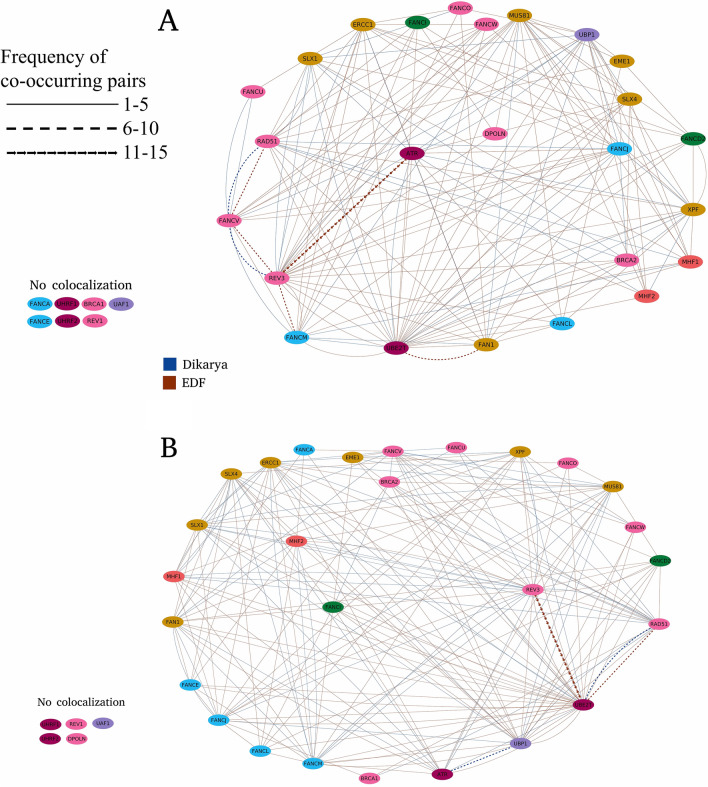
Co-occurrence of FA genes within distances of (**A**) 250 kb and (**B**) greater than 250 kb observed in *Dikarya* and EDF assemblies.

Fungal genes involved in one metabolic process are often clustered in fungi^47^. We found that several genes involved in FA pathway are located on the same contig. Usually, only two or three FA genes were colocalized, however, we found up to ten genes co-occurring on a single contig in selected *Ascomycota* (10.5281/zenodo.10911400, Supplementary Table S1). Colocalization with another FA gene within a distance of 250 kb was observed for 18% of *Dikarya* FA genes and for 19% of EDF genes encoding FA proteins. The distance of 250 kb was chosen as a threshold based on the occurrence of biologically important interactions (such as contacts between enhancers and promoters) in this range^48^. A hypergeometric distribution test performed on 56 genome datasets showed that the observed co-occurrences of FA genes are not expected at random (with the highest *p* value = 0.009) (10.5281/zenodo.10911400, Supplementary Table S1). Co-occurrence of proteins in distances greater than 250 kb were classified into long-range colocalization patterns^49^. FA genes were not localized on the same contig with any other FA gene in a minority of taxa (7/36 *Dikarya* and 30/116 EDF) (10.5281/zenodo.10911400, Supplementary Table S1). Not all FA genes are equally likely to co-occur with others; we did not observe any proximity to other FA genes for FANCA, FANCE, UHRF1, UHRF2, BRCA1, REV1 and UAF1 within 250 kb distance range (Fig. 4A). FA genes in *Chytrids* and *Blastocladiomycota* co-occurred in groups of four, the distances among each gene ranging between 200 and 300 kb (10.5281/zenodo.10911400, Supplementary Table S1).

In *Dikarya*, we mainly observed long-range colocalization between FA genes. However, genes UHRF1, UHRF2, DPOLN, REV1 and UAF1 did not co-occur even in large distances for all fungal groups (Fig. 4B).

Genes coding proteins which directly interact, for instance FANCD2 and FANCI, were not located together in any of the analyzed genomes. However, these genes tend to co-occur with a ubiquitination, an endonuclease or a deubiquitination gene across all EDF.

Endonucleases are the products of genes most often co-occurring with other FA components, particularly the genes for REV3 and UBE2T or ATR are located close to each other (Fig. 4).

Four *Chytridiomycota* representatives have two-domain FA proteins: an N-terminal FANCM domain and a C-terminal XPF endonuclease (TPX77543.1, *Chytriomyces confervae*; TPX61370.1, *Powellomyces hirtus*; ORY52110.1, *Rhizoclosmatium globosum*; KNC96281.1, *Spizellomyces punctatus* DAOM BR117) (10.5281/zenodo.10911400, Supplementary Table S1).

### Differences in domain architecture

For 30 out of 32 predicted FA proteins, fungal sequences clustered together with the animal reference sequences. However, SLX4 and FANCJ sequences formed separate clusters. Further analysis of these proteins revealed that SLX4 and FANCJ homologs display a few differences in their lengths and domain composition. This points to a possible change in protein function or specificity in fungi.

**Figure 5:**
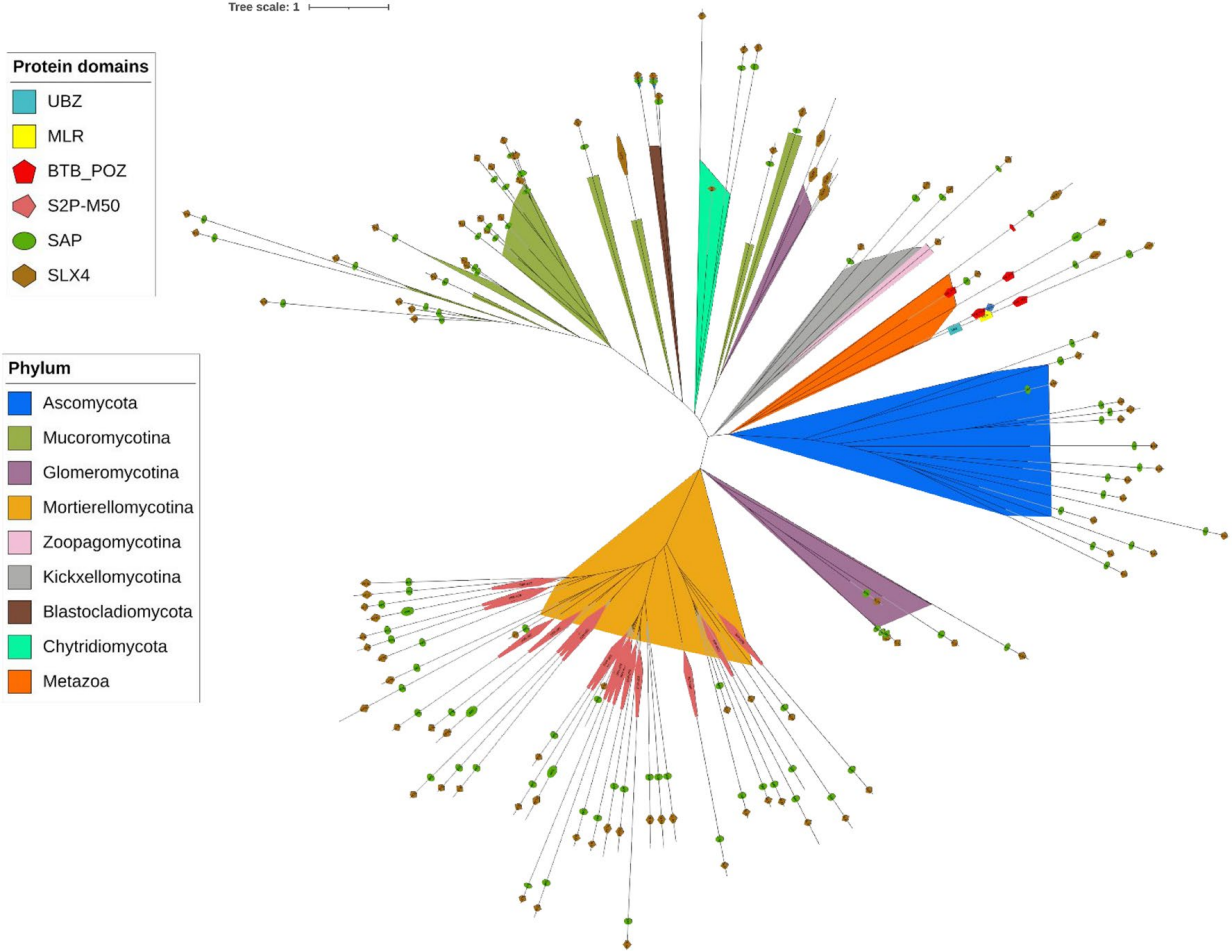
An unrooted Maximum Likelihood phylogenetic tree of SLX4 scaffold protein across selected eukaryotic lineages, drawn in iTOL^50^.

We found that SLX4 harbors an N-terminal SAP domain and a C-terminal SLX4 domain across diverse fungal lineages (Fig. 5). On the other hand, the human SLX4 is a 1834 amino acid long protein which, apart from the two domains, contains three additional domains in its N-terminus: UBZ, MLR and BTB/POZ. The BTB/POZ domain is present in SLX4 homologs of animals including sponges, but absent from all fungal homologs. The UBZ and MLR domains are also absent in fungi. Moreover, the fungal homologs are significantly shorter than their animal homologs with lengths ranging from 151 aa in *Allomyces macrogynus* (KNE55791.1, *Blastocladiomycota*) up to 423 aa in *Rhizophagus irregularis* (POG76120.1, *Glomeromycotina*). Mortierellomycotina SLX4 homologs are as long as the ones in animals (for instance, SLX4 of *Haplosporangium bisporale* KAF8951561.1, is 1993 aa long) and they also contain an additional N-terminal S2P-M50 domain.

Differences were also observed in the domain architecture of FANCJ. The FANCJ is a 5′-3′ DNA binding helicase with conserved N-terminal DEAH/DEAD box helicase and C-terminal RAD3 helicase domains. Multiple sequence alignment of FANCJ homologues in EDF points towards a deletion of the DEAD box domain. FANCJ homologs in *Olpidiomycota* and *Microsporidia* are significantly truncated with a solo DEAD/DEAH box domain without the C-terminal RAD3 helicase. These deviations from the core FANCJ domain architecture might impair its function.

### Phylogenetics of Fanconi Anemia proteins

Phylogenetic trees for 32 FA proteins are in agreement with the species tree, pertaining to the vertical inheritance of housekeeping genes in eukaryotes (10.5281/zenodo.10911400**,** Supplementary Materials SM1, Supplementary Dataset SD1). Most FA proteins occur as a single copy per proteome. A conserved domain architecture was observed in all of the analyzed Opisthokonts, with exceptions in FANCJ helicase and SLX4 scaffold protein. In addition to the differences in protein length as well as domain architecture, gene duplications were observed in members of *Blastocladiomycota*, *Entomophtoromycotina* and *Mucoromycotina* respectively. For instance, in *Allomyces macrogynus*, 2 copies were observed for proteins FANCL, FANCM, FANCO, ATR, UHRF1, UHRF2, SLX4, FAN1, whereas proteins FANCJ, and ERCC1 had four copies each. A series of duplications were observed in 6 FA proteins in *Entomophthora muscae*, namely FANCD2, FANCL, UBE2T, SLX1, ERCC1 and FAN1. The MUS81 endonuclease was found in 10 copies in *J. flammicorona*. Duplications were also observed in EDF species containing the ID proteins (FANCD2-FANCI), FANCJ helicase and endonucleases MUS81, SLX1 and FAN1.

**Figure 6:**
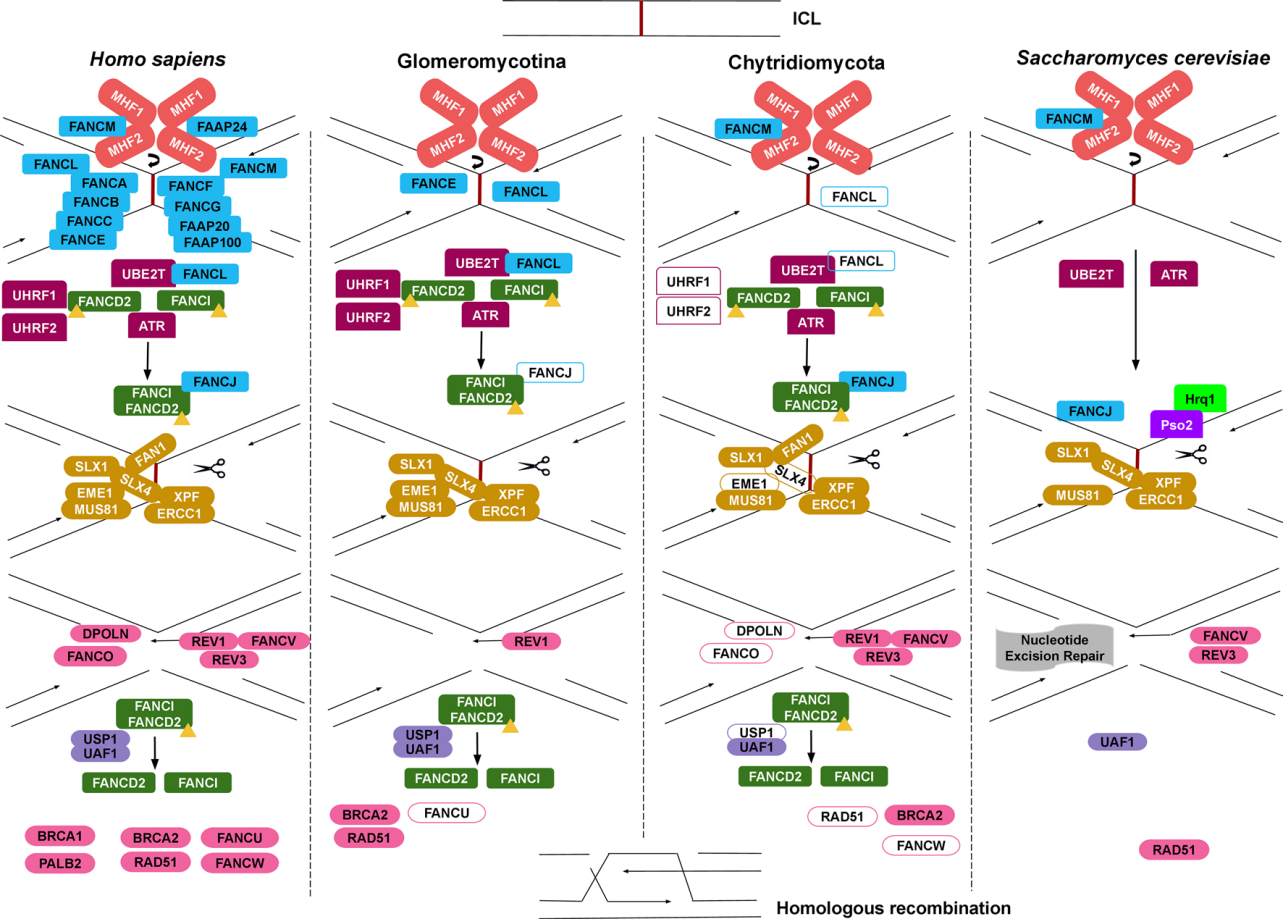
Speculative FA pathway model for *Glomeromycotina* and *Chytridiomycota* compared to *Saccharomyces cerevisiae*^51^ and *Homo sapiens*^7^. The filled shapes indicate presence of the protein in all members of the group; the shapes outlined indicate presence of the protein in 50% of the members of that group.

Taking into account all the homologs of FA components found in EDF, a model of minimal FA machinery could be proposed (depicted as an example in *Glomeromycotina* and *Chytridiomycota*) (Fig. 6). The presence of FANCM-MHF1-MHF2 activation complex could signal the existing core proteins FANCL and FANCJ to bind to the ICL site, bringing together the ubiquitination proteins. This process would be followed by monoubiquitination of FANCD2 and FANCI forming the ID complex, which along with FANCJ, would enable the build-up of endonucleases around the site of ICL. The subsequent unhooking of ICL would be followed by TLS and deubiquitination of the ID complex with USP1-UAF1 complex. The final step would be complemented with the HR proteins along with proteins from NER and DSB repair pathways. The absence of ID proteins in *Dikarya* might enable Pso2 nuclease and Hrq1 helicase to associate with FANCJ helicase and endonuclease components of FA in order to complete the unhooking of ICL.

## Discussion

In this study we show that the FA pathway is fragmentarily present in basal Opisthokonts like *S. rosetta*, *C. owczarzaki*, *M. brevicollis* and *S. arctica*, as well as in many fungal lineages, especially EDF. Recently, diverse traits were reported to exist in EDF long after their separation from the common ancestor with animals^52^. Now, the FA pathway extends the list of known ancient pathways retained in fungi.

The work of Tao and coworkers revealed that MHF 1/2 functioning requires a stable association with FANCM^12^. FANCM homolog in *Archaea* (Hef protein) resolves replication forks and possesses both helicase and endonuclease activities^53^. The FANCM ortholog of *Schizosaccharomyces pombe* (Fml1), also has FA-independent roles in DNA damage response. FANCM-MHF worked in parallel with EME1-MUS81 to process meiotic joint DNA molecules limiting crossovers^54^. On the other hand, the FANCM ortholog in *A. thaliana* was found to have no direct role in ICL DNA repair, but necessary in HR pathways in somatic cells^55^. FANCM also promotes RAD51-dependent gene conversion at stalled replication forks^56^. The absence of FANCM in *Glomeromycotina* hence, puts an open question on their mechanism of FA activation and subsequent DNA repair process. The endonuclease complex is the most conserved across all lineages; this is consistent with their involvement in universal repair pathways beyond FA. FA-specific proteins forming the core binding complex have a patchy taxonomic distribution with the whole set limited to mammals. The currently known ensemble of core binding proteins likely originated in the ancestor of vertebrates. The work of Alpi et al. (2008) demonstrated that FANCL is the crucial E3 ligase subunit and that UBE2T, FANCL and FANCI were sufficient for robust FANCD2 monoubiquitination^17^. They also speculated that flies, worms and *Dictyostelium* may possess a simplified FA monoubiquitination pathway with just three components: UBE2T, FANCL, and FANCI. The conservation of FANCD2, FANCI, UBE2T and FANCL in EDF (Fig. 2), along with their expression during stress conditions (Fig. 3) provides evidence of a simplified, functioning core complex.

Ubiquitination of both FANCD2 and FANCI forms a dual ubiquitin-locking mechanism needed for the ID complex function^57^. The ID complex is retained in EDF but lost in *Dikarya* (Fig. 2). Its absence in *Dikarya* might point to the lack of sensitivity to ICL in yeasts. It is also possible that other DNA repair systems are involved in ICL repair in this group of organisms.

A significant part of the FA pathway is the process of unhooking, covered mostly by the SLX1-SLX4 complex. Studies of SLX4 in humans have pointed out that loss of the UBZ domain (which recruits SLX4 to the site of ICL) in SLX4 would abolish FA-independent interactions, increasing the number of chromosomal aberrations^58^. The absence of the UBZ domain in members of the fungal kingdom leaves a gap in knowledge of the docking sites for SLX4 (Fig. 5). Interestingly, SLX4 genes were expressed neither in normal nor in stress conditions.

The transcriptomic analysis carried out in this study showed that SLX1 is highly upregulated in *Rhizophagus irregularis* associated with *Medicago truncatula* treated with strigolactone for 24 h.

This result suggests that SLX1 would partner with another inactive nuclease for substrate binding instead of SLX4 or that SLX4 would function differently in EDF than in mammals. The upregulation of FA core and repair genes during exposure of *R. delemar* and *R. microsporus* spores to phagocytosis (murine macrophages) leads to active catabolic pathways, nucleic acid binding, transcription and regulation via polymerase, indicating the involvement of genome maintenance pathways^41^. The association of AM fungi with plant roots triggers plant host defense mechanisms. We recognized downregulation of FA genes in *R. irregularis* in response to its colonization to *Lotus japonicus* roots at different phosphate concentrations of 20, 100, 300, and 500 μM (Fig. 3). It indicates that higher phosphate concentrations silence the FA gene expression, coupled with decrease in AM fungal colonization in *Glomeromycotina*.

Yeasts lack most of the FA pathway components and deal with the ICL threat by a replication and recombination-independent mechanism. In such a case, endonucleolytic unhooking depends on the Pso2 nuclease facilitated by Hrq1 helicase^51^, which is followed by translesion synthesis. This pathway may be responsible for the removal of ICL in FA pathway deficient cells^59^. We found Pso2 homologs in all classes of fungi, except *Microsporidia*. This opens up the possibility of an alternative for FA pathway in most of the fungal taxa. The presence of Pso2 is the basis of our model for ICL repair in *Saccharomycotina* and could, in general, be extended to *Dikarya* (Fig. 6).

FANCJ consists of N-terminal DEAH/DEAD box helicase and C-terminal Rad3 helicase domains. DEAD box helicase domain is missing from some of the FANCJ homologues in EDF. This might be a valid domain truncation or gene calling error missing the first exon in some of the taxa. Studies report that the DEAH/DEAD box domain binds to helix extensions, helps in target recognition and unwinding^60^. Lack of DEAD box helicase may reduce the catalytic activity of FANCJ in some of the EDF. However, it is also possible that Rad3 helicase domain alone can perform the necessary function.

The repair of ICL DNA damage undergoes a final step involving the vertebrate—specific DNA polymerase ν (DPOLN). DPOLN depletion results in low HR efficiency and an increased sensitivity to ICL causing agents^23^. With the occurrence of DPOLN in *Chytridiomycota*, we can speculate that this protein is of ancestral origin. A study by Huang and Cook points out that the rate of HR mediated DNA repair is not the same across fungal species^61^. It is likely that the uneven distribution of HR proteins across the fungal tree explains their observation.

The duplications observed in the phylogenetic trees of FA proteins are likely attributed to whole genome duplications or polyploidy in a given taxon (like in the case of *A. macrogynus*), a bigger genome size (240 Mb genome size in the case of *J. flammicorona*) and occurrence of genomes full of repetitive elements and potential functional diploidy (in case of *E. muscae*)^62^.

We observed previously unreported FA components to be conserved beyond fungi and mammals. *D. discoideum* is remarkably resistant to DNA damage^63^ and was previously reported to have a minimal FA pathway consisting of FANCD2, FANCI, FANCL, FANCM, FANCJ and UBE2T, components which possibly evolved in the last eukaryotic common ancestor^64^. We additionally found other FA members: MHF1, FANCO, ATR, FAN1, MUS81, XPF, ERCC1, REV1, REV3, USP1 and UAF1 (Fig. 2) in the social amoeba proteome (10.5281/zenodo.10911400, Supplementary Table S1), which supports the hypothesis of the early evolutionary origins of FA pathway in general. Another experimental study on *D. discoideum* proved that excision repair nuclease XPF was necessary to repair ICL^64^. Also, early animals including sponges possess a relatively complete FA pathway (Fig. 2).

Interestingly, in *Arthropods*, we observed a rather depleted FA protein repertoire. The presence of FA core binding proteins: FANCJ, FANCL, FANCM, with conserved functions of FANCD2 and FANCI in *D. melanogaster* points to a reduction of FA pathway in arthropods^38^. It was also found that in flies, each nuclease can act individually, without the need to form a complex during ICL repair^65^. Based on our results in nematodes, we may speculate there is a functional equivalent of the FA pathway for ICL repair due to the conservation of activation proteins, ID complex proteins, along with FANCJ, FANCM and FANCO, all of which are essential for pathway progression and ICL repair.

The co-occurrence of FA pathway genes in fungal genomes suggests their involvement in a common network. The genomic proximity in fungi is often linked with co-expression of genes which are needed by the cell to work in one process (Fig. 4). This non-random organization of FA genes is another confirmation of a possible functionally active FA repair system for ICL in EDF.

The localization of ID complex genes with ubiquitins, endonucleases and DNA repair genes stands true with the chronology of events (10.5281/zenodo.10911400, Supplementary Table S1). This is particularly visible in *Mucorales* and *Mortierellales*.

Despite the divergence of *Dikarya* around 650 million years ago^66^, the genomic proximity of FA components is preserved, particularly in *Ascomycota* (10.5281/zenodo.10911400, Supplementary Table S1). The co-localization of FA genes across the fToL implies an evolutionary pressure exerted on these genes to cluster together and maintain a functional pathway, perhaps for genome maintenance.

The existence of two FA pathway components fused in a single protein in most members of *Chytridiomycetes* (10.5281/zenodo.10911400, Supplementary Table S1), supports the existence of a gene fusion event around *Chytrid* evolution. However, the proteins are atypically long (1800–1900 aa) and there is only one transcript in EST database supporting such a gene model (XM_016756588.1: *Spizellomyces punctatus* DAOM BR117) with the presence of both protein domains in a single protein. This does not rule out the possibility of gene fusion since transcriptomic data for *Chrytidiomycota* is scarce. Regardless of the robustness of gene fusion, there is genetic proximity between the FANCM and XPF endonuclease in this taxon.

The lack of genomic proximity of FA core genes in *Glomeromycotina* can be attributed to high activity and proliferation of transposons in their genomes leading to genome reshuffling. The high interspecific diversity in the genomes of *Glomeromycotina* affects all known protein domains^67^.

Observed differences in FA conservation among *Opisthokonta* were summarized in the proposed model of FA repair pathway in *Glomero-* and *Chytridiomycota* groups (Fig. 6). We speculate the presence of a minimal FA pathway in early diverging fungi that promotes ICL repair. The expression of genes encoding proteins from NER, DSB and MMR pathways co-occurs with the expression of FA components, opening a possibility of coordination of these pathways to maintain genome stability in EDF. The presence of FANCE and FANCL in EDF, proteins that play a crucial role in mediating the monoubiquitination and formation of the ID complex, could be sufficient to kick-start an active FA pathway. The absence of the above proteins coupled with the absence of an ID complex in *Dikarya* could be the answer to insensitivity of ICL reported in yeasts, despite the discovery of FA homologs^4^. Numerous animal models for FA pathway have been developed and yet, no model has come up regarding the dynamics of FA pathway proteins in the fungal kingdom. Identification of putative homologs of FA proteins in EDF groups could pave the way for understanding the biology of ICL repair in the fungal tree of life.

Taken together, our study points to the ancient origin of the FA pathway and its conservation beyond mammals. At the same time, the pathway was shaped by massive gene loss in model animals and *Dikarya*. We hypothesize the existence of a minimal form of Fanconi Anemia pathway in the early diverging fungal lineages.

### Methods

FA pathway reference protein list was built based on keywords “fanconi”, “Fanconi Anemia”, “FANC” searched in the following databases: UniProt^68^, Reactome version 83^69^, Interpro^70^ and OrthoDB^71^. This resulted in a list of 40 FA proteins. The dataset of FA proteins comprises sequences from five model organisms: *Homo sapiens* (GCF_000001405.40), *Mus musculus* (GCF_000001635.27), *Caenorhabditis elegans* (GCF_000002985.6), *Dictyostelium discoideum* (GCA_000004695.1) and *Saccharomyces cerevisiae* acquired from UniProt database (10.5281/zenodo.10911400, Supplementary Table S1). These reference FA proteins were used as BLASTp queries against 183 fungal proteomes representing the diversity of sequenced fungi (e-value ≤ 1e−5) (10.5281/zenodo.10911400, Supplementary Table S1). For each protein, clustering in CLANS^72^ was performed to group the sets of fungal homologous sequences from potentially non-specific hits (*p* value of 1e−10, 1e−20 and 1e−30 and attraction exponent value of 2). Sequence groups were inspected for protein domain conservation and domain architecture using NCBI’s conserved domain database^73^ and PfamScan assessed by pfam_scan.pl^74^.

In order to verify the ancestral origin of the FA pathway, we also searched for the protein sets of five basal Opisthokonts [*Capsaspora owczarzaki* (GCF_000151315.2)^75^, *Sphaeroforma arctica* (GCF_001186125.1)^76^, *Fonticula alba* (GCF_000388065.1)^76^, *Salpingoeca rosetta* (GCF_000188695.1)^77^, *Monosiga brevicollis* (GCF_000002865.3)^78^]. Multiple sequence alignments were performed using MAFFT (v7.407) local alignment method^79^ with a maximum number of iterative refinements set to 100. Phylogenetic tree construction with maximum likelihood method was carried out using IQ-TREE (v1.6.9) with automated model selection, tree search and SH-aLRT test and ultrafast bootstrap^80^ (10.5281/zenodo.10911400, Supplementary Dataset SD1). A phylogenomic species tree was built with OrthoFinder^81^ (with mmseqs) for a set of 39 fungal taxa, 5 basal Opisthokonts and 5 Metazoa with *Dictyostelium discoideum* (GCA_000004695.1) and *Arabidopsis thaliana* (GCF_000001735.4) as outgroups. The trees were visualized and represented using the iTOL online tool^50^ (10.5281/zenodo.10911400, Supplementary Materials SM1, Fig. 5).

The expression of FA proteins was checked in publicly available EDF transcriptomes (10.5281/zenodo.10911400, Supplementary Table S1). The limited dataset contained transcriptomes obtained from pure cultures of *Mucor lusitanicus* MU402*, Jimgerdemannia flammicorona* AD002*, Umbelopsis isabellina* M6-22*, Endogone sp.* FLAS-F59071*, Lobosporangium transversale* NRRL 3116 along with transcriptomes of EDF obtained from diverse cultural environments: *Mucor lusitanicus* MS12*, Rhizopus microsporus* FP469*, Rhizopus delemar* 99-880*, Gigaspora rosea* DAOM 194757 and *Rhizophagus irregularis* DAOM 197198.

The data were downloaded from the ENA server^82^ as RNA-Seq fastq files. Their quality was checked using FASTQC (v0.11.8)^83^. The adapters were trimmed with fastp (v0.19.6) using default parameters^84^. The Hisat2 tool (v2.1.0)^85^ was used to align the fastq reads with the reference genome of the respective organism downloaded from Ensembl Fungi database (release 53, downloaded between May–June 2022). The SAM files obtained from the alignment were compressed into binary file format (BAM) using samtools (v1.10)^86^, and the aligned reads were counted using featureCounts (v1.6.3)^87^. The Transcript Per Million reads (TPM) values were calculated from the aligned reads using StringTie (v2.1.3b)^88^.

The differential expression analysis was carried out using the DESeq2 R package^89^ for protein-coding gene expression from condition-specific transcriptomic datasets. The *Padj* value was set to ≤ 0.05, and RNA-Seq reads were mapped on the FA protein-coding gene sequences. The log2fold change criteria [*downregulation* < *0* > *upregulation*] was used to determine the gene expression profiles. In addition to this, using the same approach, we also looked at the expression profiling of genes involved in DNA repair pathways of NER, DSB and mismatch repair (MMR).

To determine genomic localization of FA protein coding genes, contig names and coordinates were retrieved from NCBI using edirect tools. The genes occurring in a single contig were grouped together and their corresponding distances were calculated. Genomic distances up to 250 kb between locus pairs were grouped in colocalization pattern^48^, while distances greater than 250 kb were grouped under long-range colocalization patterns^49^. We tested the possibility of randomly finding two FA genes in a window of 250 kb by taking into account: average gene length, gene distance and gene number in a given genome. We applied a hypergeometric distribution test using values derived from GFF files. We were able to download 56/183 GFF files. The hypergeometric probabilities were calculated for each of the 56 GFF files and the standard P-value (*P* < 0.01) was chosen as the filtering criterion. The frequency of co-localization was computed and the results were visualized using Cytoscape v3.10.1^90^.

### Supplementary Information

Supplementary Information 1.

Supplementary Information 2.

Supplementary Figures.

Supplementary Information 3.

## Data Availability

All metadata processed in this study are deposited in zenodo: 10.5281/zenodo.10911400. All protein identifiers, genomic assemblies, transcriptomic datasets, hypergeometric test values are listed in Supplementary Table S1. The figures of phylogenetic trees for FA proteins are depicted in Supplementary Materials SM1 and available as a newick file format in Supplementary Dataset SD1. Supplementary Results provide a detailed commentary on the taxonomic distribution depicted in Fig. 2.

## Abbreviations

FA: Fanconi Anemia
ICL: Interstrand crosslinks
EDF: Early diverging fungi
DSB: Double-strand break
NER: Nucleotide excision repair
MMR: Mismatch repair
fToL: Fungal tree of life
MMS: Methyl methane sulfonate
HR: Homologous recombination
TLS: Translesion DNA synthesis
TPM: Transcripts per million
AM: Arbuscular mycorrhiza
UBZ: Ubiquitin binding Zinc finger
μM: Micromolar
