# Supplementary material for: A minimal Fanconi Anemia complex in early diverging fungi
Source: Sci Rep. 2024 Apr 30;14:9922. doi: 10.1038/s41598-024-60318-w (PMC11061109; doi:10.1038/s41598-024-60318-w)
Supplement: Supplementary file 2 — Supplementary Information 2. [file 41598_2024_60318_MOESM2_ESM.pdf]

****
****
ATR
****
****
(Mucor ambiguus_GAN03386.1:0.2075334290,((((((((((((Schizosaccharomyces
pombe_CAB40165.1:0.7034549391,Schizosaccharomyces japonicus
yFS275_EEB05355.1:0.5792658717)/1:1.0340565306,Pneumocystis carinii
B80_KTW27047.1:0.9455747016)/1:0.2596668923,(((((Blumeria graminis f. sp.
triticale_CAD6502046.1:0.5125333670,Sclerotinia sclerotiorum 1980 UF-
70_EDN95718.1:0.5444890849)/1:0.2196580132,(Neurospora crassa
OR74A_EDO65367.2:0.7748023740,(Trichoderma reesei
QM6a_EGR46540.1:0.5171162141,Verticillium dahliae
VdLs.17_EGY19868.1:0.5387432924)/1:0.1973094812)/1:0.3344395117)/1:0.3548377976,
(((Aspergillus nidulans FGSC A4_EAA61621.1:0.5751699168,Coccidioides immitis
RS_EAS29766.2:0.5228499701)/1:0.2499452399,Exophiala dermatitidis
NIH/UT8656_EHY59053.1:0.8877860016)/1:0.1921737198,(Zymoseptoria tritici
IPO323_EGP89591.1:1.1189184035,Bacidia
gigantensis_KAG8530063.1:0.7813846432)/1:0.1191853734)/1:0.1396239871)/
1:0.2900711187,Tuber melanosporum_CAZ80117.1:0.8730803282)/1:0.1308626779,
(Orbilia oligospora ATCC 24927_EGX52537.1:0.2639619628,Arthrobotrys
entomopaga_KAF3927530.1:0.2545737028)/1:0.9697723192)/1:0.3110839289)/
1:0.2027266280,(Candida albicans SC5314_AOW29815.1:1.5661693015,Saccharomyces
cerevisiae S288C_NP_009694.3:2.0229713865)/1:1.2021996932)/1:0.4114423286,
((Cryptococcus neoformans var. grubii H99_AFR95585.2:1.2294874963,
(((((Coprinopsis cinerea okayama7#130_EAU84196.2:1.0055133758,(Serpula lacrymans
var. lacrymans S7.9_EGO27933.1:0.4994518452,Amanita muscaria Koide
BX008_KIL70329.1:0.5071540806)/1:0.1575479562)/1:0.2304338825,Ramaria
rubella_KAF8592595.1:0.8908178702)/1:0.2389886923,Rhizoctonia
solani_QRW15803.1:1.2446815368)/1:0.1273369560,Dacryopinax
primogenitus_EJU06214.1:0.9980790165)/1:0.2270029358,Wallemia ichthyophaga EXF-
994_EOR00774.1:1.3512836518)/0.984:0.0976772760)/1:0.1527579742,(((Malassezia
globosa CBS 7966_EDP44639.1:1.0494310079,Ustilago maydis
521_KIS71202.1:0.8839445260)/1:0.2123459256,Tilletiaria anomala UBC
951_KDN44907.1:1.1793939909)/1:0.4268356168,((Puccinia graminis f. sp. tritici
CRL 75-36-700-3_EFP86122.1:0.6440062988,Melampsora larici-populina
98AG31_EGG02755.1:0.6297727162)/1:0.8828123746,Mixia osmundae IAM
14324_KEI39694.1:1.2504867143)/1:0.2110166476)/0.581:0.0852740122)/
1:0.4783105338)/0.998:0.0969673766,((((Batrachochytrium dendrobatidis
JAM81_EGF84391.1:1.6239111175,Dimargaris
cristalligena_RKP36998.1:1.5662776416)/0.986:0.1164397536,(((((Neocallimastix
sp. JGI-2020a_KAG4101513.1:0.0046821164,Neocallimastix
californiae_ORY26196.1:0.0011695231)/1:0.1205433196,Anaeromyces
robustus_ORX81535.1:0.1010521000)/0.958:0.0293410721,Piromyces
finnis_ORX56486.1:0.1115289017)/1:0.9082829184,Basidiobolus meristosporus CBS
931.73_ORX89906.1:0.6840630102)/1:0.1308380333,((((Spizellomyces punctatus DAOM
BR117_KND02053.1:0.0399580224,Spizellomyces sp.
'palustris'_TPX68250.1:0.0432274854)/1:0.5126898093,Powellomyces
hirtus_TPX55724.1:1.2377238463)/1:0.3971453754,(Rhizoclosmatium
globosum_ORY51967.1:0.4432212634,Chytriomyces
confervae_TPX75625.1:0.5166400309)/1:0.7487184186)/0.688:0.1091491060,
(Synchytrium microbalum_TPX33900.1:0.4065807118,Synchytrium
endobioticum_TPX39347.1:0.3991600926)/1:1.1819322736)/1:0.1807135185)/
1:0.0934242336)/0.928:0.0560592209,Gonapodya prolifera
JEL478_KXS21067.1:1.4588959157)/1:0.1330178979,((((((((Dissophora
ornata_KAF8940517.1:0.2007154857,Dissophora
globulifera_KAG0312109.1:0.2262034393)/1:0.1426534636,((Mortierella sp.
NVP85_KAF9364890.1:0.2650403676,Mortierella sp.
GBA43_KAG0246449.1:0.2510551237)/1:0.1929525260,Gamsiella
multidivaricata_KAG0364892.1:0.3036381802)/1:0.0463833117)/0.991:0.0363864730,
(((((Entomortierella lignicola_KAF8984393.1:0.0040594931,Haplosporangium sp. Z
27_KAF9205154.1:0.0055522547)/1:0.1336554238,(((Mortierella sp.
AD010_KAF9175721.1:0.0000020792,Mortierella sp.
AD011_KAF9403696.1:0.0000025009)/1:0.0177350357,Entomortierella

chlamydospora_KAG0002584.1:0.0142695536)/1:0.0366208070,Mortierella sp.
AD094_KAF9354246.1:0.0392489156)/1:0.0799411121)/1:0.0367166089,Mortierella sp.
AM989_KAF9115524.1:0.1454686319)/1:0.0364241587,Entomortierella
beljakovae_KAF9435330.1:0.1962849758)/1:0.1199106781,Lobosporangium
transversale_ORZ11293.1:0.3813184239)/1:0.0799883040)/1:0.1367144314,
(((Haplosporangium sp. Z 767_KAF9190175.1:0.0000022247,Haplosporangium sp. Z
11_KAF9192364.1:0.0000023085)/1:0.0282109278,Mortierella
polycephala_KAG0263666.1:0.0376946860)/1:0.4080070303,(Mortierella
antarctica_KAF9988600.1:0.2265124437,Mortierella sp.
GBA30_KAG0209441.1:0.1637086805)/1:0.3168416876)/1:0.0893699101)/1:0.0506631715,
((((Haplosporangium gracile_KAF8942568.1:0.0310746437,Linnemannia
schmuckeri_KAF9155074.1:0.0180156480)/1:0.0351081652,((Mortierella sp.
GBA39_KAF9141799.1:0.0146108403,(Mortierella
hygrophila_KAF9550394.1:0.0146520191,Linnemannia
hyalina_KAG9067389.1:0.0161617121)/1:0.0047999721)/1:0.0377802514,Linnemannia
elongata
AG-77_OAQ35638.1:0.0329474910)/1:0.0264169364)/1:0.0462395502,Linnemannia
gamsii_KAG0293331.1:0.1371710564)/1:0.1142463103,((Mortierella sp.
14UC_KAF9122997.1:0.0330857116,Linnemannia
zychae_KAF9908954.1:0.0476954787)/1:0.0653382583,(Linnemannia
exigua_KAG0273107.1:0.0410642606,Mortierella sp.
AD032_KAG0381061.1:0.0347811848)/1:0.0738384923)/1:0.1153284524)/1:0.1590805817,
((Mortierella sp. AD031_KAF9084154.1:0.0003852089,Mortierella sp.
NVP41_KAG0206912.1:0.0012436787)/0.999:0.0018205156,Mortierella sp.
GBA35_KAF9100333.1:0.0043752592)/1:0.2208059933)/1:0.3802752072)/1:0.1273578410,
Lunasporangiospora
selenospora_KAF9584971.1:0.5936770422)/1:0.0659660774,Gryganskiella
cystojenkinii_KAG0055729.1:0.7323089725)/1:0.0875020456,((((Haplosporangium
bisporale_KAF9025840.1:0.0005276932,Podila verticillata NRRL
6337_KFH65039.1:0.0010418554)/1:0.1055973554,(Podila
horticola_KAF9311094.1:0.0399414571,(Podila
clonocystis_KAG0013511.1:0.0415039514,((Podila
clonocystis_KAG0025132.1:0.0360783496,Podila
epicladia_KAG0092823.1:0.0361896509)/0.826:0.0094773870,Podila
minutissima_KAG0351390.1:0.0278995878)/1:0.0104821431)/1:0.0111876354)/
1:0.0848103764)/1:0.2174038073,Podila
epigama_KAF9427653.1:0.5169478707)/0.954:0.0758259597,Podila
humilis_KAG0349509.1:0.5792243819)/1:0.3337307249)/1:0.3069217940,Actinomortiere
lla ambigua_KAG0264158.1:1.2639028903)/1:1.0046313523)/1:0.1314712080)/
1:0.0914726942,((((((((((Blumeria graminis f. sp.
triticale_CAD6505980.1:0.8827423836,Sclerotinia sclerotiorum 1980 UF-
70_EDO00751.1:0.5937485002)/1:0.1883704927,Verticillium dahliae
VdLs.17_EGY14732.1:0.7315275754)/1:0.3527538988,((Aspergillus nidulans FGSC
A4_EAA65357.1:0.6878441901,Coccidioides immitis
RS_KJF61358.1:0.5155045126)/1:0.2396279430,Exophiala dermatitidis
NIH/UT8656_EHY53217.1:1.2191956360)/1:0.1630581633)/1:0.6956296922,Pneumocystis
carinii B80_KTW28981.1:1.6346066966)/1:0.3771803470,Yarrowia lipolytica
CLIB122_CAG80568.1:3.4146704659)/0.864:0.1792487746,(Cryptococcus neoformans
var. grubii H99_AFR96091.2:0.0000024151,Cryptococcus neoformans var. grubii
H99_AGV14486.1:0.0000021868)/1:2.4322614677)/0.998:0.1635737077,
(((Neocallimastix sp. JGI-2020a_KAG4102476.1:1.4575396775,Basidiobolus
meristosporus CBS 931.73_ORX90217.1:1.3031757302)/1:0.3760503934,(Spizellomyces
punctatus DAOM BR117_KNC99195.1:0.0580833911,Spizellomyces sp.
'palustris'_TPX67003.1:0.0493663770)/1:1.3320202659)/1:0.2370088932,
(Rhizoclosmatium globosum_ORY44473.1:0.6483968906,Chytriomyces
confervae_TPX75368.1:0.6347852469)/1:1.4646284152)/1:0.4465257583)/
1:1.8871021585,(Encephalitozoon intestinalis ATCC
50506_ADM11106.1:4.1194611241,Mitosporidium
daphniae_KGG52004.1:3.1171738660)/0.927:0.2329652165)/0.999:0.2207149940,Rozella
allomycis CSF55_EPZ33635.1:2.1140353380)/0.999:0.1873251851,(((Allomyces
macrogynus ATCC 38327_KNE56447.1:0.0000029282,Allomyces macrogynus ATCC
38327_KNE56448.1:0.0000021349)/1:1.3854029242,Catenaria anguillulae
PL171_ORZ38038.1:1.3629837514)/1:1.5152653034,Smittium
culicis_OMJ14013.1:2.2384988956)/0.91:0.1761422112)/0.992:0.0793419256,

((((sp_Q13535_ATR_HUMAN:0.0614467346,sp_Q9JKK8_ATR_MOUSE:0.0597514663)/
1:0.8713933270,Pomacea
canaliculata_PVD19877.1:0.9878758726)/1:0.3155026287,Amphimedon
queenslandica_XP_019850314.1:1.6759504076)/1:0.2623733046,Drosophila
melanogaster_NP_523369.2:2.5764220897)/1:0.5334583590)/0.996:0.0815533536,Thamno
cephalis
sphaerospora_RKP09385.1:1.7241966881)/0.804:0.0725897147)/1:0.8010466047,
(Umbelopsis isabellina_KAG2174128.1:0.5307723311,Umbelopsis
vinacea_KAG2180550.1:0.5984637341)/1:1.3911608640)/1:0.5199864196,(Hesseltinella
vesiculosa_ORX44443.1:1.1440813577,Absidia
repens_ORZ05245.1:1.3117356007)/1:0.8647364776)/0.958:0.1341092268,
(((Apophysomyces ossiformis_KAF7722872.1:0.2023111439,((Apophysomyces sp.
BC1015_KAG0169780.1:0.0000020852,Apophysomyces sp.
BC1021_KAG0179297.1:0.0034624014)/0.131:0.0000024995,Apophysomyces sp.
BC1034_KAG0189763.1:0.0000021222)/1:0.2391456308)/1:0.9715037293,Syncephalastrum
racemosum_ORY98518.1:1.6657180810)/1:0.1201098856,Mucor
circinatus_KAG2216150.1:1.2546985268)/0.88:0.1021973299)/1:0.8067280336,
(Rhizopus delemar RA 99-880_EIE82128.1:0.4191605427,(Rhizopus microsporus ATCC
52813_PHZ08210.1:0.0322202032,Rhizopus
azygosporus_RCH89120.1:0.0004860729)/1:0.5677782937)/1:0.6553795556)/
1:0.2689220450,(Mucor saturninus_KAG2194555.1:0.7272102384,Thamnidium
elegans_KAG2234017.1:0.8486368460)/1:0.4235645583)/1:0.2884348696,Choanephora
cucurbitarum_OBZ87784.1:1.5300864880)/1:0.5001204953,(Parasitella
parasitica_CEP14753.1:0.2588941987,Mucor
plumbeus_KAG2215177.1:0.2920619796)/1:0.1840487918)/1:0.5151785451,(Mucor
lusitanicus_KAF1798393.1:0.3960173380,Mucor
lusitanicus_KAF1806238.1:0.0170573260)/1:0.2243802318);

****
****
CENPS
****
****
(Mucor ambiguus_GAN09845.1:0.0627346441,((((((((((Rhizophagus
clarus_GES89132.1:0.0857887354,(Rhizophagus irregularis DAOM 181602=DAOM
197198_POG76125.1:0.0112417972,Rhizophagus
diaphanus_RGB42141.1:0.0042521100)/1:0.0553537363)/0.968:0.0430996038,Glomus
cerebriforme_RIA92602.1:0.0706166009)/0.738:0.1176909359,(Gigaspora
margarita_KAF0504808.1:0.0000025914,Gigaspora
rosea_RIB08793.1:0.0309896872)/1:0.6591493826)/1:0.5704370372,(((((Dissophora
ornata_KAF8930445.1:0.3052369981,Podila
epigama_KAF9414437.1:0.2390810284)/0.989:0.1233681427,(Mortierella sp.
AD010_KAF9172271.1:0.0063647590,(Mortierella sp.
AD094_KAF9352856.1:0.0530069741,Entomortierella
chlamydospora_KAG0000849.1:0.0076277906)/1:0.0389338408)/1:0.1072219768)/
1:0.1089077083,((((Mortierella sp. 14UC_KAF9126006.1:0.0083026700,(Linnemannia
zychae_KAF9909444.1:0.1317263391,(Linnemannia exigua_KAG0270810.1:0.1476575525,
(Mortierella sp. AD032_KAG0375234.1:0.0109700729,Syncephalis
pseudoplumigaleata_RKP23070.1:0.8966530357)/0.808:0.0202898246)/1:0.2027698417)/
1:0.2003450489)/0.999:0.0623495832,(Linnemannia
gamsii_KAG0285714.1:0.0536210850,Linnemannia elongata AG-
77_OAQ35186.1:0.0450782694)/0.942:0.0418734483)/1:0.1155398821,(Mortierella sp.
NVP85_KAF9366388.1:0.0491174303,Modicella
reniformis_KAG0000688.1:0.0181253565)/1:0.1281303791)/0.498:0.0337511185,Mortier
ella sp.
GBA30_KAG0211812.1:0.0900373043)/0.938:0.0455424688)/0.965:0.0312094616,Mortiere
lla polycephala_KAG0254188.1:0.0864189294)/0.197:0.0116208087,Podila
verticillata NRRL 6337_KFH63577.1:0.2804975058)/1:0.3896570237,Actinomortierella
ambigua_KAG0261400.1:0.1366220174)/1:0.3358694694)/0.494:0.0455294239,
((((((((Blumeria graminis f. sp. triticale_CAD6502011.1:0.5976646371,((Orbilia
oligospora ATCC 24927_EGX43039.1:0.1538217945,Drechslerella
brochopaga_KAF3915630.1:0.0732837698)/0.221:0.0150115184,Saccharomyces
cerevisiae_NP_076910.1:1.8987226853)/1:0.4029748123)/0.812:0.0906312516,(Tuber
melanosporum_CAZ81356.1:0.2737087185,((Neurospora crassa

OR74A_EAA32120.2:0.1602089939,(Trichoderma reesei
QM6a_EGR52138.1:0.1617215229,Verticillium dahliae
VdLs.17_EGY17651.1:0.3138241679)/0.993:0.1125739131)/1:0.2272132627,Sclerotinia
sclerotiorum 1980
UF-70_EDN91003.1:0.2837326706)/0.986:0.1108700002)/0.965:0.1262713185)/
0.993:0.2017001840,Exophiala dermatitidis
NIH/UT8656_EHY59286.1:0.6056499903)/0.126:0.0000023095,Zymoseptoria tritici
IPO323_EGP92241.1:0.7747421999)/1:0.1811608084,(Pneumocystis carinii
B80_KTW30176.1:0.4972738342,Absidia
repens_ORZ18537.1:0.9933388174)/0.999:0.3013870883)/0.126:0.0000020688,
(((Candida albicans SC5314_AOW26899.1:1.1499451353,Mixia osmundae IAM
14324_KEI41410.1:0.6367354261)/0.92:0.1882840260,(Puccinia graminis f. sp.
tritici CRL 75-36-700-3_EFP74157.1:0.5123636304,Melampsora larici-populina
98AG31_EGG03272.1:0.4123913207)/1:0.6813375109)/0.991:0.2639959917,(Coccidioides
immitis RS_EAS34683.3:1.1278040280,(Schizosaccharomyces japonicus
yFS275_EEB05910.1:0.0851186158,Schizosaccharomyces
pombe_NP_596235.2:0.1664607602)/1:0.8299712258)/0.964:0.2083551708)/
0.965:0.1180825189)/1:0.1003430037,(Yarrowia lipolytica
CLIB122_CAR64283.1:1.5013620820,Neolecta irregularis DAH-
3_OLL26884.1:0.8029120157)/0.778:0.2094032904)/1:0.1559448969,(((Spizellomyces
punctatus DAOM BR117_KND03612.1:0.0138893776,Spizellomyces sp.
'palustris'_TPX70703.1:0.1635889543)/1:0.3707794947,(Rhizoclosmatium
globosum_ORY51605.1:0.3601553723,(Chytriomyces
confervae_TPX58938.1:0.1483965559,Chytriomyces
confervae_TPX63410.1:0.0194043347)/1:0.7648919050)/1:0.6040352715)/
1:0.1988321011,((((((Allomyces macrogynus ATCC
38327_KNE62640.1:0.0015100851,Allomyces macrogynus ATCC
38327_KNE62641.1:0.4244919057)/0.274:0.0090226528,Allomyces macrogynus ATCC
38327_KNE65671.1:0.0874161565)/1:0.4984701800,Catenaria anguillulae
PL171_ORZ30379.1:0.8031618130)/1:0.4371108106,Caenorhabditis
elegans_NP_001254386.1:0.9452392209)/0.846:0.1211762635,(((Homo
sapiens_NP_954988.1:0.1491953723,Mus
musculus_NP_081539.1:0.1855227543)/1:0.5751270060,Amphimedon
queenslandica_XP_003386995.1:0.7288023789)/0.972:0.1788774543,Pomacea
canaliculata_PVD36969.1:0.8987950496)/0.494:0.0562542329)/1:0.1436717615,Gonapod
ya prolifera
JEL478_KXS12335.1:0.7787249862)/0.673:0.0435798615)/0.999:0.0488132043)/
0.412:0.0289465874,(Entomophthora muscae_KAF7751029.1:0.6604556691,Conidiobolus
coronatus NRRL
28638_KXN66422.1:0.9271963837)/0.99:0.2414091297)/0.837:0.0567670681,
(Batrachochytrium dendrobatidis JAM81_EGF80430.1:0.9556572442,(Linderina
pennispora_ORX73231.1:0.3012221869,Coemansia reversa NRRL
1564_PIA16112.1:0.4216861394)/1:0.5461287232)/0.884:0.2583115476)/
0.887:0.0663777423)/0.995:0.1784324654,Phycomyces blakesleeanus NRRL
1555_OAD66028.1:0.6861051334)/0.803:0.0920411948,Syncephalastrum
racemosum_ORY93417.1:0.7684643013)/0.769:0.0843316345,(Umbelopsis
vinacea_KAG2179595.1:0.3226613220,Umbelopsis
isabellina_KAG2185905.1:0.0000025855)/1:0.5238713278)/0.793:0.0813736332,Mucor
circinatus_KAG2225422.1:0.4855586934)/0.972:0.1278601215,(Absidia
glauca_SAL95749.1:0.3528981455,Absidia
repens_ORZ17860.1:0.6360654380)/1:0.6878118193)/0.699:0.0808498142,(((Rhizopus
delemar RA 99-880_EIE92310.1:0.3619517143,(Rhizopus microsporus ATCC
52813_PHZ16503.1:0.0000022064,Rhizopus
azygosporus_RCH90432.1:0.0741988792)/1:0.1545230023)/1:0.3360137060,Thamnidium
elegans_KAG2229681.1:0.3787966611)/0.602:0.0835007247,Choanephora
cucurbitarum_OBZ87936.1:0.3631528507)/0.819:0.0710785334)/1:0.1875649847,
(Parasitella parasitica_CEP19315.1:0.3455083288,Mucor
plumbeus_KAG2212801.1:0.1886752420)/0.768:0.0373152230)/0.595:0.0568455894,Mucor
lusitanicus_KAF1798576.1:0.1341692895);

****
****

CENPX

****

****
(Rhizophagus clarus_GES86586.1:0.0422170272,((((((((Absidia
glauca_SAM08177.1:0.2821591235,Absidia
repens_ORZ20697.1:0.4352071575)/0.999:0.4476533621,(Hesseltinella
vesiculosa_ORX49089.1:0.3745694457,Caenorhabditis
elegans_NP_501398.1:3.5114706179)/0.999:0.7035360636)/0.969:0.2368418684,
(Melampsora larici-populina 98AG31_EGG05239.1:1.7422255321,Entomophthora
muscae_KAF7758127.1:1.8228189242)/0.68:0.2926038282)/0.145:0.0066955533,
((((Rhizopus delemar RA 99-880_EIE82837.1:0.2004711425,Rhizopus
azygosporus_RCH99043.1:0.5603047104)/1:0.6368545107,Syncephalastrum
racemosum_ORY93209.1:0.8885163297)/0.427:0.0793859774,(((Mucor
lusitanicus_KAF1799744.1:0.5218649651,Mucor
plumbeus_KAG2206193.1:0.2990648773)/0.92:0.1569157851,Choanephora
cucurbitarum_OBZ82774.1:0.9805007900)/0.525:0.1278991082,(Mucor
saturninus_KAG2192325.1:1.9679015469,Thamnidium
elegans_KAG2234512.1:0.5559757433)/0.956:0.3532463372)/0.919:0.2023612422)/
1:0.3436824125,((Mucor circinatus_KAG2223131.1:0.9990831295,(Rhizoclosmatium
globosum_ORY51358.1:0.4504133213,Chytriomyces
confervae_TPX78026.1:0.6136077190)/1:1.3043828793)/0.125:0.0000029343,Phycomyces
blakesleeanus NRRL
1555_OAD68882.1:1.4289004716)/0.86:0.4053190286)/0.967:0.2033596710)/
0.999:0.2306385667,(((((((((((Haplosporangium
gracile_KAF8929660.1:0.0216557802,Linnemannia
schmuckeri_KAF9148507.1:0.0165117958)/0.993:0.0314301563,(((Mortierella sp.
GBA39_KAF9131213.1:0.0000023686,Mortierella
hygrophila_KAF9544877.1:0.0171043568)/0:0.0000026553,Linnemannia
hyalina_KAG9072522.1:0.0102771205)/0.987:0.0250545789,Linnemannia elongata AG-
77_OAQ24099.1:0.0379557268)/0.999:0.0501054370)/1:0.2367669064,Linnemannia
gamsii_KAG0290685.1:0.1425615959)/0.991:0.0447612539,((Mortierella sp.
14UC_KAF9130353.1:0.0423776493,Linnemannia
zychae_KAF9910780.1:0.0539157934)/1:0.0468637677,(Linnemannia
exigua_KAG0281456.1:0.0721712122,Mortierella sp.
AD032_KAG0380722.1:0.0320250323)/1:0.0760662441)/1:0.0934539057)/1:0.1297389969,
(Mortierella sp. AD031_KAF9088336.1:0.0000023575,Mortierella sp.
NVP41_KAG0218095.1:0.0053086646)/1:0.1817299267)/1:0.3042371934,
((((Haplosporangium sp. Z 11_KAF9177017.1:0.0000024876,Haplosporangium sp. Z
767_KAF9194668.1:0.0000027403)/0.966:0.0184923540,Mortierella
polycephala_KAG0266589.1:0.0450326130)/1:0.5372486436,(Mortierella
antarctica_KAF9989753.1:0.2609389432,Mortierella sp.
GBA30_KAG0205827.1:0.2812668266)/0.889:0.0784592636)/1:0.1203043203,Gryganskiell
a
cystojenkinii_KAG0046410.1:0.2696978969)/1:0.0951840691)/0.403:0.0253854865,Diss
ophora globulifera_KAG0317254.1:0.4274686193)/0.914:0.0806682925,((Dissophora
ornata_KAF8930711.1:0.1734012976,Modicella
reniformis_KAF9951147.1:0.0799565347)/0.725:0.1215211994,(((((Entomortierella
lignicola_KAF8985838.1:0.0163159334,Haplosporangium sp. Z
27_KAF9176959.1:0.0000023181)/1:0.2531033618,Entomortierella
beljakovae_KAF9436336.1:0.1884643670)/0.815:0.0326135837,(Mortierella sp.
AD010_KAF9169763.1:0.0930248708,(Mortierella sp.
AD094_KAF9354990.1:0.0347529071,Entomortierella
chlamydospora_KAF9995849.1:0.0124676418)/0.994:0.0250414170)/1:0.0882023347)/
0.903:0.0663290411,Lobosporangium
transversale_ORZ28650.1:0.5489968205)/0.986:0.0799734460,((Mortierella sp.
NVP85_KAF9362793.1:0.1227001945,Mortierella sp.
GBA43_KAG0213116.1:0.2116246692)/1:0.2927793261,Gamsiella
multidivaricata_KAG0366028.1:0.2084621919)/0.993:0.0729913927)/1:0.1466643544)/
0.901:0.0616555093)/0.783:0.1095957245,Lunasporangiospora
selenospora_KAF9584776.1:0.6014049465)/0.951:0.0781783239,(Actinomortierella
wolfii_KAG0238001.1:0.1866205972,Actinomortierella
ambigua_KAG0249865.1:0.1836334600)/1:0.6485587538)/1:0.2438318752,Podila
epigama_KAF9426970.1:0.2791438759)/0.982:0.1708837749,((((((Haplosporangium
bisporale_KAF9009163.1:0.0000023930,Podila verticillata NRRL
6337_KFH68494.1:0.0000028692)/1:0.1921621912,Podila
clonocystis_KAG0020060.1:0.0323306628)/1:0.0309876671,Podila

epicladia_KAG0094925.1:0.0598810049)/0.797:0.0093421142,Podila minutissima_KAG0346326.1:0.0382012882)/0.275:0.0058208814,Podila horticola_KAF9321049.1:0.1083229619)/0.994:0.1382995709,Podila humilis_KAG0330786.1:0.6438522501)/0.18:0.0108853294)/1:0.8026081690,((Allomyces macrogynus ATCC 38327_KNE60531.1:0.9154947244,Catenaria anguillulae PL171_ORZ38204.1:0.7573235828)/1:0.9021321916,(Gonapodya prolifera JEL478_KXS18439.1:1.3236623750,Basidiobolus meristosporus CBS 931.73_ORX88873.1:0.8408192352)/0.248:0.0679111393)/0.997:0.3538346029)/0.28:0.0411966659)/0.966:0.1749211688,(Umbelopsis isabellina_KAG2173662.1:0.1929547241,Umbelopsis vinacea_KAG2186571.1:0.0268037653)/1:0.8322567576)/0.683:0.1681589409,((((Neurospora crassa OR74A_EAA33966.2:0.7811922981,(Arthrobotrys entomopaga_KAF3913125.1:0.1296918863,Drechslerella brochopaga_KAF3926140.1:0.1956174720)/1:1.0690163887)/1:0.7096689442,(((Schizosaccharomyces japonicus yFS275_EEB07375.1:0.1906492341,Schizosaccharomyces pombe_NP_588439.1:0.4784400475)/1:1.4024882672,(Pneumocystis carinii B80_KTW28930.1:1.1074207239,Saccharomyces cerevisiae_NP_878060.1:3.4056732203)/0.384:0.2568364508)/0.489:0.1843535237,Neolecta irregularis DAH-3_OLL22519.1:1.0842861209)/0.822:0.2888512031)/1:0.3180093487,((Homo sapiens_NP_659435.2:0.5568239355,Mus musculus_NP_001334540.1:0.0083028308)/1:1.0555020558,Amphimedon queenslandica_XP_019849221.1:1.2558232931)/0.999:0.7198760098)/0.703:0.1541773679,Conidiobolus coronatus NRRL 28638_KXN66206.1:2.1764160748)/0.932:0.2279300793)/1:0.7756581791,Gigaspora margarita_KAF0513132.1:0.8410363379)/0.995:0.3818736713,Glomus cerebriforme_RIA86267.1:0.1055962056)/0.835:0.0685043738,(Rhizophagus irregularis DAOM 181602=DAOM 197198_POG66696.1:0.0202967369,Rhizophagus diaphanus_RGB40196.1:0.1362153236)/1:0.0945902926);

****

DPOLN

****

(Mortierella sp. GBA39_KAF9133876.1:1.0263369518,(Mortierella sp. GBA39_KAF9134845.1:0.7862621696,(Podila clonocystis_KAG0018810.1:0.2411104866,((Apophysomyces sp. BC1015_KAG0161972.1:0.0000029962,Apophysomyces sp. BC1034_KAG0194724.1:0.0000023241)/1:0.2373810492,Linnemannia gamsii_KAG0293379.1:0.2846111741)/0.994:0.2313993692)/1:0.6452525730)/0.462:0.1479835414,((((((Spizellomyces punctatus DAOM BR117_KND02903.1:0.0483910616,Spizellomyces sp. 'palustris'_TPX62729.1:0.0656143774)/1:0.5886765918,Powellomyces hirtus_TPX57778.1:0.7346512773)/1:0.2187624398,Blyttiomyces helicus_RKO83809.1:0.6888812373)/0.735:0.0959490138,Blyttiomyces helicus_RKO84801.1:0.3170706739)/1:0.2308648085,Gonapodya prolifera JEL478_KXS16810.1:0.9757098254)/0.912:0.1119918309,(Synchytrium microbalum_TPX37004.1:0.2849976516,Synchytrium endobioticum_TPX50567.1:0.2437497226)/1:0.5927085071)/1:0.8125404858,(Homo sapiens_NP_861524.2:0.2289971970,Mus musculus_NP_862905.1:0.1667851117)/1:1.9572818223)/1:1.0669240180);

****

EME1

****

(Rhizophagus clarus_GES74087.1:0.0768922512,((((((((Absidia glauca_SAM02303.1:0.5584495318,Absidia repens_ORZ20795.1:0.5087838266)/1:0.6104812184,((Apophysomyces ossiformis_KAF7725982.1:0.6118008149,Phycomyces blakesleeanus NRRL 1555_OAD73828.1:0.6561616064)/0.999:0.1120670690,((Mucor circinatus_KAG2221402.1:0.7248437127,Rhizopus microsporus ATCC

52813_PHZ14561.1:1.2167002624)/0.581:0.1164821855,Syncephalastrum
racemosum_ORZ03210.1:0.9221624687)/0.97:0.1162497021)/0.991:0.1218380340)/
1:0.4909736882,(Bifiguratus adelaidae_OZJ05768.1:1.3721746508,((Jimgerdemannia
flammicorona_RUP43285.1:0.2399809439,Jimgerdemannia
flammicorona_RUP50704.1:0.6660417814)/0.768:0.0730224774,Jimgerdemannia
flammicorona_RUP50114.1:0.1181547688)/1:1.8324997129)/0.987:0.2726436640)/
0.944:0.0825850216,((((((((Malassezia globosa CBS
7966_EDP44141.1:1.4647190546,Ustilago maydis
521_KIS70129.1:1.2902654517)/1:0.4039990392,Entomophthora
muscae_KAF7741469.1:2.4223259082)/0.425:0.2371928686,Paramicrosporidium
saccamoebae_PJF17826.1:2.6730376604)/0.855:0.1413053046,(((((Homo
sapiens_NP_689676.2:0.1897419864,Mus
musculus_NP_808420.1:0.3089997857)/1:1.0175556513,Amphimedon
queenslandica_XP_019862746.1:1.3355542566)/0.974:0.1705478876,Pomacea
canaliculata_XP_025099763.1:1.6244378870)/0.876:0.1763354506,Drosophila
melanogaster_NP_610611.1:2.6337655088)/0.997:0.3167351184,(Monosiga brevicollis
MX1_XP_001747395.1:1.3203383658,Salpingoeca
rosetta_XP_004998646.1:1.0452386540)/1:0.6244988876)/1:0.3990816730)/
0.903:0.0693062898,(Coemansia reversa NRRL
1564_PIA14598.1:1.8190173276,Dimargaris
cristalligena_RKP36081.1:1.7038854265)/1:0.4129640489)/0.847:0.1236758483,Allomy
ces macrogynus ATCC 38327_KNE55588.1:1.8610201208)/0.558:0.0726772753,
(Thamnocephalis sphaerospora_RKP05774.1:0.8049730654,Syncephalis
pseudoplumigaleata_RKP26398.1:1.2034222120)/1:0.9720828401)/1:0.1774313432,
((((Orbilia oligospora ATCC 24927_EGX45981.1:0.4645099906,Drechslerella
brochopaga_KAF3924491.1:0.3238437427)/1:0.9169770946,Coccidioides immitis
RS_KJF60424.1:1.3074445812)/0.905:0.2160463901,Pneumocystis carinii
B80_KTW31514.1:1.7158135470)/1:0.2636893124,((((((Haplosporangium
bisporale_KAF8928400.1:0.0033553085,Podila verticillata NRRL
6337_KFH61956.1:0.0073053484)/1:0.1678082672,((Podila
horticola_KAF9316133.1:0.1427534990,Podila
clonocystis_KAG0013941.1:0.1406384470)/1:0.0325290267,(Podila
epicladia_KAG0095201.1:0.1086225002,Podila
minutissima_KAG0346773.1:0.0469452497)/1:0.0505812516)/1:0.0897376454)/
1:0.2147713032,Podila
humilis_KAG0341931.1:0.7158461181)/1:0.4508280890,Gryganskiella
cystojenkinii_KAG0044786.1:1.0091796352)/0.812:0.1155329790,
((((((Haplosporangium gracile_KAF8943063.1:0.0762905579,Linnemannia
schmuckeri_KAF9153982.1:0.0566262625)/1:0.0600002213,(Mortierella sp.
GBA39_KAF9144632.1:0.0307879862,(Mortierella
hygrophila_KAF9548733.1:0.0436423956,Linnemannia
hyalina_KAG9064818.1:0.0409451041)/0.828:0.0065164439)/1:0.0946975420)/
1:0.0341719578,Linnemannia gamsii_KAG0288267.1:0.1942147632)/1:0.1265150877,
((Mortierella sp. 14UC_KAF9124098.1:0.0871395980,Linnemannia
zychae_KAF9911798.1:0.1511189225)/1:0.1297025220,(Linnemannia
exigua_KAG0273318.1:0.0767477109,Mortierella sp.
AD032_KAG0367393.1:0.0722841667)/1:0.0848826077)/1:0.1099535986)/1:0.5577746951,
Modicella reniformis_KAF9940289.1:0.6233607022)/0.998:0.1323390283,
((Haplosporangium sp. Z 11_KAF9181252.1:0.0011752001,Haplosporangium sp. Z
767_KAF9183603.1:0.0000024283)/1:0.0910229387,Mortierella
polycephala_KAG0250925.1:0.0695785025)/1:0.4892953102)/0.978:0.0754731963,Lunasp
orangiospora
selenospora_KAF9582611.1:1.0777878623)/0.988:0.1063599008)/0.977:0.1080288936,
(Actinomortierella wolfii_KAG0227374.1:0.2442817137,Actinomortierella
ambigua_KAG0259118.1:0.4697811957)/1:0.8431711778)/1:0.5517106312)/
0.937:0.1186602052)/0.994:0.1005130100,((((Batrachochytrium dendrobatidis
JAM81_EGF82095.1:1.6141625705,(Spizellomyces punctatus DAOM
BR117_KND04284.1:0.0845683495,Spizellomyces sp.
'palustris'_TPX69605.1:0.0789488011)/1:1.1107906331)/0.957:0.2150716514,Chytriom
yces confervae_TPX77057.1:1.5996695959)/0.999:0.1644241524,Gonapodya prolifera
JEL478_KXS11888.1:1.6370133874)/0.56:0.0660577394,((((Neocallimastix sp. JGI-
2020a_KAG4089999.1:0.0000026926,Neocallimastix
californiae_ORY56272.1:0.0035039820)/1:0.4105595094,Anaeromyces
robustus_ORX76893.1:0.3106484767)/0.931:0.0866188732,Piromyces

finnis_ORX51757.1:0.2420648535)/1:1.8202929695,Olpidium
bornovanus_KAG5458601.1:1.8219847562)/0.989:0.2931120429)/0.992:0.1068808434)/
1:0.1815400557)/0.999:0.1913507327,Basidiobolus meristosporus CBS
931.73_ORY07633.1:1.4135446864)/0.898:0.1527407485,(Jimgerdemannia
flammicorona_RUP46178.1:0.3143550248,Endogone sp. FLAS-
F59071_RUS21004.1:0.3519278547)/1:0.6054443411)/1:0.3457414016,Geosiphon
pyriformis_KAG9290570.1:0.6430694833)/1:0.2353994953,((Gigaspora
margarita_KAF0407987.1:0.0355383211,Gigaspora
rosea_RIB19150.1:0.1880940851)/1:0.3171731391,Diversispora
epigaea_RHZ46109.1:0.4788049626)/1:0.1325031928)/1:0.4960933874,Glomus
cerebriforme_RIA93270.1:0.1984561902)/1:0.0786813880,Rhizophagus irregularis
DAOM 181602=DAOM 197198_POG73572.1:0.0531154817);

****
ERCC1
****
(Mucor ambiguus_GAN07378.1:0.0969835722,((((((((((Rhizophagus
clarus_GES98699.1:0.0304240009,((Rhizophagus irregularis DAOM 181602=DAOM
197198_POG66328.1:0.0033771603,Rhizophagus
diaphanus_RGB34242.1:0.0142492791)/0.793:0.0223003906,Glomus
cerebriforme_RIA88379.1:0.2466317085)/0.741:0.0334921604)/1:0.4534619831,
((Gigaspora margarita_KAF0442096.1:0.0107144181,Gigaspora
rosea_RIB08303.1:0.0014906323)/1:0.2158678129,Diversispora
epigaea_RHZ57157.1:0.4139635908)/1:0.1489847637)/0.993:0.1189368072,Geosiphon
pyriformis_KAG9293063.1:0.5259741462)/1:0.9204077255,
((((((((Schizosaccharomyces pombe_CAA20735.1:0.4316638685,Schizosaccharomyces
japonicus yFS275_EEB07724.2:0.6578210923)/1:0.9953873985,(Pneumocystis carinii
B80_KTW28575.1:1.4084720904,Neolecta irregularis DAH-
3_OLL24669.1:1.0006218870)/0.637:0.1515496542)/0.991:0.2256776790,
((((((Blumeria graminis f. sp. triticale_CAD6503600.1:0.7531162989,Sclerotinia
sclerotiorum 1980 UF-70_EDO00533.1:0.2765932257)/1:0.1524941760,(Neurospora
crassa OR74A_EAA31095.1:0.6885967458,(Trichoderma reesei
QM6a_EGR51574.1:0.6620040251,Verticillium dahliae
VdLs.17_EGY20454.1:0.3026641737)/0.998:0.1157645211)/0.997:0.1295661856)/
1:0.2456157763,Zymoseptoria tritici
IPO323_EGP91781.1:0.8369977502)/0.96:0.1007281359,Bacidia
gigantensis_KAG8533466.1:0.4808379229)/0.998:0.1601964466,(Aspergillus nidulans
FGSC A4_EAA60492.1:0.4506104096,Coccidioides immitis
RS_EAS33174.3:0.8065607454)/1:0.4345891442)/0.484:0.0721656644,Exophiala
dermatitidis NIH/UT8656_EHY55690.1:0.5012033562)/1:0.3656971887,((Orbilia
oligospora ATCC 24927_EGX45033.1:0.3414227850,Drechslerella
brochopaga_KAF3913044.1:1.0891925087)/0.935:0.1030551729,Arthrobotrys
entomopaga_KAF3922393.1:0.2921090641)/1:1.0058657323)/1:0.2188536040)/
0.994:0.2539557662,Yarrowia lipolytica
CLIB122_CAG81492.1:2.0484028528)/0.912:0.2451908987,(Candida albicans
SC5314_AOW26246.1:2.1467098101,Saccharomyces cerevisiae
S288C_NP_013614.1:3.1063180133)/1:0.8089907564)/0.995:0.2135312084,
(((((((((((Encephalitozoon intestinalis ATCC 50506_ADM12453.1:0.4654433956,
(Encephalitozoon hellem ATCC 50504_AFM99227.1:0.1534637040,Encephalitozoon
romaleae
SJ-2008_AFN83937.1:0.1133447964)/0.929:0.1542422656)/0.973:0.3171533381,Ordospor
a colligata OC4_KHN69724.1:1.0059983135)/1:1.1475776203,(((Nosema bombycis
CQ1_EOB12760.1:0.0576188201,Nosema bombycis
CQ1_EOB13352.1:0.0272028022)/1:0.8154013956,Nosema
granulosis_KAF9765078.1:0.5531489011)/1:0.4380150220,Nosema
ceranae_KKO76096.1:0.9850031520)/1:0.8445023212)/0.974:0.2959663292,(Vittaforma
corneae ATCC 50505_ELA42834.1:1.8129535662,(Enterocytozoon
hepatopenaei_OQS54639.1:2.6052276116,Hepatospora
eriocheir_ORD96838.1:1.9497121632)/1:0.7254822499)/0.999:0.4846803792)/
1:0.3080700374,(Tubulinosema
ratisbonensis_RVD93459.1:2.2752978416,Hamiltosporidium
magnivora_TBU09672.1:1.5184156547)/1:0.5859121133)/0.761:0.1180296867,((Vavraia

culicis subsp. floridensis_ELA47375.1:0.2036756766,Trachipleistophora
hominis_ELQ74779.1:0.4189837612)/1:0.8912473056,Pseudoloma
neurophilia_KRH93371.1:1.4845783750)/1:2.1427959692)/0.936:0.3368091527,Edhazard
ia aedis USNM 41457_EJW04985.1:1.8981082829)/0.989:0.3958327523,Thelohania
contejeani_KAF7683292.1:2.4781585006)/0.791:0.2412618346,Dictyocoela
muelleri_KAG0440389.1:1.6623342813)/0.999:1.1781026990,((Nematocida parisii
ERTm1_EIJ94843.1:0.0271184944,Nematocida sp.
ERTm5_OAG30645.1:0.0830007666)/1:1.2276137296,Nematocida sp. 1
ERTm6_KFG27185.1:0.5491214025)/1:5.2123323695)/1:1.1703093275,(Capsaspora
owczarzaki ATCC 30864_XP_004365012.1:8.9118539372,Sphaeroforma arctica
JP610_XP_014156159.1:1.1622474184)/0.79:0.3031842739)/0.997:0.3047538777,Capsasp
ora owczarzaki ATCC 30864_XP_004342694.1:1.2401390589)/0.936:0.2204155509,
(Rozella allomycis CSF55_EPZ34475.1:2.1091568588,(((((Conidiobolus coronatus
NRRL 28638_KXN71965.1:1.7454895608,Drosophila
melanogaster_NP_477468.1:0.8789108848)/1:0.7490704466,Amphimedon
queenslandica_XP_019864306.1:1.9882196149)/0.484:0.1079180718,((Homo
sapiens_NP_001974.1:0.1089585736,Mus
musculus_NP_031974.2:0.1973982327)/1:0.7881045316,Pomacea
canaliculata_PVD37947.1:1.0014427012)/1:0.3011031287)/0.911:0.1534291025,Caenorh
abditis elegans_NP_001369985.1:4.2276379616)/0.873:0.1471050172,(Monosiga
brevicollis MX1_XP_001742166.1:1.3908425936,Salpingoeca
rosetta_XP_004996535.1:0.8354504129)/1:0.9286170897)/0.658:0.1503479175)/
0:0.0420526715)/1:0.2176385421)/0.719:0.0483682059,(Olpidium
bornovanus_KAG5460917.1:1.6278262648,Thamnocephalis
sphaerospora_RKP08909.1:0.9753710520)/0.99:0.4186201964)/0.988:0.0569179544,
((((((Cryptococcus neoformans var. grubii H99_AFR96181.1:1.6113695936,
((((((Coprinopsis cinerea okayama7#130_EAU91778.2:0.4233311180,Amanita muscaria
Koide BX008_KIL66266.1:0.8231613188)/0.965:0.1356076226,Serpula lacrymans var.
lacrymans S7.9_EGO25466.1:0.4045333493)/0.8:0.0555853329,Postia placenta Mad-
698-R_EED78769.1:0.4719736069)/1:0.2918100238,Ramaria
rubella_KAF8590878.1:0.6147866071)/1:0.2484958784,Dacryopinax
primogenitus_EJU04486.1:0.8791113035)/0.992:0.2001253936,Rhizoctonia
solani_QRW21838.1:1.3822055256)/1:0.3141230657,Wallemia ichthyophaga EXF-
994_EOQ98802.1:1.5280449212)/0.868:0.0992464235)/0.478:0.0734641721,((Puccinia
graminis f. sp. tritici CRL 75-36-700-3_EFP86196.1:0.9443469779,Melampsora
larici-populina 98AG31_EGG00371.1:0.9186965368)/1:0.4586092763,Mixia osmundae
IAM 14324_KEI37007.1:1.7974857740)/0.707:0.1652558438)/0.998:0.1242102422,
((Malassezia globosa CBS 7966_EDP44281.1:1.0908573803,Tilletiaria anomala UBC
951_KDN43419.1:0.9145029180)/0.669:0.1601921659,Ustilago maydis
521_KIS65841.1:0.9389100742)/1:0.2286402948)/1:0.2019546015,(((Batrachochytrium
dendrobatidis JAM81_EGF78952.1:1.4195869280,(((Mitosporidium
daphniae_KGG50181.1:2.8266775745,Smittium
angustum_PWA03497.1:1.6271665400)/0:0.0000020749,Caulochytrium
protostelioides_RKP00462.1:1.7662002945)/0.997:0.2478316346,(Rhizoclosmatium
globosum_ORY46500.1:0.6007618060,Chytriomyces
confervae_TPX66324.1:0.7964860170)/1:0.5541263653)/0.783:0.1420574966)/
0.465:0.1046821682,Olpidium
bornovanus_KAG5456082.1:2.5142973600)/0.859:0.1804906608,(((((((Allomyces
macrogynus ATCC 38327_KNE58248.1:0.0000022457,Allomyces macrogynus ATCC
38327_KNE58251.1:0.1325974742)/0.998:0.0672201337,Allomyces macrogynus ATCC
38327_KNE58249.1:0.0000021548)/0.401:0.0234181039,Allomyces macrogynus ATCC
38327_KNE67375.1:0.3851352771)/1:0.8640130174,Catenaria anguillulae
PL171_ORZ41703.1:0.9114008126)/0.701:0.1230825689,Fonticula
alba_XP_009494375.1:4.7136455211)/0.989:0.4033711662,Gonapodya prolifera
JEL478_KXS18281.1:2.1116026419)/0.418:0.1066756685,Endogone sp. FLAS-
F59071_RUS14446.1:1.9370395819)/0.996:0.1548611921)/0.956:0.0878433525)/
0.949:0.0291774670,((((Entomophthora
muscae_KAF7715789.1:0.0346632135,Entomophthora
muscae_KAF7753013.1:0.0000022433)/1:2.0231492702,Piptocephalis
cylindrospora_RKP13292.1:1.5570725793)/0.543:0.1313588404,Dimargaris
cristalligena_RKP40058.1:0.9964855622)/1:0.1463864468,(((((Neocallimastix sp.
JGI-2020a_KAG4105768.1:0.0000020169,Neocallimastix
californiae_ORY34187.1:0.0758307248)/1:0.1658783922,Piromyces sp.
E2_OUM68238.1:0.1051647800)/0.981:0.0645230641,Anaeromyces

robustus_ORX72587.1:0.2722623199)/0.196:0.0082355061,Piromyces
finnis_ORX57712.1:0.2245964118)/1:1.2059991418,((Smittium
mucronatum_OLY80708.1:0.8118181419,(Smittium
simulii_PVU95762.1:0.9647309675,Smittium
megazygosporum_PVV00853.1:0.6619266485)/1:0.7144323060)/0.946:0.3103527865,
(Linderina pennispora_ORX68049.1:0.6639500125,Coemansia reversa NRRL
1564_PIA16760.1:0.9162163816)/1:0.9542075803)/0.964:0.2333918120)/
0.827:0.1003356533)/0.999:0.1873846629)/0.994:0.0542356768,(((Spizellomyces
punctatus DAOM BR117_KND01765.1:0.0216388546,Spizellomyces sp.
'palustris'_TPX72716.1:0.1473001275)/1:0.5446985957,Powellomyces
hirtus_TPX60038.1:0.5797838779)/1:0.3043659460,(Synchytrium
microbalum_TPX30058.1:0.4794810216,Synchytrium
endobioticum_TPX43350.1:0.6919820249)/1:0.4902860517)/1:0.2453334794)/
1:0.0684770696)/1:0.1477392921,((((((((((Dissophora
ornata_KAF8930819.1:0.3120142106,Gamsiella
multidivaricata_KAG0370656.1:0.3141765434)/1:0.1208341440,Dissophora
globulifera_KAG0312679.1:0.3619023933)/0.934:0.0341307722,(((Mortierella sp.
NVP85_KAF9347918.1:0.2428075438,(Lunasporangiospora
selenospora_KAF9577119.1:0.8240631407,Mortierella sp.
GBA43_KAG0245894.1:0.3912780701)/0.858:0.0939535816)/0.999:0.0694862173,Modicell
a reniformis_KAG0007136.1:0.2776407787)/0.946:0.0851317457,Lobosporangium
transversale_ORZ10355.1:0.3547709517)/0.919:0.0588358310)/1:0.0712108610,
(((Entomortierella lignicola_KAF8955912.1:0.0224865098,Haplosporangium sp. Z
27_KAF9201893.1:0.0123727133)/1:0.2592971768,(Mortierella sp.
AM989_KAF9116815.1:0.2368918129,(((Mortierella sp.
AD010_KAF9170713.1:0.0000022667,Mortierella sp.
AD011_KAF9402953.1:0.0000025442)/1:0.0501540072,Entomortierella
chlamydospora_KAF9995481.1:0.0332739432)/1:0.0951489200,Mortierella sp.
AD094_KAF9350013.1:0.0518790894)/1:0.0947533436)/1:0.0898591937)/0.918:0.0393748
774,(Entomortierella beljakovae_KAF9435701.1:0.3150393694,(Smittium
culicis_OMJ12129.1:0.6673822132,(Monosiga brevicollis
MX1_XP_001743923.1:0.8632818561,(Salpingoeca
rosetta_XP_004990750.1:0.6910677808,Sphaeroforma arctica
JP610_XP_014150642.1:1.1409350110)/0.492:0.6606950091)/1:4.3455878085)/
0.994:1.2243401355)/0.997:0.4130391059)/0.998:0.0788904695)/1:0.0471995901,
(Mortierella antarctica_KAF9986057.1:0.3760124578,Mortierella sp.
GBA30_KAG0203016.1:0.2648717837)/1:0.0980337429)/0.886:0.0409495444,
(Haplosporangium sp. Z 767_KAF9192481.1:0.1065631736,Mortierella
polycephala_KAG0263331.1:0.0758143608)/1:0.2756068531)/0.993:0.0401219892,Grygan
skiella cystojenkinii_KAG0047266.1:0.4396184000)/0.999:0.0498185033,
((((Haplosporangium bisporale_KAF8958216.1:0.0000024508,Podila verticillata NRRL
6337_KFH73481.1:0.0000027525)/0.967:0.0657889502,((Haplosporangium
bisporale_KAF8968659.1:0.1209262060,Podila
minutissima_KAG0352807.1:0.2349597691)/0:0.0173059835,Podila
humilis_KAG0345762.1:0.7379076166)/1:0.2008738442)/0.994:0.0860736984,(Podila
horticola_KAF9321934.1:0.0941141182,(Podila
clonocystis_KAG0033876.1:0.1188853002,Podila
epicladia_KAG0096489.1:0.1804648818)/0.938:0.0233946349)/1:0.0965999742)/
1:0.2425795903,Podila
epigama_KAF9428591.1:0.2898753516)/1:0.1635183143)/0.964:0.0501394222,
(((((Haplosporangium gracile_KAF8943825.1:0.0602855001,Linnemannia
schmuckeri_KAF9149764.1:0.0423638122)/1:0.0405502310,(((Mortierella sp.
GBA39_KAF9143519.1:0.0329453610,Linnemannia
hyalina_KAG9068307.1:0.0403804113)/0.978:0.0074407700,Mortierella
hygrophila_KAF9547315.1:0.0257836450)/1:0.0264143687,Linnemannia elongata AG-
77_OAQ27757.1:0.0630650723)/1:0.0397339067)/0.996:0.0437325771,Linnemannia
gamsii_KAG0291372.1:0.1738533833)/1:0.1549196968,((Mortierella sp.
14UC_KAF9122884.1:0.1165646248,Linnemannia
zychae_KAF9911577.1:0.0901901962)/1:0.0749721330,(Linnemannia
exigua_KAG0275022.1:0.0786196439,Mortierella sp.
AD032_KAG0365118.1:0.1914168764)/1:0.0754883831)/1:0.1202279499)/0.953:0.0372872
713,(Mortierella sp. AD031_KAF9091493.1:0.0031110695,Mortierella sp.
GBA35_KAF9105838.1:0.0000023554)/1:0.2460447221)/1:0.1391201841)/1:0.1680843133,
(Actinomortierella wolfii_KAG0239872.1:0.2206251794,Actinomortierella

ambigua_KAG0258936.1:0.2390133210)/1:0.4154138898)/1:0.5385674059,Basidiobolus meristosporus CBS 931.73_ORX91967.1:1.1426080155)/0.743:0.0645248720)/0.994:0.1410246988)/1:0.2090723174,(Umbelopsis isabellina_KAG2180194.1:0.3817478793,Umbelopsis vinacea_KAG2181517.1:0.2657933094)/1:0.3129312437)/1:0.2462392224,((((((Absidia glauca_SAM02218.1:0.3668225526,Absidia repens_ORZ15660.1:0.3200662159)/0.994:0.1200520387,Hesseltinella vesiculosa_ORX56374.1:0.7555994809)/1:0.3265580650,Phycomyces blakesleeanus NRRL 1555_OAD74263.1:1.0560182587)/0.969:0.1683818201,(Apophysomyces ossiformis_KAF7721264.1:0.2562350337,Apophysomyces sp. BC1015_KAG0162125.1:0.0964339705)/1:0.5131754410)/0.977:0.1374220337,Mucor circinatus_KAG2222375.1:0.8475003049)/0.9:0.0520239178,Syncephalastrum racemosum_ORY98681.1:0.8444182973)/0.858:0.0356716742)/1:0.3663931513,(Mucor saturninus_KAG2196033.1:0.5383754071,Thamnidium elegans_KAG2237642.1:0.5058557898)/1:0.2709058378)/0.999:0.1270196086,Choanephora cucurbitarum_OBZ89413.1:0.4764684616)/0.939:0.1119356432,(Rhizopus delemar RA 99-880_EIE83894.1:0.6752295610,(Rhizopus microsporus ATCC 52813_PHZ17624.1:0.0420306029,Rhizopus azygosporus_RCH82036.1:0.0000029458)/1:0.5177278584)/1:0.3761039756)/0.958:0.1110450836,Parasitella parasitica_CEP13840.1:0.2986529999)/0.555:0.0520948405,Mucor plumbeus_KAG2202448.1:0.1078160784)/1:0.1431967688,Mucor lusitanicus_KAF1806336.1:0.0972913884);

****

FAN1

****

(Mucor ambiguus_GAN01392.1:0.1328935197,(((((((((((((Schizosaccharomyces pombe_CAB46759.1:0.0000023016,Schizosaccharomyces pombe_NP_595395.1:0.0000029061)/1:1.3474982819,Schizosaccharomyces japonicus yFS275_EEB08822.1:1.1479851389)/0.942:0.3794800284,Gonapodya prolifera JEL478_KXS16034.1:3.2216063701)/0.994:0.5123749521,((((Blumeria graminis f. sp. triticale_CAD6504192.1:0.9735096395,Sclerotinia sclerotiorum 1980 UF-70_EDO00123.1:0.5682708773)/0.993:0.1411096795,((Neurospora crassa OR74A_EAA34805.3:0.7136334742,(Trichoderma reesei QM6a_EGR50769.1:0.5306465808,Verticillium dahliae VdLs.17_EGY21620.1:0.4500509648)/1:0.1202068970)/1:0.2220780158,(((Aspergillus nidulans FGSC A4_EAA57621.1:0.5585547945,Coccidioides immitis RS_EAS33670.3:0.6233874271)/1:0.2774813606,Bacidia gigantensis_KAG8527045.1:0.3661159064)/0.993:0.0959790594,(Zymoseptoria tritici IPO323_EGP87218.1:0.4843779456,Exophiala dermatitidis NIH/UT8656_EHY59639.1:0.7524428667)/1:0.0911456736)/1:0.1760592973)/0.583:0.0772436379)/1:0.4769024354,(Orbilia oligospora ATCC 24927_EGX51974.1:0.2955695434,Drechslerella brochopaga_KAF3905947.1:0.2018200683)/1:0.6571054918)/1:0.4478459132,Neolecta irregularis DAH-3_OLL22858.1:1.0128396936)/1:0.2905287279)/1:0.2242113929,((((Cryptococcus neoformans var. grubii H99_AFR98455.2:1.7096863116,(((((Coprinopsis cinerea okayama7#130_EAU84333.2:1.0691508533,Amanita muscaria Koide BX008_KIL56797.1:0.9164079022)/1:0.5846690031,(Serpula lacrymans var. lacrymans S7.9_EGO21271.1:0.2937126222,Serpula lacrymans var. lacrymans S7.9_EGO21273.1:0.0291749618)/1:0.8530140432)/1:0.3552852771,Ramaria rubella_KAF8582657.1:1.4651999469)/0.867:0.1078764781,Rhizoctonia solani_QRW16100.1:1.5108349999)/0.973:0.1623112925,Dacryopinax primogenitus_EJU00598.1:1.2936521990)/0.993:0.1838483315)/0.995:0.2149228975,Wallemia ichthyophaga EXF-994_EOR04921.1:2.3156941964)/1:0.2486555656,(Malassezia globosa CBS 7966_EDP42695.1:1.8848788603,(Tilletiaria anomala UBC 951_KDN45269.1:0.9642983574,Ustilago maydis 521_KIS68720.1:1.0135501388)/0.919:0.2026456375)/1:0.6762174636)/0.929:0.0823027339,((Melampsora larici-populina 98AG31_EGG09831.1:1.1166870059,Puccinia graminis f. sp. tritici CRL 75-36-700-3_EHS64085.1:1.4622587777)/1:0.4280642187,Mixia osmundae IAM 14324_KEI36679.1:1.1576370759)/1:0.3222423606)/1:0.2091738866)/1:0.1540158482,

((((((Postia placenta Mad-698-R_EED77405.1:1.2438667715,(((Paramicrosporidium
saccamoebae_PJF16464.1:2.0300256486,(Sphaeroforma arctica
JP610_XP_014158096.1:0.0064236269,Sphaeroforma arctica
JP610_XP_014158097.1:0.0002638174)/1:1.8823871430)/0.985:0.2744823339,((((Homo
sapiens_NP_055782.3:0.2645958743,Mus
musculus_NP_808561.2:0.2614687142)/1:1.2368078121,Amphimedon
queenslandica_XP_019863346.1:2.6756242978)/0.46:0.1565318848,(Caenorhabditis
elegans_NP_500997.1:0.0000028005,Caenorhabditis
elegans_CDK13373.1:0.0000026192)/1:2.5711731085)/1:0.3109191501,((Monosiga
brevicollis MX1_XP_001743887.1:1.9211754063,Salpingoeca
rosetta_XP_004997067.1:1.1480236669)/1:0.5497997444,Capsaspora owczarzaki ATCC
30864_XP_004365129.2:1.6943104232)/1:0.3891495038)/0.771:0.1062640280)/
1:0.2031568836,(Capsaspora owczarzaki ATCC
30864_XP_004364343.1:3.0925227693,Sphaeroforma arctica
JP610_XP_014158607.1:3.1073215636)/0.969:0.3173910300)/0.978:0.2110108976)/
0.295:0.0373492369,(((Spizellomyces punctatus DAOM
BR117_KND04064.1:0.0000021919,Spizellomyces punctatus DAOM
BR117_KND04065.1:0.0000023355)/1:0.0567645074,Spizellomyces sp.
'palustris'_TPX67861.1:0.0965481465)/1:0.7393151247,Powellomyces
hirtus_TPX54486.1:0.8936729825)/1:1.7063030824)/1:0.2096392905,
((((Batrachochytrium dendrobatidis JAM81_EGF78296.1:1.8958138835,
(Rhizoclosmatium globosum_ORY52408.1:1.1014540248,Chytriomyces
confervae_TPX46337.1:0.8676838263)/1:1.3845031362)/0.433:0.0983744708,(Olpidium
bornovanus_KAG5459748.1:1.6103306991,Blyttiomyces
helicus_RKO84673.1:0.5303224597)/0.84:0.2393755891)/0.875:0.2839105866,Gonapodya
prolifera JEL478_KXS16035.1:2.8708107858)/0.949:0.1942384885,((Allomyces
macrogynus ATCC 38327_KNE58938.1:0.7150566473,Allomyces macrogynus ATCC
38327_KNE71579.1:0.0000025568)/1:1.2625923831,Catenaria anguillulae
PL171_ORZ31832.1:1.8142430309)/1:0.9150869037)/0.999:0.2725668227)/
0.865:0.1501517813,(Conidiobolus coronatus NRRL 28638_KXN64990.1:1.0183395414,
((Conidiobolus coronatus NRRL 28638_KXN66745.1:1.0404218378,Conidiobolus
coronatus NRRL 28638_KXN66747.1:1.1970830694)/0.986:0.2078178855,Conidiobolus
coronatus NRRL
28638_KXN70148.1:0.8958028092)/1:0.5044614877)/1:1.5897220953)/0.964:0.088365387
4,(((((Smittium mucronatum_OLY78724.1:0.7668329864,Smittium
culicis_OMJ17368.1:0.6248702372)/1:0.5587770330,(Furculomyces
boomerangus_PVU94819.1:0.0065164995,Smittium
angustum_PWA01215.1:0.0034417122)/1:0.9865248745)/0.993:0.1871720351,((Smittium
simulii_PVU89062.1:0.0000023732,Smittium
simulii_PVU89065.1:0.0000020348)/1:0.8638421068,(Smittium
megazygosporum_PVU89527.1:0.0178473765,Smittium
megazygosporum_PVV01157.1:0.0000025444)/1:0.7628080503)/1:0.5095984235)/
0.947:0.2131674011,Zancudomyces
culisetae_OMH84222.1:1.7835810645)/1:0.7062609498,(Linderina
pennispora_ORX70821.1:1.1412088446,Coemansia reversa NRRL
1564_PIA13903.1:0.9769787817)/1:0.9239443140)/1:0.4709686710)/0.996:0.1117387075
,((((Entomophthora muscae_KAF7726308.1:0.0040274042,Entomophthora
muscae_KAF7726313.1:0.0264849378)/1:2.0696025107,Dimargaris
cristalligena_RKP34066.1:1.5242517015)/0.99:0.2626460026,(Thamnocephalis
sphaerospora_RKP10812.1:0.9978564070,Syncephalis
pseudoplumigaleata_RKP26820.1:1.4329516209)/1:0.7101660487)/0.988:0.1337318374,B
asidiobolus meristosporus CBS
931.73_ORY06734.1:1.3400818333)/1:0.2689742734)/1:0.1809115090)/1:0.1805962020,
((((((((Dissophora ornata_KAF8926201.1:0.2542991528,Gamsiella
multidivaricata_KAG0367820.1:0.2529979877)/0.998:0.0447897190,
((((Entomortierella lignicola_KAF8982803.1:0.0172189076,Haplosporangium sp. Z
27_KAF9206370.1:0.0028704466)/1:0.2488805934,(Mortierella sp.
AM989_KAF9111855.1:0.1773588539,((((Mortierella sp.
AD010_KAF9174736.1:0.0000027264,Mortierella sp.
AD011_KAF9400427.1:0.0000021743)/1:0.1542202738,Entomortierella
chlamydospora_KAG0005039.1:0.0263641886)/1:0.0857050104,Mortierella sp.
AD094_KAF9354788.1:0.0415779107)/0.36:0.0109455691,Mortierella sp.
AD094_KAF9354789.1:0.1371743737)/1:0.0935742365)/0.986:0.0505390565)/
1:0.0537072951,Entomortierella

beljakovae_KAF9437609.1:0.3548568922)/1:0.0614238846,Lobosporangium
transversale_ORZ27752.1:0.4855451999)/1:0.0450622868)/0.95:0.0312600948,
(((Mortierella sp. NVP85_KAF9366293.1:0.2547092060,Mortierella sp.
GBA43_KAG0240423.1:0.2184013334)/1:0.0762374069,Modicella
reniformis_KAF9959989.1:0.2329798446)/1:0.1545158196,Dissophora
globulifera_KAG0322036.1:0.4572826815)/1:0.0388993251)/1:0.1011169470,
(((Haplosporangium sp. Z 767_KAF9184399.1:0.0017361606,Haplosporangium sp. Z
11_KAF9189109.1:0.0000029655)/1:0.0877793904,Mortierella
polycephala_KAG0254905.1:0.0983430555)/1:0.2077128658,Mortierella sp.
GBA30_KAG0205744.1:0.3520545183)/0.995:0.0438556129)/1:0.0512815831,
((((Haplosporangium gracile_KAF8948933.1:0.0548382077,Linnemannia
schmuckeri_KAF9154113.1:0.0457055213)/1:0.0418018280,(((Mortierella sp.
GBA39_KAF9139715.1:0.0148245939,(Mortierella
hygrophila_KAF9542429.1:0.0235510652,Linnemannia
hyalina_KAG9068458.1:0.0256191589)/0.955:0.0045694252)/1:0.0335287890,Linnemanni
a elongata AG-77_OAQ31740.1:0.0460876904)/1:0.0223697630,Linnemannia
gamsii_KAG0296357.1:0.0905776625)/0.433:0.0062144502)/1:0.0557614971,
((Mortierella sp. 14UC_KAF9129516.1:0.0530756325,Linnemannia
zychae_KAF9901708.1:0.1061857654)/1:0.0361170197,Mortierella sp.
AD032_KAG0371657.1:0.0937218763)/1:0.0457414100)/1:0.0676799130,((Mortierella
sp. AD031_KAF9092218.1:0.0022620963,Mortierella sp.
NVP41_KAG0210009.1:0.0000027262)/1:0.0041339788,Mortierella sp.
GBA35_KAF9101400.1:0.0026887357)/1:0.1019346331)/1:0.1757817799)/1:0.0736831144,
Gryganskiella cystojenkinii_KAG0054639.1:0.3843571980)/0.986:0.0687744545,
((((Haplosporangium bisporale_KAF8934735.1:0.0000026988,Podila verticillata NRRL
6337_KFH73530.1:0.0000030097)/1:0.0808275122,((Podila
horticola_KAF9321982.1:0.0791977284,Podila
clonocystis_KAG0035376.1:0.0663367493)/0.985:0.0118932465,(Podila
epicladia_KAG0097390.1:0.0543524405,Podila
minutissima_KAG0347932.1:0.0313509605)/1:0.0214640827)/1:0.0396678058)/
1:0.1350653387,Podila humilis_KAG0339586.1:0.4374664877)/1:0.0748322452,Podila
epigama_KAF9431030.1:0.4266413671)/1:0.2329967898)/0.997:0.0997861144,Lunasporan
giospora selenospora_KAF9581062.1:0.3988068336)/1:0.4569349230,
(Actinomortierella wolfii_KAG0237113.1:0.4407592820,Actinomortierella
ambigua_KAG0266069.1:0.1566638974)/1:0.5066202718)/1:1.1595229854)/
1:0.1725423321,(Bifiguratus adelaidae_OZJ07000.1:1.0833610664,(Jimgerdemannia
flammicorona_RUO95662.1:1.0475963028,Endogone sp. FLAS-
F59071_RUS18970.1:0.4155845297)/1:0.3803004857)/1:0.2314750341)/1:0.2459198876,
(Umbelopsis isabellina_KAG2183177.1:0.5758128781,Umbelopsis
vinacea_KAG2187344.1:0.5944989789)/1:0.7262362553)/1:0.3890264410,
(((Apophysomyces ossiformis_KAF7727731.1:0.4428402640,((Apophysomyces sp.
BC1015_KAG0168669.1:0.0000020916,Apophysomyces sp.
BC1034_KAG0191351.1:0.0000028665)/0.126:0.0000023026,Apophysomyces sp.
BC1021_KAG0179196.1:0.0000021795)/1:0.6733320425)/1:0.4483076315,Mucor
circinatus_KAG2221561.1:0.9854207218)/0.443:0.1724155127,Syncephalastrum
racemosum_ORY96863.1:0.9452822431)/0.776:0.0472209486)/1:0.1598029823,((Absidia
glauca_SAM05527.1:0.2252585847,Absidia
repens_ORZ15780.1:0.4138174897)/1:0.4369241600,Hesseltinella
vesiculosa_ORX54373.1:0.8163281068)/1:0.3678048907)/1:0.5785989746,(Rhizopus
delemar RA 99-880_EIE82270.1:0.2318690219,((Rhizopus microsporus ATCC
52813_PHZ13745.1:0.0849848589,Rhizopus
azygosporus_RCH98203.1:0.0161717658)/0.946:0.0222505387,Rhizopus
azygosporus_RCH78920.1:0.0458809663)/1:0.3114597406)/1:0.5244524589)/
0.965:0.1371072767,(Mucor saturninus_KAG2210228.1:0.4434438610,Thamnidium
elegans_KAG2229872.1:0.4125047655)/1:0.2806503241)/1:0.1480063805,Choanephora
cucurbitarum_OBZ84791.1:0.6099090785)/1:0.3246826538,(Parasitella
parasitica_CEP13314.1:0.4548463458,Mucor
plumbeus_KAG2204394.1:0.2653916748)/0.947:0.0682097561)/1:0.3081993934,Mucor
lusitanicus_KAF1799975.1:0.0874732951);

****

FANCA

****

****
(Mucor ambiguus_GAN01546.1:0.0793886181,(((Parasitella parasitica_CEP14355.1:0.3404022299,Mucor plumbeus_KAG2192548.1:0.1595223048)/0.822:0.0513653355,((((((Absidia glauca_SAM05007.1:0.3174851723,Absidia repens_ORZ21635.1:0.5132714146)/1:0.5909203094,Hesseltinella vesiculosa_ORX48264.1:1.3411306760)/1:0.4649956118,((Apophysomyces ossiformis_KAF7728419.1:0.5798546568,(Apophysomyces sp. BC1015_KAG0165206.1:0.0000026619,Apophysomyces sp. BC1034_KAG0190805.1:0.0000026916)/1:0.3640775521)/1:0.7526867615,Phycomyces blakesleeanus NRRL 1555_OAD68996.1:0.9018486833)/0.869:0.1649897201)/1:0.2041047514,Mucor circinatus_KAG2224356.1:1.0181495038)/0.765:0.0664587346,(((Basidiobolus meristosporus CBS 931.73_ORY08175.1:1.4376485341,(((Homo sapiens_NP_000126.2:0.3221502378,Mus musculus_NP_058621.2:0.1499489377)/1:1.0379110301,Pomacea canaliculata_PVD21324.1:1.4248809270)/0.996:0.2775860260,Amphimedon queenslandica_XP_019856935.1:1.2845344354)/1:0.7248390358)/0.999:0.3600267848,Endogone sp. FLAS-F59071_RUS21520.1:1.1591302572)/1:0.3810223555,Syncephalastrum racemosum_ORY96444.1:1.2687057640)/0.226:0.0510045550)/1:0.7165340167,(Rhizopus delemar RA 99-880_EIE87991.1:0.6582588503,(Rhizopus microsporus ATCC 52813_PHZ08052.1:0.0332977435,(Rhizopus azygosporus_RCH98460.1:0.0153631454,Rhizopus azygosporus_RCI00193.1:0.0466865320)/0.945:0.0251892682)/0.937:0.2593653092)/1:0.4841458563)/1:0.2247843387,(Mucor saturninus_KAG2200303.1:0.2416383804,Thamnidium elegans_KAG2237817.1:0.3102746003)/1:0.2659820332)/1:0.4819119072)/1:0.1913105906,Mucor lusitanicus_KAF1798129.1:0.1735968140)/0.481:0.0185914513,Mucor lusitanicus_KAF1798128.1:0.1259336209);

****
FANCD2
****
(Mucor ambiguus_GAN01786.1:0.0890239455,((((((((((((((Rhizophagus clarus_GES92113.1:0.0000027496,Rhizophagus clarus_GES92114.1:0.0000024185)/1:0.0712447761,(Rhizophagus clarus_GES92115.1:0.0794713778,(Rhizophagus irregularis DAOM 181602=DAOM 197198_POG62244.1:0.0043439364,Rhizophagus diaphanus_RGB39818.1:0.0094452306)/1:0.0398352088)/0.632:0.0165178532)/1:0.0590595910,Glomus cerebriforme_RIA85935.1:0.0860000701)/1:0.4182326423,((Gigaspora margarita_KAF0459630.1:0.0225723944,Gigaspora rosea_RIB11525.1:0.0084430900)/1:0.3582396312,Diversispora epigaea_RHZ86697.1:0.4124306222)/1:0.2817422044)/1:0.2156047666,Geosiphon pyriformis_KAG9294638.1:0.8209004029)/1:0.4776678637,((((((Batrachochytrium dendrobatidis JAM81_EGF82370.1:1.8545585495,((Rhizoclosmatium globosum_ORY40978.1:0.7596846458,Chytriomyces confervae_TPX75892.1:0.7391581772)/1:1.0599533084,Blyttiomyces helicus_RKO85638.1:1.4584890578)/0.931:0.1011272209)/0.444:0.1135172597,(Synchytrium microbalum_TPX35761.1:0.6407089146,Synchytrium endobioticum_TPX46664.1:0.6167288735)/1:1.3513952470)/0.935:0.0822541232,Blyttiomyces helicus_RKO93488.1:1.2094424510)/0.992:0.0933330072,(Thamnocephalis sphaerospora_RKP05816.1:1.0898506698,Syncephalis pseudoplumigaleata_RKP22732.1:0.9620227088)/1:1.1132188492)/0.997:0.0701110111,((((Rozella allomycis CSF55_EPZ33440.1:3.8668994103,((((Allomyces macrogynus ATCC 38327_KNE62556.1:0.1610794142,Allomyces macrogynus ATCC 38327_KNE65757.1:0.0401660510)/0.955:0.1278774816,Allomyces macrogynus ATCC 38327_KNE62557.1:0.3264683746)/1:2.8883503251,Dictyostelium discoideum_XP_647652.1:2.6705464709)/0.997:0.2142155743,((((Homo sapiens_NP_149075.2:0.1644870927,Mus musculus_NP_001028416.2:0.2777267307)/1:0.9623988681,Pomacea canaliculata_PVD24835.1:1.4389903983)/1:0.2813253451,(Drosophila

melanogaster_NP_996246.1:3.2256297777,Amphimedon
queenslandica_XP_019851844.1:1.8758269479)/0.978:0.1596711428)/1:0.1769631996,
((Caenorhabditis elegans_NP_001255848.1:4.7973075747,(Monosiga brevicollis
MX1_XP_001744543.1:2.2254283250,Salpingoeca
rosetta_XP_004991565.1:1.5527323923)/1:0.3724169288)/0.976:0.1727293355,Capsaspo
ra owczarzaki ATCC
30864_XP_004349087.2:1.7299917731)/0.69:0.1290909043)/1:0.1773868636)/
0.966:0.1149172138)/0.126:0.0000026820,(Thamnocephalis
sphaerospora_RKP05815.1:2.3347379481,Dimargaris
cristalligena_RKP40479.1:1.2791935293)/0.754:0.1832680809)/0.994:0.1075773799,
(Gonapodya prolifera JEL478_KXS17734.1:2.1458340544,(((((((Smittium
mucronatum_OLY84117.1:0.3948589978,Smittium
culicis_OMJ07124.1:0.2212554621)/1:0.7035256088,Furculomyces
boomerangus_PVU93253.1:0.8891682617)/0.95:0.1175304855,(Smittium
simulii_PVU97872.1:0.8279861728,Smittium
megazygosporum_PVV01099.1:0.9121850931)/1:0.4284853322)/1:0.2828708857,(Smittium
simulii_PVU97871.1:0.6870996156,Smittium
megazygosporum_PVV00889.1:1.0661583681)/1:0.2719403526)/0.863:0.1821732208,Furcu
lomyces boomerangus_PVU93254.1:0.7556038821)/0.999:0.3131054303,Zancudomyces
culisetae_OMH82503.1:2.2069191338)/1:0.7319455590,(Linderina
pennispora_ORX74404.1:0.7533223595,Coemansia reversa NRRL
1564_PIA18579.1:0.9997206919)/1:0.9813500679)/1:0.3653352450,
((Paramicrosporidium saccamoebae_PJF16622.1:3.4147440877,Caulochytrium
protostelioides_RKP00536.1:2.6388831245)/0.733:0.1898667702,Piptocephalis
cylindrospora_RKP15418.1:1.9374547097)/0.936:0.1705286227)/0.852:0.1087514735)/
1:0.1518377958)/0.996:0.0770366144,((Entomophthora
muscae_KAF7754323.1:0.0000026143,Entomophthora
muscae_KAF7754991.1:0.0199178595)/1:1.6122130149,Conidiobolus coronatus NRRL
28638_KXN68624.1:2.0159820794)/1:0.7461496733)/0.992:0.1095792684)/
1:0.1826190486,(((((Neocallimastix sp. JGI-
2020a_KAG4107475.1:0.0006623496,Neocallimastix
californiae_ORY20298.1:0.0000021469)/1:0.2432543490,((((Piromyces
finnis_ORX59191.1:0.1231313602,Piromyces sp.
E2_OUM66618.1:0.3273615768)/0.279:0.0102071217,Piromyces sp.
E2_OUM66617.1:0.0706933562)/0.548:0.0233528447,Piromyces sp.
E2_OUM66616.1:0.0191157967)/0.296:0.0127733508,Piromyces sp.
E2_OUM66615.1:0.0332061899)/1:0.0953222122)/0.889:0.0359785997,Anaeromyces
robustus_ORX85340.1:0.1680869070)/1:1.6071483495,Olpidium
bornovanus_KAG5458847.1:1.2679377619)/0.883:0.1523624959,Basidiobolus
meristosporus CBS
931.73_ORX90317.1:1.0117036803)/0.993:0.1568764289)/0.968:0.1122285852,
(((((((Dissophora ornata_KAF8927424.1:0.1717673951,Dissophora
globulifera_KAG0324848.1:0.3341145224)/1:0.0288614245,(((Mortierella sp.
NVP85_KAF9361554.1:0.1462655386,Mortierella sp.
GBA43_KAG0246993.1:0.2114680545)/1:0.0374047328,Modicella
reniformis_KAF9963205.1:0.1743397912)/1:0.1960637358,Gamsiella
multidivaricata_KAG0361306.1:0.2832696688)/0.638:0.0285825538)/1:0.0224021284,
(((((Entomortierella lignicola_KAF8982937.1:0.0132860814,Haplosporangium sp. Z
27_KAF9198259.1:0.0060047582)/1:0.1072814101,(((Mortierella sp.
AD010_KAF9174856.1:0.0000026004,Mortierella sp.
AD011_KAF9401655.1:0.0000029773)/1:0.0263048108,Entomortierella
chlamydospora_KAF9997114.1:0.0174097679)/1:0.0534422278,Mortierella sp.
AD094_KAF9359265.1:0.0258082768)/1:0.0657508596)/1:0.0311317474,Mortierella sp.
AM989_KAF9105210.1:0.1411300548)/1:0.0490922567,Entomortierella
beljakovae_KAF9438579.1:0.2354193346)/1:0.1048977604,Lobosporangium
transversale_ORZ28109.1:0.2816096895)/1:0.0443778186)/1:0.0548022536,
((((((Haplosporangium gracile_KAF8943507.1:0.0293924657,Linnemannia
schmuckeri_KAF9150212.1:0.0199001858)/1:0.0342778989,(((Mortierella sp.
GBA39_KAF9134145.1:0.0187862064,(Mortierella
hygrophila_KAF9548785.1:0.0181755174,Linnemannia
hyalina_KAG9064770.1:0.0126759887)/1:0.0034970277)/1:0.0090487586,Linnemannia
elongata AG-77_OAQ31907.1:0.0412839883)/1:0.0197818870,Linnemannia elongata AG-
77_OAQ31908.1:0.0479841196)/1:0.0257749444)/1:0.0171984892,Linnemannia
gamsii_KAG0288630.1:0.0849378247)/1:0.0459513771,((Mortierella sp.

14UC_KAF9132558.1:0.0454753762,Linnemannia
zychae_KAF9911840.1:0.0883588073)/1:0.0266260678,(Linnemannia
exigua_KAG0275935.1:0.0382213617,Mortierella sp.
AD032_KAG0372089.1:0.0622064511)/1:0.0365420236)/1:0.0507956595)/1:0.0830389743,
((Mortierella sp. AD031_KAF9082104.1:0.0000021232,Mortierella sp.
NVP41_KAG0199249.1:0.0015047472)/0.147:0.0000092516,Mortierella sp.
GBA35_KAF9089043.1:0.0045097250)/1:0.0954159839)/1:0.2018347578,
((Haplosporangium sp. Z 767_KAF9180986.1:0.0000029424,Haplosporangium sp. Z
11_KAF9181157.1:0.0000020450)/1:0.0460316834,Mortierella
polycephala_KAG0251113.1:0.0662419393)/1:0.2946190370)/0.911:0.0207275466,
(Mortierella antarctica_KAF9981163.1:0.1553893025,Mortierella sp.
GBA30_KAG0207657.1:0.1677608142)/1:0.1508914955)/1:0.0391988649)/0.999:0.0397318
011,Lunasporangiospora
selenospora_KAF9581589.1:0.5903870093)/0.995:0.0448883979,Gryganskiella
cystojenkinii_KAG0054908.1:0.4238962006)/1:0.0618748927,(((((Haplosporangium
bisporale_KAF8985277.1:0.0004287242,Podila verticillata NRRL
6337_KFH73454.1:0.0080159669)/1:0.0737243751,((Podila
horticola_KAF9314590.1:0.0645093778,(Podila epicladia_KAG0082666.1:0.0486156469,
(Podila epicladia_KAG0088820.1:0.0636692102,Podila
minutissima_KAG0356563.1:0.0184957797)/0.145:0.0004008411)/1:0.0170612166)/
0.264:0.0090709291,Podila
clonocystis_KAG0025316.1:0.0494561555)/1:0.0439187475)/1:0.0960127773,Podila
epigama_KAF9398004.1:0.3244763763)/0.39:0.0152382222,Podila
epigama_KAF9409653.1:0.3046813535)/0.946:0.0576269331,Podila
humilis_KAG0328620.1:0.4912797025)/1:0.1729988160)/1:0.1725223646,
(Actinomortierella wolfii_KAG0240616.1:0.2174203744,Actinomortierella
ambigua_KAG0270710.1:0.1890482800)/1:0.4973609784)/1:0.9549805427)/
1:0.2002916924)/1:0.1128370206,((((Jimgerdemannia
flammicorona_RUP46562.1:0.0671531270,Endogone sp. FLAS-
F59071_RUS21067.1:0.8750845483)/0.98:0.1462524642,Endogone sp. FLAS-
F59071_RUS21065.1:0.4616893175)/0.704:0.0752646206,Endogone sp. FLAS-
F59071_RUS21066.1:0.7764475495)/0.389:0.0526103565,Endogone sp. FLAS-
F59071_RUS21064.1:0.4634922258)/1:0.3678949506)/0.616:0.0592454724,Bifiguratus
adelaidae_OZJ06305.1:2.0552452165)/1:0.3164318838,(Umbelopsis
isabellina_KAG2177140.1:0.8753235165,(Umbelopsis
isabellina_KAG2177141.1:0.5563274569,Umbelopsis
vinacea_KAG2182703.1:0.1873233997)/0.3:0.0444942889)/1:0.9325085569)/
1:0.4466030782,(Mucor circinatus_KAG2226033.1:0.5932478804,Syncephalastrum
racemosum_ORY92990.1:0.8370089159)/0.934:0.1126393677)/0.992:0.0545498002,
((((Absidia glauca_SAM08641.1:0.2374929921,Absidia
repens_ORZ16363.1:0.3187801218)/1:0.1907642910,Hesseltinella
vesiculosa_ORX54485.1:1.4128011814)/0.231:0.0166747801,Hesseltinella
vesiculosa_ORX54484.1:0.7669966842)/0.624:0.0673643284,Hesseltinella
vesiculosa_ORX54486.1:1.0337954092)/1:0.3171356232)/0.993:0.0524877114,
((Apophysomyces ossiformis_KAF7722276.1:0.2620144933,(Apophysomyces sp.
BC1015_KAG0165038.1:0.2611426364,(Apophysomyces sp.
BC1015_KAG0165039.1:0.0000029600,Apophysomyces sp.
BC1021_KAG0173219.1:0.0174333775)/0.999:0.1768000485)/0:0.0000027323)/
1:0.3534298729,Phycomyces blakesleeanus NRRL
1555_OAD66923.1:0.7398751930)/1:0.1283072564)/1:0.2395266881,(Rhizopus delemar
RA 99-880_EIE92163.1:0.2154203546,(Rhizopus delemar RA 99-
880_EIE92164.1:0.3147075920,((Rhizopus microsporus ATCC
52813_PHZ09814.1:0.0259547106,Rhizopus
azygosporus_RCH84605.1:0.0386258057)/0.966:0.0176953629,Rhizopus
azygosporus_RCH80279.1:0.0974242864)/1:0.1627542351)/0.219:0.0113380960)/
1:0.3425475282)/1:0.1042559706,Choanephora
cucurbitarum_OBZ85627.1:0.4738197150)/0.982:0.0569068378,(Mucor
saturninus_KAG2201193.1:0.2559309944,Thamnidium
elegans_KAG2235852.1:0.1874222793)/1:0.1650674236)/1:0.3714334750,(Parasitella
parasitica_CEP15345.1:0.2763017772,Mucor
plumbeus_KAG2203881.1:0.1682058763)/1:0.0589137680)/1:0.1905241254,Mucor
lusitanicus_KAF1805430.1:0.0976867072);

****

****
FANCE
****
****
(Mucor ambiguus_GAN09205.1:0.1431962762,(((((((((((((((Rhizophagus
clarus_GES78490.1:0.0000028319,Rhizophagus
clarus_GES78491.1:0.0312066322)/1:0.0792106466,(Rhizophagus irregularis DAOM
181602=DAOM 197198_POG66492.1:0.0287561673,Rhizophagus
diaphanus_RGB42831.1:0.0104554779)/1:0.0591113643)/1:0.0611532865,Glomus
cerebriforme_RIA97945.1:0.0937900266)/1:0.4759063382,(Gigaspora
margarita_KAF0532448.1:0.0388148840,Gigaspora
rosea_RIB24671.1:0.0031643567)/1:0.4856220052)/0.887:0.1128055177,Diversispora
epigaea_RHZ81192.1:0.6424330389)/1:0.2799810176,Geosiphon
pyriformis_KAG9303111.1:1.0271802795)/0.998:0.2817571371,Jimgerdemannia
flammicorona_RUP47654.1:0.5008637715)/0.943:0.2861143044,Linnemannia
gamsii_KAG0276651.1:2.2355667672)/0.477:0.1741567733,(Basidiobolus meristosporus
CBS 931.73_ORX96829.1:1.2914662411,(((Homo sapiens_NP_068741.1:0.2320099057,Mus
musculus_NP_001157291.1:0.3242147862)/1:1.2822036224,Pomacea
canaliculata_XP_025086429.1:1.6138583001)/0.914:0.2413870464,Amphimedon
queenslandica_XP_019855713.1:2.0686813570)/1:0.5755606510)/0.885:0.1782067075)/
0.969:0.2139304480,(Umbelopsis
isabellina_KAG2180096.1:2.0456049224,Dictyostelium discoideum
AX4_XP_641588.1:2.3844692644)/0.962:0.3997352975)/1:0.5295719248,Mucor
circinatus_KAG2226146.1:1.4874554196)/0.999:0.2904524205,Phycomyces
blakesleeanus NRRL 1555_OAD72538.1:0.6896172499)/0.777:0.0801277200,((Absidia
glauca_SAM02112.1:0.4981869643,Absidia
repens_ORZ23345.1:0.4635333708)/0.978:0.1423964045,Hesseltinella
vesiculosa_ORX61481.1:1.1464762601)/1:0.1834191252)/1:0.1637819105,
(((Apophysomyces sp. BC1015_KAG0168758.1:0.0000020431,Apophysomyces sp.
BC1021_KAG0178417.1:0.0000023142)/0.135:0.0000021216,Apophysomyces sp.
BC1034_KAG0188825.1:0.0031655030)/1:0.5876820170,Syncephalastrum
racemosum_ORY98105.1:1.0017608300)/0.13:0.0006131500)/1:0.1997132917,(Rhizopus
delemar RA 99-880_EIE83065.1:0.2825369822,(Rhizopus microsporus ATCC
52813_PHZ17932.1:0.0219559343,Rhizopus
azygosporus_RCH90546.1:0.0690728014)/1:0.3165670275)/1:0.4185095435)/
0.701:0.0811990732,(Mucor saturninus_KAG2198668.1:0.3450387258,Thamnidium
elegans_KAG2229666.1:0.2809516995)/1:0.1115057491)/0.998:0.1180069433,Choanephor
a cucurbitarum_OBZ84676.1:0.7060568506)/1:0.2719089709,(Parasitella
parasitica_CEP17470.1:0.2174133808,Mucor
plumbeus_KAG2197907.1:0.1121614648)/0.414:0.0435817583)/1:0.1933093297,Mucor
lusitanicus_KAF1796241.1:0.1549431823);

****
****
FANCI
****
****
(Mucor ambiguus_GAN03661.1:0.0727286978,(((((((((((((Rhizophagus
clarus_GES91536.1:0.0785722723,(Rhizophagus irregularis DAOM 181602=DAOM
197198_POG67280.1:0.0096724849,Rhizophagus
diaphanus_RGB29081.1:0.0351586637)/1:0.0594296630)/1:0.0904678167,Glomus
cerebriforme_RIA87302.1:0.1327201797)/1:0.4267293671,((Gigaspora
margarita_KAF0524594.1:0.0175507419,Gigaspora
rosea_RIB09784.1:0.0339590868)/1:0.4588007270,((((Diversispora
epigaea_RHZ75476.1:0.0038134025,Diversispora
epigaea_RHZ85811.1:0.3361051701)/0.788:0.0329782857,Diversispora
epigaea_RHZ78479.1:0.1819016099)/0.996:0.1089756259,Diversispora
epigaea_RHZ76634.1:0.0587235886)/1:1.6298379412,(Diversispora
epigaea_RHZ81659.1:0.1004353455,Diversispora
epigaea_RHZ81817.1:2.4906526748)/0.964:0.1857835545)/1:0.2366476026)/
1:0.2088934211)/1:0.2834859440,Geosiphon
pyriformis_KAG9306620.1:0.7915217914)/1:0.5403307178,(((((((Batrachochytrium
dendrobatidis JAM81_EGF79704.1:2.9760917730,Rozella allomycis
CSF55_EPZ32320.1:2.9250866824)/0.988:0.4321499684,Salpingoeca

rosetta_XP_004993096.1:2.3320757407)/0.969:0.2008190875,Dictyostelium discoideum_tr|Q54BR4|Q54BR4_DICDI:3.1433364804)/0.635:0.0809286914,((((Homo sapiens_sp|Q9NVI1|FANCI_HUMAN:0.1503322769,Mus musculus_sp|Q8K368|FANCI_MOUSE:0.1786418382)/1:0.8552062249,Pomacea canaliculata_PVD30204.1:0.9932108535)/1:0.2535660005,Amphimedon queenslandica_XP_019851889.1:1.7856324099)/1:0.2688168195,(Drosophila melanogaster_AAF59016.2:3.1325167359,(Capsaspora owczarzaki ATCC 30864_XP_004346672.2:0.0000031924,Capsaspora owczarzaki ATCC 30864_XP_011270486.1:0.0008893470)/1:2.1697610571)/0.829:0.2568719270)/0.993:0.1553078381)/1:0.2230929896,((((Entomophthora muscae_KAF7752257.1:2.0886848311,Conidiobolus coronatus NRRL 28638_KXN70248.1:2.8031693894)/0.989:0.4740431151,Blyttiomyces helicus_RKO88610.1:1.0193058871)/0.435:0.0955640883,Dimargaris cristalligena_RKP34223.1:2.1552200668)/0.92:0.1352478491,(((Gonapodya prolifera JEL478_KXS10830.1:2.2349442931,Caenorhabditis elegans_tr|Q7Z151|Q7Z151_CAEEL:4.0945118001)/0.854:0.2927618606,(Thamnocephalis sphaerospora_RKP11010.1:2.5656232155,Piptocephalis cylindrospora_RKP13439.1:2.7981715430)/0.99:0.3463126920)/0.989:0.1832053280,((((((Smittium mucronatum_OLY85066.1:0.6284450387,Smittium culicis_OMJ17225.1:0.5327581047)/1:0.7584624619,((Furculomyces boomerangus_PVU97410.1:0.0012741766,Smittium angustum_PVZ98243.1:0.0000024739)/0.985:0.0012735287,Smittium angustum_PVZ96972.1:0.0000023268)/1:1.0099620328)/1:0.2709909018,(Smittium simulii_PVU89686.1:1.0416772320,((Smittium simulii_PVU89687.1:0.0099413839,Smittium simulii_PVU89715.1:0.0000022669)/1:1.0979497203,Smittium megazygosporum_PVU90263.1:1.1924936844)/0.222:0.0541541725)/0.981:0.4335515173)/1:1.0037981990,Zancudomyces culisetae_OMH81125.1:2.3695282846)/1:0.6476919123,(Linderina pennispora_ORX72413.1:1.0743421438,Coemansia reversa NRRL 1564_PIA18244.1:1.7225620842)/1:1.1547531305)/0.757:0.1323629694,Paramicrosporidium saccamoebae_PJF17248.1:3.7243132461)/1:0.3653882930)/0.975:0.1313049461)/1:0.1276953658)/0.986:0.1099774335,((Batrachochytrium dendrobatidis JAM81_EGF79705.1:2.5802946154,Blyttiomyces helicus_RKO82875.1:1.4121833709)/0.287:0.0366283105,(((((Neocallimastix sp. JGI-2020a_KAG4101863.1:0.0000026401,Neocallimastix californiae_ORY35124.1:0.0146398288)/0.999:0.0200937076,Neocallimastix californiae_ORY35123.1:0.0000026771)/1:0.2703172637,((Piromyces finnis_ORX34875.1:0.0933304261,Piromyces finnis_ORX55468.1:0.0000029367)/1:0.1426986157,Piromyces sp. E2_OUM69556.1:0.0669295974)/1:0.0910843403)/1:0.1001840305,Anaeromyces robustus_ORX86912.1:0.0987306167)/1:1.7108261498,(((((Allomyces macrogynus ATCC 38327_KNE55440.1:0.0000024582,Allomyces macrogynus ATCC 38327_KNE60098.1:0.2058840126)/0.504:0.0353615781,Allomyces macrogynus ATCC 38327_KNE60097.1:0.1904218215)/1:2.8454148947,Rhizoclosmatium globosum_ORY51858.1:2.3172751884)/0.938:0.2397516281,(Synchytrium microbalum_TPX37174.1:0.6539452154,Synchytrium endobioticum_TPX48165.1:0.6181216144)/1:2.2186439328)/0.987:0.2073629094,Blyttiomyces helicus_RKO89266.1:1.4094519528)/0.752:0.0372427464)/0.923:0.1016520339)/0.804:0.0809985428)/1:0.3102365094,(((((((Haplosporangium bisporale_KAF8932703.1:0.0000024501,Podila verticillata NRRL 6337_KFH71813.1:0.0118100908)/1:0.0895536726,(Podila horticola_KAF9309754.1:0.0564320362,(Podila clonocystis_KAG0023662.1:0.0701102290,((Podila epicladia_KAG0095160.1:0.0504286246,Podila minutissima_KAG0345124.1:0.0434730631)/0.858:0.0131947251,Podila minutissima_KAG0363141.1:0.0288183984)/1:0.0062987228)/1:0.0141933154)/1:0.0250307192)/1:0.0835914711,Podila humilis_KAG0346576.1:0.3908647460)/1:0.0878400610,Podila epigama_KAF9422171.1:0.3220998603)/1:0.1688401755,((((((((Dissophora ornata_KAF8935766.1:0.1469409731,Gamsiella multidivaricata_KAG0365943.1:0.2503246650)/0.949:0.0201727762,((((((Entomortierella lignicola_KAF8963051.1:0.0097986254,Haplosporangium sp. Z 27_KAF9204889.1:0.0000025498)/0.81:0.0041286556,Entomortierella lignicola_KAF8977816.1:0.0083659025)/1:0.1430620029,((((Mortierella sp.

AD010_KAF9170258.1:0.0000026849,Mortierella sp.
AD011_KAF9387950.1:0.0044664213)/0:0.0000027586,Zancudomyces
culisetae_OMH81126.1:3.7537253201)/0.213:0.0229754491,((Mortierella sp.
AD010_KAF9170651.1:0.0280517550,Mortierella sp.
AD011_KAF9397745.1:0.0000031804)/1:0.0993325139,Entomortierella
chlamydospora_KAG0001818.1:0.0189540176)/0.195:0.0019699876)/1:0.0588179734,Mort
ierella sp.
AD094_KAF9354159.1:0.0434000374)/1:0.0618629781)/1:0.0246191191,Mortierella sp.
AM989_KAF9108452.1:0.1261081485)/1:0.0382005636,Entomortierella
beljakovae_KAF9433039.1:0.2735190023)/1:0.0818301603,Lobosporangium
transversale_ORZ26962.1:0.3552144216)/1:0.0529200522,((Mortierella sp.
NVP85_KAF9358231.1:0.1449464628,Mortierella sp.
GBA43_KAG0242738.1:0.1878338806)/1:0.0531903234,Modicella
reniformis_KAG0002734.1:0.1332145609)/1:0.1241677729)/1:0.0251201472)/
0.201:0.0031564408,(Dissophora ornata_KAF8936189.1:0.1436084517,Dissophora
globulifera_KAG0311005.1:0.2898443639)/0.979:0.0618221596)/0.818:0.0207032927,Di
ssophora globulifera_KAG0316095.1:0.2515754542)/1:0.0401393521,
(((Haplosporangium sp. Z 767_KAF9186914.1:0.0006937251,Haplosporangium sp. Z
11_KAF9188462.1:0.0000026150)/1:0.0364067037,Mortierella
polycephala_KAG0261101.1:0.0781949816)/1:0.2557320248,(Mortierella
antarctica_KAF9987587.1:0.1487656096,Mortierella sp.
GBA30_KAG0200521.1:0.1868681974)/1:0.1202325074)/1:0.0450442885)/0.964:0.0355329
208,((((((Haplosporangium gracile_KAF8949078.1:0.0316742587,Linnemannia
schmuckeri_KAF9154413.1:0.0220274588)/0.999:0.0340907585,Haplosporangium
gracile_KAF8949079.1:0.0441891511)/0.997:0.0216952640,((Mortierella sp.
GBA39_KAF9144745.1:0.0383318042,(Mortierella
hygrophila_KAF9546780.1:0.0104956911,Linnemannia
hyalina_KAG9070815.1:0.0158958911)/1:0.0031217433)/1:0.0191734005,Linnemannia
elongata
AG-77_OAQ36263.1:0.0324268378)/1:0.0425725666)/1:0.0221163156,Linnemannia
gamsii_KAG0298035.1:0.0940114107)/1:0.0279963699,((Mortierella sp.
14UC_KAF9130739.1:0.0586457180,Linnemannia
zychae_KAF9913165.1:0.0925688479)/1:0.0192635723,(Linnemannia
exigua_KAG0281573.1:0.0719242291,Mortierella sp.
AD032_KAG0380276.1:0.0392499780)/1:0.0355456829)/1:0.0411510908)/1:0.0736199175,
((Mortierella sp. AD031_KAF9095148.1:0.0006703885,Mortierella sp.
NVP41_KAG0214023.1:0.0020132461)/0.988:0.0018400613,Mortierella sp.
GBA35_KAF9096291.1:0.0055560551)/1:0.0940728762)/1:0.2433688227)/1:0.0511754714,
Gryganskiella
cystojenkinii_KAG0051669.1:0.4452037925)/0.97:0.0426644627,Lunasporangiospora
selenospora_KAF9581425.1:0.5196004457)/1:0.1171829169)/1:0.1668845019,
((Actinomortierella wolfii_KAG0228412.1:0.1108214135,Actinomortierella
ambigua_KAG0267401.1:0.2513072931)/0.997:0.0751529017,Actinomortierella
ambigua_KAG0267402.1:0.2427902925)/1:0.5562015670)/1:0.9937471379,Bifiguratus
adelaidae_OZJ03311.1:1.9424651804)/0.923:0.1373113045)/0.993:0.0980308373,Bifigu
ratus adelaidae_OZJ04599.1:1.7307249680)/0.843:0.0589830099)/0.996:0.0970487184,
(Jimgerdemannia flammicorona_RUP48470.1:0.1989967226,Endogone sp. FLAS-
F59071_RUS21607.1:0.2307109339)/1:0.5497012257)/1:0.3314736272,(Umbelopsis
isabellina_KAG2171576.1:0.3013895101,Umbelopsis
vinacea_KAG2173516.1:0.3972717235)/1:0.8748631998)/1:0.6080260006,Syncephalastru
m racemosum_ORZ02314.1:1.1222766283)/0.786:0.0462225087,Phycomyces blakesleeanus
NRRL 1555_OAD70725.1:0.7602900023)/0.996:0.0867649602,(Apophysomyces
ossiformis_KAF7730861.1:0.3129523211,Apophysomyces sp.
BC1015_KAG0167084.1:0.2185300568)/1:0.5333893317)/0.994:0.0807798107,((Absidia
glauca_SAM09346.1:0.2947639604,Absidia
repens_ORZ10530.1:0.2712927200)/1:0.2864268340,Hesseltinella
vesiculosa_ORX62870.1:1.0291617752)/1:0.3684180061)/1:0.4765037247,((Rhizopus
delemar RA 99-880_EIE91574.1:0.3419790814,(Rhizopus microsporus ATCC
52813_PHZ12216.1:0.0443484425,(Rhizopus
azygosporus_RCH86323.1:0.0425084176,Rhizopus
azygosporus_RCH91569.1:0.0336700560)/1:0.0220593777)/1:0.3131253982)/
0.411:0.0337928103,Rhizopus delemar RA
99-880_EIE91575.1:0.2354393040)/1:0.4702691582)/0.997:0.1022563936,(Mucor
saturninus_KAG2213869.1:0.2693228844,Thamnidium

elegans_KAG2232755.1:0.2861091365)/1:0.2038867384)/1:0.1184031103,Choanephora
cucurbitarum_OBZ87914.1:0.4764947560)/1:0.3250478396,(Parasitella
parasitica_CEP15833.1:0.2187306239,Mucor
plumbeus_KAG2203245.1:0.1379214872)/0.999:0.0589961750)/1:0.1399505480,Mucor
lusitanicus_KAF1801677.1:0.0703752714);

****
****
FANCJ
****
****

(Mucor ambiguus_GAN01912.1:0.0803857511,((((((((((((Rhizophagus
clarus_GES76756.1:0.1097372919,(Rhizophagus irregularis DAOM 181602=DAOM
197198_POG75624.1:0.0593962892,Rhizophagus
diaphanus_RGB37783.1:0.0098727459)/1:0.0699969078)/1:0.0926984424,Glomus
cerebriforme_RIA96456.1:0.1445696423)/1:0.6699771887,Geosiphon
pyriformis_KAG9290759.1:0.8221465841)/0.999:0.2214399803,Basidiobolus
meristosporus CBS 931.73_ORX98335.1:1.3368470952)/0.99:0.2109418058,
((((((Schizosaccharomyces pombe_CAB16287.1:0.9847129311,Schizosaccharomyces
japonicus yFS275_EEB06601.2:1.4972720830)/1:1.1370178833,((((((((Blumeria
graminis f. sp. triticale_CAD6502977.1:0.9045125882,Sclerotinia sclerotiorum
1980 UF-70_EDN92053.1:1.0359472166)/0.503:0.1943223580,(Trichoderma reesei
QM6a_EGR48488.1:0.4855058867,Verticillium dahliae
VdLs.17_EGY19214.1:0.6115794362)/1:0.9844500235)/1:0.2208453029,Zymoseptoria
tritici IPO323_EGP86574.1:1.5088808226)/0.976:0.1529859711,(((Aspergillus
nidulans FGSC A4_EAA62984.1:0.6704290510,Coccidioides immitis
RS_EAS36726.3:0.8613745787)/1:0.2240431098,Exophiala dermatitidis
NIH/UT8656_EHY58322.1:1.2755094935)/0.994:0.1716203982,Bacidia
gigantensis_KAG8529327.1:0.7993453069)/1:0.1911635642)/1:0.1686221556,(Tuber
melanosporum_CAZ82635.1:0.9591437144,((Orbilia oligospora ATCC
24927_EGX46339.1:0.3579941987,Drechslerella
brochopaga_KAF3928382.1:0.2504028739)/0.968:0.0851340744,Arthrobotrys
entomopaga_KAF3908903.1:0.1778412598)/1:0.8951295318)/0.994:0.1466211248)/
1:0.1987058646,Neolecta irregularis
DAH-3_OLL24306.1:1.3290161958)/1:0.1705349529,Pneumocystis carinii
B80_KTW27717.1:1.4239149014)/0.999:0.1054398340,(Yarrowia lipolytica
CLIB122_CAG82511.1:1.6029888580,(Candida albicans
SC5314_AOW27172.1:1.4152432776,Saccharomyces
cerevisiae_sp_P22516_CHL1_YEAST:1.7432313881)/1:0.3938383217)/1:0.7764275306)/
0.986:0.1271092445)/1:0.3337306388,((((Cryptococcus neoformans var. grubii
H99_AFR93526.2:1.4828701466,(((((((Coprinopsis cinerea
okayama7#130_EAU85951.2:0.6520250241,Amanita muscaria Koide
BX008_KIL67908.1:0.7732382567)/1:0.2583746209,(Postia placenta Mad-698-
R_EED85297.1:1.2841159232,Serpula lacrymans var. lacrymans
S7.9_EGO23609.1:0.8287873908)/0.997:0.1829893348)/1:0.3605347680,Ramaria
rubella_KAF8579814.1:0.6963962523)/0.869:0.1062149829,Dacryopinax
primogenitus_EJU03236.1:1.1358432919)/0.791:0.1229380089,Rhizoctonia
solani_QRW20304.1:1.0571600479)/0.219:0.0221526379,Rhizoctonia
solani_QRW20305.1:1.4196840002)/0.999:0.1325457940,(Dacryopinax
primogenitus_EJT96518.1:0.0000020544,Dacryopinax
primogenitus_EJU03231.1:0.0000025219)/1:1.0707551372)/1:0.3374905397)/
0.979:0.1601147622,Wallemia ichthyophaga
EXF-994_EOR01680.1:1.8750373629)/1:0.1219588410,((Puccinia graminis f. sp.
tritici CRL 75-36-700-3_EFP75463.2:0.9970786763,Melampsora larici-populina
98AG31_EGG02802.1:0.7830844930)/1:0.7513310245,Mixia osmundae IAM
14324_KEI40283.1:1.3246658411)/1:0.3652318464)/0.993:0.0886692906,(Malassezia
globosa CBS 7966_EDP42741.1:1.5287843189,(Tilletiaria anomala UBC
951_KDN45742.1:1.3476524314,Ustilago maydis
521_KIS68622.1:0.9297956668)/0.999:0.2200326889)/1:0.6106856220)/1:0.3720278776)
/1:0.1765230381,((Entomophthora muscae_KAF7748174.1:2.0722806271,Conidiobolus
coronatus NRRL 28638_KXN74979.1:1.7198116807)/1:0.5807067541,((Allomyces
macrogynus ATCC 38327_KNE56807.1:1.9212338952,Catenaria anguillulae
PL171_ORZ39452.1:1.2662996312)/1:1.0751799650,(((((Smittium
mucronatum_OLY83698.1:0.7417930733,(Smittium

culicis_OMJ18951.1:0.0000028852,Smittium
culicis_OMJ23568.1:0.0036600483)/1:0.5379265368)/1:0.4452113950,(Smittium
simulii_PVU97932.1:0.7558784399,Smittium
megazygosporum_PVV04678.1:1.6257161167)/1:0.2370933572)/1:0.1489904582,
(Furculomyces boomerangus_PVU96159.1:0.0024418797,Smittium
angustum_PWA00233.1:0.0024341802)/1:0.7001205609)/1:0.4684919492,(Zancudomyces
culisetae_OMH80602.1:0.0000020869,Zancudomyces
culisetae_OMH82001.1:0.0047403841)/1:1.0127335200)/1:1.0160921347,(Linderina
pennispora_ORX72489.1:0.6221821103,Coemansia reversa NRRL
1564_PIA13365.1:0.7332200697)/1:1.0593822592)/1:0.5354572712)/0.396:0.0633166042
)/1:0.1710247670)/1:0.0984702503,((((((((((((((((Parasitella
parasitica_CEP10455.1:0.2495207932,Mucor
lusitanicus_KAF1803765.1:0.2969920140)/0.967:0.0514626681,Mucor
plumbeus_KAG2205872.1:0.2882300832)/1:0.6089601104,Choanephora
cucurbitarum_OBZ86540.1:0.7726466145)/1:0.3754857287,Mucor
saturninus_KAG2192614.1:0.9783030957)/1:0.1897616366,(Rhizopus delemar RA 99-
880_EIE76120.1:0.6277434022,(Rhizopus delemar RA 99-880_EIE77313.1:0.3768176654,
((Rhizopus microsporus ATCC 52813_PHZ13700.1:0.0436001169,Rhizopus
azygosporus_RCI00371.1:0.0459071039)/0.924:0.0347010334,Rhizopus
azygosporus_RCH85262.1:0.0219384320)/1:0.4350624597)/1:0.1151704927)/
1:0.5450981768)/1:0.3251056943,((Absidia glauca_SAM09669.1:0.3036212511,Absidia
repens_ORZ11058.1:0.4208316788)/1:0.5907022036,Hesseltinella
vesiculosa_ORX56159.1:1.2375957689)/1:0.4624868036)/0.965:0.0725486539,
(((Apophysomyces ossiformis_KAF7722068.1:0.4433170196,(Apophysomyces sp.
BC1015_KAG0162853.1:0.0000024423,Apophysomyces sp.
BC1021_KAG0172792.1:0.0000022671)/1:0.4353698204)/1:0.9841233259,(Mucor
circinatus_KAG2226344.1:1.2034979270,Syncephalastrum
racemosum_ORY98222.1:1.1378910026)/1:0.4687109093)/1:0.1430211616,Phycomyces
blakesleeanus NRRL
1555_OAD65243.1:1.2011843200)/0.991:0.0745002975)/1:0.2300543133,(Absidia
glauca_SAM08073.1:4.6589268264,Absidia
repens_ORZ25718.1:0.9810849109)/1:0.9945034789)/0.993:0.1641977901,((Umbelopsis
isabellina_KAG2182432.1:0.6731811678,Umbelopsis
vinacea_KAG2188733.1:0.5502710435)/1:0.9527970631,(Jimgerdemannia
flammicorona_RUP49480.1:0.7620592670,(Endogone sp. FLAS-
F59071_RUS20705.1:0.0000022546,Endogone sp. FLAS-
F59071_RUS20706.1:0.0355896821)/1:0.5281119416)/1:0.7132929492)/
0.995:0.1952419597)/1:0.3065956105,(Linderina
pennispora_ORX69163.1:0.9642473277,Coemansia reversa NRRL
1564_PIA13149.1:0.9436630658)/1:1.5569695739)/0.992:0.1114920715,((((Olpidium
bornovanus_KAG5456416.1:0.0210677061,Olpidium
bornovanus_KAG5459303.1:0.1544183108)/1:0.7578245612,Olpidium
bornovanus_KAG5460589.1:1.7729370705)/1:0.4325806246,(((Allomyces macrogynus
ATCC 38327_KNE57240.1:0.0156368324,Allomyces macrogynus ATCC
38327_KNE65964.1:0.2427441370)/0.758:0.0311611700,Allomyces macrogynus ATCC
38327_KNE65972.1:0.1230901382)/1:1.1220802534,Catenaria anguillulae
PL171_ORZ33420.1:1.3084427207)/1:0.7738077201)/1:0.2755333103,((Gonapodya
prolifera JEL478_KXS17118.1:1.5268709692,((Blyttiomyces
helicus_RKO85320.1:2.2598278657,Synchytrium
microbalum_TPX34559.1:1.1787087136)/0.827:0.2256355480,Blyttiomyces
helicus_RKO89259.1:1.5797917446)/0.993:0.2692758572)/0.996:0.1939277115,(Homo
sapiens_sp_Q9BX63_FANCJ_HUMAN:0.2678814114,Mus
musculus_sp_Q5SXJ3_FANCJ_MOUSE:0.1879447874)/1:1.8252048694)/0.988:0.1461758931)
/0.981:0.0957113403)/1:0.1811810921,(((Nosema bombycis
CQ1_EOB12297.1:0.0111571319,Nosema bombycis
CQ1_EOB13222.1:0.0000023106)/1:1.7954795172,Dictyocoela
roeselum_KAG0419496.1:2.9996739263)/1:1.0710298569,Paramicrosporidium
saccamoebae_PJF18013.1:1.5256231606)/1:0.8025156477)/0.653:0.0690486307,
((((((Dissophora ornata_KAF8935261.1:0.5618514816,Dissophora
globulifera_KAG0329215.1:0.7176071900)/1:0.2087750678,(((((Entomortierella
lignicola_KAF8986447.1:0.0129300749,Haplosporangium sp. Z
27_KAF9207679.1:0.0443926799)/1:0.4827609881,(Mortierella sp.
AM989_KAF9116314.1:0.4132245972,(((Mortierella sp.
AD010_KAF9171454.1:0.0000022101,Mortierella sp.

AD011_KAF9398539.1:0.0015551020)/1:0.0554278924,Entomortierella
chlamydospora_KAG0006130.1:0.0249789988)/1:0.1594388785,Mortierella sp.
AD094_KAF9351726.1:0.0876388993)/1:0.2489020169)/0.994:0.0768971358)/
1:0.1499266922,Entomortierella
beljakovae_KAF9438368.1:0.7385930467)/1:0.2373237192,Lobosporangium
transversale_ORZ15459.1:0.8487788562)/0.823:0.0906587863,Mortierella sp.
NVP85_KAF9343248.1:1.6860726954)/0:0.0000011574)/1:0.0709770633,
((((((Haplosporangium gracile_KAF8944385.1:0.0505312138,Linnemannia
schmuckeri_KAF9149935.1:0.0428497830)/1:0.0642878733,((Mortierella sp.
GBA39_KAF9138078.1:0.0209201723,(Mortierella
hygrophila_KAF9547198.1:0.0373956413,Linnemannia
hyalina_KAG9064061.1:0.0219604495)/1:0.0073772396)/1:0.0504978156,Linnemannia
elongata
AG-77_OAQ24143.1:0.0689584270)/1:0.0687796668)/1:0.0454303005,Linnemannia
gamsii_KAG0297868.1:0.1621452532)/1:0.0815122546,((Mortierella sp.
14UC_KAF9130769.1:0.1014156939,Linnemannia
zychae_KAF9907262.1:0.1452358638)/1:0.0555210807,(Linnemannia
exigua_KAG0268519.1:0.1192878400,Mortierella sp.
AD032_KAG0375170.1:0.0823152829)/1:0.0630183749)/1:0.1198365460)/1:0.1071732385,
((Mortierella sp. AD031_KAF9093634.1:0.0067507798,Mortierella sp.
GBA35_KAF9104791.1:0.0033203109)/0.976:0.0016094820,Mortierella sp.
NVP41_KAG0218376.1:0.0040272331)/1:0.2407060261)/1:0.4841081907,
(((Haplosporangium bisporale_KAF8988006.1:0.0026911034,Podila verticillata NRRL
6337_KFH69534.1:0.0015595836)/1:0.1897648738,(Podila
horticola_KAF9321611.1:0.2130094386,(Podila
epicladia_KAG0096794.1:0.0981553636,Podila
minutissima_KAG0358707.1:0.0504255685)/1:0.1725899669)/1:0.1856221335)/
1:0.1611200678,Podila humilis_KAG0340558.1:0.5537176873)/1:0.2854063981,Podila
epigama_KAF9421671.1:1.0375676514)/1:0.2734128820)/0.995:0.0816767884,
(((Haplosporangium sp. Z 767_KAF9183665.1:0.0000026807,Haplosporangium sp. Z
11_KAF9187812.1:0.0000021076)/1:0.0942777935,Mortierella
polycephala_KAG0260437.1:0.1337286855)/1:0.5818375880,(Mortierella
antarctica_KAF9984021.1:0.5842157527,Mortierella sp.
GBA30_KAG0216106.1:0.4236482830)/1:0.2802962434)/1:0.0759255271)/1:0.1080505714)
/1:0.1493588307,Lunasporangiospora
selenospora_KAF9584990.1:1.2442642889)/0.392:0.0505943325,Mortierella sp.
GBA43_KAG0244286.1:1.0259150198)/1:0.4276113252,(Actinomortierella
wolfii_KAG0236526.1:0.3176565601,Actinomortierella
ambigua_KAG0258858.1:0.5208834023)/1:1.2230029595)/1:0.4443830758)/
1:0.4614914316,Dictyostelium discoideum
AX4_XP_637574.1:2.7431454042)/1:0.5261527619,(Allomyces
macrogynus ATCC 38327_KNE73400.1:1.9202481801,(Paramicrosporidium
saccamoebae_PJF18139.1:1.6500545954,Jimgerdemannia
flammicorona_RUP44246.1:1.8534995253)/0.999:0.4220671448)/1:0.5187362343)/
0.921:0.2579387051,Pseudoloma
neurophilia_KRH92890.1:3.4720530460)/1:1.4486990041,Paramicrosporidium
saccamoebae_PJF18615.1:2.6545557667)/0.995:0.2531919102,Caulochytrium
protostelioides_RKP01865.1:2.3928386755)/1:0.4845573122,((Batrachochytrium
dendrobatidis JAM81_EGF80098.1:1.8475446737,(((Spizellomyces punctatus DAOM
BR117_KNC95958.1:0.0451950111,Spizellomyces sp.
'palustris'_TPX68230.1:0.1041505355)/1:0.8015511512,Powellomyces
hirtus_TPX61643.1:0.8109615179)/1:0.5617021479,((Rhizoclosmatium
globosum_ORY24846.1:0.0076817766,Rhizoclosmatium
globosum_ORY32485.1:0.0302984696)/1:0.6463327107,Chytriomyces
confervae_TPX76091.1:0.6898844206)/1:0.9671001682)/0.379:0.1215266734)/
0.996:0.1977831112,((((Neocallimastix sp. JGI-
2020a_KAG4105813.1:0.0000023992,Neocallimastix
californiae_ORY28694.1:0.0058671198)/1:0.1993281697,(Piromyces
finnis_ORX45561.1:0.1627520817,Piromyces sp.
E2_OUM58708.1:0.1350226352)/1:0.0896854947)/0.894:0.0696159975,Anaeromyces
robustus_ORX85967.1:0.1057239213)/1:1.4042651262,Gonapodya prolifera
JEL478_KXS13688.1:2.2793050005)/0.868:0.1538511164)/1:0.2076275207)/
1:0.1600377325)/1:0.1258083689,((((((Haplosporangium
bisporale_KAF8935067.1:0.0000026921,Podila verticillata NRRL

6337_KFH65683.1:0.0039855383)/1:0.1523091965,((Podila
horticola_KAF9312900.1:0.1111093487,Podila
clonocystis_KAG0031020.1:0.1177578734)/0.857:0.0082518130,(Podila
epicladia_KAG0092493.1:0.1181519696,Podila
minutissima_KAG0356106.1:0.0411905080)/1:0.0188369193)/1:0.0708304134)/
1:0.2419405440,Podila
humilis_KAG0335186.1:0.6430614739)/0.993:0.0705222784,Podila
epigama_KAF9425369.1:0.5325662080)/1:0.1765927927,(((((((Dissophora
ornata_KAF8939783.1:0.1661311806,Dissophora
globulifera_KAG0307282.1:0.3154645256)/0.995:0.0311724601,Lobosporangium
transversale_ORZ07234.1:0.4505134036)/0.998:0.0364170796,((Mortierella sp.
NVP85_KAF9353576.1:0.1937213254,Mortierella sp.
GBA43_KAG0242257.1:0.2377082914)/1:0.1174663931,Gamsiella
multidivaricata_KAG0370665.1:0.2741284834)/1:0.0410636176)/1:0.0356941183,
(((Entomortierella lignicola_KAF8979262.1:0.0092019496,Haplosporangium sp. Z
27_KAF9203423.1:0.0063362164)/1:0.1494515698,(Mortierella sp.
AM989_KAF9116824.1:0.2075552530,((Mortierella sp.
AD010_KAF9173392.1:0.0527489044,Entomortierella
chlamydospora_KAG0001055.1:0.0395600196)/1:0.0610571543,Mortierella sp.
AD094_KAF9352346.1:0.0554955921)/1:0.0625423432)/1:0.0363953742)/1:0.0413447268,
Entomortierella
beljakovae_KAF9431667.1:0.3096500972)/1:0.1444603724)/1:0.0791219994,
(((Haplosporangium sp. Z 767_KAF9187050.1:0.0000022845,Haplosporangium sp. Z
11_KAF9188409.1:0.0000020991)/1:0.0465009415,Mortierella
polycephala_KAG0256848.1:0.0721720604)/1:0.2534373209,(Mortierella
antarctica_KAF9990293.1:0.3013028558,Mortierella sp.
GBA30_KAG0209113.1:0.2662808152)/1:0.1127204775)/0.96:0.0484827723)/
1:0.0567903836,(((((Haplosporangium
gracile_KAF8946397.1:0.0711471893,Linnemannia
schmuckeri_KAF9156440.1:0.0521009296)/1:0.0236226186,(((Mortierella sp.
GBA39_KAF9128473.1:0.0303311816,Linnemannia
hyalina_KAG9068956.1:0.0291621560)/0.949:0.0026048365,Mortierella
hygrophila_KAF9541234.1:0.0334108028)/1:0.0332700854,Linnemannia elongata AG-
77_OAQ26135.1:0.0583072173)/1:0.0639597236)/1:0.0393703031,Linnemannia
gamsii_KAG0275228.1:0.1167469051)/1:0.0692140247,((Mortierella sp.
14UC_KAF9136116.1:0.0716797727,Linnemannia
zychae_KAF9911357.1:0.0881013377)/1:0.0429483993,(Linnemannia
exigua_KAG0276503.1:0.0617916360,Mortierella sp.
AD032_KAG0371448.1:0.0559353421)/1:0.0479906384)/1:0.0940850132)/1:0.0542507297,
((Mortierella sp. AD031_KAF9086721.1:0.0012155039,Mortierella sp.
GBA35_KAF9103896.1:0.0012152273)/0.252:0.0004208118,Mortierella sp.
NVP41_KAG0203919.1:0.0044460108)/1:0.1582930727)/1:0.2843527519)/0.999:0.0529283
224,Gryganskiella
cystojenkinii_KAG0052493.1:0.4673384591)/0.807:0.0622098532,Lunasporangiospora
selenospora_KAF9582943.1:0.5596813473)/1:0.1007432507)/1:0.2157971447,
(Actinomortierella wolfii_KAG0241451.1:0.3705048398,Actinomortierella
ambigua_KAG0250681.1:0.2685580717)/1:0.3048048840)/1:1.0639393115)/
0.979:0.0964527354,(((Thamnocephalis
sphaerospora_RKP06517.1:0.0244919175,Thamnocephalis
sphaerospora_RKP08780.1:0.1995387035)/1:1.0242232703,Piptocephalis
cylindrospora_RKP12669.1:1.9619472911)/1:0.3916795349,Dimargaris
cristalligena_RKP37098.1:1.6427632301)/0.993:0.2159643893)/1:0.2068559460)/
1:0.1763224771,(Jimgerdemannia flammicorona_RUP49912.1:0.3199448628,Endogone sp.
FLAS-F59071_RUS17736.1:0.4657779356)/1:0.6592903915)/1:0.3282457301,(Umbelopsis
isabellina_KAG2174939.1:0.4554517255,Umbelopsis
vinacea_KAG2185177.1:0.4511342556)/1:0.4912739637)/1:0.4112848105,
((Apophysomyces ossiformis_KAF7721013.1:0.4545073890,Apophysomyces sp.
BC1015_KAG0166449.1:0.2068910164)/1:0.7196316404,(Mucor
circinatus_KAG2221187.1:0.7987858779,Syncephalastrum
racemosum_ORZ01236.1:0.8312165026)/1:0.2050941862)/0.894:0.0856640102)/
0.623:0.0674538528,(Phycomyces blakesleeanus NRRL 1555_OAD67841.1:0.7622301784,
(Hesseltinella vesiculosa_ORX62296.1:0.9293671391,Absidia
repens_ORZ12843.1:0.5870751137)/1:0.3563261351)/0.993:0.0998621330)/
1:0.3533685989,(Rhizopus delemar RA 99-880_EIE91320.1:0.3479117177,(Rhizopus

delemar RA 99-880_EIE91321.1:0.2175389617,(Rhizopus
azygosporus_RCH96665.1:0.0222871665,Rhizopus
azygosporus_RCH97083.1:0.0311956582)/1:0.2347290776)/0.335:0.0325553517)/
1:0.2776347672)/1:0.1744253707,Choanephora
cucurbitarum_OBZ91603.1:0.4477413373)/0.99:0.0755496105,(Mucor
saturninus_KAG2210921.1:0.2384872720,Thamnidium
elegans_KAG2237618.1:0.1723890337)/1:0.2122856836)/1:0.1413389510,Parasitella
parasitica_CEP13877.1:0.1758829941)/1:0.0365467854,Mucor
plumbeus_KAG2204615.1:0.1416932058)/1:0.0796383838,Mucor
lusitanicus_KAF1798358.1:0.0643755873);

****
****
FANCL
****
****
(Mucor ambiguus_GAN04869.1:0.0867615032,(((((((((((((((Rhizophagus
clarus_GES94643.1:0.0000023197,Rhizophagus
clarus_GES94644.1:0.0161978400)/0.129:0.0000027054,Rhizophagus
clarus_GES94645.1:0.1024787361)/1:0.0465532554,(Rhizophagus irregularis DAOM
181602=DAOM 197198_POG78854.1:0.0000022035,(Rhizophagus irregularis DAOM
181602=DAOM 197198_POG78855.1:0.1586484789,Rhizophagus
diaphanus_RGB24208.1:0.0000023337)/0.367:0.0036121697)/1:0.0437130503)/
0.988:0.0277435484,Glomus cerebriforme_RIA97495.1:0.0679772995)/1:0.2491412108,
(((Gigaspora margarita_KAF0501354.1:0.0000026808,Gigaspora
margarita_KAF0501355.1:0.0000031368)/0.793:0.0092365506,Gigaspora
rosea_RIB17933.1:0.0310992743)/1:0.1794826768,Diversispora
epigaea_RHZ59955.1:0.3868698088)/1:0.0883481878)/1:0.1817973742,Geosiphon
pyriformis_KAG9288637.1:0.4527129026)/0.988:0.0979999087,Basidiobolus
meristosporus CBS 931.73_ORY06328.1:0.4760576335)/1:0.1844504146,Jimgerdemannia
flammicorona_RUP50519.1:1.0550651827)/0.496:0.0359220811,(((((Batrachochytrium
dendrobatidis JAM81_EGF78132.1:1.3902496376,Blyttiomyces
helicus_RKO88049.1:0.7740099651)/0.349:0.1144766899,Salpingoeca
rosetta_XP_004990350.1:1.4375935944)/0.753:0.2080354502,Amphimedon
queenslandica_XP_019849844.1:0.8778915436)/0.653:0.0694486231,((sp|Q9NW38|
FANCL_HUMAN:0.1042555044,sp|Q9CR14|FANCL_MOUSE:0.1719005909)/1:0.6022824284,
(Pomacea canaliculata_PVD26310.1:0.6274301384,Capsaspora owczarzaki ATCC
30864_XP_004349480.1:0.7482512031)/0.993:0.1931838549)/1:0.2120354574)/
1:0.1381305150,(((((((Haplosporangium bisporale_KAF8924440.1:0.0000027136,Podila
verticillata NRRL 6337_KFH72826.1:0.0000023741)/1:0.0795184035,((Podila
horticola_KAF9310279.1:0.0604444091,Podila
minutissima_KAG0352122.1:0.0539663659)/0.912:0.0080302965,Podila
clonocystis_KAG0024688.1:0.0431218010)/1:0.0412029919)/1:0.1308627844,Podila
humilis_KAG0347694.1:0.3484188915)/1:0.1050530000,Podila
epigama_KAF9423182.1:0.2747645203)/1:0.1076037484,(((((Haplosporangium
gracile_KAF8937559.1:0.0220429008,Linnemannia
schmuckeri_KAF9150703.1:0.0310373314)/1:0.0140412050,((Mortierella sp.
GBA39_KAF9139701.1:0.0223096870,(Mortierella
hygrophila_KAF9550070.1:0.0159039165,Linnemannia
hyalina_KAG9073178.1:0.0230952240)/0.127:0.0000023369)/1:0.0298353792,Linnemanni
a elongata AG-77_OAQ25702.1:0.0391942692)/1:0.0208817275)/0.998:0.0165724238,
(((Mortierella sp. 14UC_KAF9126808.1:0.0427699169,Mortierella sp.
AD032_KAG0374144.1:0.0593008144)/0.357:0.0088697631,Linnemannia
exigua_KAG0273171.1:0.0811287807)/1:0.0799078587,Linnemannia
gamsii_KAG0292926.1:0.1159013303)/1:0.0552187300)/1:0.0571347889,(Mortierella
sp. AD031_KAF9082479.1:0.0000027777,Mortierella sp.
NVP41_KAG0199236.1:0.0469212080)/1:0.0790477322)/1:0.1440684616,
(((((Entomortierella lignicola_KAF8978333.1:0.0178878184,Haplosporangium sp. Z
27_KAF9203380.1:0.0044454540)/1:0.1798062981,Entomortierella
beljakovae_KAF9435420.1:0.3136762179)/1:0.1320373888,(Mortierella sp.
NVP85_KAF9362995.1:0.1190177449,(Modicella
reniformis_KAF9939158.1:0.1064832718,Mortierella sp.
GBA43_KAG0227739.1:0.1993947367)/0.843:0.0767841703)/0.928:0.0421464386)/
0.992:0.0252364277,(((Lunasporangiospora

selenospora_KAF9585314.1:0.5505801001,Lobosporangium
transversale_ORZ08813.1:0.2743883281)/0.997:0.0767539377,Gamsiella
multidivaricata_KAG0357499.1:0.3052289656)/0.759:0.0241036192,Dissophora
globulifera_KAG0327689.1:0.2243437531)/0.947:0.0243430200)/1:0.0532990960,
((((Haplosporangium sp. Z 11_KAF9180047.1:0.0000022830,Haplosporangium sp. Z
767_KAF9180101.1:0.0000022202)/1:0.0318680847,Mortierella
polycephala_KAG0253091.1:0.0463494408)/1:0.2004939158,(Mortierella
antarctica_KAF9990683.1:0.2015914709,Mortierella sp.
GBA30_KAG0206660.1:0.3773312140)/1:0.1871899022)/0.831:0.0354019229,Gryganskiell
a
cystojenkinii_KAG0053592.1:0.3396465711)/0.971:0.0288409827)/0.994:0.0460676168)
/1:0.1372151423)/1:0.3717738807,Actinomortierella
ambigua_KAG0251427.1:0.9126557289)/1:0.3531926062,(Gonapodya prolifera
JEL478_KXS21705.1:0.9742838798,(Rhizoclosmatium
globosum_ORY40408.1:1.6937388754,Drosophila
melanogaster_AHN57265.1:2.4478503214)/0.426:0.1152986966)/0.996:0.1824257053)/
0.996:0.1300000285)/0.969:0.1050177885)/0.754:0.0426972104,((Allomyces
macrogynus ATCC 38327_KNE55593.1:0.1987797495,Allomyces macrogynus ATCC
38327_KNE58402.1:0.0000029245)/1:1.3847572093,Catenaria anguillulae
PL171_ORZ31903.1:1.9551365561)/1:0.9076338437)/0.994:0.1046717002,
(((Entomophthora muscae_KAF7725090.1:0.0000027867,Entomophthora
muscae_KAF7748860.1:0.0145972155)/1:1.4372857882,((((Smittium
mucronatum_OLY77915.1:1.1171686718,Furculomyces
boomerangus_PVV00056.1:1.1327936048)/0.995:0.3074786241,Smittium
simulii_PVU97027.1:1.1541065819)/1:0.4476983684,Coemansia reversa NRRL
1564_PIA19577.1:1.5766617544)/1:0.2952299806,(Thamnocephalis
sphaerospora_RKP10238.1:1.3269839694,Dimargaris
cristalligena_RKP39694.1:1.0976724091)/0.866:0.1884204510)/0.964:0.1155543093)/
0.893:0.0489474420,Monosiga brevicollis
MX1_XP_001743224.1:1.7824582238)/0.454:0.0305355262)/0.997:0.0667070918,
(Bifiguratus adelaidae_OZJ03670.1:1.6215159943,(Synchytrium
microbalum_TPX30102.1:0.4899812734,Synchytrium
endobioticum_TPX53587.1:0.2646641423)/1:1.1596498388)/0.967:0.2612793762)/
0.656:0.0319087044,(((Umbelopsis vinacea_KAG2176848.1:0.5311864754,Umbelopsis
isabellina_KAG2181111.1:0.6480733148)/1:0.5459081487,Piptocephalis
cylindrospora_RKP11639.1:2.0984195597)/0.856:0.1678873528,(((Neocallimastix sp.
JGI-2020a_KAG4097016.1:0.0000028691,Neocallimastix
californiae_ORY72342.1:0.0000021924)/0.988:0.0751380758,(Piromyces
finnis_ORX55314.1:0.0888657772,Piromyces sp.
E2_OUM59438.1:0.0824791694)/0.818:0.0467628484)/1:1.0237376375,(Conidiobolus
coronatus NRRL 28638_KXN66664.1:1.5489424946,Dictyostelium discoideum
AX4_XP_629481.1:1.1401926039)/0.397:0.1018029136)/0.997:0.2898384052)/
0.535:0.1162909489)/1:0.3432972304,(((Absidia
glauca_SAM06879.1:0.2981968378,Absidia
repens_ORZ17037.1:0.3335093477)/1:0.1736835603,Hesseltinella
vesiculosa_ORX50108.1:0.7421820190)/1:0.3459866645,((Apophysomyces
ossiformis_KAF7721028.1:0.3049223529,(Apophysomyces sp.
BC1015_KAG0172645.1:0.0000021677,Apophysomyces sp.
BC1034_KAG0189674.1:0.0000024139)/1:0.2700446591)/1:0.4807217230,(Mucor
circinatus_KAG2221802.1:0.9150652414,(Phycomyces blakesleeanus NRRL
1555_OAD77547.1:0.5594863476,Syncephalastrum
racemosum_ORZ01405.1:0.9338240284)/0.958:0.1114745791)/0.46:0.0843620774)/
0.994:0.0880872900)/0.983:0.0827565429)/1:0.4269827546,(Rhizopus delemar RA 99-
880_EIE90477.1:0.3044550831,(Rhizopus microsporus ATCC
52813_PHZ13256.1:0.0980442511,Rhizopus
azygosporus_RCH94792.1:0.0000024117)/1:0.2663255885)/1:0.2419608151)/
1:0.1666319093,(Mucor saturninus_KAG2213491.1:0.1951887917,Thamnidium
elegans_KAG2231704.1:0.3303700293)/1:0.2600178663)/0.999:0.1288020861,Choanephor
a cucurbitarum_OBZ85092.1:0.5863193665)/1:0.3840305074,(Parasitella
parasitica_CEP18045.1:0.2729237184,Mucor
plumbeus_KAG2191379.1:0.0933216847)/1:0.1117998137)/0.965:0.0410492306,Mucor
lusitanicus_KAF1806185.1:0.0313181060);

****

****
FANCM
****
****
(Podila epigama_KAF9426991.1:0.6220217855,((((((((((Homo sapiens_NP_065988.1:0.2014601231,Mus musculus_NP_849243.2:0.3062613413)/1:0.8653246468,Pomacea canaliculata_PVD26266.1:1.0425319620)/1:0.3139677978,Drosophila melanogaster_AAF55897.2:1.8804984755)/1:0.1490053024,(Olpidium bornovanus_KAG5455736.1:1.1187881514,Dimargaris cristalligena_RKP33636.1:1.2768848591)/0.885:0.1745530481)/0.999:0.1317610528,((((Blyttiomyces helicus_RKO92847.1:0.7137336108,((Spizellomyces punctatus DAOM BR117_KNC96281.1:0.0454180180,Spizellomyces sp. 'palustris'_TPX65811.1:0.0615315703)/1:0.9353858540,Powellomyces hirtus_TPX61370.1:0.7621799321)/0.994:0.1088558778)/1:0.1465847631,(Rhizoclosmatium globosum_ORY52110.1:0.9186328943,Chytriomyces confervae_TPX77543.1:0.6669938873)/1:0.4510375082)/0.99:0.1134006536,(Batrachochytrium dendrobatidis JAM81_EGF84245.1:0.8065136198,Blyttiomyces helicus_RKO87912.1:1.1820132697)/0.99:0.2606498884)/1:0.1586435284,Rozella allomycis CSF55_EPZ32328.1:1.6798435172)/0.846:0.0417053560)/0.872:0.0401875724,((Gonapodya prolifera JEL478_KXS20023.1:1.5257707095,Paramicrosporidium saccamoebae_PJF17574.1:1.4497967361)/0.996:0.2064772056,(Thamnocephalis sphaerospora_RKP07512.1:1.2356706632,Piptocephalis cylindrospora_RKP12932.1:1.7051242930)/1:0.4220953250)/1:0.2201179836)/0.999:0.0572345928,((((((((((Parasitella parasitica_CEP17934.1:0.2742236492,Mucor plumbeus_KAG2196177.1:0.2491969847)/1:0.0690406552,(Mucor ambiguus_GAN04941.1:0.1025170534,Mucor lusitanicus_KAF1799200.1:0.1732036992)/1:0.2468594278)/1:0.4064780216,Choanephora cucurbitarum_OBZ85207.1:0.6201767416)/0.997:0.0704698171,((Mucor saturninus_KAG2202402.1:0.3682203675,Mucor saturninus_KAG2213388.1:0.7691495206)/1:0.1736646175,Thamnidium elegans_KAG2231319.1:0.5326670137)/1:0.2100830288)/0.981:0.0993407443,(Rhizopus delemar RA 99-880_EIE89293.1:0.3526682152,(Rhizopus microsporus ATCC 52813_PHZ17749.1:0.0061049340,Rhizopus azygosporus_RCH90460.1:0.0246416088)/1:0.3008872978)/1:0.5024656209)/1:0.5007424413,((Absidia glauca_SAL99886.1:0.4796386230,Absidia repens_ORZ19347.1:0.5149730673)/1:0.3806992880,Hesseltinella vesiculosa_ORX58999.1:1.0285725698)/1:0.3122203553)/0.988:0.0862450039,((Apophysomyces ossiformis_KAF7729998.1:0.3524520353,Apophysomyces sp. BC1015_KAG0174207.1:0.3438215682)/1:0.4677672157,Phycomyces blakesleeanus NRRL 1555_OAD69888.1:0.6583101072)/1:0.1767047758)/0.76:0.0588016796,(Syncephalastrum racemosum_ORZ02607.1:0.9892159043,Mucor circinatus_KAG2217039.1:0.8116017703)/1:0.1785935967)/1:0.1290668419,(Umbelopsis isabellina_KAG2186213.1:0.5337833563,Umbelopsis vinacea_KAG2188360.1:0.3724118260)/1:0.6953383303)/1:0.2895385510,(Bifiguratus adelaidae_OZJ02481.1:1.0381946211,(Jimgerdemannia flammicorona_RUP43351.1:2.2148141486,Endogone sp. FLAS-F59071_RUS19389.1:0.8243784769)/0.863:0.1863360094)/1:0.1469009049)/1:0.1282869645,((Basidiobolus meristosporus CBS 931.73_ORX98858.1:0.2547114042,Basidiobolus meristosporus CBS 931.73_ORY07243.1:0.4444155616)/1:0.6551741714,Entomophthora muscae_KAF7748688.1:1.3193077250)/1:0.1622609149)/1:0.0827004441)/0.939:0.0437447067,(((((((((Bacidia gigantensis_KAG8527002.1:0.6640165783,Exophiala dermatitidis NIH/UT8656_EHY55280.1:0.6425526270)/1:0.1217989026,Coccidioides immitis RS_EAS33746.3:0.7774151913)/0.982:0.0670675627,Zymoseptoria tritici IPO323_EGP88167.1:0.8633205879)/0.944:0.1080090105,((Blumeria graminis f. sp. triticale_CAD6504314.1:0.5755916566,Sclerotinia sclerotiorum 1980 UF-70_EDO01590.1:0.5354254017)/1:0.1824538283,(Neurospora crassa OR74A_EAA34786.1:0.5073853514,(Trichoderma reesei QM6a_EGR52674.1:0.5104209315,Verticillium dahliae VdLs.17_EGY21711.1:0.4507813933)/1:0.1243657680)/1:0.2641571687)/1:0.1847194645)/1:0.4407242697,((Orbilia oligospora ATCC 24927_EGX49662.1:0.1910055644,

(Arthrobotrys entomopaga_KAF3905550.1:0.2257257777,Drechslerella
brochopaga_KAF3920859.1:0.3101046483)/0.891:0.0743003111)/1:0.6586058437,Tuber
melanosporum_CAZ84534.1:0.9044864252)/1:0.2026079390)/1:0.2027047260,Neolecta
irregularis DAH-3_OLL27108.1:1.2927192069)/0.988:0.0987946906,
(((Schizosaccharomyces japonicus
yFS275_EEB07211.1:0.6062220852,Schizosaccharomyces
pombe_CAB57423.2:0.6649456161)/1:0.6049510299,(Schizosaccharomyces
pombe_CAC19734.1:1.1910218897,Schizosaccharomyces japonicus
yFS275_EEB07593.1:0.8846862062)/1:1.1244793989)/0.997:0.1633988590,Pneumocystis
carinii B80_KTW30691.1:1.2581712841)/1:0.1908372246)/1:0.1663118026,((Candida
albicans SC5314_AOW29345.1:0.8411759088,Saccharomyces
cerevisiae_NP_012267.1:0.7393827738)/1:0.6759647680,Yarrowia lipolytica
CLIB122_CAG82387.2:1.8117406569)/1:0.3211187480)/1:0.1205200897,(((((Dacryopinax
primogenitus_EJT98921.1:0.9793975244,(Amanita muscaria Koide
BX008_KIL71049.1:0.7397335017,(Serpula lacrymans var. lacrymans
S7.9_EGO21290.1:0.5191794401,Ramaria
rubella_KAF8587346.1:0.8394735976)/0.973:0.1262489216)/1:0.2437986989)/
1:0.1553837305,Rhizoctonia solani_QRW18968.1:1.3650608106)/0.941:0.0788294595,
(Cryptococcus neoformans var. grubii H99_AFR98393.2:1.2012931217,Wallemia
ichthyophaga EXF-994_EOR02812.1:1.2523478690)/1:0.2192027406)/1:0.1281885859,
(Malassezia globosa CBS 7966_EDP41956.1:1.3108736434,(Tilletiaria anomala UBC
951_KDN37577.1:1.0687784002,Ustilago maydis
521_KIS70993.1:0.8442394272)/1:0.1658168771)/1:0.4349339128)/1:0.0955341364,
((Puccinia graminis f. sp. tritici CRL 75-36-700-
3_EFP92023.2:0.3393872576,Melampsora larici-populina
98AG31_EGG02918.1:0.4195863424)/1:0.8734922720,Mixia osmundae IAM
14324_KEI42522.1:1.1796505165)/1:0.3098628970)/1:0.2745889714)/0.992:0.057855929
7,((Dictyostelium discoideum AX4_XP_644014.1:1.7016645513,(Rozella allomycis
CSF55_EPZ36819.1:2.7998100341,(Smittium
mucronatum_OLY81249.1:0.6697254463,Smittium
simulii_PVU93672.1:0.7211616251)/1:1.2137830963)/1:0.9996952628)/1:0.2516690205,
(((((Smittium mucronatum_OLY82176.1:0.5197836180,Smittium
culicis_OMJ24797.1:0.5096527256)/1:0.3598081746,(Furculomyces
boomerangus_PVU99478.1:0.0000028927,Smittium
angustum_PVZ99644.1:0.0079558751)/1:0.7309820748)/1:0.1148853802,(Smittium
simulii_PVU89710.1:0.7443734921,Smittium
megazygosporum_PVV02623.1:0.7463894260)/1:0.1793383545)/0.942:0.1101403793,Zancu
domyces culisetae_OMH82903.1:1.2076076303)/1:0.5578699713,(Linderina
pennispora_ORX68305.1:0.6240696971,Coemansia reversa NRRL
1564_PIA19253.1:0.6008484273)/1:0.8803811448)/1:0.4119441336)/1:0.1237719290)/
1:0.1285127969)/1:0.1643225936,(((Allomyces macrogynus ATCC
38327_KNE58598.1:0.0000020841,Allomyces macrogynus ATCC
38327_KNE55793.1:0.3301532024)/1:0.9124701272,Catenaria anguillulae
PL171_ORZ33960.1:0.9782060248)/1:0.8008317497,Olpidium
bornovanus_KAG5462884.1:1.1358383063)/0.555:0.0697298025)/1:0.3156946340,
(Actinomortierella wolfii_KAG0228274.1:0.3108020022,Actinomortierella
ambigua_KAG0261460.1:0.3122799343)/1:0.7342626797)/1:0.2221767370,
((((((Mortierella sp. 14UC_KAF9117969.1:0.1146658277,Linnemannia
zychae_KAF9911565.1:0.1485367093)/1:0.0544869505,(Linnemannia
exigua_KAG0272880.1:0.1104671441,Mortierella sp.
AD032_KAG0374158.1:0.0889872390)/1:0.0681461862)/1:0.1420547344,(((Linnemannia
schmuckeri_KAF9151877.1:0.0707205469,Haplosporangium
gracile_KAF8948422.1:0.0578197989)/1:0.0564528214,(((Mortierella
hygrophila_KAF9547302.1:0.0422626136,Linnemannia
hyalina_KAG9068293.1:0.0244378689)/1:0.0090790508,Mortierella sp.
GBA39_KAF9146052.1:0.0295037831)/1:0.0357392680,Linnemannia elongata AG-
77_OAQ27778.1:0.0728165384)/1:0.0791608253)/1:0.0374201331,Linnemannia
gamsii_KAG0295438.1:0.1558606582)/1:0.1059652205)/1:0.1188184224,(Mortierella
sp. GBA35_KAF9092043.1:0.0000028130,(Mortierella sp.
AD031_KAF9096564.1:0.0016222987,Mortierella sp.
NVP41_KAG0208266.1:0.0032450984)/1:0.0089495488)/1:0.2208766260)/1:0.4398293047,
Gryganskiella cystojenkinii_KAG0041327.1:0.7108719005)/0.988:0.0778632596,
(((((Haplosporangium sp. Z 11_KAF9184788.1:0.0015717648,Haplosporangium sp. Z
767_KAF9185368.1:0.0000020993)/1:0.0819461776,Mortierella

polycephala_KAG0260623.1:0.0671689542)/1:0.4006636878,(Mortierella
antarctica_KAF9987996.1:0.3720258319,Mortierella sp.
GBA30_KAG0203440.1:0.2999837740)/1:0.1456881796)/1:0.1283261519,
(((((Haplosporangium sp. Z 27_KAF9199879.1:0.3147655126,((Mortierella sp.
AD094_KAF9360483.1:0.0507869297,(Entomortierella
chlamydospora_KAG0006258.1:0.0331072926,Mortierella sp.
AD010_KAF9175355.1:0.0483795164)/1:0.0976473058)/1:0.1911487027,Mortierella sp.
AM989_KAF9110573.1:0.2136989450)/1:0.0686985405)/1:0.0630071270,Entomortierella
beljakovae_KAF9435297.1:0.5252030918)/1:0.1125338270,Lobosporangium
transversale_ORZ12050.1:0.5758994864)/1:0.0723314783,(Dissophora
ornata_KAF8941149.1:0.3898354611,Gamsiella
multidivaricata_KAG0362734.1:0.4594155842)/1:0.0812258951)/1:0.0507501311,
(((Mortierella sp. NVP85_KAF9362368.1:0.2868370576,Modicella
reniformis_KAF9983232.1:0.3512043860)/0.999:0.0611520269,Mortierella sp.
GBA43_KAG0217145.1:0.4131330601)/1:0.1792208432,Dissophora
globulifera_KAG0323600.1:0.5226167128)/0.995:0.0560182547)/1:0.0840755005)/
1:0.0681153369,Lunasporangiospora
selenospora_KAF9582293.1:0.8065992201)/0.991:0.0502733874)/1:0.1260899355)/
1:0.3870973017,(Podila humilis_KAG0344141.1:0.6206998640,(((Haplosporangium
bisporale_KAF8941914.1:0.0000029019,Podila verticillata NRRL
6337_KFH67609.1:0.0000022303)/1:0.1074225082,Haplosporangium
bisporale_KAF9027359.1:0.8547843846)/1:0.1043980335,(Podila
horticola_KAF9316223.1:0.1063304463,(Podila
clonocystis_KAG0033227.1:0.1392944113,(Podila
epicladia_KAG0096837.1:0.0847397143,Podila
minutissima_KAG0363760.1:0.1020972694)/1:0.0744285871)/0.621:0.0211949808)/
1:0.0809085411)/1:0.2562858833)/1:0.3098964824);

****
FANCO
****

(Dictyostelium discoideum_XP_638535.1:1.0303980686,(Mus
musculus_NP_444499.1:0.0726412164,Homo
sapiens_NP_002867.1:0.1002063565)/1:0.7553030752,((((((((Rhizophagus
clarus_GES85838.1:0.0211185027,Rhizophagus irregularis DAOM 181602=DAOM
197198_POG80325.1:0.0275777186)/0.994:0.0333922787,Glomus
cerebriforme_RIA97301.1:0.0541710850)/1:0.2933545659,((((Gigaspora
margarita_KAF0491430.1:0.0000021718,Gigaspora
margarita_KAF0491432.1:0.0000028085)/0.125:0.0000026515,Gigaspora
margarita_KAF0491431.1:0.0000021312)/0.136:0.0000026991,Gigaspora
rosea_RIB16529.1:0.0207769472)/1:0.6112844605,Diversispora
epigaea_RHZ79069.1:0.2274436063)/1:0.2811824169)/0.75:0.0567660891,Geosiphon
pyriformis_KAG9300620.1:0.3811858140)/1:0.1405629850,((Neocallimastix sp. JGI-
2020a_KAG4094938.1:0.0851048042,Piromyces
finnis_ORX58624.1:0.1356547011)/1:0.8692231905,(((Spizellomyces punctatus DAOM
BR117_KND00383.1:0.0142929184,Spizellomyces sp.
'palustris'_TPX71167.1:0.0525759053)/1:0.2964457073,Powellomyces
hirtus_TPX61040.1:0.3159551260)/0.997:0.1517455500,((Gonapodya prolifera
JEL478_KXS20047.1:1.3292124992,Chytriomyces
confervae_TPX68649.1:0.9543539523)/0.929:0.1745680528,(Synchytrium
microbalum_TPX30708.1:1.1149077513,Synchytrium
endobioticum_TPX42121.1:0.7248462263)/1:0.6703766244)/0.989:0.1555090857)/
1:0.1787987176)/1:0.1109542138)/0.919:0.1055312219,Jimgerdemannia
flammicorona_RUP26539.1:0.5579323291)/0.973:0.0964997664,(((((((Parasitella
parasitica_CEP08957.1:0.9711385024,Mucor
saturninus_KAG2208072.1:0.9437664205)/0.973:0.1384780462,(Choanephora
cucurbitarum_OBZ87558.1:0.5481465646,(Rhizopus
azygosporus_RCH80534.1:0.0350285741,Rhizopus
azygosporus_RCH83229.1:0.0205116652)/1:0.4723429020)/1:0.1861785129)/
1:0.2399780234,((Absidia glauca_SAL99084.1:0.5176450969,Absidia
repens_ORZ15694.1:0.6412579516)/1:0.3696339361,Hesseltinella
vesiculosa_ORX47759.1:0.9607993936)/1:0.2849289542)/0.974:0.0816639154,

(Apophysomyces ossiformis_KAF7729148.1:0.5618958015,(Apophysomyces sp.
BC1015_KAG0170764.1:0.0000028699,(Apophysomyces sp.
BC1021_KAG0179254.1:0.0023893297,Apophysomyces sp.
BC1034_KAG0192433.1:0.0000022298)/1:0.0149027095)/1:0.3459727836)/
1:0.8865457655)/0.546:0.0489346771,(Mucor
circinatus_KAG2219673.1:0.8135842892,Syncephalastrum
racemosum_ORZ03530.1:0.8999881583)/0.741:0.1092843152)/1:0.3972160185,
((((((Dissophora ornata_KAF8934453.1:0.2389581332,(((Entomortierella
lignicola_KAF8981337.1:0.0345158863,Haplosporangium sp. Z
27_KAF9203268.1:0.0061116751)/1:0.2556018315,(Mortierella sp.
AM989_KAF9113704.1:0.1488356211,((Mortierella sp.
AD010_KAF9171344.1:0.0124607384,Entomortierella
chlamydospora_KAF9992504.1:0.0070606515)/1:0.0620475417,Mortierella sp.
AD094_KAF9358132.1:0.0257815046)/1:0.1145684692)/0.81:0.0446381819)/
1:0.1356962959,(Dissophora globulifera_KAG0317538.1:0.3153270223,Lobosporangium
transversale_ORZ26457.1:0.4574014788)/0.699:0.0497013111)/0.821:0.0376179732)/
0.54:0.0311737242,(Modicella reniformis_KAF9934557.1:0.1590080401,Mortierella
sp. GBA43_KAG0222719.1:0.2575206061)/1:0.0669278057)/1:0.0733871351,
(Haplosporangium sp. Z 767_KAF9183214.1:0.0329722290,Mortierella
polycephala_KAG0248987.1:0.1807526332)/1:0.3921020863)/0.879:0.0561648238,
((((Haplosporangium bisporale_KAF8985933.1:0.0000023670,Podila verticillata NRRL
6337_KFH65016.1:0.0053021278)/1:0.3517212202,Podila
epigama_KAF9430778.1:0.5842469654)/1:0.5378322553,Lunasporangiospora
selenospora_KAF9582241.1:0.5619426836)/0.776:0.0708700210,(Mortierella
antarctica_KAF9981573.1:0.2343364286,Mortierella sp.
GBA30_KAG0212275.1:0.2330595999)/1:0.1039292207)/0.949:0.0596526229)/
0.995:0.1373925594,(((((Haplosporangium
gracile_KAF8943769.1:0.0388996665,Linnemannia
schmuckeri_KAF9149699.1:0.0440478796)/1:0.0289694555,(Mortierella sp.
GBA39_KAF9124726.1:0.0213654851,(Mortierella
hygrophila_KAF9541247.1:0.0066341497,Linnemannia
hyalina_KAG9067681.1:0.0235757876)/1:0.0186581210)/1:0.0334282432)/
1:0.0357165657,Linnemannia gamsii_KAG0288921.1:0.0713614142)/1:0.0768884609,
((Mortierella sp. 14UC_KAF9127347.1:0.0949795822,Linnemannia
zychae_KAF9904885.1:0.0780771253)/0.947:0.0245656693,(Linnemannia
exigua_KAG0260177.1:0.0484368402,Mortierella sp.
AD032_KAG0380801.1:0.0463956797)/1:0.0618180716)/1:0.0703490573)/1:0.0612969436,
((Mortierella sp. AD031_KAF9085975.1:0.0059006410,Mortierella sp.
GBA35_KAF9095570.1:0.0000028587)/0.128:0.0000024482,Mortierella sp.
NVP41_KAG0202935.1:0.0033559506)/1:0.0949119563)/1:0.2478879158)/1:0.2562310862,
(Actinomortierella wolfii_KAG0227665.1:0.2531051839,Actinomortierella
ambigua_KAG0264648.1:0.2434059985)/1:0.6496484927)/1:0.5367642195,Bifiguratus
adelaidae_OZJ03309.1:1.2628211050)/0.777:0.1271200553)/0.749:0.0618537551,
(((Umbelopsis vinacea_KAG2173566.1:0.1836887749,Umbelopsis
isabellina_KAG2183794.1:0.3911570313)/1:1.0983607051,(Allomyces macrogynus ATCC
38327_KNE68750.1:0.1067492687,Allomyces macrogynus ATCC
38327_KNE69262.1:0.0534757691)/1:1.7831961721)/0.454:0.0899570176,Olpidium
bornovanus_KAG5461318.1:1.0493989900)/1:0.2016591354)/1:0.1074538769)/
0.977:0.1167824569,Cryptococcus neoformans var. grubii
H99_AFR92517.2:2.3910438174)/0.862:0.1007874961);

****
****
MUS81
****
****

(Mucor ambiguus_GAN01801.1:0.0820561653,((((((((((((Rhizophagus
clarus_GES80631.1:0.0636326134,(Rhizophagus irregularis DAOM 181602=DAOM
197198_POG55179.1:0.0129345358,Rhizophagus
diaphanus_RGB32717.1:0.0091169712)/1:0.0519149907)/0.966:0.0212042469,Glomus
cerebriforme_RIA93696.1:0.0897379892)/1:0.3342325729,(((Gigaspora
margarita_KAF0504848.1:0.0000025459,Gigaspora
margarita_KAF0504849.1:0.0000029820)/1:0.0696005615,Gigaspora
rosea_RIB08576.1:0.0314361421)/1:0.2383917289,Diversispora

epigaea_RHZ64173.1:0.3097896157)/1:0.1288842685)/1:0.1866796364,Geosiphon
pyriformis_KAG9305645.1:0.5302226102)/1:0.2177237169,Basidiobolus meristosporus
CBS 931.73_ORY04066.1:0.6666532874)/1:0.1206679662,(Bifiguratus
adelaidae_OZJ01815.1:0.9124740865,((((Jimgerdemannia
flammicorona_RUO95470.1:0.0000026296,Jimgerdemannia
flammicorona_RUO95578.1:0.0348320791)/1:0.5962208990,((Jimgerdemannia
flammicorona_RUP49671.1:0.0620505743,Jimgerdemannia
flammicorona_RUP50707.1:0.0618615267)/1:0.3804156531,Endogone sp. FLAS-
F59071_RUS13225.1:0.8572383522)/0.997:0.1570879112)/1:0.3915869474,
(Jimgerdemannia flammicorona_RUP34718.1:0.0295440090,Jimgerdemannia
flammicorona_RUP45822.1:0.0000029018)/1:1.4888074632)/0.997:0.1844569726,Jimgerd
emannia
flammicorona_RUP47862.1:0.4030492951)/1:0.1480742592)/0.759:0.0781955591)/
0.989:0.1071338601,((((((((Blumeria graminis f. sp.
triticale_CAD6501896.1:0.7997225682,(Neurospora crassa
OR74A_EAA34672.1:0.6165171740,(Trichoderma reesei
QM6a_EGR45943.1:0.3300373480,Verticillium dahliae
VdLs.17_EGY21755.1:0.3090429604)/1:0.1026118041)/0.997:0.0805682003)/
0.993:0.0822979407,(Aspergillus nidulans FGSC
A4_EAA63689.1:0.4102516712,Coccidioides immitis
RS_EAS32427.3:0.3847887688)/1:0.2371504713)/1:0.1439110639,((Sclerotinia
sclerotiorum 1980 UF-70_EDN96658.1:0.7714480639,Exophiala dermatitidis
NIH/UT8656_EHY61186.1:0.6125521487)/0.996:0.0992312417,Bacidia
gigantensis_KAG8525763.1:0.8124364402)/0.918:0.0530759434)/1:0.2195380117,Tuber
melanosporum_CAZ82773.1:0.6988350274)/0.997:0.0955171747,(Orbilia oligospora
ATCC 24927_EGX51410.1:0.2526600516,(Arthrobotrys
entomopaga_KAF3913447.1:0.2911835694,Drechslerella
brochopaga_KAF3921175.1:0.2886835701)/0.72:0.0479379461)/1:0.8692367775)/
1:0.1041070987,(((Schizosaccharomyces
pombe_CAJ77393.1:0.4665601438,Schizosaccharomyces japonicus
yFS275_EEB06018.1:0.4663522515)/1:0.4092205360,Pneumocystis carinii
B80_KTW26178.1:1.0812248342)/0.995:0.1663229928,Neolecta irregularis DAH-
3_OLL23383.1:0.7902438994)/1:0.2002956905)/1:0.1166801373,((((((((Yarrowia
lipolytica CLIB122_CAG82161.1:1.6217275594,(Candida albicans
SC5314_AOW30002.1:0.8074478641,Saccharomyces cerevisiae
S288C_NP_010674.3:1.0477117260)/1:0.3072227051)/0.95:0.1887846897,
(((((((((Encephalitozoon intestinalis ATCC 50506_ADM11132.1:0.3388821291,
(Encephalitozoon hellem ATCC 50504_AFM97886.1:0.1869466881,Encephalitozoon
romaleae
SJ-2008_AFN82605.1:0.2151323822)/0.994:0.1181273451)/1:0.2663319881,Ordospora
colligata OC4_KHN70297.1:0.7374225807)/1:0.4441177471,(((Nosema bombycis
CQ1_EOB13799.1:0.0189382264,Nosema bombycis
CQ1_EOB13810.1:0.0165448429)/1:0.3406900713,Nosema
granulosis_KAF9760984.1:0.3265322760)/1:0.3619983164,(Nosema apis BRL
01_EQB62257.1:0.5623513668,Nosema
ceranae_KKO75551.1:0.3789480037)/1:0.2259629578)/1:0.2424112227)/1:0.2718974828,
Edhazardia aedis USNM 41457_EJW02594.1:0.9545100562)/0.652:0.0260019949,
((Thelohania contejeani_KAF7684173.1:0.6651969508,(Dictyocoela
roeselum_KAG0419137.1:0.3891142833,Dictyocoela
muelleri_KAG0440471.1:0.3077534486)/1:0.8605896995)/0.999:0.1716275429,
(Hamiltosporidium magnivora_TBU00153.1:0.0130527852,Hamiltosporidium
tvaerminnensis_TBU11565.1:0.0318141748)/1:0.8011838508)/1:0.1712060228)/
0.972:0.1318218813,((Vavraia culicis subsp.
floridensis_ELA48438.1:0.3464186349,Trachipleistophora
hominis_ELQ75587.1:0.2392350778)/1:0.4497354347,Pseudoloma
neurophilia_KRH92050.1:0.8346295990)/1:0.8385570983)/0.524:0.0490836709,
(Anncaliia algerae PRA339_KCZ81545.1:0.5471635482,(Tubulinosema
ratisbonensis_RVD91023.1:0.4319330761,Tubulinosema
ratisbonensis_RVD92093.1:0.0668695926)/1:0.3301493041)/1:0.6145849687)/
1:0.2518170848,(Vittaforma corneae ATCC 50505_ELA41963.1:1.1137941401,
((Enterocytozoon hepatopenaei_OQS55808.1:0.9093777613,Enterospora
canceri_ORD93466.1:1.0660945803)/1:0.2004861489,Hepatospora
eriocheir_ORD98110.1:1.1256492937)/0.23:0.0341026191)/1:0.2873712221)/
0.994:0.1468635284,Cucumispora

dikerogammari_KAF7691535.1:1.7582574610)/1:0.2340314406,(((Nematocida parisii
ERTm1_EIJ94922.1:0.0480864002,Nematocida sp.
ERTm5_OAG30574.1:0.0257799802)/1:0.3699876771,Nematocida sp. 1
ERTm6_KFG27116.1:0.4245245400)/1:0.6675230504,Nematocida
displodere_OAG31696.1:0.9931706744)/1:1.4339395068)/1:0.3715565213)/
1:0.1612421042,(Paramicrosporidium saccamoebae_PJF18267.1:1.2575363225,
(Blyttiomyces helicus_RKO94516.1:2.2362465804,((Jimgerdemannia
flammicorona_RUO95367.1:0.0000026911,Jimgerdemannia
flammicorona_RUP47881.1:0.0647855741)/1:1.7524737883,Dictyostelium discoideum
AX4_XP_643164.1:1.3964944918)/0.215:0.0405102347)/0.977:0.1021345161)/
1:0.1935838107)/0.613:0.0864968919,(Rozella allomycis
CSF55_EPZ36724.1:1.1449466149,((((Neocallimastix sp. JGI-
2020a_KAG4098779.1:0.0000024190,Neocallimastix
californiae_ORY30636.1:0.0000025989)/1:0.1700505760,Anaeromyces
robustus_ORX78456.1:0.2124057927)/1:0.1671025642,(Piromyces
finnis_ORX58377.1:0.1755970956,Piromyces sp.
E2_OUM59897.1:0.1246612244)/1:0.1564826854)/0.907:0.0823900785,Neocallimastix
californiae_ORY30635.1:0.1587679804)/1:0.9153479410)/1:0.2116457247)/
0.996:0.1112843141,((Amphiamblys sp. WSBS2006_OIR56202.1:2.0687375619,
(Capsaspora owczarzaki ATCC 30864_XP_004363775.1:1.6399877586,(Fonticula
alba_XP_009493998.1:0.0000032549,Fonticula
alba_XP_009493999.1:0.0000033747)/1:1.3124397384)/0.716:0.0954427698)/
0.978:0.1914907634,((((((sp|Q96NY9|MUS81_HUMAN:0.1202797530,sp|Q91ZJ0|
MUS81_MOUSE:0.1613210203)/1:0.5879520872,Drosophila
melanogaster_NP_569873.1:1.2906720947)/1:0.3942363796,Pomacea
canaliculata_PVD25483.1:0.8480637285)/0.6:0.1050074101,Amphimedon
queenslandica_XP_019864298.1:0.7835522919)/1:0.2085220172,(Monosiga brevicollis
MX1_XP_001742785.1:1.0673967586,(Salpingoeca
rosetta_XP_004989308.1:1.4435038674,(Salpingoeca
rosetta_XP_004992907.1:0.1426580216,Salpingoeca
rosetta_XP_004992909.1:0.0000034160)/1:1.7365675791)/0.994:0.2975506821)/
0.995:0.1467471772)/0.784:0.0534218074,Sphaeroforma arctica
JP610_XP_014159113.1:1.6371565506)/0.995:0.1627631633)/1:0.1223077176)/
1:0.1352962534,(((Batrachochytrium dendrobatidis JAM81_EGF76586.1:1.0072233890,
((Rhizoclosmatium globosum_ORY42827.1:0.4639793450,Chytriomyces
confervae_TPX64868.1:0.4827747358)/1:0.5626107260,Caenorhabditis
elegans_NP_491341.1:2.2636622502)/0.55:0.0707035224)/0.981:0.1106700086,
(((Spizellomyces punctatus DAOM BR117_KND02828.1:0.0256117301,Spizellomyces sp.
'palustris'_TPX64476.1:0.0643342576)/1:0.3774984347,Powellomyces
hirtus_TPX62379.1:0.3506015353)/1:0.6182858196,Blyttiomyces
helicus_RKO92748.1:0.9314786272)/0.914:0.0927001242)/0.983:0.0923575959,
(((Allomyces macrogynus ATCC 38327_KNE56802.1:0.5469314802,Catenaria anguillulae
PL171_ORZ39416.1:0.7323156210)/1:0.5565715893,Caulochytrium
protostelioides_RKO98429.1:1.6918688156)/0.944:0.1496917578,Gonapodya prolifera
JEL478_KXS10082.1:0.9715279530)/1:0.2487001018)/1:0.0897153940)/0.756:0.04500717
17,((Entomophthora muscae_KAF7754229.1:0.7440110693,Conidiobolus coronatus NRRL
28638_KXN72869.1:1.0315773007)/1:0.4406331390,(((((Smittium
mucronatum_OLY84563.1:0.6601469367,(Smittium
simulii_PVU89735.1:0.5339406892,Smittium
megazygosporum_PVV02454.1:0.3509349173)/0.965:0.0776713969)/0.952:0.1035937687,
((Furculomyces boomerangus_PVU89547.1:0.0027703016,Smittium
angustum_PWA00015.1:0.0000026734)/0.957:0.0014332176,Smittium
angustum_PWA02536.1:0.0000027901)/1:0.5925314539)/1:0.1923517506,Zancudomyces
culisetae_OMH83600.1:1.0938985808)/1:0.5228681092,(Linderina
pennispora_ORX66766.1:0.4028215183,Coemansia reversa NRRL
1564_PIA17124.1:0.3312612079)/1:0.6199099608)/1:0.2833189393,Dimargaris
cristalligena_RKP36400.1:1.0271613106)/0.993:0.1633384009)/1:0.1436811724)/
0.989:0.0499661153,(Olpidium bornovanus_KAG5459096.1:0.0355361788,Olpidium
bornovanus_KAG5459283.1:0.0000029531)/1:0.9965496215)/1:0.0733760662,
((((Cryptococcus neoformans var. grubii H99_AFR93423.2:0.8906355357,((((Puccinia
graminis f. sp. tritici CRL 75-36-700-3_EFP74928.2:0.3979874181,Puccinia
graminis f. sp. tritici CRL
75-36-700-3_EFP81072.2:0.0000027358)/1:0.6367931203,Melampsora larici-populina
98AG31_EGF99391.1:0.6759126085)/0.999:0.1200515258,(Melampsora larici-populina

98AG31_EGG11949.1:0.5450533323,Puccinia graminis f. sp. tritici CRL 75-36-700-3_EHS64084.1:0.5987868922)/1:0.2399400254)/1:0.5150130859,Mixia osmundae IAM 14324_KEI40105.1:1.2187521861)/0.998:0.1393299035)/1:0.1058423797,(Dacryopinax primogenitus_EJT98091.1:1.1913607623,(Ramaria rubella_KAF8573882.1:1.0275345442,Rhizoctonia solani_QRW27333.1:0.8270407859)/0.358:0.1447194525)/1:0.2235055844)/0.804:0.0669453759,(Malassezia globosa CBS 7966_EDP42310.1:1.0224127296,(Tilletiaria anomala UBC 951_KDN44739.1:0.7773542095,Ustilago maydis 521_KIS67531.1:0.7590723549)/0.968:0.1555842370)/1:0.2343174233)/1:0.1622041078,((Thamnocephalis sphaerospora_RKP09827.1:0.4090787737,Syncephalis pseudoplumigaleata_RKP23476.1:0.8638109818)/0.99:0.1977463829,Piptocephalis cylindrospora_RKP14562.1:1.4786453719)/1:0.3129743916)/0.987:0.0437659978)/0.999:0.0491577101)/0.843:0.0470534561,(((((((Dissophora ornata_KAF8941018.1:0.3572405076,Gamsiella multidivaricata_KAG0358139.1:0.1717321689)/0.999:0.0261763980,(((Mortierella sp. NVP85_KAF9358882.1:0.3896981619,Mortierella sp. GBA43_KAG0246494.1:0.3480905803)/1:0.1117896466,Modicella reniformis_KAF9984788.1:0.4199235042)/1:0.1231197741,Dissophora globulifera_KAG0321514.1:0.4453877602)/1:0.0730130165)/0.997:0.0133438180,((((Entomortierella lignicola_KAF8977811.1:0.0087779939,Haplosporangium sp. Z 27_KAF9204473.1:0.0227874432)/1:0.1310623219,(Mortierella sp. AM989_KAF9109758.1:0.2297065793,(((Mortierella sp. AD010_KAF9168811.1:0.0000026301,Mortierella sp. AD011_KAF9397645.1:0.0000030403)/1:0.0463202917,Entomortierella chlamydospora_KAG0005093.1:0.0106341780)/1:0.0795954601,Mortierella sp. AD094_KAF9349053.1:0.0553261487)/1:0.1234989045)/1:0.0528705163)/1:0.0386335950,Entomortierella beljakovae_KAF9436387.1:0.4431338265)/1:0.0787331970,Lobosporangium transversale_ORZ16142.1:0.4904590248)/1:0.0304814785)/0.999:0.0208531331,(((Haplosporangium sp. Z 767_KAF9187703.1:0.0012337077,Haplosporangium sp. Z 11_KAF9190671.1:0.0000033183)/1:0.0555154027,Mortierella polycephala_KAG0249375.1:0.0691867882)/1:0.3104378012,(Mortierella antarctica_KAF9991178.1:0.2956109417,Mortierella sp. GBA30_KAG0209919.1:0.3195875096)/1:0.1467859056)/1:0.0945391701)/0.961:0.0219081986,((Lunasporangiospora selenospora_KAF9585190.1:0.3238695860,Jimgerdemannia flammicorona_RUP51772.1:2.8570623838)/0.761:0.2389958295,Gryganskiella cystojenkinii_KAG0057282.1:0.5739865048)/0.997:0.0940044043)/1:0.0480426532,((((Haplosporangium bisporale_KAF8977269.1:0.0683239090,Podila verticillata NRRL 6337_KFH65266.1:0.0000032897)/1:0.1391526180,(Podila horticola_KAF9322379.1:0.0858199883,(Podila clonocystis_KAG0021926.1:0.1126159187,(Podila epicladia_KAG0089454.1:0.0778248597,Podila minutissima_KAG0351226.1:0.0573429604)/1:0.0222025698)/1:0.0156922441)/1:0.0207941731)/1:0.0986852616,Podila humilis_KAG0341131.1:0.3939442103)/1:0.0452587157,Podila epigama_KAF9427549.1:0.4141463642)/1:0.1323699419)/0.989:0.0288717459,(((((Haplosporangium gracile_KAF8945158.1:0.0517643843,Linnemannia schmuckeri_KAF9141753.1:0.0516853799)/1:0.0710487707,(((Mortierella sp. GBA39_KAF9124027.1:0.0284567467,Linnemannia hyalina_KAG9065321.1:0.0166458967)/0.439:0.0059171893,Mortierella hygrophila_KAF9538988.1:0.0305175977)/1:0.0392059899,Linnemannia elongata AG-77_OAQ27994.1:0.0657411173)/1:0.0643718069)/1:0.0675232581,Linnemannia gamsii_KAG0282522.1:0.1615374039)/1:0.1696965326,((Mortierella sp. 14UC_KAF9133335.1:0.0876765732,Linnemannia zychae_KAF9907634.1:0.1230236437)/1:0.1682850799,(Linnemannia exigua_KAG0279007.1:0.0329717148,Mortierella sp. AD032_KAG0372548.1:0.0463547253)/1:0.0443704586)/1:0.0659282859)/0.993:0.0385882979,(Mortierella sp. AD031_KAF9083487.1:0.0027642500,Mortierella sp. GBA35_KAF9107327.1:0.0021784860)/1:0.3527275738)/1:0.1582617265)/1:0.2531456839,(Actinomortierella wolfii_KAG0244055.1:0.2351126532,Actinomortierella ambigua_KAG0253601.1:0.2686820552)/1:0.4223215464)/1:0.6402162795)/1:0.1018167067)/1:0.2411779918,(Umbelopsis isabellina_KAG2175711.1:0.4442434848,Umbelopsis vinacea_KAG2186659.1:0.4574008215)/1:0.4694987307)/1:0.1166492966,((Absidia

glauca_SAL95528.1:0.3201274232,Absidia
repens_ORZ16587.1:0.3367417370)/1:0.1991636101,Hesseltinella
vesiculosa_ORX46032.1:0.6151140709)/1:0.2191166145)/1:0.0625810078,
(((Apophysomyces ossiformis_KAF7720894.1:0.2731833681,(Apophysomyces sp.
BC1015_KAG0175564.1:0.0000025384,Apophysomyces sp.
BC1021_KAG0183365.1:0.0014223819)/1:0.1595797384)/1:0.2504701591,Phycomyces
blakesleeanus NRRL 1555_OAD77591.1:0.4832307731)/1:0.0973860253,(Mucor
circinatus_KAG2224874.1:0.5587853125,Syncephalastrum
racemosum_ORY93132.1:0.5464031748)/1:0.0929361892)/0.781:0.0618766136)/
1:0.2221227358,(Rhizopus delemar RA 99-880_EIE83137.1:0.4070220012,(Rhizopus
microsporus ATCC 52813_PHZ16600.1:0.0252740025,Rhizopus
azygosporus_RCH97419.1:0.0335651147)/1:0.1650549957)/1:0.3634925183)/
1:0.1185345094,Choanephora
cucurbitarum_OBZ86660.1:0.3807291621)/0.958:0.0773048324,(Mucor
saturninus_KAG2197998.1:0.2896035518,Thamnidium
elegans_KAG2228930.1:0.3430958931)/1:0.2107378919)/1:0.2295576715,(Parasitella
parasitica_CEP15304.1:0.1761141803,Mucor
plumbeus_KAG2203997.1:0.1643846970)/0.999:0.0497662574)/1:0.1211449176,Mucor
lusitanicus_KAF1805445.1:0.1016018452);

****
****
REV1
****
****
(Anaeromyces robustus_ORX71674.1:0.3022694368,((Rozella allomycis
CSF55_EPZ33259.1:2.4129110969,((((((Spizellomyces punctatus DAOM
BR117_KNC96285.1:0.0758921585,Spizellomyces sp.
'palustris'_TPX65815.1:0.0717640452)/1:1.4404042211,(Synchytrium
microbalum_TPX32278.1:0.6623692956,Synchytrium
endobioticum_TPX41824.1:0.7838840092)/1:1.1302589965)/1:0.4009653395,
(Chytriomyces confervae_TPX73373.1:1.0600408417,Rhizoclosmatium
globosum_ORY36367.1:0.7803764709)/1:1.1777681370)/1:0.2595567024,Caulochytrium
protostelioides_RKP02661.1:2.4396188990)/1:0.4548762571,((Basidiobolus
meristosporus CBS 931.73_ORY06463.1:1.7804263255,(Syncephalis
pseudoplumigaleata_RKP27377.1:1.0969289642,Thamnocephalis
sphaerospora_RKP06391.1:1.0669553488)/1:1.0740351199)/1:0.3868200549,Paramicrosp
oridium
saccamoebae_PJF17322.1:3.0724352088)/0.993:0.2169514961)/0.944:0.0468388807,
(((((((((((Mucor ambiguus_GAN05676.1:0.1292944457,Mucor
lusitanicus_KAF1805498.1:0.1193289931)/1:0.2207705050,Parasitella
parasitica_CEP18316.1:0.3531493908)/0.876:0.0728925403,Mucor
plumbeus_KAG2215102.1:0.2208427580)/1:0.4477336565,Choanephora
cucurbitarum_OBZ82755.1:0.7137128485)/0.989:0.0958761647,(Mucor
saturninus_KAG2198709.1:0.7306629778,Thamnidium
elegans_KAG2229942.1:0.6722710084)/1:0.1900903843)/1:0.1491992118,(Rhizopus
microsporus ATCC 52813_PHZ11168.1:0.0934422165,(Rhizopus
azygosporus_RCH86140.1:0.0658851092,Rhizopus
azygosporus_RCH97514.1:0.0467512071)/0.997:0.0240410631)/1:0.9148034429)/
1:0.3619788722,((((Absidia glauca_SAM00860.1:0.4636151162,Absidia
repens_ORZ24424.1:0.4956232865)/1:0.3753666068,Hesseltinella
vesiculosa_ORX50861.1:1.1501420901)/1:0.4517469552,((Apophysomyces
ossiformis_KAF7721374.1:0.4605128769,((Apophysomyces sp.
BC1015_KAG0166163.1:0.0000027378,Apophysomyces sp.
BC1034_KAG0186522.1:0.0000021732)/0.126:0.0000020014,Apophysomyces sp.
BC1021_KAG0176158.1:0.0000020514)/1:0.4594726991)/1:0.4536981834,(Circinella
minor_KAG2219697.1:1.1918523158,Syncephalastrum
racemosum_ORZ03555.1:0.8216024693)/1:0.2049755959)/1:0.1642571699)/
0.345:0.0262266025,Phycomyces blakesleeanus NRRL
1555_OAD69587.1:1.6111779794)/0.833:0.0904799047)/1:0.3037885917,(Umbelopsis
vinacea_KAG2179478.1:0.5758632137,Umbelopsis
isabellina_KAG2180052.1:0.6752473341)/1:0.8878234411)/1:0.1729957269,((Olpidium
bornovanus_KAG5460925.1:2.0429552883,(Jimgerdemannia
flammicorona_RUP45435.1:0.3878421470,Endogone sp. FLAS-

F59071_RUS21765.1:0.7539012345)/1:0.4706735873)/0.798:0.1536983957,Bifiguratus adelaidae_OZJ05683.1:1.3375468714)/1:0.2132693514)/1:0.3603645802,((((((((Dissophora ornata_KAF8939859.1:0.4326940932,Gamsiella multidivaricata_KAG0365283.1:0.4765770936)/0.931:0.0556740910,((Mortierella sp. NVP85_KAF9350614.1:0.3094355297,Mortierella sp. GBA43_KAG0245010.1:0.3403244721)/1:0.0947020837,Modicella reniformis_KAF9984948.1:0.3409279408)/1:0.2164400031)/1:0.0531353918,(Dissophora globulifera_KAG0321679.1:0.5283776855,Lobosporangium transversale_ORZ27840.1:0.5542051772)/0.939:0.0665169575)/0.995:0.0617020028,(((Entomortierella lignicola_KAF8979791.1:0.0172593725,Haplosporangium sp. Z 27_KAF9204528.1:0.0450335972)/1:0.3781932448,(Mortierella sp. AM989_KAF9113153.1:0.3320444551,(((Mortierella sp. AD010_KAF9173499.1:0.0152714297,Mortierella sp. AD011_KAF9401055.1:0.0000025567)/1:0.0516918226,Entomortierella chlamydospora_KAG0000760.1:0.0376761575)/1:0.1117794205,Mortierella sp. AD094_KAF9356858.1:0.1085011633)/1:0.1785260600)/1:0.0720184420)/1:0.0927627564,Entomortierella beljakovae_KAF9434802.1:0.6029379451)/1:0.2136034418)/1:0.1414149903,((((((Haplosporangium gracile_KAF8946703.1:0.0546146315,Linnemannia schmuckeri_KAF9147615.1:0.0495578351)/0.983:0.0373498654,Linnemannia gamsii_KAG0284319.1:0.1917743067)/0.912:0.0191901678,((Mortierella sp. GBA39_KAF9142110.1:0.0275330661,(Mortierella hygrophila_KAF9539314.1:0.0280395522,Linnemannia hyalina_KAG9060906.1:0.0397631464)/1:0.0070865854)/1:0.0461046936,Linnemannia elongata AG-77_OAQ22958.1:0.0795787359)/1:0.0571747415)/1:0.0397375276,Linnemannia gamsii_KAG0284318.1:0.1606193334)/1:0.0655651848,((Mortierella sp. 14UC_KAF9125676.1:0.0795262305,Linnemannia zychae_KAF9900957.1:0.1459886237)/1:0.0543750050,(Linnemannia exigua_KAG0272814.1:0.0866923319,Mortierella sp. AD032_KAG0371091.1:0.0756173502)/1:0.0687807315)/1:0.1100513054)/1:0.1313695984,((Mortierella sp. AD031_KAF9088704.1:0.0032462648,Mortierella sp. GBA35_KAF9094211.1:0.0024516803)/0.578:0.0007146886,Mortierella sp. NVP41_KAG0206260.1:0.0009067594)/1:0.1677435100)/1:0.4805954401)/0.414:0.0295702943,(((Haplosporangium sp. Z 11_KAF9188156.1:0.0007879405,Haplosporangium sp. Z 767_KAF9188244.1:0.0000026415)/1:0.0755511865,Mortierella polycephala_KAG0257811.1:0.1016218533)/1:0.4817072705,(Mortierella antarctica_KAF9989297.1:0.3907159127,Mortierella sp. GBA30_KAG0212720.1:0.3020770343)/1:0.1785683836)/1:0.1049503823)/1:0.1293531500,Gryganskiella cystojenkinii_KAG0045659.1:0.6834474418)/1:0.1569627100,((((Haplosporangium bisporale_KAF8953500.1:0.0017664255,Podila verticillata NRRL 6337_KFH69672.1:0.0008870254)/1:0.1505292582,(Podila horticola_KAF9311417.1:0.1047483839,(Podila clonocystis_KAG0021977.1:0.1011461684,Podila minutissima_KAG0352655.1:0.0898295374)/0.987:0.0158689597)/1:0.0692669522)/1:0.1951817273,Podila humilis_KAG0340126.1:0.4631771047)/1:0.1075788237,Podila epigama_KAF9425206.1:0.5171085440)/1:0.3572595122)/0.996:0.1355390671,Lunasporangiospora selenospora_KAF9581565.1:1.0526606500)/1:0.2565562160,(Actinomortierella wolfii_KAG0229916.1:0.3766611943,Actinomortierella ambigua_KAG0256333.1:0.3022063300)/1:0.5576958461)/1:1.1224001542)/1:0.2055318907,((((Schizosaccharomyces pombe_CAA22130.2:1.2442798064,Schizosaccharomyces japonicus yFS275_EEB08738.1:1.2991228355)/1:1.2402054569,((Candida albicans SC5314_AOW29329.1:2.0168437836,NP_014991.1:2.5505278908)/1:0.7732174481,Yarrowia lipolytica CLIB122_CAG78000.1:2.3397240551)/1:0.3890159287)/0.995:0.1879303873,((((((Neurospora crassa OR74A_EAA34773.1:0.7059597558,(Trichoderma reesei QM6a_EGR51527.1:0.6426209229,Verticillium dahliae VdLs.17_EGY22729.1:0.5216185889)/1:0.1496455885)/1:0.1883109629,(Blumeria graminis f. sp. triticale_CAD6504149.1:1.2573380698,Sclerotinia sclerotiorum 1980 UF-70_EDN96373.1:0.3919721805)/1:0.2485893879)/1:0.0947269578,(((Aspergillus nidulans FGSC A4_EAA59218.1:0.6780631622,Coccidioides immitis RS_EAS35739.3:0.4793844513)/1:0.2318745825,Exophiala dermatitidis NIH/UT8656_EHY59900.1:0.6979009486)/0.865:0.0844694652,(Zymoseptoria tritici IPO323_EGP86877.1:0.6808972072,Bacidia

gigantensis_KAG8532688.1:0.5697856427)/1:0.1555110414)/0.997:0.0923801931)/
1:0.5525280841,(Orbilia oligospora ATCC 24927_EGX48330.1:0.1770511049,
(Drechslerella brochopaga_KAF3905979.1:0.2570588897,Arthrobotrys
entomopaga_KAF3928173.1:0.2375480671)/0.981:0.0777992703)/1:1.0568709711)/
1:0.2093812011,Neolecta irregularis
DAH-3_OLL22619.1:1.4836026201)/0.998:0.1666028599,Tuber
melanosporum_CAZ83226.1:1.7186583304)/1:0.2478194171)/1:0.4150245650,
((((Cryptococcus neoformans var. grubii H99_AFR92314.2:1.8226681627,
(((((Coprinopsis cinerea okayama7#130_EAU81859.1:0.6141866963,Amanita muscaria
Koide BX008_KIL68526.1:1.3612379871)/0.998:0.1698679395,Serpula lacrymans var.
lacrymans S7.9_EGO21833.1:0.6092111542)/1:0.3601202918,Ramaria
rubella_KAF8579478.1:0.8987587601)/1:0.1684801387,Rhizoctonia
solani_QRW26746.1:1.6664445475)/1:0.1563856288,Dacryopinax
primogenitus_EJU05804.1:0.8815305712)/1:0.3865796825)/0.978:0.1008871270,
((Puccinia graminis f. sp. tritici CRL 75-36-700-
3_EFP81154.2:0.9209415386,Melampsora larici-populina
98AG31_EGG07252.1:0.8633579544)/1:1.0053302245,Mixia osmundae IAM
14324_KEI39728.1:1.8509563356)/1:0.4494395064)/1:0.1560314396,(Malassezia
globosa CBS 7966_EDP45583.1:2.2232589865,Tilletiaria anomala UBC
951_KDN48842.1:1.4747901346)/1:0.7585427144)/0.681:0.1411193379,Wallemia
ichthyophaga
EXF-994_EOR04140.1:2.7014556767)/1:0.2984004332)/1:0.2146175351)/0.912:0.0769087
459,((Linderina pennispora_ORX73924.1:0.8946340453,Coemansia reversa NRRL
1564_PIA13782.1:0.9976652761)/1:1.2046019488,Dimargaris
cristalligena_RKP37076.1:2.1815058938)/1:0.7445258784)/0.994:0.0981875120,
(((Allomyces macrogynus ATCC 38327_KNE68836.1:0.0199290166,Allomyces macrogynus
ATCC 38327_KNE69354.1:0.3398295703)/1:1.2487835512,Catenaria anguillulae
PL171_ORZ38105.1:1.4994420926)/1:1.0821758519,Gonapodya prolifera
JEL478_KXS11055.1:1.9895254284)/0.988:0.2693553996)/1:0.1401065073)/
1:0.3207800269,(Drosophila melanogaster_NP_612047.1:2.6054933968,(Homo
sapiens_NP_057400.1:0.0955735649,Mus
musculus_NP_062516.2:0.1297669997)/1:1.3304002755)/1:0.7646489882)/
1:0.5868702696)/1:1.7950513872,(Piromyces
finnis_ORX58569.1:0.3489755500,Piromyces sp.
E2_OUM69657.1:0.3634781899)/1:0.1848766339)/1:0.1515028722,(Neocallimastix sp.
JGI-2020a_KAG4094995.1:0.0000023965,Neocallimastix
californiae_ORY27023.1:0.0000025056)/1:0.4922308799);

****
****
REV3
****
****
(Mortierella antarctica_KAF9982575.1:0.4711426731,((((Gamsiella
multidivaricata_KAG0363797.1:0.2665078327,(Mortierella sp.
AD010_KAF9173245.1:0.1075986625,Mortierella sp.
AD094_KAF9358962.1:0.0515631393)/1:0.2345775062)/0.394:0.0223419545,Modicella
reniformis_KAF9956249.1:0.3705135318)/1:0.1103617620,Haplosporangium sp. Z
11_KAF9185070.1:0.3617009764)/1:0.0556564309,(Gryganskiella
cystojenkinii_KAG0052062.1:0.4924728199,Linnemannia
exigua_KAG0255800.1:0.3144646893)/1:0.0838691968)/0.864:0.0262598264,
(((((((((((Rhizopus azygosporus_RCH93904.1:0.0558300704,((Rhizopus
azygosporus_RCH98670.1:0.0438296663,Rhizopus
azygosporus_RCH93903.1:0.0454508548)/1:0.0377561006,Rhizopus microsporus ATCC
52813_PHZ08955.1:0.0421684500)/0.525:0.0167002671)/0.528:0.0068422281,Rhizopus
azygosporus_RCH98669.1:0.0360721815)/1:0.4607671304,Rhizopus delemar RA 99-
880_EIE76811.1:0.3096096499)/1:0.2697140047,((((Mucor
ambiguus_GAN09365.1:0.1977626257,Mucor
lusitanicus_KAF1799869.1:0.0347250081)/1:0.0823058244,(Parasitella
parasitica_CEP18773.1:0.2576918663,Mucor
plumbeus_KAG2194480.1:0.1572895401)/0.999:0.0520442992)/1:0.2565646360,Choanepho
ra cucurbitarum_OBZ91491.1:0.7231455469)/1:0.1284404736,(Thamnidium
elegans_KAG2233310.1:0.4075200704,Mucor
saturninus_KAG2199822.1:0.5744791428)/1:0.1485512084)/1:0.1856822034)/

1:0.3263832331,Phycomyces blakesleeanus NRRL
1555_OAD74983.1:1.0858210013)/0.98:0.0720980533,(Apophysomyces
ossiformis_KAF7730556.1:0.4665494556,(Apophysomyces sp.
BC1015_KAG0163504.1:0.0000028687,Apophysomyces sp.
BC1021_KAG0168869.1:0.0000020600)/1:0.3386845264)/1:0.5202002701)/
0.773:0.0485182336,(Hesseltinella vesiculosa_ORX46984.1:0.8056045661,(Absidia
glauca_SAM02587.1:0.4044178505,Absidia
repens_ORZ16246.1:0.3509892912)/1:0.4544729928)/1:0.2652493363)/0.666:0.05435449
28,(Syncephalastrum racemosum_ORY95936.1:1.0029869727,(Syncephalastrum
racemosum_ORY95935.1:0.7743589204,Mucor
circinatus_KAG2226275.1:0.6793179618)/0.893:0.1198994853)/1:0.1495128049)/
1:0.2940230691,(Umbelopsis isabellina_KAG2172880.1:0.3732147735,Umbelopsis
vinacea_KAG2174660.1:0.3051600745)/1:0.8884562182)/1:0.2996650194,(Bifiguratus
adelaidae_OZJ05850.1:0.9834042679,Endogone sp. FLAS-
F59071_RUS18701.1:1.2243114140)/1:0.2357734459)/1:0.2010772679,
((((((Neocallimastix californiae_ORY22294.1:0.0000026069,Neocallimastix sp. JGI-
2020a_KAG4087626.1:0.0029141919)/1:0.1920061843,(Anaeromyces
robustus_ORX83805.1:0.2733217891,Piromyces
finnis_ORX46867.1:0.3307958115)/0.985:0.0621029189)/1:2.5101991475,((Gonapodya
prolifera JEL478_KXS22183.1:1.3718253897,(Caulochytrium
protostelioides_RKP00368.1:2.2223951004,((Rhizoclosmatium
globosum_ORY46572.1:0.6037966213,(Chytriomyces
confervae_TPX68633.1:0.4808211388,Rhizoclosmatium
globosum_ORY46574.1:0.7431434349)/1:0.6541332900)/1:0.5600850425,Synchytrium
microbalum_TPX33312.1:1.7190623722)/0.995:0.1417701005)/0.944:0.0705402180)/
0.976:0.0722697337,(((Spizellomyces punctatus DAOM
BR117_KNC96642.1:0.0423012682,Spizellomyces sp.
'palustris'_TPX57882.1:0.0794580975)/1:0.6056594740,Powellomyces
hirtus_TPX57984.1:0.8800764654)/1:0.3313937666,Blyttiomyces
helicus_RKO94213.1:1.2578997792)/1:0.2314548420)/1:0.1342068803)/1:0.1382729957,
((((((Dictyocoela muelleri_KAG0436441.1:1.4176800772,Cucumispora
dikerogammari_KAF7701628.1:2.6944053780)/1:0.5494843837,(((Gigaspora
margarita_KAF0378306.1:0.5201460585,Yarrowia lipolytica
CLIB122_CAG83802.1:0.8157970690)/0.707:0.1469591632,Mixia osmundae IAM
14324_KEI36868.1:0.6283423558)/1:0.2426364682,Mitosporidium
daphniae_KGG53117.1:0.9191573079)/1:0.9869087727)/1:1.3787080022,Gonapodya
prolifera JEL478_KXS19990.1:3.5618934318)/1:2.6977151029,Caenorhabditis
elegans_NP_001299902.1:4.1188700718)/1:0.7777052875,((Pomacea
canaliculata_PVD28374.1:1.9200902336,(Homo sapiens_NP_002903.3:0.0679263338,Mus
musculus_NP_035394.2:0.0881108975)/1:0.8799368945)/0.963:0.2189804321,Drosophila
melanogaster_NP_524881.2:1.6360043741)/1:0.2278965586)/1:0.2783301468,Dictyostel
ium discoideum AX4_XP_645553.1:2.0203269322)/1:0.2191216297,Rozella allomycis
CSF55_EPZ34123.1:2.8811508368)/1:0.2127239332)/1:0.0700116637,((((Linderina
pennispora_ORX73789.1:0.9946458521,Coemansia reversa NRRL
1564_PIA14409.1:1.2953491503)/1:0.8133752603,Piptocephalis
cylindrospora_RKP15488.1:3.0114755829)/1:0.5371536576,((Syncephalis
pseudoplumigaleata_RKP26760.1:1.6510279109,Thamnocephalis
sphaerospora_RKP04713.1:1.0103906851)/1:1.6661974042,((Allomyces macrogynus ATCC
38327_KNE68733.1:0.3298104138,Allomyces macrogynus ATCC
38327_KNE69242.1:0.1017604059)/1:0.8970672369,Catenaria anguillulae
PL171_ORZ34448.1:1.3653523988)/1:0.9298697764)/0.911:0.1354386626)/
1:0.1963087387,(Dimargaris cristalligena_RKP36804.1:1.8687825609,Basidiobolus
meristosporus CBS
931.73_ORX94592.1:1.3332845109)/0.999:0.2368172152)/1:0.1442779656)/
1:0.0721252558,((((Mixia osmundae IAM 14324_KEI40821.1:1.0978547727,(Puccinia
graminis f. sp. tritici CRL 75-36-700-3_EFP77731.2:0.5600251500,Melampsora
larici-populina 98AG31_EGG11103.1:0.5290162519)/1:0.5892456282)/1:0.4456599229,
((Tilletiaria anomala UBC 951_KDN41161.1:1.0057074579,Ustilago maydis
521_KIS70779.1:0.8527657592)/1:0.3369108725,Malassezia globosa CBS
7966_EDP45105.1:2.4258972946)/1:0.2746828209)/0.995:0.1146891936,((((((Serpula
lacrymans var. lacrymans S7.9_EGO24942.1:0.6776177001,(Coprinopsis cinerea
okayama7#130_EFI28614.1:0.9446051815,Amanita muscaria Koide
BX008_KIL70820.1:0.9347503053)/1:0.1801588789)/1:0.1530235752,Ramaria
rubella_KAF8581637.1:0.9410531274)/1:0.2017148064,Rhizoctonia

solani_QRW19011.1:1.2646434533)/1:0.1944374915,Dacryopinax
primogenitus_EJU02050.1:1.1649250100)/1:0.1723900224,Wallemia ichthyophaga EXF-994_EOR00295.1:1.9126509265)/0.987:0.1199707047,Cryptococcus neoformans var. grubii H99_AFR93380.1:1.4706458448)/1:0.1847049150)/1:0.1486809431,
((((((((Neurospora crassa OR74A_EAA34999.2:0.7255726693,Trichoderma reesei QM6a_EGR49874.1:0.7727114889)/0.902:0.0925402189,Verticillium dahliae VdLs.17_EGY20914.1:0.5794569571)/1:0.2218167002,(Blumeria graminis f. sp. triticale_CAD6498914.1:0.8030039930,Sclerotinia sclerotiorum 1980 UF-70_EDO01534.1:0.6494952268)/0.999:0.0869155045)/1:0.1620726649,(((Aspergillus nidulans FGSC A4_EAA60359.1:0.7578105657,Coccidioides immitis RS_EAS35045.3:0.5719905344)/1:0.1798554039,Exophiala dermatitidis NIH/UT8656_EHY60912.1:0.5657047096)/1:0.0795736845,Bacidia gigantensis_KAG8534241.1:0.6382800231)/1:0.0804269729)/1:0.0844114703,Zymoseptoria tritici IPO323_EGP82687.1:0.7952510905)/1:0.2060655137,(((Drechslerella brochopaga_KAF3912680.1:0.2561225080,Arthrobotrys entomopaga_KAF3917252.1:0.1622674081)/1:0.1020331148,Orbilia oligospora ATCC 24927_EGX48925.1:0.2588660203)/1:0.5807737165,Tuber melanosporum_CAZ85646.1:1.2500806351)/0.722:0.1063169462)/1:0.1654896010,Neolecta irregularis DAH-3_OLL22088.1:0.9207177154)/1:0.2881965930,(Schizosaccharomyces japonicus yFS275_EEB05155.1:1.5922697477,Schizosaccharomyces pombe_CAB90776.1:1.1991546231)/1:1.3803095058)/0.978:0.0890324467,
((Saccharomyces cerevisiae S288C_NP_015158.1:1.6525413045,Candida albicans SC5314_AOW28616.1:2.0287210195)/1:0.6596271423,Yarrowia lipolytica CLIB122_CAG82421.1:2.1405397987)/1:0.2894073201)/1:0.2231961613)/1:0.2488401027)/1:0.0874574216)/1:0.8414088833,Actinomortierella ambigua_KAG0270384.1:0.7304307660)/1:0.2964761524,Podila horticola_KAF9315501.1:0.4211434630)/1:0.0836085034);

****
SLX1
****

(GAN09719.1:0.0813034220,((((((((((GES96768.1:0.1153170034,
(POG63706.1:0.0024700577,RGB36546.1:0.0122549933)/1:0.0963416846)/1:0.1850420744,RIA84080.1:0.1614939270)/1:0.6792789064,(RHZ75883.1:0.5080617590,
(RIB05573.1:0.8235931711,(RIB12050.1:0.0000028529,RIB17064.1:0.3291996186)/0.474:0.0887503028)/1:0.6980887677)/1:0.3883014743)/1:0.3709467419,
(KAG9292215.1:0.6599141416,KAG9295049.1:0.7085066467)/1:0.5064418315)/0.994:0.2036851340,(((KAF7724511.1:0.0082277948,KAF7750116.1:0.2447216409)/1:1.0882844584,RKP23975.1:0.8631656697)/0.971:0.1808715831,RKP14455.1:1.2153727044)/0.764:0.1727047782)/0.448:0.0719870318,(((((((CAB76036.1:1.2663063973,EEB06434.1:0.9747599818)/1:0.6043646971,((((AFR92528.1:1.0469877858,
(EFP85032.2:0.3844848085,EGF98452.1:0.4383742001)/1:0.6058038774)/0.996:0.2087310461,((((EDP44616.1:0.9715984317,KIS70699.1:0.8178779908)/0.899:0.1809633705,KDN42998.1:0.7205388948)/1:0.4874734005,KEI39923.1:0.7567430461)/0.994:0.1457723307,KAG5461815.1:1.8744054223)/0.763:0.0859751150)/0.955:0.0730500963,((((EFI28179.1:1.3809531761,KIL68887.1:0.3992315998)/0.97:0.3955002602,(EJU03443.1:0.5520255693,KAF8574179.1:0.3941084159)/0.615:0.1108735697)/0.909:0.2073036753,QRW25920.1:0.7966284640)/0.999:0.2770706853,EJU06070.1:1.1677807571)/1:0.2539362714)/0.989:0.0776083916,
(KTW29774.1:1.6589475557,OLL24711.1:0.9024706321)/1:0.3682101219)/0.935:0.0608492860)/0.664:0.0570971249,
((((((CAD6500382.1:0.7480125015,EDN96549.1:1.3968040970)/0:0.0000020397,
((EAA32366.1:0.6466063937,EGY18974.1:0.3088817488)/0.967:0.0968585989,EGR49607.1:0.4176841294)/1:0.3152905755)/1:0.2080343361,
((CAZ84758.1:0.9340426407,KAG8529778.1:0.6130796878)/0.855:0.1590781529,EGP84857.1:0.7334622160)/0.37:0.1129500288)/0.971:0.0768591436,EHY54180.1:0.6717331789)/1:0.2378909897,
(EAA58781.1:0.8196651093,KJF60596.1:0.8104831833)/1:0.2651129196)/1:0.3382076945,(((CAG78504.1:1.0236464474,AOW29469.1:1.1564776544)/0.981:0.1694930650,NP_009787.3:1.1562468350)/

0.989:0.3102865593,KXN72777.1:2.3395404528)/0.964:0.2021702388)/
1:0.2208964177)/1:0.1673078352,((((sp|Q9BQ83|SLX1_HUMAN:0.1852438782,sp|Q8BX32|
SLX1_MOUSE:0.2195200528)/1:0.6040420328,
(((NP_491541.1:1.3850129635,PVD32960.1:0.8521077766)/
0.892:0.1487707663,XP_011406479.2:1.0868184657)/
0.937:0.0861168741,NP_649484.3:1.1225137288)/0.863:0.1465104720)/1:0.4381865184,
(XP_001746382.1:1.4804448800,XP_004997826.1:0.9862322296)/1:0.3580855494)/
1:0.1590626504,(XP_004364864.2:1.4385969571,XP_014147819.1:1.7872676996)/
0.208:0.0133542454)/1:0.2594351826)/1:0.0972716172,
((((((KAF9027572.1:0.0024965118,KFH62914.1:0.0000020260)/1:0.1497225522,
((KAF9310176.1:0.4082771639,
(KAG0091750.1:0.0551745492,KAG0353714.1:0.0445009772)/0.998:0.0326809970)/
0.424:0.0075836287,KAG0026258.1:0.1006161506)/0.993:0.0551400556)/
1:0.2369387972,KAF9414159.1:0.5502129457)/1:0.2919589729,
(((((KAF9168652.1:0.0000026053,KAF9390914.1:0.0075395744)/
0.998:0.1580694730,KAF9352100.1:0.3631712003)/
0.539:0.1347578460,KAG0325667.1:0.7461199523)/0.994:0.3076408130,
(KAG0351763.1:0.4601966382,ORZ25006.1:0.5312883384)/0.858:0.0957996268)/
1:0.1329752492,KAG0263810.1:0.5135009428)/0.575:0.0438031636,
(KAG0057020.1:0.6903765969,KAG0201661.1:0.5163985440)/0.994:0.1658397577)/
0.964:0.1000265742)/0.895:0.1010634260,
((KAF9097706.1:0.0113407244,KAF9104087.1:0.0074790386)/1:0.2486620041,
(((KAF9129091.1:0.0273861762,OAQ35658.1:0.1223158902)/
0.949:0.0117112870,KAF9545210.1:0.0313770749)/
0.711:0.0044829362,KAG9062469.1:0.0481708132)/1:0.2592551109)/1:0.2896800114)/
1:0.2398887238,(KAG0245446.1:0.3288378398,KAG0261396.1:0.0856564961)/
1:0.6860490242)/1:0.4075510442,(KNE70914.1:1.0544686328,
(KXS17726.1:1.0608442172,ORZ29526.1:0.2443683053)/0.885:0.2603874320)/
1:0.5026818212)/0.855:0.1043523274)/0.987:0.0585456172,
(((EGF81182.1:1.2084162793,(ORY47709.1:0.6510792127,TPX77456.1:0.8905775437)/
1:0.3476873919)/0.98:0.2523635910,
(((((KND03795.1:0.0126302234,TPX63374.1:0.1310718538)/
1:0.3004285800,TPX62571.1:0.5113593087)/
1:0.2907082543,RKO93358.1:0.2146716844)/
1:0.4663616136,RKP02590.1:1.0703120171)/0.425:0.0971290814,
(TPX30494.1:0.2786269852,TPX43560.1:0.7011905239)/1:0.6026201512)/
0.999:0.2110580772)/0.964:0.0936746423,
(ORX97842.1:0.5028428702,RKP08226.1:1.4828575418)/0.995:0.4480589498)/
0.999:0.1082752862)/0.771:0.0515331869,
(RUP46083.1:0.5795821862,RUS18099.1:1.0489762869)/1:0.5659453210)/
0.993:0.1162065662)/0.823:0.1109685953,
(((OMH83243.1:1.1149553818,PVU84770.1:0.5919268431)/0.995:0.3833933497,
(PVU98113.1:0.8742649483,PVV01961.1:0.9819694937)/0.976:0.1568531637)/
0.99:0.1821474780,(ORX70031.1:0.5982557732,PIA17820.1:0.5297012736)/
1:0.9075600600)/1:0.3986257148)/1:0.4158726007,
((SAM04371.1:0.3662171701,ORZ25253.1:0.3601667453)/
1:0.6449844740,OAD77899.1:0.7471119513)/0.918:0.1835034185)/1:0.4999674455,
(KAG2208950.1:0.3919481407,KAG2231602.1:0.3501284900)/1:0.2693015883)/
1:0.2334622797,OBZ88379.1:0.3881427393)/
1:0.3298127988,KAG2202958.1:0.1361476629)/
1:0.1462942620,KAF1796128.1:0.0711135525);

****
****
SLX4
****
****
(Mucor ambiguus_GAN04294.1:0.1569219428,((((((((((((((((((Blumeria graminis
f. sp. triticale_CAD6505290.1:1.5734674487,Sclerotinia sclerotiorum 1980 UF-
70_EDN95873.1:0.7361491244)/0.993:0.2133733541,(Neurospora crassa
OR74A_EAA35600.1:1.1259694855,Verticillium dahliae
VdLs.17_EGY15391.1:1.0882570539)/0.999:0.2124498219)/0.998:0.1507835112,
(Zymoseptoria tritici IPO323_EGP90807.1:1.7318022788,Bacidia
gigantensis_KAG8533660.1:1.3647550905)/0.782:0.1386905345)/0.996:0.1610106599,

(Coccidioides immitis RS_EAS33523.3:1.3002675857,Exophiala dermatitidis NIH/UT8656_EHY56462.1:1.3469458108)/1:0.3824742578)/1:0.2055555325,Tuber melanosporum_CAZ80784.1:1.9499417126)/0.842:0.1233587270,((Orbilia oligospora ATCC 24927_EGX44355.1:0.5555967646,Drechslerella brochopaga_KAF3920030.1:0.6541990829)/0.759:0.0831156432,Arthrobotrys entomopaga_KAF3907573.1:0.5395590606)/1:1.4057408785)/0.979:0.2643534979,Pneumocystis carinii B80_KTW26793.1:2.0260268657)/0.702:0.1069631576,
(Schizosaccharomyces japonicus yFS275_EEB05160.1:2.2074718811,Neolecta irregularis DAH-3_OLL23519.1:1.6053040046)/0.954:0.5417923425)/1:0.8111247092,
((((Homo sapiens_NP_115820.2:0.3828348737,Mus musculus_NP_803423.2:0.2030864661)/1:1.5103817486,Pomacea canaliculata_PVD35253.1:2.3063818916)/0.895:0.2487532330,Amphimedon queenslandica_XP_019855666.1:2.0354099565)/0.662:0.0965292792,Drosophila melanogaster_AAF50581.2:2.7756184674)/0.978:0.1621520735)/1:0.1670728505,
(((Entomophthora muscae_KAF7754158.1:2.1673128509,Smittium culicis_OMJ15165.1:2.0450587309)/0.773:0.3672273262,Coemansia reversa NRRL 1564_PIA18173.1:2.1455832141)/0.889:0.2557465692,(Rhizoclosmatium globosum_ORY52012.1:1.7586139112,Dimargaris cristalligena_RKP37522.1:1.5678479642)/0.941:0.4084296169)/0.999:0.2939707516)/0.363:0.0503678043,((((Gigaspora margarita_KAF0491320.1:0.8647976025,Diversispora epigaea_RHZ82587.1:1.2479500238)/0.998:0.4897592901,((Rhizophagus irregularis DAOM 181602=DAOM 197198_POG65663.1:0.0000027588,Rhizophagus diaphanus_RGB34353.1:0.1093648197)/0.915:0.0963964101,Glomus cerebriforme_RIA99142.1:0.2131534947)/1:1.6942154923)/0.977:0.4364267346,Geosiphon pyriformis_KAG9292699.1:2.6666449661)/0.918:0.2212148117,
(((((((Haplosporangium gracile_KAF8947257.1:0.0590011393,Linnemannia schmuckeri_KAF9153323.1:0.0680086578)/1:0.0778855515,((Mortierella sp. GBA39_KAF9137634.1:0.0324797710,(Mortierella hygrophila_KAF9548067.1:0.0324611214,Linnemannia hyalina_KAG9063081.1:0.0252416460)/1:0.0079856710)/1:0.0408952984,Linnemannia elongata
AG-77_OAQ34005.1:0.0958157536)/1:0.0338060480)/1:0.0287524457,Linnemannia gamsii_KAG0294069.1:0.1848295652)/1:0.1003343980,((Mortierella sp. 14UC_KAF9121241.1:0.1114536417,Linnemannia zychae_KAF9909362.1:0.1913884233)/1:0.0499824844,(Linnemannia exigua_KAG0278966.1:0.1076133497,Mortierella sp. AD032_KAG0378424.1:0.0679839876)/1:0.0750975101)/1:0.1303832335)/1:0.0904707313,
((Mortierella sp. GBA35_KAF9095704.1:0.0039800094,Mortierella sp. NVP41_KAG0201404.1:0.0068359596)/0.814:0.0009956494,Mortierella sp. AD031_KAF9096764.1:0.0088760269)/1:0.2329234602)/1:0.4646077915,
((((((Entomortierella lignicola_KAF8976673.1:0.0254532616,Haplosporangium sp. Z 27_KAF9206730.1:0.0141425021)/1:0.3806204852,(((Mortierella sp. AD010_KAF9168479.1:0.0000025436,Mortierella sp. AD011_KAF9377709.1:0.0000024083)/1:0.0471843538,Entomortierella chlamydospora_KAF9999566.1:0.0288373507)/1:0.0879903684,Mortierella sp. AD094_KAF9361940.1:0.0781483520)/1:0.1511395466)/0.865:0.0439220730,Mortierella sp. AM989_KAF9097382.1:0.3204380773)/1:0.3613532226,(((Mortierella sp. NVP85_KAF9363396.1:0.3536055104,Mortierella sp. GBA43_KAG0229259.1:0.5927784649)/1:0.1542361903,Modicella reniformis_KAF9948424.1:0.3916021982)/1:0.2683320903,Lobosporangium transversale_ORY95999.1:0.8210699171)/0.618:0.0620361704)/1:0.1394010164,
(Mortierella antarctica_KAF9984667.1:0.4444665531,Mortierella sp. GBA30_KAG0201841.1:0.3506059209)/1:0.2769691393)/0.658:0.0531037153,
((Haplosporangium sp. Z 767_KAF9188832.1:0.0005325622,Haplosporangium sp. Z 11_KAF9195676.1:0.0000028451)/1:0.0684007503,Mortierella polycephala_KAG0249887.1:0.0910378057)/1:0.4779995209)/1:0.1257842984)/1:0.1367606079,Lunasporangiospora selenospora_KAF9585207.1:1.0405536121)/0.601:0.0442941306,(((((Haplosporangium bisporale_KAF8951561.1:0.0013691958,Podila verticillata NRRL 6337_KFH68771.1:0.0029927049)/1:0.1182821376,((Podila horticola_KAF9318518.1:0.0902947679,Podila clonocystis_KAG0032118.1:0.1140808823)/1:0.0226569017,(Podila epicladia_KAG0099950.1:0.1026660398,Podila

minutissima_KAG0344103.1:0.0460185512)/1:0.0351485871)/1:0.0442262442)/1:0.2711917982,Podila humilis_KAG0343589.1:0.7258588992)/0.864:0.0583638030,Podila epigama_KAF9429871.1:1.2820812063)/0.562:0.0484125579,Podila epigama_KAF9429872.1:0.5703112698)/1:0.2767307388)/1:0.8825738525)/1:0.3499726514)/1:0.1960315109,((((Gigaspora margarita_KAF0489281.1:0.7602851880,Diversispora epigaea_RHZ59877.1:0.9708159876)/1:0.4172031742,((Rhizophagus irregularis DAOM 181602=DAOM 197198_POG76120.1:0.0099063435,Rhizophagus diaphanus_RGB42146.1:0.0211638502)/0.972:0.0873899210,Glomus cerebriforme_RIA92597.1:0.2464518245)/1:0.9511984927)/1:0.8461183781,Jimgerdemannia flammicorona_ RUS34494.1:2.7068887342)/0.611:0.1660815053,Bifiguratus adelaidae_OZJ03080.1:1.6046968327)/1:0.3679913651)/0.699:0.0917732755,(Spizellomyces punctatus DAOM BR117_KND02277.1:2.5063199451,(Blyttiomyces helicus_RKO92700.1:1.2978536939,(Synchytrium microbalum_TPX36927.1:0.9931873309,Synchytrium endobioticum_TPX41112.1:0.8657990135)/1:0.9727374089)/0.698:0.1933483021)/0.947:0.2268303098)/0.962:0.1790011839,((Allomyces macrogynus ATCC 38327_KNE55792.1:0.1086605412,Allomyces macrogynus ATCC 38327_KNE58596.1:0.0980284591)/1:1.3004616684,Catenaria anguillulae PL171_ORZ39386.1:1.4266655535)/1:1.3504504712)/0.548:0.1110289498,Endogone sp. FLAS-F59071_RUS14531.1:1.9303563027)/0.993:0.3575178347,Umbelopsis isabellina_KAG2184687.1:2.2144295639)/1:0.4945353067,((((Absidia glauca_SAM04252.1:0.4586630749,Absidia repens_ORZ18742.1:0.5972352702)/1:0.2892385833,((Hesseltinella vesiculosa_ORX49061.1:0.0365650776,Hesseltinella vesiculosa_ORX61335.1:0.0415015113)/0.756:0.0308039872,(Hesseltinella vesiculosa_ORX50905.1:0.1759407673,(Hesseltinella vesiculosa_ORX50971.1:0.0942399634,Hesseltinella vesiculosa_ORX50980.1:0.0791810005)/1:0.0682098643)/0.809:0.0336161702)/1:0.8008186275)/1:0.6164955462,Mucor circinatus_KAG2227093.1:1.0126484174)/0.998:0.1892818866,((Apophysomyces ossiformis_KAF7730591.1:0.4128518187,Apophysomyces sp. BC1015_KAG0165650.1:0.2760190589)/1:0.7789152170,Phycomyces blakesleeanus NRRL 1555_OAD75020.1:1.2104739158)/0.739:0.1468795686)/0.996:0.1857081804)/1:0.3492299180,(Mucor saturninus_KAG2196205.1:1.8350140624,(Rhizopus microsporus ATCC 52813_PHZ17549.1:0.0807979156,Rhizopus azygosporus_RCH87559.1:0.0282747151)/1:0.8530340013)/0.339:0.0980553906)/1:0.3319674866,Choanephora cucurbitarum_OBZ83460.1:0.9156484685)/1:0.3337972257,(Apophysomyces ossiformis_KAF7726568.1:0.2792556314,(Apophysomyces sp. BC1015_KAG0166577.1:0.0184338476,Apophysomyces sp. BC1034_KAG0185166.1:0.0017012286)/1:0.3720740762)/1:0.9437513200)/1:0.2332661045,(Parasitella parasitica_CEP16274.1:0.3787637293,Mucor plumbeus_KAG2211796.1:0.2300833449)/0.982:0.0867800424)/1:0.1486429429,Mucor lusitanicus_KAF1800200.1:0.1308063559);

****
UAF1
****

(Mucor ambiguus_GAN06004.1:0.0457633223,((((((((((Rhizophagus clarus_GES99091.1:0.0941927559,(Rhizophagus irregularis DAOM 181602=DAOM 197198_POG77238.1:0.0110815803,Rhizophagus diaphanus_RGB41683.1:0.0078547888)/1:0.0574393397)/1:0.0887249584,Glomus cerebriforme_RIA86949.1:0.1046298214)/1:0.3650710578,(((Gigaspora margarita_KAF0482057.1:0.0480777330,Gigaspora rosea_RIB12846.1:0.0073462694)/1:0.4907506514,Diversispora epigaea_RHZ70594.1:0.5087496554)/1:0.0736553179,Geosiphon pyriformis_KAG9300435.1:0.8112527220)/0.862:0.0725353350)/1:0.5243505483,(((((((((Schizosaccharomyces pombe_CAA90472.1:1.2063550228,Schizosaccharomyces japonicus yFS275_EEB05778.1:1.1453798697)/1:1.1484446452,Pneumocystis carinii B80_KTW26648.1:2.4198029609)/0.994:0.2606453707,((((((Blumeria graminis f. sp. triticale_CAD6502618.1:1.1794583076,Sclerotinia sclerotiorum 1980 UF-

70_EDN93717.1:0.4152190215)/0.994:0.0903847646,(Neurospora crassa
OR74A_EAA26970.3:0.4274865879,(Trichoderma reesei
QM6a_EGR46090.1:0.5207541562,Verticillium dahliae
VdLs.17_EGY17563.1:0.4071575986)/0.996:0.0820820074)/1:0.2675347857)/
1:0.2705609481,Bacidia
gigantensis_KAG8527502.1:0.8868820829)/0.936:0.0790289497,(Aspergillus nidulans
FGSC A4_EAA65293.1:0.6489068429,Coccidioides immitis
RS_KJF61341.1:0.8088057412)/1:0.3926399042)/0.999:0.0952677280,(Zymoseptoria
tritici IPO323_EGP84705.1:0.9326550167,Exophiala dermatitidis
NIH/UT8656_EHY53142.1:0.7094598902)/0.989:0.1072026468)/1:0.2249961932,(Tuber
melanosporum_CAZ82349.1:0.4985229299,Orbilia oligospora ATCC
24927_EGX53497.1:0.8093756308)/1:0.2295100844)/1:0.4136118483,Neolecta
irregularis DAH-3_OLL22332.1:2.5020934326)/0.962:0.1720277499)/1:0.2151292263,
(((Yarrowia lipolytica CLIB122_CAG80673.1:2.1629688202,Candida albicans
SC5314_AOW26898.1:3.4416909668)/0.994:0.4596065978,((((Smittium
mucronatum_OLY80951.1:0.5791369349,(Smittium
culicis_OMJ18871.1:0.0000021227,Smittium
culicis_OMJ21102.1:0.0000026865)/1:0.4795558125)/1:0.5372873926,((Smittium
simulii_PVU95148.1:0.8293168421,Smittium
megazygosporum_PVV05014.1:0.8570554552)/1:0.4947353846,(Furculomyces
boomerangus_PVU98469.1:0.0023331861,Smittium
angustum_PVZ99499.1:0.0000022226)/1:0.9612632430)/0.992:0.1754903357)/
1:0.4071922809,(Zancudomyces culisetae_OMH81623.1:0.0020643695,Zancudomyces
culisetae_OMH82256.1:0.0000029008)/1:1.8363706636)/1:1.1619132690,Coemansia
reversa NRRL
1564_PIA13383.1:1.6918458537)/1:0.5243698273)/0.643:0.2067604143,Mortierella sp.
AD011_KAF9402550.1:4.3078022530)/1:0.3166709547)/0.997:0.1795943639,
(((Cryptococcus neoformans var. grubii H99_AFR98517.1:1.7203596504,Wallemia
ichthyophaga EXF-994_EOR02105.1:2.0634703677)/1:0.3093634514,((Puccinia graminis
f. sp. tritici CRL 75-36-700-3_EFP89517.2:0.8173776939,Melampsora larici-
populina 98AG31_EGG07426.1:0.9027595458)/1:0.6055754749,Mixia osmundae IAM
14324_KEI41626.1:1.4980458086)/0.996:0.1922450908)/0.397:0.0596004706,
((((((Coprinopsis cinerea okayama7#130_EAU81628.2:0.7301376374,Serpula lacrymans
var. lacrymans S7.9_EGO22940.1:0.5076946764)/1:0.1491306827,Postia placenta Mad-
698-R_EED79602.1:0.6130294046)/0.995:0.1422186203,Amanita muscaria Koide
BX008_KIL59657.1:1.0027465931)/0.976:0.1298798949,Ramaria
rubella_KAF8585272.1:1.0779913313)/1:0.2228800457,Rhizoctonia
solani_QRW21057.1:1.5483247339)/1:0.3479645812,(Malassezia globosa CBS
7966_EDP44715.1:3.4967131874,(Tilletiaria anomala UBC
951_KDN40106.1:1.2613952165,Ustilago maydis
521_KIS66105.1:1.0266055665)/1:0.2729889475)/1:0.2757392521)/0.999:0.0970979441)
/1:0.5133010588)/1:0.1569096026,(((Rozella allomycis
CSF55_EPZ32897.1:4.8888803641,((((Homo
sapiens_sp_Q8TAF3_WDR48_HUMAN:0.0000026925,Mus
musculus_sp_Q8BH57_WDR48_MOUSE:0.0229426968)/1:0.3443335625,(Drosophila
melanogaster_NP_725018.1:1.3671938404,Pomacea
canaliculata_XP_025091365.1:0.4452317010)/0.967:0.2058075291)/1:0.4480248112,Amp
himedon
queenslandica_XP_019855207.1:2.0611057663)/0.986:0.3472300174,Caenorhabditis
elegans_NP_001129837.1:1.8009960095)/1:1.3861680743)/1:0.9204119529,
((((((Neocallimastix sp. JGI-2020a_KAG4086350.1:0.0000026291,Neocallimastix
californiae_ORY21314.1:0.0064300917)/1:0.2894432424,Neocallimastix
californiae_ORY29177.1:0.2078264943)/1:0.1396911489,Anaeromyces
robustus_ORX86193.1:0.2722468399)/0.993:0.0804810745,(Piromyces
finnis_ORX45759.1:0.2093932548,Piromyces sp.
E2_OUM65273.1:0.1488429314)/0.994:0.0509410718)/1:2.0048913127,((((Spizellomyces
punctatus DAOM BR117_KNC97696.1:0.0386918427,Spizellomyces sp.
'palustris'_TPX70229.1:0.0345028186)/1:0.7274260451,Powellomyces
hirtus_TPX57410.1:1.3167467142)/1:0.3905791883,(Rhizoclosmatium
globosum_ORY38779.1:0.5675188288,(Chytriomyces
confervae_TPX65605.1:0.0162272398,Chytriomyces
confervae_TPX65703.1:0.0196885467)/1:0.5043349162)/1:1.1802506481)/
1:0.1498711499,(Synchytrium microbalum_TPX32848.1:0.3910477743,Synchytrium
endobioticum_TPX42846.1:0.4074002472)/1:1.0698030401)/1:0.1596067172)/

1:0.1719756719,(Gonapodya prolifera JEL478_KXS19961.1:1.5564775946,Caulochytrium protostelioides_RKO99040.1:2.6632319708)/0.968:0.2411124591)/1:0.2221259936)/1:0.2063780202,((Entomophthora muscae_KAF7740114.1:2.5036035484,Conidiobolus coronatus NRRL 28638_KXN74841.1:1.3189502513)/1:0.6219033674,((Allomyces macrogynus ATCC 38327_KNE54926.1:0.0572060996,Allomyces macrogynus ATCC 38327_KNE56578.1:0.0582478747)/1:0.9123260504,Catenaria anguillulae PL171_ORZ39446.1:0.9461378525)/1:1.4585019724)/1:0.2520031073)/0.931:0.0633890790)/1:0.1446075987,((Thamnocephalis sphaerospora_RKP05196.1:0.6463760681,Syncephalis pseudoplumigaleata_RKP25812.1:1.1768982088)/1:0.5785226753,Piptocephalis cylindrospora_RKP12039.1:2.1850765714)/1:0.5974852809)/0.943:0.1191921974,((((((((Dissophora ornata_KAF8930569.1:0.1993643218,Dissophora globulifera_KAG0330129.1:0.2176861390)/1:0.0556877115,Gamsiella multidivaricata_KAG0364651.1:0.2121071595)/0.83:0.0200361460,(((((Entomortierella lignicola_KAF8982692.1:0.0016158733,Haplosporangium sp. Z 27_KAF9202058.1:0.0187929732)/1:0.1629875506,(Mortierella sp. AD094_KAF9357749.1:0.0507074120,(Mortierella sp. AD011_KAF9401314.1:0.0472964114,Entomortierella chlamydospora_KAG0000173.1:0.0469263060)/1:0.0890675570)/1:0.0627634240)/0.768:0.0217105773,Mortierella sp. AM989_KAF9115155.1:0.1411418107)/1:0.0468548756,Entomortierella beljakovae_KAF9435757.1:0.2645004267)/1:0.0972752053,Lobosporangium transversale_ORZ16192.1:0.3129451850)/1:0.0309982413)/1:0.0270091881,(Mortierella sp. NVP85_KAF9353785.1:0.2161781079,Mortierella sp. GBA43_KAG0232601.1:0.2840525878)/1:0.1960558863)/1:0.0466941855,((Haplosporangium sp. Z 11_KAF9195553.1:0.0539095592,Mortierella polycephala_KAG0265759.1:0.0547487629)/1:0.2303149950,(Mortierella antarctica_KAF9989775.1:0.1758563177,Mortierella sp. GBA30_KAG0208294.1:0.1846627495)/1:0.1182999289)/1:0.0528571202)/1:0.0518461087,(((Mortierella sp. AD031_KAF9087953.1:0.0010181532,Mortierella sp. NVP41_KAG0216357.1:0.0000026658)/0.999:0.0037254686,Mortierella sp. GBA35_KAF9097274.1:0.0055638185)/1:0.0879756662,(((Mortierella sp. 14UC_KAF9122597.1:0.0681894623,Linnemannia zychae_KAF9909186.1:0.0599816408)/1:0.0128850701,(Linnemannia exigua_KAG0276724.1:0.0412221208,Mortierella sp. AD032_KAG0375145.1:0.0288256118)/1:0.0279113302)/1:0.0374869989,((((Mortierella sp. GBA39_KAF9142973.1:0.0156160398,(Mortierella hygrophila_KAF9541159.1:0.0203550898,Linnemannia hyalina_KAG9069447.1:0.0143902267)/1:0.0076288561)/1:0.0242442739,Linnemannia elongata AG-77_OAQ32512.1:0.0373952205)/1:0.0178231026,Linnemannia schmuckeri_KAF9152661.1:0.0465797381)/1:0.0137122224,Linnemannia gamsii_KAG0297416.1:0.1192307217)/1:0.0271312690)/0.999:0.0184047261)/1:0.2628509150)/1:0.0499866397,(((((Haplosporangium bisporale_KAF9021358.1:0.0000021267,Podila verticillata NRRL 6337_KFH71405.1:0.0011113145)/1:0.0524994301,(Podila horticola_KAF9319555.1:0.0361473068,(Podila clonocystis_KAG0011749.1:0.0356419430,Podila minutissima_KAG0362124.1:0.0350071961)/0.517:0.0064833557)/1:0.0304916913)/1:0.0853676043,Podila humilis_KAG0340983.1:0.2097687404)/0.996:0.0406540687,Podila epigama_KAF9425093.1:0.2592883236)/1:0.1857470618,Gryganskiella cystojenkinii_KAG0055558.1:0.3323769367)/1:0.0466896788)/1:0.0681204254,Lunasporangiospora selenospora_KAF9583033.1:0.3426310993)/1:0.2266539332,(Actinomortierella wolfii_KAG0234464.1:0.1864835468,Actinomortierella ambigua_KAG0255251.1:0.1969374351)/1:0.3857211202)/1:0.7789694404)/1:0.1438661019,Basidiobolus meristosporus CBS 931.73_ORX90173.1:1.2082992583)/0.967:0.0695716672,Dimargaris cristalligena_RKP36173.1:2.1643742916)/0.702:0.0685416872)/1:0.2857102560,Bifiguratus adelaidae_OZJ05112.1:1.1818239739)/1:0.3665718844,(Umbelopsis vinacea_KAG2178276.1:0.2446963282,Umbelopsis isabellina_KAG2185698.1:0.2361582333)/1:0.5800526990)/1:0.3747703084,(Mucor circinatus_KAG2224003.1:0.5711818080,Syncephalastrum racemosum_ORY94461.1:0.7479161710)/1:0.2160273218)/1:0.1201788977,(((((Absidia glauca_SAM06382.1:0.1571438987,Absidia

repens_ORZ23372.1:0.2031832797)/0.996:0.0624507831,Absidia
repens_ORZ23544.1:0.3352202693)/1:0.2200381795,Hesseltinella
vesiculosa_ORX58265.1:0.6682769848)/1:0.3119409874,Phycomyces blakesleeanus NRRL
1555_OAD78892.1:0.7586904928)/0.997:0.1278488888,(Apophysomyces
ossiformis_KAF7731707.1:0.1817635790,(Apophysomyces sp.
BC1015_KAG0167708.1:0.0000026670,(Apophysomyces sp.
BC1021_KAG0176969.1:0.0000028453,Apophysomyces sp.
BC1034_KAG0187838.1:0.0000021054)/0.682:0.0015878791)/1:0.1250324580)/
1:0.3946386118)/0.746:0.0790548713)/1:0.3085452025,(Rhizopus delemar RA 99-
880_EIE87749.1:0.2453653395,((Rhizopus microsporus ATCC
52813_PHZ08927.1:0.0100170741,Rhizopus
azygosporus_RCH83628.1:0.0237244063)/0.957:0.0135234484,Rhizopus
azygosporus_RCH89467.1:0.0120726701)/1:0.2117379658)/1:0.2653244141)/
0.977:0.0649890481,Choanephora
cucurbitarum_OBZ91909.1:0.4261693594)/1:0.0936603677,(Mucor
saturninus_KAG2197154.1:0.1429678215,Thamnidium
elegans_KAG2236102.1:0.1620892674)/1:0.1506168493)/1:0.1923992500,(Parasitella
parasitica_CEP07720.1:0.1889665290,Mucor
plumbeus_KAG2198169.1:0.0564855283)/0.956:0.0142684996)/1:0.0327197627,Mucor
lusitanicus_KAF1796190.1:0.0195863189);

****
UBE2T
****
(Homo sapiens_NP_054895.1:0.2816607454,Mus musculus_NP_080300.1:0.1596770745,
(((((Geosiphon pyriformis_KAG9289972.1:1.2102170850,((((Rhizophagus
clarus_GES99406.1:0.0835636582,Glomus
cerebriforme_RIA99482.1:0.2101268623)/0.671:0.0536257690,(Rhizophagus
irregularis DAOM 181602=DAOM 197198_POG77416.1:0.0117755506,Rhizophagus
diaphanus_RGB27378.1:0.0247020954)/1:0.0727871603)/1:0.5748579999,(Gigaspora
margarita_KAF0445981.1:0.1309931697,Gigaspora
rosea_RIB04901.1:0.1025604196)/1:0.3266927058)/0.933:0.3011523883,Diversispora
epigaea_RHZ75676.1:0.8165108515)/0.953:0.3567239955)/1:0.9032206477,
(((((Lobosporangium transversale_ORZ24902.1:0.7698606842,(((Entomortierella
lignicola_KAF8977123.1:0.8460536225,(((Mortierella sp.
AD010_KAF9162854.1:0.0000034023,Mortierella sp.
AD011_KAF9388804.1:0.0000030125)/1:0.0823457443,Entomortierella
chlamydospora_KAF9995070.1:0.0414616449)/1:0.0969249657,Mortierella sp.
AD094_KAF9346149.1:0.1293334890)/1:0.2064407087)/1:0.1579106310,Mortierella sp.
AM989_KAF9111692.1:0.5793301614)/1:0.2488068142,Entomortierella
beljakovae_KAF9438090.1:1.3341299183)/0.665:0.1100196497)/0.388:0.0837199440,
((((((((Linnemannia elongata AG-77_OAQ28995.1:0.0284027419,(Mortierella sp.
GBA39_KAF9146431.1:0.0426656696,(Mortierella
hygrophila_KAF9549655.1:0.0423081610,Linnemannia
hyalina_KAG9062529.1:0.0371838460)/0.996:0.0136530300)/0:0.0000035363)/
1:0.1402099433,(Haplosporangium gracile_KAF8932398.1:0.0826136508,Linnemannia
schmuckeri_KAF9137329.1:0.1015408663)/1:0.0824205541)/1:0.0638711173,Linnemannia
gamsii_KAG0288676.1:0.2632304347)/1:0.1236223190,((Mortierella sp.
14UC_KAF9124681.1:0.1808243627,Linnemannia
zychae_KAF9910042.1:0.1940414727)/1:0.0627242369,(Linnemannia
exigua_KAG0270930.1:0.1473850473,Mortierella sp.
AD032_KAG0377997.1:0.1087408898)/1:0.1203425766)/1:0.1378391868)/1:0.1421122992,
((Mortierella sp. GBA35_KAF9089956.1:0.1501959524,Mortierella sp.
AD031_KAF9094564.1:0.0015629204)/0.749:0.0014642853,Mortierella sp.
NVP41_KAG0220176.1:0.0000032021)/1:0.2864548681)/1:0.3926322178,(Haplosporangium
bisporale_KAF8987345.1:0.2600836623,((Podila
horticola_KAF9318644.1:0.5371156871,Podila
clonocystis_KAG0014511.1:0.0871161309)/0.291:0.0245419739,(Podila
epicladia_KAG0098404.1:0.0594087488,Podila
minutissima_KAG0359321.1:0.0883277161)/0.999:0.1728212674)/1:0.4798251704)/
1:1.4851250531)/0.997:0.3158363388,((Haplosporangium sp. Z
767_KAF9193720.1:0.0057836592,Haplosporangium sp. Z

11_KAF9194423.1:0.0022833077)/0.981:0.0871436459,Mortierella
polycephala_KAG0260589.1:0.1255645553)/1:1.1963308799)/1:0.2249249849,
(Dissophora ornata_KAF8931003.1:0.9481132106,((((Mortierella sp.
NVP85_KAF9363092.1:0.8415096473,Mortierella sp.
GBA43_KAG0217820.1:0.6662715846)/0.968:0.1174110703,Modicella
reniformis_KAF9931513.1:0.6684987450)/1:0.3727447094,Gamsiella
multidivaricata_KAG0369476.1:0.9381117060)/0.559:0.0586231338,Dissophora
globulifera_KAG0320379.1:1.2201990015)/0.805:0.1225018373)/0.93:0.0476086887)/
0.999:0.1599478801)/0.947:0.2468602448,Actinomortierella
wolfii_KAG0239348.1:3.0520253849)/1:0.4871692096,(Dimargaris
cristalligena_RKP33913.1:0.7241464011,Dimargaris
cristalligena_RKP37486.1:0.0290737516)/1:2.2616772593)/0.941:0.1319752966,
((((((((Apophysomyces ossiformis_KAF7725770.1:0.3098981699,Apophysomyces sp.
BC1015_KAG0172427.1:0.5080811237)/1:0.3919708280,Phycomyces blakesleeanus NRRL
1555_OAD75043.1:1.1538535333)/0.994:0.2970780421,((((((Mucor
ambiguus_GAN00685.1:0.1758453553,Mucor
lusitanicus_KAF1807134.1:0.2951518799)/1:0.6185438530,Mucor
plumbeus_KAG2191613.1:0.2086668820)/0.933:0.2357330389,Parasitella
parasitica_CEP16216.1:0.2811350238)/1:0.6372395281,Mucor
saturninus_KAG2193625.1:1.3103588211)/0.554:0.1849257534,(Choanephora
cucurbitarum_OBZ80388.1:0.0000038393,Choanephora
cucurbitarum_OBZ88305.1:0.0099494954)/1:0.7381487154)/0.991:0.2567018487,
((Rhizopus microsporus ATCC 52813_PHZ12538.1:0.0000037738,Rhizopus
azygosporus_RCI00311.1:0.0253026139)/0:0.0000031875,Rhizopus
azygosporus_RCH92618.1:0.0581842786)/1:1.0158327774)/1:0.7462181351)/
0.967:0.4637589787,Syncephalastrum
racemosum_ORZ02420.1:1.7205733756)/0.686:0.2296494523,((Absidia
glauca_SAM08754.1:1.8166236266,Absidia
repens_ORZ22108.1:1.1294306925)/1:0.6389045575,Hesseltinella
vesiculosa_ORX43014.1:2.3364286560)/0.948:0.3062140965)/1:0.1974113444,(Mucor
circinatus_KAG2222156.1:1.5667622520,(Smittium simulii_PVU92793.1:1.7473371757,
(Furculomyces boomerangus_PVU96021.1:0.0007715828,Smittium
angustum_PWA01309.1:0.0042255923)/1:1.0787787009)/1:1.5367624575)/
0.993:0.2944157289)/0.756:0.1231452098,(Conidiobolus coronatus NRRL
28638_KXN69474.1:2.6453812328,Coemansia reversa NRRL
1564_PIA17634.1:3.7628630414)/0.998:1.4891997494)/1:0.3325156511,(Umbelopsis
isabellina_KAG2176276.1:0.6900279091,Umbelopsis
vinacea_KAG2187432.1:0.9840398042)/1:0.7488553653)/1:0.3410045408,Jimgerdemannia
flammicorona_RUP46715.1:1.7887869332)/0.996:0.1609132634)/0.454:0.0475249171)/
0.817:0.0778463993,Basidiobolus meristosporus CBS
931.73_ORX87830.1:1.5848028307)/0.994:0.2100308857,((((Neocallimastix sp. JGI-
2020a_KAG4104728.1:0.0000033910,Neocallimastix
californiae_ORY80140.1:0.2537770160)/1:0.2727688342,Piromyces sp.
E2_OUM59557.1:0.0000038665)/0.989:0.0635617724,Piromyces
finnis_ORX60002.1:0.1185519927)/0.701:0.0902292178,Anaeromyces
robustus_ORX87042.1:0.0184325267)/1:1.7944248566)/0.97:0.2018409459,
(((Entomophthora muscae_KAF7746143.1:0.0213557806,Entomophthora
muscae_KAF7756356.1:0.0150015308)/1:2.9833042964,(Piptocephalis
cylindrospora_RKP15093.1:2.5655720064,Chytriomyces
confervae_TPX73553.1:2.7868824439)/0.809:0.3971589241)/0.878:0.2098399082,
(Gonapodya prolifera JEL478_KXS20353.1:2.6118526468,(Synchytrium
microbalum_TPX30665.1:1.2141824768,Synchytrium
endobioticum_TPX50958.1:1.3705538639)/1:0.9975889502)/0.849:0.3032449512)/
0.985:0.1499199086)/1:1.8731414322);

****
UHRF1
****

(Rhizophagus clarus_GES98839.1:0.0863605463,(((((((((((((((((Tuber
melanosporum_CAZ81145.1:0.7084434768,Neolecta irregularis DAH-
3_OLL24290.1:0.6803002727)/1:0.3363556980,((((Gigaspora
margarita_KAF0492460.1:0.0255509794,Gigaspora

rosea_RIB16757.1:0.0543651706)/1:0.4134530163,((Rhizophagus irregularis DAOM 181602=DAOM 197198_POG66335.1:0.0199666044,Rhizophagus diaphanus_RGB34248.1:0.0000021069)/1:0.1293017704,Glomus cerebriforme_RIA88323.1:0.1516536770)/1:0.3084900332)/0.749:0.0806253278,Diversispora epigaea_RHZ83652.1:0.4850674548)/1:0.2683444500,Geosiphon pyriformis_KAG9302660.1:0.6148644104)/0.957:0.1572600531)/0.996:0.1412853035,((((Cryptococcus neoformans var. grubii H99_AFR92806.2:0.8609865838,Ramaria rubella_KAF8591869.1:0.4760351248)/0.995:0.1290375264,((((Coprinopsis cinerea okayama7#130_EAU93010.2:0.5913585257,(Serpula lacrymans var. lacrymans S7.9_EGO29217.1:0.7143047547,Amanita muscaria Koide BX008_KIL70800.1:0.3417256362)/0.522:0.0830740607)/0.993:0.0822926967,(Postia placenta Mad-698-R_EED77985.1:0.0000021303,Postia placenta Mad-698-R_EED79579.1:0.0229111164)/1:0.6464440382)/0.295:0.0343316398,Rhizoctonia solani_QRW23708.1:0.6177911850)/0.898:0.0742772323,((Serpula lacrymans var. lacrymans S7.9_EGO24569.1:1.9225898836,Amanita muscaria Koide BX008_KIL59367.1:1.7413440267)/0:0.0000023456,Dacryopinax primogenitus_EJU01741.1:0.3940333459)/1:0.3374485855)/0.966:0.0480199376)/0.906:0.0680569868,(Serpula lacrymans var. lacrymans S7.9_EGO24537.1:1.4976735923,Ramaria rubella_KAF8591878.1:0.3074890446)/1:0.4530612205)/1:0.1014459555,(Puccinia graminis f. sp. tritici CRL 75-36-700-3_EFP86608.2:0.7869485093,(Melampsora larici-populina 98AG31_EGG03470.1:0.0053435805,Melampsora larici-populina 98AG31_EGG03472.1:0.0000026777)/1:0.7087674616)/1:0.3403510651)/1:0.0997801028)/0.967:0.0477828874,(Blyttiomyces helicus_RKO87597.1:0.6740422735,Endogone sp. FLAS-F59071_RUS13915.1:0.7433802172)/0.91:0.1233653307)/1:0.1273733452,(((Spizellomyces punctatus DAOM BR117_KNC97706.1:0.0878856574,Spizellomyces sp. 'palustris'_TPX70147.1:0.0914411537)/1:0.3422318632,Powellomyces hirtus_TPX57111.1:0.4438744181)/1:0.2343830152,(Gonapodya prolifera JEL478_KXS15836.1:0.8372660649,Gonapodya prolifera JEL478_KXS15837.1:0.5126939201)/0.993:0.3799674500)/0.999:0.1542466361)/0.97:0.1164688117,(Entomophthora muscae_KAF7745688.1:0.0000030487,Entomophthora muscae_KAF7751697.1:0.0155161588)/1:0.9120618903)/0.953:0.1644829502,((Allomyces macrogynus ATCC 38327_KNE55589.1:0.2034884638,Allomyces macrogynus ATCC 38327_KNE58396.1:0.0933286283)/1:0.4138772858,Catenaria anguillulae PL171_ORZ34731.1:1.1070430276)/1:0.8632985663)/0.993:0.1747440923,Neolecta irregularis DAH-3_OLL23852.1:1.2968550290)/0.989:0.1916096972,(Coprinopsis cinerea okayama7#130_EAU81689.1:1.0686249650,Postia placenta Mad-698-R_EED78905.1:1.0567112080)/1:0.7325722325)/0.97:0.1270950920,((((((Tuber melanosporum_CAZ84611.1:1.2574968322,Bacidia gigantensis_KAG8533905.1:0.8581622558)/1:0.6573155760,Exophiala dermatitidis NIH/UT8656_EHY58608.1:1.4727605224)/0.938:0.2574055071,(Tuber melanosporum_CAZ85865.1:1.0644393149,(Zymoseptoria tritici IPO323_EGP85349.1:1.2361921022,Bacidia gigantensis_KAG8530138.1:1.1097094483)/0.997:0.2984045184)/0.984:0.1296213507)/1:0.3233789608,(Puccinia graminis f. sp. tritici CRL 75-36-700-3_EFP82430.2:1.0699463061,Puccinia graminis f. sp. tritici CRL 75-36-700-3_EFP89919.1:0.9724132486)/1:0.7625746858)/0.422:0.0519772260,(Orbilia oligospora ATCC 24927_EGX50774.1:1.3625753632,Arthrobotrys entomopaga_KAF3914702.1:0.8962017153)/1:1.3674735424)/0.991:0.1070119476,Neolecta irregularis DAH-3_OLL25679.1:1.7249534427)/0.997:0.1068713801)/1:0.3732914555,(((Apophysomyces ossiformis_KAF7722263.1:0.6245262416,Mucor circinatus_KAG2216217.1:0.9513903656)/0.325:0.0686229399,Phycomyces blakesleeanus NRRL 1555_OAD78800.1:0.5223081945)/1:0.6003233195,Endogone sp. FLAS-F59071_RUS16724.1:1.1416217242)/1:0.3236160139)/0.319:0.0523757289,Entomophthora muscae_KAF7748579.1:1.5805326838)/0.985:0.1559208774,(Blyttiomyces helicus_RKO85294.1:0.6845849802,(Homo sapiens_NP_037414.3:0.1484757067,Mus musculus_NP_001104549.1:0.1533040642)/1:0.5279646049)/0.996:0.1470408098)/1:0.1148199946,((((((((Haplosporangium gracile_KAF8942923.1:0.0611638518,Linnemannia schmuckeri_KAF9151706.1:0.0678238582)/1:0.0268093647,(((Mortierella sp. GBA39_KAF9135029.1:0.0668453315,(Mortierella hygrophila_KAF9547005.1:0.0897019608,Linnemannia hyalina_KAG9071309.1:0.0431406361)/1:0.0229022624)/1:0.0965576934,Linnemannia elongata AG-77_OAQ23967.1:0.1788324317)/1:0.1202393124,Linnemannia

gamsii_KAG0280772.1:0.2678959985)/0.99:0.0566251042)/1:0.1634821815,
((Mortierella sp. 14UC_KAF9128393.1:0.0587641091,Linnemannia
zychae_KAF9911712.1:0.2848359947)/1:0.0528725818,(Linnemannia
exigua_KAG0273158.1:0.0832262083,Mortierella sp.
AD032_KAG0377022.1:0.1294322914)/1:0.1788428920)/1:0.1841568550)/1:0.2622012901,
(Mortierella sp. AD031_KAF9081301.1:0.0292457181,(Mortierella sp.
GBA35_KAF9101601.1:0.0048170284,Mortierella sp.
NVP41_KAG0208771.1:0.0000025982)/0.13:0.0000022537)/1:0.5732198304)/
1:0.7920164425,Dissophora globulifera_KAG0327314.1:0.3048546122)/1:0.2862930074,
(((Entomortierella lignicola_KAF8985371.1:0.0367315676,Haplosporangium sp. Z
27_KAF9201264.1:0.0000624719)/1:0.2071941093,Mortierella sp.
AD094_KAF9344554.1:0.2456698429)/1:0.0729991381,Entomortierella
beljakovae_KAF9432920.1:0.2423160967)/1:0.1333239133)/0.997:0.1067039927,
((Haplosporangium bisporale_KAF8953255.1:0.0108981073,Podila verticillata NRRL
6337_KFH69196.1:0.0023634541)/1:0.2323102925,(Podila
horticola_KAF9320230.1:0.0463405458,(Podila
clonocystis_KAG0026230.1:0.0556611131,(Podila
epicladia_KAG0099849.1:0.0228396861,Podila
minutissima_KAG0359199.1:0.0367937087)/1:0.0248227063)/0.861:0.0137169301)/
1:0.1149737142)/1:0.3814793647)/0.999:0.1146114133,Gryganskiella
cystojenkinii_KAG0046603.1:1.0755458759)/0.995:0.1054453431,(Gryganskiella
cystojenkinii_KAG0047352.1:0.8966037718,Gryganskiella
cystojenkinii_KAG0057329.1:0.9556345245)/0.583:0.1193952908)/1:0.1057922856)/
1:0.0690022533,((((((((Parasitella parasitica_CEP18119.1:0.2471220064,Mucor
lusitanicus_KAF1805708.1:0.4445819779)/0.816:0.0258722547,Mucor
plumbeus_KAG2212464.1:0.1887829508)/1:0.2919566116,(((Rhizopus delemar RA 99-
880_EIE89579.1:0.3420320160,((Rhizopus microsporus ATCC
52813_PHZ09449.1:0.0472676048,Rhizopus
azygosporus_RCH81184.1:0.0453004845)/0.977:0.0135681006,Rhizopus
azygosporus_RCH91102.1:0.0000023677)/1:0.2798543557)/1:0.4566767563,Choanephora
cucurbitarum_OBZ88071.1:0.4627763628)/1:0.1066539082,(Mucor
saturninus_KAG2203637.1:0.3607630569,Thamnidium
elegans_KAG2233688.1:0.3699551075)/1:0.1530378350)/1:0.0922917759)/
1:0.4275415820,((Apophysomyces
ossiformis_KAF7729969.1:0.1692835674,Apophysomyces sp.
BC1015_KAG0165534.1:0.1249787990)/1:0.2929114570,Mucor
circinatus_KAG2220699.1:0.4185895989)/1:0.1039470043)/1:0.1966217705,
((((Jimgerdemannia flammicorona_RUO96083.1:0.4701078022,Jimgerdemannia
flammicorona_RUP44194.1:0.0494755850)/0.938:0.1213416168,Jimgerdemannia
flammicorona_RUO96084.1:0.1297031671)/1:0.2454723850,Jimgerdemannia
flammicorona_RUP18993.1:0.5017205343)/0.841:0.0418522002,Endogone sp. FLAS-
F59071_RUS17141.1:0.2209482055)/1:0.3394001320)/0.994:0.1136590165,Catenaria
anguillulae PL171_ORZ37451.1:1.1990759609)/1:0.1173183422,(Gonapodya prolifera
JEL478_KXS19403.1:0.8791390610,Neolecta irregularis DAH-
3_OLL25756.1:1.0290039803)/0.758:0.0513007566)/0.97:0.0285418570,((Linderina
pennispora_ORX67290.1:0.3457534629,(Linderina
pennispora_ORX68382.1:1.0041117298,Linderina
pennispora_ORX72449.1:0.0175460819)/0.984:0.0790344391)/1:0.3494567406,Coemansia
reversa NRRL
1564_PIA18290.1:0.4162351441)/1:0.3600002956)/0.99:0.0343911532)/0.828:0.0555400
201,(((Entomophthora muscae_KAF7752823.1:0.4629115593,((Conidiobolus coronatus
NRRL 28638_KXN73339.1:0.4693992815,Conidiobolus coronatus NRRL
28638_KXN73340.1:0.1273239589)/1:0.3327617187,Conidiobolus coronatus NRRL
28638_KXN73341.1:1.1642676273)/1:0.2706253638)/0.997:0.0978073253,Dimargaris
cristalligena_RKP34926.1:0.5301122167)/0.996:0.0840314629,Basidiobolus
meristosporus CBS
931.73_ORX90637.1:0.4140888022)/1:0.1078789484)/1:0.1501728080,Geosiphon
pyriformis_KAG9289816.1:0.5267435541)/1:0.2069306326,((Gigaspora
margarita_KAF0444535.1:0.0242774516,Gigaspora
rosea_RIB02314.1:0.0191436304)/1:0.3034946283,Diversispora
epigaea_RHZ84872.1:0.3414347827)/0.973:0.0666072699)/1:0.2803232714,Glomus
cerebriforme_RIA81528.1:0.1606205212)/0.814:0.0446267503,(Rhizophagus
irregularis DAOM 181602=DAOM 197198_POG71742.1:0.0106267273,Rhizophagus
diaphanus_RGB27451.1:0.0083179053)/1:0.0421209995);

****
UHRF2
****
(Rhizophagus clarus_GES98839.1:0.0785765479,((((((((((((((((Tuber melanosporum_CAZ81145.1:0.7870709017,Neolecta irregularis DAH-3_OLL24290.1:0.6432992432)/1:0.2908203323,((((Gigaspora margarita_KAF0492460.1:0.0259948960,Gigaspora rosea_RIB16757.1:0.0490826390)/1:0.4481984543,Diversispora epigaea_RHZ83652.1:0.4435655457)/0.796:0.1041948443,((Rhizophagus irregularis DAOM 181602=DAOM 197198_POG66335.1:0.0190261229,Rhizophagus diaphanus_RGB34248.1:0.0000026187)/1:0.1309920997,Glomus cerebriforme_RIA88323.1:0.1482088374)/1:0.2645716928)/1:0.2224683896,Geosiphon pyriformis_KAG9302660.1:0.5732543950)/0.694:0.0655175511)/0.999:0.1203896653,(((Cryptococcus neoformans var. grubii H99_AFR92806.2:0.7017037474,Ramaria rubella_KAF8591869.1:0.4381766758)/0.957:0.1013595995,((((Coprinopsis cinerea okayama7#130_EAU93010.2:0.3300010971,Serpula lacrymans var. lacrymans S7.9_EGO24537.1:1.7990199478)/0.986:0.2517465807,(Serpula lacrymans var. lacrymans S7.9_EGO29217.1:0.7172648759,Amanita muscaria Koide BX008_KIL70800.1:0.3051917197)/0.365:0.0714469562)/0.997:0.0877041408,(Postia placenta Mad-698-R_EED77985.1:0.0000033331,Postia placenta Mad-698-R_EED79579.1:0.0164525672)/1:0.5691493381)/0.996:0.0937729704,((Dacryopinax primogenitus_EJU01741.1:0.4289565986,Ramaria rubella_KAF8591878.1:0.7347465680)/0.988:0.1466163038,Rhizoctonia solani_QRW23708.1:0.5822411520)/0.748:0.1162907707)/0.989:0.0839294646)/1:0.1996151983,(Puccinia graminis f. sp. tritici CRL 75-36-700-3_EFP86608.2:0.7156337352,(Melampsora larici-populina 98AG31_EGG03470.1:0.0049422686,Melampsora larici-populina 98AG31_EGG03472.1:0.0000027550)/1:0.5434066062)/1:0.4524574598)/1:0.0790072833)/0.997:0.0575976655,(Blyttiomyces helicus_RKO87597.1:0.7256325950,Endogone sp. FLAS-F59071_RUS13915.1:0.8231965613)/0.686:0.0781207926)/1:0.1021412796,(((Spizellomyces punctatus DAOM BR117_KNC97706.1:0.0713436579,Spizellomyces sp. 'palustris'_TPX70147.1:0.0992614828)/1:0.3336136640,Powellomyces hirtus_TPX57111.1:0.4563656611)/0.996:0.1651033712,Gonapodya prolifera JEL478_KXS15837.1:0.9942683926)/1:0.1813819968)/0.994:0.1314886157,(((Coprinopsis cinerea okayama7#130_EAU81689.1:0.9162261780,Amanita muscaria Koide BX008_KIL59367.1:0.9975856072)/0.989:0.2738751426,Postia placenta Mad-698-R_EED78905.1:1.0652187671)/1:0.6688459211,(Entomophthora muscae_KAF7745688.1:0.0087824675,Entomophthora muscae_KAF7751697.1:0.0056540048)/1:0.5102342690)/1:0.3246656085)/0.996:0.1573233656,Neolecta irregularis DAH-3_OLL23852.1:1.3811692384)/0.882:0.1124690372,((Allomyces macrogynus ATCC 38327_KNE55589.1:0.1857758858,Allomyces macrogynus ATCC 38327_KNE58396.1:0.0993227437)/1:0.4012773481,Catenaria anguillulae PL171_ORZ34731.1:1.0834576273)/1:0.7435899330)/1:0.3458990154,((((Tuber melanosporum_CAZ85865.1:1.5290519789,(Zymoseptoria tritici IPO323_EGP85349.1:1.4582623253,Bacidia gigantensis_KAG8530138.1:1.2174132225)/0.759:0.1122077357)/1:0.4351201751,((Puccinia graminis f. sp. tritici CRL 75-36-700-3_EFP82430.2:1.0615357603,Puccinia graminis f. sp. tritici CRL 75-36-700-3_EFP89919.1:0.9906519080)/1:0.7297073304,Exophiala dermatitidis NIH/UT8656_EHY58609.1:2.0130518067)/0.783:0.1808275951)/0.918:0.0868721912,(Orbilia oligospora ATCC 24927_EGX50774.1:1.7385468733,Arthrobotrys entomopaga_KAF3914702.1:0.8655819162)/1:1.3656321352)/0.979:0.0807380579,Neolecta irregularis DAH-3_OLL25679.1:1.7554104334)/0.957:0.0458052330)/1:0.3544596505,((((Apophysomyces ossiformis_KAF7722263.1:0.6476582662,Mucor circinatus_KAG2216217.1:0.8606161653)/0.958:0.0977808271,Phycomyces blakesleeanus NRRL 1555_OAD78800.1:0.5161723546)/1:0.4392513490,Endogone sp. FLAS-F59071_RUS16724.1:1.0293839550)/1:0.4973480410,Entomophthora muscae_KAF7748579.1:1.4243513328)/0.946:0.1427886913)/0.965:0.0579448523,(Homo sapiens_NP_690856.1:0.0633234831,Mus musculus_NP_659122.2:0.0359456408)/1:0.7634388700)/1:0.1506886802,

((((((((((Haplosporangium gracile_KAF8942923.1:0.0695235551,Linnemannia
schmuckeri_KAF9151706.1:0.0652034574)/1:0.0527280977,((Mortierella sp.
GBA39_KAF9135029.1:0.0606132469,(Mortierella
hygrophila_KAF9547005.1:0.0832504423,Linnemannia
hyalina_KAG9071309.1:0.0420032216)/1:0.0232351111)/1:0.0870015978,Linnemannia
elongata
AG-77_OAQ23967.1:0.1658235084)/1:0.1256647002)/1:0.0563591627,Linnemannia
gamsii_KAG0280772.1:0.1910875403)/1:0.1618537716,((Mortierella sp.
14UC_KAF9128393.1:0.0589518532,Linnemannia
zychae_KAF9911712.1:0.2585481967)/1:0.0621617307,(Linnemannia
exigua_KAG0273158.1:0.0802815516,Mortierella sp.
AD032_KAG0377022.1:0.1198933896)/1:0.1582111972)/1:0.1558051404)/1:0.1647220266,
((Mortierella sp. AD031_KAF9081301.1:0.0281648033,Mortierella sp.
NVP41_KAG0208771.1:0.0000025096)/0.61:0.0044988284,Mortierella sp.
GBA35_KAF9101601.1:0.0000024536)/1:0.5931571423)/1:0.5739661769,Dissophora
globulifera_KAG0327314.1:0.1824030769)/1:0.3334471674,(((Entomortierella
lignicola_KAF8985371.1:0.0351207997,Haplosporangium sp. Z
27_KAF9201264.1:0.0014837993)/1:0.1989326924,Mortierella sp.
AD094_KAF9344554.1:0.2215054584)/1:0.0753386968,Entomortierella
beljakovae_KAF9432920.1:0.2343273113)/1:0.1257826865)/1:0.1254195975,
(((Haplosporangium bisporale_KAF8952282.1:0.0000024144,Podila verticillata NRRL
6337_KFH69196.1:0.0000033046)/0.502:0.0024295881,Haplosporangium
bisporale_KAF8953255.1:0.0103495633)/1:0.2114130922,(Podila
horticola_KAF9320230.1:0.0468482991,(Podila
clonocystis_KAG0026230.1:0.0547693615,((Podila
epicladia_KAG0099849.1:0.0095368110,Podila
minutissima_KAG0359200.1:0.0257922336)/0.691:0.0118694032,Podila
minutissima_KAG0359199.1:0.0358805286)/1:0.0253806603)/0.87:0.0129995973)/
1:0.1247905925)/1:0.4061777285)/0.984:0.0982931574,Gryganskiella
cystojenkinii_KAG0046603.1:0.9197285369)/1:0.0998409789,Gryganskiella
cystojenkinii_KAG0057329.1:0.9932796994)/0.78:0.0371340693,Gryganskiella
cystojenkinii_KAG0047352.1:0.9378801384)/1:0.1084488989)/0.997:0.0576429084,
((((((((Parasitella parasitica_CEP18119.1:0.2419594203,Mucor
lusitanicus_KAF1805708.1:0.4260977607)/0.993:0.0315976376,Mucor
plumbeus_KAG2212464.1:0.1746743252)/1:0.3504444867,((Mucor
saturninus_KAG2203637.1:0.3574958403,Thamnidium
elegans_KAG2233688.1:0.3408738005)/1:0.1358540892,Choanephora
cucurbitarum_OBZ88071.1:0.4424939741)/0.997:0.0794480368)/0.975:0.0995761441,
(Rhizopus delemar RA 99-880_EIE89579.1:0.3509604346,((Rhizopus microsporus ATCC
52813_PHZ09449.1:0.0467102131,Rhizopus
azygosporus_RCH91102.1:0.0120965473)/0.96:0.0192039418,Rhizopus
azygosporus_RCH81184.1:0.0244911580)/1:0.2437845383)/1:0.3873577941)/
1:0.4083396313,((Apophysomyces
ossiformis_KAF7729969.1:0.1565357765,Apophysomyces sp.
BC1015_KAG0165534.1:0.1251823952)/1:0.2822015338,Mucor
circinatus_KAG2220699.1:0.4075915441)/0.995:0.0801824340)/1:0.1964223115,
(((Gonapodya prolifera JEL478_KXS15836.1:2.2252435649,(((Jimgerdemannia
flammicorona_RUO96083.1:0.4903744410,Jimgerdemannia
flammicorona_RUP44194.1:0.0405135947)/0.287:0.0166217193,Jimgerdemannia
flammicorona_RUP43119.1:0.1323738598)/0.979:0.1157026829,Jimgerdemannia
flammicorona_RUO96084.1:0.1213861875)/0.301:0.0262883707)/0.93:0.2185630431,Jimg
erdemannia flammicorona_RUP18993.1:0.4498620163)/0.82:0.0409472326,Endogone sp.
FLAS-F59071_RUS17141.1:0.2326595287)/1:0.2787240856)/0.998:0.1066775601,Catenari
a anguillulae PL171_ORZ37451.1:1.2190255244)/1:0.1017751627,((Gonapodya
prolifera JEL478_KXS19403.1:0.7764183411,Blyttiomyces
helicus_RKO85294.1:0.7369748010)/0.762:0.0506558052,Neolecta irregularis DAH-
3_OLL25756.1:0.9591126520)/0.992:0.0756831223)/1:0.0677937229,(((Entomophthora
muscae_KAF7752823.1:0.4506449019,(Conidiobolus coronatus NRRL
28638_KXN73339.1:0.4661880709,Conidiobolus coronatus NRRL
28638_KXN73340.1:0.1079192070)/1:0.5233555834)/1:0.1212921859,Dimargaris
cristalligena_RKP34926.1:0.4944986633)/0.995:0.0937425167,((Linderina
pennispora_ORX67290.1:0.3305234636,(Linderina
pennispora_ORX68382.1:0.9128511986,Linderina
pennispora_ORX72449.1:0.0207457124)/0.976:0.0626924715)/1:0.3246701983,Coemansia

reversa NRRL 1564_PIA18290.1:0.4197693928)/1:0.3287083173)/0.714:0.0547462877)/
1:0.0494623290)/1:0.0784263019,Basidiobolus meristosporus CBS
931.73_ORX90637.1:0.4950289673)/1:0.1398397335,Geosiphon
pyriformis_KAG9289816.1:0.5166203236)/1:0.1525594443,((Gigaspora
margarita_KAF0444535.1:0.0224630394,Gigaspora
rosea_RIB02314.1:0.0217710406)/1:0.3003203767,Diversispora
epigaea_RHZ84872.1:0.3201494710)/0.973:0.0548902656)/1:0.2928090974,Glomus
cerebriforme_RIA81528.1:0.1569707515)/0.76:0.0432535224,((Rhizophagus
irregularis DAOM 181602=DAOM 197198_POG67111.1:0.0084385528,Rhizophagus
irregularis DAOM 181602=DAOM
197198_POG71742.1:0.0084023836)/0.959:0.0034146660,Rhizophagus
diaphanus_RGB27451.1:0.0076199442)/1:0.0476176239);

****
****
USP1
****
****
(Saccharomyces cerevisiae S288C_NP_010161.1:1.1485215813,(((((((((((((((Mucor
ambiguus_GAN01631.1:0.1471492913,Mucor
lusitanicus_KAF1797960.1:0.1871433943)/1:0.5970639555,(Parasitella
parasitica_CEP14438.1:0.5464941733,Mucor
plumbeus_KAG2214701.1:0.1383129298)/1:0.1922206576)/1:0.7524934061,(Mucor
saturninus_KAG2213164.1:0.5057068041,Thamnidium
elegans_KAG2228590.1:0.4112314133)/1:0.2220732770)/0.96:0.0958723133,(Rhizopus
delemar RA 99-880_EIE83632.1:0.4106459405,Rhizopus microsporus ATCC
52813_PHZ08735.1:0.6635155952)/1:0.6259958900)/1:0.5534187380,((Absidia
glauca_SAM04751.1:0.4260704906,Absidia
repens_ORZ20776.1:0.6619193841)/1:0.3024593887,Hesseltinella
vesiculosa_ORX62259.1:1.3842547722)/1:0.4085069189)/0.847:0.0865497000,
(((((((((Mucor ambiguus_GAN06813.1:0.0898920889,Mucor
lusitanicus_KAF1804501.1:0.0672150095)/1:0.0568694490,Parasitella
parasitica_CEP17847.1:0.2470766149)/0.522:0.0381568675,Mucor
plumbeus_KAG2210405.1:0.1309578976)/1:0.1798415354,Mucor
saturninus_KAG2196131.1:0.5743342107)/0.821:0.0924252760,Choanephora
cucurbitarum_OBZ83086.1:0.4897089808)/1:0.2228434490,(Rhizopus microsporus ATCC
52813_PHZ07232.1:0.0280431962,Rhizopus
azygosporus_RCH89888.1:0.0341685544)/1:1.0046931563)/1:0.2765649038,(Absidia
glauca_SAL98858.1:0.3031031413,Absidia
repens_ORZ19440.1:0.2935462812)/1:1.2302262709)/0.942:0.1018734113,
((Apophysomyces ossiformis_KAF7730068.1:0.4310167703,(Apophysomyces sp.
BC1015_KAG0171429.1:0.0144959556,Apophysomyces sp.
BC1034_KAG0185918.1:0.0000020620)/1:0.2976783510)/1:0.4099488431,Phycomyces
blakesleeanus NRRL
1555_OAD72915.1:0.9938800757)/0.957:0.1319593889)/1:0.1730358076,((Umbelopsis
isabellina_KAG2173152.1:0.3771462388,Umbelopsis
vinacea_KAG2177466.1:0.3199901841)/1:1.3914802149,(Mucor
circinatus_KAG2227768.1:1.0706101768,Syncephalastrum
racemosum_ORY99230.1:1.0873664185)/1:0.2884000811)/0.992:0.1792315575)/
1:0.1733773635,(((Apophysomyces ossiformis_KAF7728375.1:0.3584508639,
(Apophysomyces sp. BC1015_KAG0170760.1:0.0000023603,Apophysomyces sp.
BC1034_KAG0189271.1:0.0000022657)/1:0.1433982239)/1:0.5747217481,Phycomyces
blakesleeanus NRRL 1555_OAD77169.1:1.2230297626)/1:0.2677102528,(Mucor
circinatus_KAG2228178.1:1.0420489580,Syncephalastrum
racemosum_ORY93955.1:1.0555445349)/1:0.2703579361)/0.867:0.0630674666)/
1:0.1327437889)/1:0.4243206716,(Umbelopsis
vinacea_KAG2179530.1:0.3974027983,Umbelopsis
isabellina_KAG2185789.1:0.3138367878)/1:1.1899567192)/0.979:0.2559001891,Bifigur
atus adelaidae_OZJ03583.1:2.1994572462)/1:0.3690430745,((((((Entomophthora
muscae_KAF7743069.1:0.0000027798,Entomophthora
muscae_KAF7755267.1:0.0027031483)/0.838:0.0081209424,Entomophthora
muscae_KAF7748306.1:0.0000029904)/1:0.7025629332,Conidiobolus coronatus NRRL
28638_KXN71862.1:1.1042755756)/1:1.0061604034,(Coemansia reversa NRRL
1564_PIA17609.1:1.6730640966,Dimargaris

cristalligena_RKP38129.1:1.5004009308)/1:0.5336344956)/0.838:0.1343863633,Basidi
obolus meristosporus CBS 931.73_ORX93242.1:1.3864117468)/0.971:0.1720258086,
(((((((((Entomortierella lignicola_KAF8976287.1:0.0157044626,Haplosporangium
sp. Z 27_KAF9201495.1:0.0082752387)/1:0.1486920329,(Mortierella sp.
AM989_KAF9112928.1:0.1473970745,(((Mortierella sp.
AD010_KAF9175145.1:0.0000029545,Mortierella sp.
AD011_KAF9402340.1:0.0000029986)/1:0.0559162180,Entomortierella
chlamydospora_KAF9996191.1:0.0321447029)/1:0.0704538756,Mortierella sp.
AD094_KAF9357022.1:0.0320912215)/1:0.0591990423)/0.945:0.0136153324)/
1:0.0588563210,Entomortierella
beljakovae_KAF9435848.1:0.3170267380)/1:0.1026861955,Lobosporangium
transversale_ORZ16798.1:0.4712056467)/1:0.0492165785,((Mortierella sp.
NVP85_KAF9366537.1:0.9098116864,Dissophora
globulifera_KAG0314198.1:0.3847281534)/0.999:0.0898432480,Gamsiella
multidivaricata_KAG0363121.1:0.2330104578)/1:0.1018333012)/1:0.0875080627,
(((Haplosporangium sp. Z 767_KAF9182178.1:0.0000022627,Haplosporangium sp. Z
11_KAF9191791.1:0.0023840266)/1:0.0490671446,Mortierella
polycephala_KAG0262432.1:0.0726043123)/1:0.2435429225,Gryganskiella
cystojenkinii_KAG0045201.1:0.4728025529)/1:0.0495254464)/0.791:0.0384586073,
(Mortierella antarctica_KAF9989904.1:0.2854220975,Mortierella sp.
GBA30_KAG0197613.1:0.2933377935)/1:0.1714225037)/1:0.0921869993,((Mortierella
sp. AD031_KAF9095038.1:0.0000020546,Mortierella sp.
NVP41_KAG0212553.1:0.0000020090)/1:0.0991139093,((Mortierella sp.
14UC_KAF9130564.1:0.0636753441,Linnemannia
zychae_KAF9912637.1:0.0607488647)/1:0.0815431390,((Mortierella sp.
GBA39_KAF9137860.1:0.0386405128,Linnemannia elongata AG-
77_OAQ29707.1:0.0317789143)/1:0.0329289863,Linnemannia
gamsii_KAG0284045.1:0.0893996933)/1:0.0456615327)/1:0.0626420451)/
1:0.2577390288)/0.999:0.1350827455,(((Haplosporangium
bisporale_KAF8981935.1:0.0000021761,Podila verticillata NRRL
6337_KFH72528.1:0.0016896613)/1:0.0604521543,((Podila
horticola_KAF9321665.1:0.0418704295,(Podila
epicladia_KAG0092016.1:0.0364615593,Podila
minutissima_KAG0356769.1:0.0285568722)/1:0.0236127952)/0.67:0.0033747472,Podila
clonocystis_KAG0013664.1:0.0556855526)/1:0.0308503175)/1:0.1687901846,Podila
epigama_KAF9428932.1:0.3897302536)/1:0.5850131010)/1:0.3541424989,Actinomortiere
lla wolfii_KAG0228194.1:0.8809525695)/1:0.8029047998)/0.988:0.1838988241,
((Thamnocephalis sphaerospora_RKP07020.1:0.5763189819,Syncephalis
pseudoplumigaleata_RKP27957.1:1.1319574213)/1:1.2062193063,Piptocephalis
cylindrospora_RKP11960.1:2.3350975020)/1:0.3642998301)/0.561:0.0656223489)/
1:0.1998352922,((((((((((Mucor ambiguus_GAN02916.1:0.0273024573,Mucor
plumbeus_KAG2192020.1:0.0447547541)/0.84:0.0120582316,Parasitella
parasitica_CEP07434.1:0.0645574957)/1:0.0593382860,Choanephora
cucurbitarum_OBZ88890.1:0.1592494227)/1:0.0453183960,(Mucor
saturninus_KAG2197420.1:0.1196536051,Thamnidium
elegans_KAG2229911.1:0.1244150046)/1:0.0438922003)/1:0.0851611256,(Rhizopus
delemar RA 99-880_EIE84989.1:0.1162287719,(Rhizopus microsporus ATCC
52813_PHZ15536.1:0.0045049463,Rhizopus
azygosporus_RCH89426.1:0.0522854664)/1:0.1225797382)/1:0.1247571381)/
1:0.2785210185,(((((Absidia glauca_SAM08776.1:0.1441778646,Absidia
repens_ORZ18880.1:0.1676956331)/1:0.2458071202,Hesseltinella
vesiculosa_ORX47141.1:0.5780552863)/1:0.1762309937,(Apophysomyces
ossiformis_KAF7726781.1:0.2939204158,Apophysomyces sp.
BC1015_KAG0165557.1:0.1002086329)/1:0.1685371351)/0.99:0.0522468675,(Mucor
circinatus_KAG2219382.1:0.2974804200,Syncephalastrum
racemosum_ORY93769.1:0.3396098596)/1:0.1486133296)/0.807:0.0547868870,Phycomyces
blakesleeanus NRRL
1555_OAD68509.1:0.4430490895)/0.881:0.0526749224)/1:0.3197338596,(Umbelopsis
vinacea_KAG2173065.1:0.0992480238,Umbelopsis
isabellina_KAG2184453.1:0.1657564871)/1:0.3443942482)/1:0.3359859929,Bifiguratus
adelaidae_OZJ04135.1:1.0647434440)/1:0.5101047369,(((((Rhizophagus
clarus_GES86073.1:0.1375167406,(Rhizophagus irregularis DAOM 181602=DAOM
197198_POG81227.1:0.0261911910,Rhizophagus
diaphanus_RGB27877.1:0.0117230631)/1:0.0330996898)/0.48:0.0649172130,Glomus

cerebriforme_RIA99273.1:0.0994893746)/1:0.7681593515,((Gigaspora
margarita_KAF0427424.1:0.0270988597,Gigaspora
rosea_RIB10375.1:0.0141558586)/1:0.5155144190,Diversispora
epigaea_RHZ77424.1:0.6587827682)/0.989:0.1933299751)/1:0.3981437411,Geosiphon
pyriformis_KAG9298860.1:1.2045750829)/1:0.9646910113,(Yarrowia lipolytica
CLIB122_CAG82042.1:2.0110611197,((Tuber melanosporum_CAZ79855.1:0.7950433463,
((((Aspergillus nidulans FGSC A4_EAA63444.1:0.3562038391,Coccidioides immitis
RS_EAS32628.3:0.6233851727)/1:0.2473833963,Exophiala dermatitidis
NIH/UT8656_EHY60670.1:0.6134739408)/1:0.2677323151,(Verticillium dahliae
VdLs.17_EGY16693.1:0.9544368741,Bacidia
gigantensis_KAG8525994.1:0.7310457999)/1:0.1480720117)/0.994:0.1196354100,Zymose
ptoria tritici
IPO323_EGP90800.1:0.8578565135)/1:0.4465527735)/0.995:0.2458744910,(Arthrobotrys
entomopaga_KAF3909733.1:0.4630904581,Drechslerella
brochopaga_KAF3927674.1:0.4280439945)/1:0.5330250212)/1:1.3345684101)/
1:1.5732483362)/0.973:0.1970266004)/0.915:0.1175381941,(((((Mortierella sp.
GBA35_KAF9102125.1:0.0030067868,Mortierella sp.
NVP41_KAG0202999.1:0.0084595695)/1:0.2255731266,(((Mortierella sp.
GBA39_KAF9124791.1:0.0601370729,Linnemannia elongata AG-
77_OAQ22885.1:0.0805422063)/1:0.1387835374,Linnemannia
gamsii_KAG0282600.1:0.1257732109)/1:0.0631439885,Mortierella sp.
AD032_KAG0372599.1:0.2403847289)/1:0.0882838288)/1:0.3408471592,Haplosporangium
sp. Z 767_KAF9184633.1:0.8134928919)/0.977:0.1277834169,Lobosporangium
transversale_ORZ24886.1:0.6194793158)/1:0.2086627113,((Podila
clonocystis_KAG0032469.1:0.1606353606,Podila verticillata NRRL
6337_KFH66337.1:0.2083699097)/0.411:0.0226503482,Podila
minutissima_KAG0361561.1:0.0792151814)/0.996:0.2125729646)/1:1.6045458061)/
1:0.1786856884,(((Linderina pennispora_ORX67084.1:0.7242275741,Coemansia reversa
NRRL 1564_PIA13424.1:0.8264154365)/1:0.8153156358,(Smittium
megazygosporum_PVV02391.1:1.8894469766,Smittium
angustum_PVZ97678.1:1.5040028533)/1:0.6680897153)/1:0.8335373968,Basidiobolus
meristosporus CBS
931.73_ORX83860.1:1.6061053928)/0.976:0.2876151921)/0.732:0.0980187171,Dimargari
s cristalligena_RKP33737.1:1.9822196784)/1:0.2096778470)/0.999:0.1905290980,
((((Batrachochytrium dendrobatidis JAM81_EGF82876.1:1.5998501116,((Spizellomyces
punctatus DAOM BR117_KNC98267.1:0.1371367062,Spizellomyces sp.
'palustris'_TPX70858.1:0.0517342478)/1:0.6757778354,Powellomyces
hirtus_TPX62353.1:1.0511678700)/1:0.5417607774)/0.999:0.3012986257,Synchytrium
microbalum_TPX35704.1:1.5505413988)/1:0.3979042258,(Neocallimastix sp. JGI-
2020a_KAG4088654.1:0.1660224630,((Piromyces
finnis_ORX57165.1:0.0985160263,Piromyces sp.
E2_OUM60763.1:0.0813573891)/1:0.1587068297,Anaeromyces
robustus_ORX79564.1:0.1827655371)/0.68:0.0509534888)/1:2.3815402542)/
1:0.3474896389,((Allomyces macrogynus ATCC
38327_KNE62156.1:0.0858432265,Allomyces macrogynus ATCC
38327_KNE63139.1:0.0459780409)/1:1.1895422099,Catenaria anguillulae
PL171_ORZ32375.1:1.3823521164)/1:0.9014783755)/0.994:0.2205255157)/
1:0.2143241627,((((((((Coprinopsis
cinerea okayama7#130_EAU82675.1:0.3584256318,Amanita muscaria Koide
BX008_KIL70479.1:0.6557304540)/1:0.2442301131,Serpula lacrymans var. lacrymans
S7.9_EGO28024.1:0.4217707642)/1:0.1196155606,Postia placenta Mad-698-
R_EED81092.1:0.1460720139)/1:0.1863675302,Ramaria
rubella_KAF8592300.1:0.5582555137)/1:0.3252492622,Dacryopinax
primogenitus_EJU06674.1:1.0101625585)/0.999:0.3077617129,Rhizoctonia
solani_QRW16070.1:1.0386943927)/1:0.6510896136,Wallemia ichthyophaga EXF-
994_EOQ99282.1:2.3443371921)/0.97:0.2204417113,((Malassezia globosa CBS
7966_EDP43033.1:1.8059946913,Tilletiaria anomala UBC
951_KDN39171.1:1.1333349928)/1:0.9281985002,(((Puccinia graminis f. sp. tritici
CRL 75-36-700-3_EFP91360.2:0.9803298109,Melampsora larici-populina
98AG31_EGG02850.1:1.0124764459)/1:0.8050401492,(Melampsora larici-populina
98AG31_EGG07766.1:1.9332351015,Puccinia graminis f. sp. tritici CRL 75-36-700-
3_EHS64851.1:1.6058648805)/1:0.4346263920)/1:0.3540162194,Mixia osmundae IAM
14324_KEI41979.1:1.9354485398)/1:0.3684421455)/1:0.2417733203)/1:0.1763447160)/
1:0.5008474340,Pneumocystis carinii

B80_KTW26674.1:2.6008502917)/0.998:0.2063644519,Tuber melanosporum_CAZ85975.1:2.0462354117)/0.912:0.1271502868,((Homo sapiens_sp_O94782_UBP1_HUMAN:0.0162132951,Mus musculus_sp_Q8BJQ2_UBP1_MOUSE:0.1404100023)/1:4.3549690517,Pomacea canaliculata_PVD36997.1:4.2729319243)/1:1.6030485076)/0.901:0.1719745499,((((Blumeria graminis f. sp. triticale_CAD6506221.1:1.2058827932,Sclerotinia sclerotiorum 1980 UF-70_EDO01125.1:1.0627208877)/1:0.2127522649,((Aspergillus nidulans FGSC A4_EAA57596.1:0.8211057808,Coccidioides immitis RS_EAS28011.3:1.3633019464)/1:0.4302897499,(Zymoseptoria tritici IPO323_EGP90595.1:1.2358071015,Bacidia gigantensis_KAG8530394.1:1.2937046546)/0.769:0.1576459542)/1:0.2520316843)/1:0.2034079737,(Neurospora crassa OR74A_EAA26609.1:1.0979552685,(Trichoderma reesei QM6a_EGR49253.1:0.8080107062,Verticillium dahliae VdLs.17_EGY18901.1:0.8896245874)/1:0.2010661101)/0.999:0.1376775077)/1:0.3195455212,Exophiala dermatitidis NIH/UT8656_EHY53120.1:2.0499392794)/1:0.8850623034)/1:0.6288122578,Yarrowia lipolytica CLIB122_CAG81669.1:2.1333502290)/1:0.9067274122,Candida albicans SC5314_AOW28595.1:1.2380462783);

****

XPF

****

(Mucor ambiguus_GAN08149.1:0.0785923461,(((((((((((Rhizophagus clarus_GES78268.1:0.0692832472,(((Rhizophagus irregularis DAOM 181602=DAOM 197198_POG62828.1:0.2344522004,Rhizophagus diaphanus_RGB29554.1:0.0065589329)/0:0.0000010902,(Rhizophagus irregularis DAOM 181602=DAOM 197198_POG78933.1:0.0037076959,Rhizophagus diaphanus_RGB39369.1:0.6771606664)/0.132:0.0001807432)/1:0.0297572623,Rhizophagus irregularis DAOM 181602=DAOM 197198_POG68415.1:0.2736822669)/1:0.0324884100)/1:0.0454085687,Glomus cerebriforme_RIA94468.1:0.1035950856)/1:0.3895552075,((Gigaspora margarita_KAF0544907.1:0.0236096000,(Gigaspora rosea_RIB03254.1:0.0788979293,(Gigaspora rosea_RIB03255.1:0.0666762164,Gigaspora rosea_RIB07426.1:0.0000022348)/0.803:0.0096846404)/0.432:0.0077958065)/1:0.2504529669,(Diversispora epigaea_RHZ83651.1:0.2143310590,Gigaspora rosea_RIB09941.1:0.8060879011)/0.884:0.1079135233)/1:0.1462706407)/1:0.1769542350,Geosiphon pyriformis_KAG9288310.1:0.6087826814)/1:0.2053791691,((((Schizosaccharomyces pombe_CAA20694.2:0.5856297058,Schizosaccharomyces japonicus yFS275_EEB07471.2:0.5799032915)/1:0.5994603544,(Yarrowia lipolytica CLIB122_CAG83466.1:1.5037922673,(Candida albicans SC5314_AOW28016.1:1.0287720088,(Saccharomyces cerevisiae S288C_DAA11406.1:0.0000027688,Saccharomyces cerevisiae S288C_NP_015303.1:0.0000022012)/1:1.3500021192)/1:0.5997051623)/1:0.4056483668)/0.89:0.1103954357,(((((((Blumeria graminis f. sp. triticale_CAD6502742.1:0.3206084268,Sclerotinia sclerotiorum 1980 UF-70_EDO03119.1:0.1698962716)/1:0.0885329736,(Neurospora crassa OR74A_EAA35391.3:0.2667368827,(Trichoderma reesei QM6a_EGR51884.1:0.1868857740,Verticillium dahliae VdLs.17_EGY19777.1:0.1875619011)/1:0.0562530582)/1:0.1281845015)/1:0.1193168375,Zymoseptoria tritici IPO323_EGP87699.1:0.4955462968)/0.705:0.0403039477,(((Aspergillus nidulans FGSC A4_EAA60262.1:0.3846801044,Coccidioides immitis RS_EAS30298.2:0.3787380976)/1:0.1947873600,Exophiala dermatitidis NIH/UT8656_EHY57314.1:0.3969070102)/1:0.0716934522,Bacidia gigantensis_KAG8528736.1:0.3461407993)/1:0.0611564559)/1:0.1742417592,Tuber melanosporum_CAZ86654.1:0.4513601198)/0.998:0.0897376145,((Orbilia oligospora ATCC 24927_EGX45931.1:0.5504525677,Arthrobotrys entomopaga_KAF3916020.1:0.3971368590)/0.883:0.0935259500,Drechslerella brochopaga_KAF3901651.1:0.5790593235)/1:0.5135434567)/1:0.1412713894,Neolecta irregularis DAH-3_OLL25916.1:0.6040631936)/1:0.1583458904,Pneumocystis carinii B80_KTW27428.1:1.1080688221)/1:0.1437714427)/1:0.3163122201,((((Cryptococcus neoformans var. grubii H99_AFR98957.2:1.4266868213,((((Coprinopsis cinerea okayama7#130_EAU93561.2:0.4160794296,Amanita muscaria Koide

BX008_KIL60371.1:0.4371795621)/1:0.1218559388,(Postia placenta Mad-698-R_EED82547.1:0.4348485169,Serpula lacrymans var. lacrymans S7.9_EGO19667.1:0.3055548131)/1:0.0750861256)/0.874:0.1046499238,Ramaria rubella_KAF8580272.1:0.5348993094)/1:0.1692354351,(Dacryopinax primogenitus_EJT99777.1:0.7199962274,Rhizoctonia solani_QRW20713.1:0.8139777006)/0.814:0.0976316609)/1:0.2676627621)/1:0.1845758282,Wallemia ichthyophaga EXF-994_EOR02855.1:1.1314589118)/0.995:0.1113255971,(Malassezia globosa CBS 7966_EDP43920.1:1.0940585003,(Tilletiaria anomala UBC 951_KDN53411.1:0.5372973913,Ustilago maydis 521_KIS68614.1:0.4909926231)/0.989:0.1210936204)/1:0.1915582912)/1:0.0723221218,(((Puccinia graminis f. sp. tritici CRL 75-36-700-3_EFP79010.2:0.3730869931,Puccinia graminis f. sp. tritici CRL 75-36-700-3_EHS62509.1:0.0000028890)/1:0.5797713433,Melampsora larici-populina 98AG31_EGG12539.1:0.4692890127)/1:0.4283101234,Mixia osmundae IAM 14324_KEI42826.1:1.6009356806)/0.977:0.1149132022)/1:0.1843360595)/1:0.1343339812)/1:0.1124602907,((((((((((((((((Encephalitozoon intestinalis ATCC 50506_ADM12005.2:0.2312630108,(Encephalitozoon hellem ATCC 50504_AFM98774.1:0.1857337782,Encephalitozoon romaleae SJ-2008_AFN83491.1:0.1885037309)/1:0.1806720988)/1:0.4227605300,Ordospora colligata OC4_KHN69148.1:0.8518805289)/1:0.7031150124,(Nosema granulosis_KAF9764705.1:1.2302710724,(Nosema granulosis_KAF9764706.1:1.3384290478,Nosema ceranae_KKO76506.1:0.7761927688)/1:0.6900869352)/1:0.5236510619)/1:0.3967610090,((Edhazardia aedis USNM 41457_EJW04814.1:1.8208311075,(Hamiltosporidium magnivora_TBU05612.1:0.0123953490,Hamiltosporidium tvaerminnensis_TBU13589.1:0.0017664876)/1:1.0266729568)/0.993:0.2028769402,(Thelohania contejeani_KAF7683044.1:1.5032170577,(Dictyocoela roeselum_KAG0419121.1:0.3864685098,Dictyocoela muelleri_KAG0437317.1:0.4785763725)/1:1.5868000013)/0.9:0.2044513644)/1:0.1648934385)/0.997:0.2207669206,((Vittaforma corneae ATCC 50505_ELA42845.1:1.3300186721,Hepatospora eriocheir_ORD97693.1:2.5619742382)/0.281:0.2132115612,(Enterocytozoon hepatopenaei_OQS55870.1:2.0617055162,Enterospora canceri_ORD95125.1:1.5445194526)/1:0.6827252607)/1:0.5468285132)/1:0.1747793529,((((Vavraia culicis subsp. floridensis_ELA48066.1:0.4409531248,Trachipleistophora hominis_ELQ74038.1:0.2606891699)/1:1.1095955109,Pseudoloma neurophilia_KRH95318.1:1.3898530502)/1:0.9348651439,Cucumispora dikerogammari_KAF7700512.1:2.3682374397)/0.99:0.2754468210,((Anncaliia algerae PRA339_KCZ81357.1:0.6328428024,Tubulinosema ratisbonensis_RVD93322.1:0.3696721208)/0.676:0.1291317097,Anncaliia algerae PRA339_KCZ81358.1:0.3671087239)/1:1.6088270324)/0.768:0.1237288478)/1:0.6611515355,(((Nematocida parisii ERTm1_EIJ92805.1:0.0616368508,Nematocida sp. ERTm5_OAG33466.1:0.0568836056)/1:0.6513770584,Nematocida sp. 1 ERTm6_KFG25940.1:0.7205383625)/1:1.1676348163,Nematocida displodere_OAG32315.1:2.0579189892)/1:3.0505555703)/0.945:0.3370673490,((Spizellomyces punctatus DAOM BR117_KNC96281.1:0.5974144864,Powellomyces hirtus_TPX61370.1:0.6431188729)/1:0.5619868872,(Rhizoclosmatium globosum_ORY52110.1:0.6922624905,Chytriomyces confervae_TPX77543.1:0.4634499980)/1:0.5272871974)/1:3.1811415356)/0.995:0.2890245263,Mitosporidium daphniae_KGG51961.1:3.5272708404)/0.846:0.1987781136,(Mitosporidium daphniae_KGG53205.1:2.8287661913,Fonticula alba_XP_009493082.1:3.2716824075)/0.99:0.6010620715)/1:0.2248417175,(Rozella allomycis CSF55_EPZ37000.1:1.6925311530,Paramicrosporidium saccamoebae_PJF18489.1:1.7421245613)/0.486:0.0676254373)/1:0.2345778883,((((((Homo sapiens_NP_005227.1:0.1097340461,Mus musculus_NP_056584.2:0.1368990740)/1:0.6209546399,Pomacea canaliculata_PVD23305.1:0.7784526049)/1:0.1819262573,Drosophila melanogaster_NP_525068.1:1.6033515609)/1:0.1527039160,(Amphimedon queenslandica_XP_003385158.1:1.0426010149,Sphaeroforma arctica JP610_XP_014156463.1:1.3986484632)/0.556:0.0754153482)/0.962:0.2120341112,Caenorhabditis elegans_NP_496498.1:3.1905587295)/0.97:0.1410650292,((Monosiga

brevicollis MX1_XP_001742589.1:1.2041591869,Salpingoeca
rosetta_XP_004987615.1:1.0324160453)/1:0.6853513308,Capsaspora owczarzaki ATCC
30864_XP_004343438.1:1.2382811531)/0.911:0.1061511408)/0.999:0.1593880555,Dictyo
stelium discoideum
AX4_XP_638619.1:2.3270335824)/0.654:0.0788588367)/1:0.1333383508,((Entomophthora
muscae_KAF7744799.1:1.0433125565,Conidiobolus coronatus NRRL
28638_KXN75066.1:1.3829269478)/1:0.3520521354,((Allomyces macrogynus ATCC
38327_KNE62471.1:0.0534058140,Allomyces macrogynus ATCC
38327_KNE65850.1:0.1593555728)/1:0.6658849740,Catenaria anguillulae
PL171_ORZ36503.1:1.2374180009)/1:0.9824143042)/1:0.3256242876)/0.785:0.115985731
1,Gonapodya prolifera JEL478_KXS17054.1:1.6995362537)/0.979:0.1481368668,
((((Batrachochytrium dendrobatidis JAM81_EGF76253.1:0.9565655171,
(Rhizoclosmatium globosum_ORY53589.1:0.3733489564,Chytriomyces
confervae_TPX78583.1:0.6173958692)/1:0.7451714669)/0.975:0.1044679236,
((((Spizellomyces punctatus DAOM BR117_KNC97326.1:0.0182796030,Spizellomyces sp.
'palustris'_TPX66046.1:0.0411189855)/1:0.2700457878,Powellomyces
hirtus_TPX55659.1:0.4618486339)/1:0.2525598534,Blyttiomyces
helicus_RKO94222.1:0.8312130704)/0.965:0.0713863874,(Synchytrium
microbalum_TPX33576.1:0.2466774163,Synchytrium
endobioticum_TPX41493.1:0.3426499083)/1:0.4814712559)/0.992:0.0778598507)/
1:0.1132229815,((((((Neocallimastix sp. JGI-
2020a_KAG4083307.1:0.0000020581,Neocallimastix
californiae_ORY14852.1:0.0059821896)/1:0.1292320724,Anaeromyces
robustus_ORX83961.1:0.1234727008)/1:0.0939151808,Piromyces sp.
E2_OUM62412.1:0.0528736317)/0.822:0.0341063005,(Piromyces
finnis_ORX34880.1:0.0966249834,Piromyces
finnis_ORX49370.1:0.0000021109)/1:0.0996663169)/1:0.9722187710,Gonapodya
prolifera JEL478_KXS17050.1:1.6970959813)/0.88:0.3397142682,Caulochytrium
protostelioides_RKP00956.1:1.2701223442)/0.726:0.0973610733)/0.808:0.0829630622,
Gonapodya prolifera
JEL478_KXS17053.1:2.1342573614)/0.994:0.1290141728)/0.997:0.0503074634,
(((((((((Haplosporangium bisporale_KAF8920446.1:0.0000027949,Podila
verticillata NRRL
6337_KFH63742.1:0.0000026467)/0.128:0.0000021880,Haplosporangium
bisporale_KAF9004959.1:0.0000020376)/0.416:0.0040991323,Haplosporangium
bisporale_KAF9013755.1:0.0047194780)/1:0.0563342494,(Podila
horticola_KAF9313080.1:0.0239019879,(Podila
clonocystis_KAG0022711.1:0.0256935904,(Podila
epicladia_KAG0097557.1:0.0149534046,Podila
minutissima_KAG0345703.1:0.0229570005)/1:0.0134192764)/0.982:0.0039218341)/
1:0.0216865411)/1:0.0574979812,Podila
humilis_KAG0348609.1:0.2292006066)/1:0.0456655987,Podila
epigama_KAF9427926.1:0.1282013895)/1:0.0637461247,(((((Dissophora
ornata_KAF8925803.1:0.0703642841,Dissophora
globulifera_KAG0326679.1:0.1044686257)/1:0.0248012895,((((((Entomortierella
lignicola_KAF8983480.1:0.0049972095,Haplosporangium sp. Z
27_KAF9208092.1:0.0041707715)/1:0.0855452316,(((Mortierella sp.
AD010_KAF9169504.1:0.0000021833,Mortierella sp.
AD011_KAF9398341.1:0.0000020336)/1:0.0310862889,Entomortierella
chlamydospora_KAG0001137.1:0.0177528150)/1:0.0286039820,Mortierella sp.
AD094_KAF9360601.1:0.0089816308)/1:0.0378234578)/0.995:0.0183029182,Mortierella
sp. AM989_KAF9110385.1:0.0574998574)/0.986:0.0200432990,Entomortierella
beljakovae_KAF9437889.1:0.1591377785)/1:0.0353454047,(((Mortierella sp.
NVP85_KAF9361226.1:0.0904815294,Mortierella
sp. GBA43_KAG0239570.1:0.1243861503)/0.959:0.0157758759,Modicella
reniformis_KAF9988517.1:0.0913507275)/1:0.0544876341,Lobosporangium
transversale_ORZ05671.1:0.1466401923)/1:0.0244239560)/1:0.0256065404,Gamsiella
multidivaricata_KAG0366786.1:0.1144897247)/0.986:0.0150197047)/1:0.0391512191,
(((Haplosporangium sp. Z 767_KAF9189002.1:0.0000023308,Haplosporangium sp. Z
11_KAF9191220.1:0.0000025142)/1:0.0268456506,Mortierella
polycephala_KAG0264658.1:0.0346551349)/1:0.1287797452,(Mortierella
antarctica_KAF9989106.1:0.0934054121,Mortierella sp.
GBA30_KAG0212587.1:0.0834448869)/1:0.0375651710)/0.981:0.0122749270)/
1:0.0368294631,(((((Haplosporangium

gracile_KAF8940026.1:0.0145768950,Linnemannia
schmuckeri_KAF9147003.1:0.0198966691)/1:0.0056626779,Linnemannia
gamsii_KAG0296422.1:0.0511981220)/0.678:0.0037787452,((Mortierella sp.
GBA39_KAF9140466.1:0.0132642826,(Mortierella
hygrophila_KAF9551216.1:0.0130927457,Linnemannia
hyalina_KAG9063825.1:0.0088163579)/0.134:0.0000024563)/1:0.0155863313,Linnemanni
a elongata AG-77_OAQ26957.1:0.0185113348)/1:0.0065851753)/1:0.0283683532,
((Mortierella sp. 14UC_KAF9125554.1:0.0222763274,Linnemannia
zychae_KAF9913274.1:0.0329002751)/1:0.0086597626,(Linnemannia
exigua_KAG0275130.1:0.0305378936,Mortierella sp.
AD032_KAG0378338.1:0.0157971958)/1:0.0205385534)/1:0.0394829679)/1:0.0336335298,
(Mortierella sp. AD031_KAF9099052.1:0.0016382071,(Mortierella sp.
GBA35_KAF9104749.1:0.0032720896,Mortierella sp.
NVP41_KAG0217532.1:0.0000024743)/0.126:0.0000021364)/1:0.0371347215)/
1:0.1430113196)/0.973:0.0232195894,(Lunasporangiospora
selenospora_KAF9585211.1:0.3680790286,Gryganskiella
cystojenkinii_KAG0050984.1:0.1720164982)/0.846:0.0531242366)/1:0.0725895578)/
1:0.0828212527,Lunasporangiospora
selenospora_KAF9585212.1:0.2625242919)/1:0.1847892762,(Actinomortierella
wolfii_KAG0242467.1:0.0762515247,Actinomortierella
ambigua_KAG0259504.1:0.0843307116)/1:0.2548888248)/0.993:0.2186938574,
(Piptocephalis cylindrospora_RKP11516.1:0.0000020076,Piptocephalis
cylindrospora_RKP11517.1:0.0000029030)/1:1.9846753203)/1:0.5255057469)/
0.988:0.0721226942,(((Olpidium bornovanus_KAG5458437.1:0.9352269041,Olpidium
bornovanus_KAG5462829.1:0.0000021081)/0.8:0.4304868329,Piptocephalis
cylindrospora_RKP11404.1:1.2451145153)/0.935:0.4087518733,((((((((Smittium
mucronatum_OLY78857.1:0.2627665350,Smittium
culicis_OMJ16677.1:0.3120431624)/0.905:0.0809905181,Smittium
culicis_OMJ16678.1:0.2782934499)/1:0.3491259441,(Furculomyces
boomerangus_PVU99261.1:0.0000028867,Smittium
angustum_PWA00658.1:0.0028608767)/1:0.6977833769)/1:0.1411754575,(Smittium
simulii_PVU88444.1:0.3953062874,Smittium
megazygosporum_PVV02734.1:0.4899239963)/1:0.2553281415)/1:0.4573292376,Zancudomy
ces culisetae_OMH80751.1:1.7598090653)/1:0.3482944191,Coemansia reversa NRRL
1564_PIA18493.1:1.3211542507)/1:0.3402850487,Dimargaris
cristalligena_RKP38070.1:0.9780972660)/1:0.2006536450,((Thamnocephalis
sphaerospora_RKP08935.1:0.4605289453,Syncephalis
pseudoplumigaleata_RKP23673.1:0.4026592303)/0.74:0.1195485783,(Syncephalis
pseudoplumigaleata_RKP23098.1:0.0000026692,Syncephalis
pseudoplumigaleata_RKP23345.1:0.0495193335)/1:0.6896155591)/1:0.4335108995)/
1:0.1159242974)/0.959:0.0607274960)/1:0.0990088257,(Basidiobolus meristosporus
CBS 931.73_ORX93909.1:0.4064706519,Sphaeroforma arctica
JP610_XP_014156464.1:1.4937302492)/0.999:0.2907249763)/1:0.0742284900)/
1:0.1669974253,Bifiguratus adelaidae_OZJ03595.1:0.6294116874)/1:0.3647639861,
(Umbelopsis isabellina_KAG2184589.1:0.1704302918,Umbelopsis
vinacea_KAG2188569.1:0.2885578774)/1:0.4170500240)/1:0.3009671362,(((Absidia
glauca_SAM07091.1:0.2420087177,Absidia
repens_ORZ22348.1:0.1570218061)/1:0.1980968741,Hesseltinella
vesiculosa_ORX60262.1:0.5122277310)/1:0.2612893814,(((Apophysomyces
ossiformis_KAF7726193.1:0.1533372157,((Apophysomyces sp.
BC1015_KAG0173332.1:0.0027381777,Apophysomyces sp.
BC1034_KAG0192545.1:0.0022900575)/0.993:0.0015192261,Apophysomyces sp.
BC1021_KAG0182633.1:0.0000021789)/1:0.1993177098)/1:0.2140537311,Phycomyces
blakesleeanus NRRL 1555_OAD66009.1:0.9202405830)/1:0.1018590041,(Mucor
circinatus_KAG2226446.1:0.5425386544,Syncephalastrum
racemosum_ORY94875.1:0.4944189188)/1:0.0919749394)/0.996:0.0620139121)/
0.996:0.0628480379)/1:0.2835333604,(Rhizopus delemar RA 99-
880_EIE76768.1:0.2351361397,((Rhizopus microsporus ATCC
52813_PHZ10910.1:0.0000029599,Rhizopus
azygosporus_RCH81815.1:0.0000026535)/0.594:0.0088622720,Rhizopus
azygosporus_RCH93967.1:0.0155869071)/1:0.1641730395)/1:0.2531042865)/
1:0.0894170627,(Mucor saturninus_KAG2213458.1:0.3427889206,Thamnidium
elegans_KAG2230880.1:0.2412328221)/1:0.0702560580)/0.993:0.0583427991,Choanephor
a cucurbitarum_OBZ86984.1:0.3204877890)/1:0.1694430709,(Parasitella

parasitica_CEP16749.1:0.1628948757,Mucor
plumbeus_KAG2196440.1:0.0929957914)/0.99:0.0292333922)/1:0.0716889881,Mucor
lusitanicus_KAF1801439.1:0.0817324298);

****
BRCA1
****
(Drosophila melanogaster_AAG27544.1:2.0000520955,((Caenorhabditis
elegans_CAR97812.2:2.2421963964,(Pomacea canaliculata_PVD29745.1:1.4153477318,
(Homo sapiens_NP_009225.1:0.2767257146,Mus
musculus_NP_033894.3:0.3475940167)/1:1.0464919712)/0.979:0.1749765641)/
0.679:0.0928330125,(((((Amphimedon queenslandica_A0A1X7VV49:2.1192170920,
((((((((Podila clonocystis_KAG0020979.1:0.1220741672,(Podila
epicladia_KAG0097622.1:0.0797178236,Podila
minutissima_KAG0354002.1:0.0363554137)/1:0.0432320394)/0.906:0.0074417192,Podila
horticola_KAF9315689.1:0.1081781477)/1:0.0569460768,(Haplosporangium
bisporale_KAF9026974.1:0.8679263266,Podila verticillata NRRL
6337_KFH66509.1:0.0198188670)/1:0.1150315845)/1:0.2171685091,Podila
humilis_KAG0338598.1:0.4915093625)/1:0.3464523547,Podila
epigama_KAF9417466.1:0.6679252142)/1:0.3906572223,(((((Entomortierella
beljakovae_KAF9438621.1:0.5433033989,(((((Mortierella sp.
AD010_KAF9175997.1:0.0549804577,Entomortierella
chlamydospora_KAG0006080.1:0.0208846526)/0.15:0.0012414042,Mortierella sp.
AD011_KAF9403207.1:0.0292392493)/1:0.0742163235,Mortierella sp.
AD094_KAF9352438.1:0.0651683652)/1:0.1153511476,(Entomortierella
lignicola_KAF8986176.1:0.0109198214,Haplosporangium sp. Z
27_KAF9209886.1:0.0118653326)/1:0.2602936982)/0.87:0.0457720552,Mortierella sp.
AM989_KAF9110724.1:0.2963626598)/0.997:0.0879860874)/1:0.1584198823,(Modicella
reniformis_KAG0007149.1:0.4312818311,(Mortierella sp.
NVP85_KAF9358604.1:0.3220679791,Mortierella sp.
GBA43_KAG0235557.1:0.3092793053)/1:0.1599719974)/1:0.2940266780)/1:0.1367633370,
((Haplosporangium sp. Z 767_KAF9186476.1:0.0000035207,Haplosporangium sp. Z
11_KAF9187458.1:0.0000029437)/1:0.0779795776,Mortierella
polycephala_KAG0259601.1:0.0717027339)/1:0.4589510676)/1:0.0686249122,
((Lunasporangiospora selenospora_KAF9584145.1:0.7349932056,(((Mortierella sp.
AD032_KAG0373929.1:0.2071477191,Linnemannia
exigua_KAG0277626.1:0.0703346550)/0.986:0.0508873486,(Mortierella sp.
14UC_KAF9129334.1:0.0725625737,Linnemannia
zychae_KAF9900947.1:0.1445077342)/1:0.0378149623)/0.997:0.0430910681,
((((Haplosporangium gracile_KAF8945593.1:0.0695964809,Linnemannia
schmuckeri_KAF9155002.1:0.0493122902)/1:0.0506055635,(((Mortierella sp.
GBA39_KAF9138777.1:0.0187421209,Linnemannia
hyalina_KAG9067754.1:0.0235189161)/1:0.0098704362,Mortierella
hygrophila_KAF9538920.1:0.0311349160)/1:0.0326786720,Linnemannia elongata AG-
77_OAQ31332.1:0.0706458916)/1:0.0646146012)/0.996:0.0352576680,Linnemannia
gamsii_KAG0288886.1:0.1401881656)/1:0.0982218486,(Mortierella sp.
AD031_KAF9090455.1:0.0018192385,Mortierella sp.
GBA35_KAF9101472.1:0.0000032206)/1:0.2096170474)/0.955:0.0373249214)/
1:0.3653724596)/0.995:0.0760394615,Gryganskiella
cystojenkinii_KAG0054152.1:0.7397275948)/0.742:0.0283322876)/0.997:0.1642115833,
Mortierella
antarctica_KAF9989181.1:0.6882205470)/0.426:0.0796482800)/0.997:0.1762267448,Act
inomortierella wolfii_KAG0235271.1:1.1087511767)/0.999:0.2802526925,
((Entomophthora muscae_KAF7743574.1:0.0000033265,Entomophthora
muscae_KAF7758863.1:0.0884681715)/1:1.9061900865,Sphaeroforma arctica
JP610_XP_014153862.1:2.3373115665)/0.818:0.2180462202)/0.965:0.1629898739)/
0.84:0.0663821464,(Endogone sp. FLAS-
F59071_RUS19088.1:0.9359210105,Jimgerdemannia
flammicorona_RUP43471.1:0.3228988844)/1:0.8240425820)/0.629:0.0611882940,Paramic
rosporidium saccamoebae_PJF17061.1:1.7514023247)/1:0.2056016233,((Dictyostelium
discoideum AX4_EAL60774.1:1.5259068125,Umbelopsis
isabellina_KAG2182063.1:1.5961067578)/0.747:0.1969088860,Apophysomyces

ossiformis_KAF7722156.1:1.9038402877)/0.609:0.0927369261)/0.983:0.0516921405,
(Capsaspora owczarzaki ATCC 30864_XP_004366013.1:1.3685281204,(Salpingoeca
rosetta_XP_004998238.1:1.2644430988,Monosiga brevicollis
MX1_XP_001746034.1:1.4638573572)/1:0.3419383014)/0.968:0.1570592027)/
0.987:0.0855230683)/0.999:0.3791215787,Fonticula
alba_XP_009492366.1:1.7942271987);

****
BRCA2
****
(Homo sapiens_NP_000050.2:0.3060797578,Mus musculus_NP_033895.2:0.3305983637,
((((Dictyostelium discoideum AX4_EAL60741.1:2.2449034016,
(((((((((Caenorhabditis elegans_NP_498502.3:4.3240782456,(Thamnocephalis
sphaerospora_RKP04649.1:0.8803493264,Syncephalis
pseudoplumigaleata_RKP25322.1:1.2419930153)/1:1.6543218509)/0.881:0.2948725413,
(((Cryptococcus neoformans var. grubii H99_AFR96486.2:1.6950271012,((Melampsora
larici-populina 98AG31_EGG11618.1:1.3895417905,Puccinia graminis f. sp. tritici
CRL 75-36-700-3_EHS62527.1:1.0390492384)/1:1.0911158129,(Wallemia ichthyophaga
EXF-994_EOQ99450.1:1.6539646441,Mixia osmundae IAM
14324_KEI39015.1:1.9515325170)/0.984:0.2097188555)/0.94:0.1633518451)/
0.997:0.1203288003,(((((Coprinopsis cinerea
okayama7#130_EAU92556.2:0.8960846564,Amanita muscaria Koide
BX008_KIL71541.1:0.7506782948)/0.996:0.1585495352,((Postia placenta Mad-698-
R_EED79697.1:0.0293551024,Postia placenta
Mad-698-R_EED85186.1:0.0157881279)/1:1.3958110688,(Serpula lacrymans var.
lacrymans S7.9_EGO30985.1:0.8238353161,Ramaria
rubella_KAF8585115.1:0.9233359648)/0.686:0.0694693136)/0.884:0.0962659779)/
1:0.5104617861,Rhizoctonia
solani_QRW22267.1:1.6760514810)/0.993:0.1577211484,Dacryopinax
primogenitus_EJU02787.1:1.8169250376)/0.999:0.2173568596,((Malassezia globosa
CBS 7966_EDP42738.1:1.7413413904,Tilletiaria anomala UBC
951_KDN43004.1:1.4659511112)/0.868:0.2244814794,Ustilago maydis
521_KIS68625.1:1.3430456802)/1:0.3560164005)/0.617:0.1236311883)/0.939:0.1464153
538,Malassezia globosa CBS
7966_EDP42737.1:2.0606123614)/1:0.4019768412)/0.918:0.0871855316,((Allomyces
macrogynus ATCC 38327_KNE55806.1:0.0691135636,Allomyces macrogynus ATCC
38327_KNE58608.1:0.0696734526)/1:1.5641260718,Catenaria anguillulae
PL171_ORZ39398.1:1.6671576577)/1:0.6944427637)/0.958:0.0833656008,Gonapodya
prolifera JEL478_KXS19788.1:2.5893748536)/0.749:0.0789221527,((((((((Dissophora
ornata_KAF8932065.1:0.3991563180,Dissophora
globulifera_KAG0321648.1:0.5521314954)/1:0.1236718682,(((Mortierella sp.
NVP85_KAF9347003.1:0.6548001197,Mortierella sp.
GBA43_KAG0241040.1:0.8179642298)/1:0.1118529991,Modicella
reniformis_KAG0006737.1:0.5098834498)/1:0.1998364878,Gamsiella
multidivaricata_KAG0366571.1:0.5855787433)/1:0.0661766968)/0.997:0.0614434513,
((((Entomortierella lignicola_KAF8978861.1:0.0182060946,Haplosporangium sp. Z
27_KAF9203400.1:0.0163240955)/1:0.4406589199,((Mortierella sp.
AD010_KAF9173626.1:0.0000020069,Mortierella sp.
AD011_KAF9399320.1:0.0000028597)/1:0.0650966615,Entomortierella
chlamydospora_KAG0002160.1:0.0414095252)/1:0.2448500830)/1:0.0918124632,Mortiere
lla sp. AM989_KAF9116996.1:0.3387233301)/1:0.1066449305,Entomortierella
beljakovae_KAF9437101.1:0.7160390458)/1:0.2304784801)/1:0.0947526383,
((((((Haplosporangium gracile_KAF8932546.1:0.0670778598,Linnemannia
schmuckeri_KAF9147261.1:0.0912364515)/1:0.0671741828,((Mortierella sp.
GBA39_KAF9128491.1:0.0345350818,(Mortierella
hygrophila_KAF9537439.1:0.0444745821,Linnemannia
hyalina_KAG9061184.1:0.0377056106)/1:0.0164117050)/1:0.0847922475,Linnemannia
elongata
AG-77_OAQ25884.1:0.0908677901)/1:0.0668949659)/1:0.0256911591,Linnemannia
gamsii_KAG0282851.1:0.1866515910)/1:0.1155218165,((Mortierella sp.
14UC_KAF9128163.1:0.1301893343,Linnemannia
zychae_KAF9909039.1:0.2043606813)/1:0.0681733804,Mortierella sp.

AD032_KAG0381166.1:0.2041885753)/1:0.1339769917)/1:0.0617967883,((Mortierella
sp. AD031_KAF9097020.1:0.0016422245,Mortierella sp.
NVP41_KAG0217799.1:0.0005455117)/0.979:0.0023085873,Mortierella sp.
GBA35_KAF9097209.1:0.0083559773)/1:0.2249459839)/1:0.5648887410,
((Haplosporangium sp. Z 11_KAF9187721.1:0.0828334449,Mortierella
polycephala_KAG0249873.1:0.0974635601)/1:0.6370170991,(Mortierella
antarctica_KAF9984697.1:0.5291646025,Mortierella sp.
GBA30_KAG0197828.1:0.7146692094)/1:0.4286827592)/0.999:0.0952613572)/
0.899:0.0542035393)/1:0.2247850526,Gryganskiella
cystojenkinii_KAG0056739.1:0.9333712617)/1:0.0834772410,((((Haplosporangium
bisporale_KAF9020812.1:0.0396268572,Podila verticillata NRRL
6337_KFH73131.1:0.0093713025)/1:0.2194475458,(Podila
horticola_KAF9321102.1:0.1073263567,(Podila
clonocystis_KAG0035654.1:0.1092884697,(Podila
epicladia_KAG0100149.1:0.1020481512,Podila
minutissima_KAG0352359.1:0.0509325678)/1:0.0313068017)/1:0.0326063759)/
1:0.0665340589)/1:0.3563893586,Podila
humilis_KAG0349554.1:0.6565577615)/1:0.2129438434,Podila
epigama_KAF9426293.1:0.6471933037)/1:0.6802312538)/0.554:0.0825858576,Lunasporan
giospora selenospora_KAF9586508.1:0.9785656799)/1:0.2293492258,
(Actinomortierella wolfii_KAG0236096.1:0.3554739113,Actinomortierella
ambigua_KAG0255865.1:0.3156717831)/1:1.0664943475)/1:0.5237352746)/
0.993:0.1151228960,(((((((((((Mucor ambiguus_GAN10595.1:0.0979394800,Mucor
lusitanicus_KAF1804125.1:0.1370241023)/1:0.1298527582,(Parasitella
parasitica_CEP13748.1:0.3397408751,Mucor
plumbeus_KAG2209111.1:0.1963665618)/1:0.0796002924)/1:0.4750338702,Choanephora
cucurbitarum_OBZ84355.1:0.6154325967)/1:0.1785933783,(Mucor
saturninus_KAG2204560.1:0.6267860738,Thamnidium
elegans_KAG2233272.1:0.5806206077)/1:0.4100844482)/1:0.1301542048,((Rhizopus
delemar RA 99-880_EIE85097.1:0.5683684145,Rhizopus delemar RA 99-
880_EIE87793.1:0.4609576156)/0.999:0.1197514952,(Rhizopus microsporus ATCC
52813_PHZ14206.1:0.0486762043,Rhizopus
azygosporus_RCH82259.1:0.0498819876)/1:0.3730226703)/1:0.3120188491)/
1:0.4414196646,(((Absidia glauca_SAL98464.1:0.4803206817,Absidia
repens_ORZ18475.1:0.5556304055)/1:0.4589776926,Hesseltinella
vesiculosa_ORX61294.1:1.1580204278)/1:0.6101422627,(Apophysomyces
ossiformis_KAF7721120.1:0.4703637328,(Apophysomyces sp.
BC1015_KAG0175092.1:0.0013047039,(Apophysomyces sp.
BC1021_KAG0180309.1:0.0013144849,Apophysomyces sp.
BC1034_KAG0194791.1:0.0000022030)/0.126:0.0000020349)/1:0.5426330912)/
1:0.5500690923)/0.861:0.1454636070)/0.997:0.1216281473,Syncephalastrum
racemosum_ORY90596.1:1.6804766528)/0.876:0.1058910623,Phycomyces blakesleeanus
NRRL 1555(-)_OAD72998.1:1.3903728656)/0.993:0.1427323016,Mucor
circinatus_KAG2221581.1:1.2145650418)/1:0.3257977153,Basidiobolus meristosporus
CBS 931.73_ORX93295.1:1.4099177150)/0.98:0.1854270275,(Jimgerdemannia
flammicorona_RUP24650.1:0.5325696785,(Jimgerdemannia
flammicorona_RUP44701.1:0.6423256974,Endogone sp. FLAS-
F59071_RUS19357.1:0.6471616880)/0.995:0.3823402189)/1:0.6564929512)/
1:0.2431067335,(((Rhizophagus clarus_GES77689.1:0.1394334961,(Rhizophagus
irregularis DAOM 181602=DAOM 197198_POG61700.1:0.0149202169,Rhizophagus
diaphanus_RGB27511.1:0.0275382598)/1:0.1165375244)/1:0.1165029255,Glomus
cerebriforme_RIA85350.1:0.2434218562)/1:0.6713890725,(((Gigaspora
margarita_KAF0381412.1:0.0324707071,Gigaspora
rosea_RIB02779.1:0.0855627935)/1:0.6658188986,Diversispora
epigaea_RHZ83030.1:1.2427145549)/0.644:0.0973448782,Diversispora
epigaea_RHZ83015.1:0.6241403557)/0.354:0.1358953816)/1:0.8704413875)/
1:0.2544338800,(((((Neocallimastix sp. JGI-
2020a_KAG4099534.1:0.0015236776,Neocallimastix
californiae_ORY81143.1:0.0006224826)/1:0.3546376228,(Piromyces
finnis_ORX55527.1:0.2783251287,Piromyces sp.
E2_OUM68278.1:0.1690776235)/1:0.1823712504)/0.908:0.0535911333,(Anaeromyces
robustus_ORX45454.1:0.0000023536,Anaeromyces
robustus_ORX82727.1:0.0000021728)/1:0.2860225296)/1:1.8368837421,((Spizellomyces
punctatus DAOM BR117_KND03168.1:0.0940479561,Spizellomyces sp.

'palustris'_TPX58002.1:0.0803327265)/1:1.1416443505,Powellomyces
hirtus_TPX56012.1:1.4264094860)/1:0.3407272501)/0.996:0.1755999072,(Synchytrium
microbalum_TPX32694.1:1.0954570625,Synchytrium
endobioticum_TPX52037.1:1.5137118965)/1:0.6711958152)/1:0.1746228547)/
1:0.2131305965)/0.995:0.0742772743,((Entomophthora
muscae_KAF7743295.1:0.0221328282,Entomophthora
muscae_KAF7755177.1:0.0295302299)/1:2.5444457958,Conidiobolus coronatus NRRL
28638_KXN70736.1:2.3356070966)/1:1.0324138365)/1:0.0723798907,(Drosophila
melanogaster_NP_611925.2:4.5617677922,((((Zancudomyces
culisetae_OMH84186.1:1.9088318752,((Furculomyces
boomerangus_PVU88831.1:0.0000025559,(Furculomyces
boomerangus_PVU91246.1:0.0017545137,Smittium
angustum_PWA01933.1:0.0000020845)/0.815:0.0037024454)/1:1.1901377147,(Smittium
simulii_PVU89671.1:1.3325607586,Smittium
megazygosporum_PVV01722.1:1.2704542898)/0.999:0.1946871946)/1:0.4394385071)/
1:0.6245599361,(Linderina pennispora_ORX72199.1:0.6123843794,Coemansia reversa
NRRL 1564_PIA19457.1:1.1783797778)/1:1.4664725333)/0.777:0.1065084122,Dimargaris
cristalligena_RKP38672.1:2.0875341454)/0.824:0.1177034724,(Rhizoclosmatium
globosum_ORY33242.1:1.6166311932,Chytriomyces
confervae_TPX69853.1:1.2835737894)/1:0.8551075769)/0.905:0.0969223159)/
0.963:0.0688194209)/1:0.1189564805,(Batrachochytrium dendrobatidis
JAM81_EGF83379.1:2.0857483748,Blyttiomyces
helicus_RKO93396.1:1.3896925189)/0.994:0.2226030685)/1:0.1544771548,((Rozella
allomycis CSF55_EPZ31853.1:2.2713971889,Paramicrosporidium
saccamoebae_PJF18267.1:2.3807023264)/0.464:0.1430174630,(Amphiamblys sp.
WSBS2006_OIR57862.1:2.5628301454,Salpingoeca
rosetta_XP_004991755.1:1.9135587463)/0.991:0.2813315783)/1:0.2450785200)/
0.886:0.0528981288)/0.884:0.0705083331,Capsaspora owczarzaki ATCC
30864_XP_004346647.2:1.9919255001)/0.999:0.1660102411,Amphimedon
queenslandica_A0A1X7VMB0:1.7268201697)/1:0.3599632160,Pomacea
canaliculata_PVD21562.1:1.9571493926)/1:1.2339406759);

****
****
RAD51
****
****
(Homo sapiens_NP_002866.2:0.0069410447,Mus musculus_NP_035364.1:0.0220148822,
((((((((((((((((Anncaliia algerae PRA339_KCZ81425.1:0.1762366775,Tubulinosema
ratisbonensis_RVD93403.1:0.2616658745)/1:0.3992976686,((Nosema apis BRL
01_EQB60630.1:0.7047852642,Nosema
ceranae_KKO75157.1:0.5030940765)/0.866:0.1630916323,Nosema
granulosis_KAF9761325.1:0.4048870347)/1:0.2547299190)/0.956:0.1359597340,
(Hamiltosporidium magnivora_TBT99232.1:0.1074699126,Hamiltosporidium
tvaerminnensis_TBU10564.1:0.0112909616)/1:0.6966752858)/0.961:0.0871111854,Thelo
hania contejeani_KAF7684362.1:0.4647123022)/1:0.1551910465,(((Vavraia culicis
subsp. floridensis_ELA46012.1:0.0827671484,Trachipleistophora
hominis_ELQ75893.1:0.0757007271)/1:0.5095778349,Cucumispora
dikerogammari_KAF7702697.1:0.9740967553)/1:0.2584588413,(Dictyocoela
roeselum_KAG0419893.1:0.2111205462,Dictyocoela
muelleri_KAG0440847.1:0.1524506855)/1:0.4171970845)/0.879:0.0794173065)/
1:0.3196758168,(((Mitosporidium daphniae_KGG51533.1:0.7813352003,Amphiamblys sp.
WSBS2006_OIR58962.1:0.7879085016)/0.847:0.1708742433,Paramicrosporidium
saccamoebae_PJF18425.1:0.5182914483)/0.813:0.0646748405,Salpingoeca
rosetta_XP_004994547.1:2.5264428440)/0.775:0.0645844968)/1:0.1974199887,Rozella
allomycis CSF55_EPZ30915.1:0.8208303967)/1:0.1625046634,((((((((((Apophysomyces
sp. BC1021_KAG0180202.1:0.1300165737,Apophysomyces
ossiformis_KAF7722115.1:0.1584191136)/1:0.2165347119,((((((Mucor
plumbeus_KAG2208809.1:0.1938102707,Parasitella
parasitica_CEP12943.1:0.0856485347)/0.807:0.0114260633,((((Rhizopus
azygosporus_RCH86309.1:0.0430670592,Rhizopus
azygosporus_RCH91481.1:0.0207681009)/1:0.1636639214,Rhizopus delemar RA 99-
880_EIE79168.1:0.1847990178)/1:0.1107421993,Thamnidium
elegans_KAG2230282.1:0.1735767178)/0.661:0.0843763400,Mucor

saturninus_KAG2210020.1:0.2420278458)/0.913:0.0480586338)/1:0.0469566448,Mucor lusitanicus_KAF1807531.1:0.1526317448)/0.743:0.0187770419,Mucor ambiguus_GAN03111.1:0.0280007430)/0.966:0.0747582365,Choanephora cucurbitarum_OBZ87403.1:0.2608185544)/1:0.3839221149,Mucor circinatus_KAG2226773.1:0.3596140069)/0.997:0.0934969166)/0.908:0.0648144258,Absidia glauca_SAM05585.1:0.4495385545)/0.997:0.1059113499,Phycomyces blakesleeanus NRRL 1555_OAD78152.1:0.5774250756)/0.994:0.1430286799,(Umbelopsis vinacea_KAG2173423.1:0.4864392095,Umbelopsis isabellina_KAG2178816.1:0.2767377831)/1:0.3435829003)/0.89:0.1072657453,Jimgerdemannia flammicorona_RUO96455.1:0.6656000739)/0.964:0.1271386190,Bifiguratus adelaidae_OZJ03230.1:0.3110326652)/1:0.1222648385,((((Mortierella sp. AM989_KAF9102260.1:0.0380853346,(Haplosporangium sp. Z 27_KAF9202520.1:0.0386571991,Entomortierella lignicola_KAF8984346.1:0.0466642284)/1:0.1036939849)/0.978:0.0169283662, (Entomortierella beljakovae_KAF9434156.1:0.1076502346,Lunasporangiospora selenospora_KAF9579934.1:0.2969195394)/1:0.0375959554)/0.525:0.0089574810, ((((((((((Mortierella sp. 14UC_KAF9122731.1:0.0644265318,(Linnemannia exigua_KAG0278269.1:0.0422623482,Mortierella sp. AD032_KAG0375785.1:0.1023722881)/1:0.0600464506)/0.629:0.0116648986, ((((Linnemannia schmuckeri_KAF9143406.1:0.0791552559,Haplosporangium gracile_KAF8936030.1:0.0288758937)/1:0.0231981207,((Mortierella sp. GBA39_KAF9143794.1:0.0141392729,Linnemannia hyalina_KAG9067234.1:0.0746142608)/0:0.0000023531,Mortierella hygrophila_KAF9551034.1:0.0140409585)/1:0.0521579952)/0.988:0.0231828695,Linnemannia gamsii_KAG0286684.1:0.0432944205)/1:0.0765904486,Linnemannia zychae_KAF9908627.1:0.0355400780)/0.938:0.0091833925)/1:0.0753866689, ((Mortierella sp. GBA35_KAF9093132.1:0.0000020008,Mortierella sp. NVP41_KAG0213509.1:0.0066644871)/0.966:0.0068902547,Mortierella sp. AD031_KAF9095337.1:0.0219621039)/1:0.0544890900)/1:0.0535856385, (((((Haplosporangium bisporale_KAF8953166.1:0.0205494452,Podila verticillata NRRL 6337_KFH73305.1:0.0000027823)/1:0.0836424276,Podila clonocystis_KAG0017634.1:0.0521018709)/0.126:0.0000024393,Podila horticola_KAF9312439.1:0.0452031457)/0.589:0.0107269886,(Podila epicladia_KAG0092669.1:0.1215979455,Podila minutissima_KAG0356504.1:0.0067078561)/0.128:0.0000023763)/0.927:0.0996185421, (Podila epigama_KAF9422179.1:0.3430698352,Podila humilis_KAG0345413.1:0.4056293574)/0.999:0.0952862743)/1:0.1452987548,Gryganskiella cystojenkinii_KAG0043215.1:0.0963333892)/0.994:0.1023604290)/0.997:0.0733087984, Dissophora globulifera_KAG0326260.1:0.1077473795)/0.863:0.0241126765, ((Haplosporangium sp. Z 11_KAF9179326.1:0.0000025865,Haplosporangium sp. Z 767_KAF9181034.1:0.0000023853)/0.999:0.0395881693,Mortierella polycephala_KAG0264970.1:0.0000023954)/1:0.1039214715)/0.663:0.0241287563,Mortierella polycephala_KAG0264969.1:0.2975779700)/0.986:0.0756319128,((Modicella reniformis_KAF9998418.1:0.0296218695,Mortierella sp. GBA43_KAG0244986.1:0.0710652896)/0.999:0.0395351291,Mortierella sp. NVP85_KAF9365634.1:0.1470606075)/1:0.0437368516)/0.857:0.0211545254,(Mortierella antarctica_KAF9981586.1:0.1239860963,Mortierella sp. GBA30_KAG0210733.1:0.1000442589)/1:0.0402758853)/0.901:0.0320342234,(Dissophora ornata_KAF8932022.1:0.1355112525,Gamsiella multidivaricata_KAG0364439.1:0.0890000216)/0.998:0.0245276838)/1:0.1202662780, ((Mortierella sp. AD010_KAF9171848.1:0.0327467623,Mortierella sp. AD011_KAF9400200.1:0.0000027320)/1:0.0345875248,Entomortierella chlamydospora_KAF9996095.1:0.0000026875)/0.862:0.0270465699)/0.995:0.0507186406, Mortierella sp. AD094_KAF9350711.1:0.0874378439)/0.93:0.0211256726)/1:0.4746699424, ((((Rhizophagus clarus_GES92629.1:0.0431367186,Rhizophagus irregularis DAOM 181602=DAOM 197198_POG72131.1:0.0798281014)/0.999:0.0473525568,Glomus cerebriforme_RIA97061.1:0.0222116009)/1:0.1731247026,Diversispora epigaea_RHZ75300.1:0.1852904934)/0.998:0.0546227097,Gigaspora margarita_KAF0488439.1:0.0598891799)/1:0.1590721566,Geosiphon pyriformis_KAG9306089.1:0.2053692141)/1:0.2773700482)/0.966:0.1015947787)/1:0.1273997295,(((((((Sclerotinia sclerotiorum 1980 UF-70_EDN91591.1:0.3675362703,Blumeria graminis f. sp.

triticale_CAD6499572.1:0.6044670190)/1:0.7409000093,(Aspergillus nidulans FGSC
A4_EAA61925.1:0.1896784780,Coccidioides immitis
RS_EAS29013.3:0.1204959925)/1:0.2702734526)/0.997:0.1390631163,Tuber
melanosporum_CAZ85163.1:0.3586732967)/1:0.2577742722,(Orbilia oligospora ATCC
24927_EGX44320.1:0.0943787008,Drechslerella
brochopaga_KAF3924209.1:0.0919216415)/1:0.3672845281)/1:0.3332702801,(Candida
albicans SC5314_AOW26871.1:0.4815273765,Saccharomyces cerevisiae
S288C_DAA07842.1:0.6752536258)/1:0.3910698978)/1:0.2590900239,
((((Schizosaccharomyces japonicus
yFS275_EEB07184.2:0.0821978723,Schizosaccharomyces
pombe_CAA17024.1:0.2306195196)/1:0.4334793848,Pneumocystis carinii
B80_KTW29839.1:0.5295097593)/1:0.1462705217,Neolecta irregularis DAH-
3_OLL24075.1:0.4957356617)/1:0.1758469327,Puccinia graminis f. sp. tritici CRL
75-36-700-3_EFP89931.1:0.9488810049)/0.951:0.0591801156)/1:0.1664013765,
((Cryptococcus neoformans var. grubii H99_AFR98404.1:0.8549410257,((Coprinopsis
cinerea okayama7#130_EAU92763.2:0.0946139961,Amanita muscaria Koide
BX008_KIL71707.1:0.9428703819)/1:0.2681214889,Rhizoctonia
solani_QRW24868.1:0.5835513265)/1:0.4396441928)/1:0.1893079724,Wallemia
ichthyophaga
EXF-994_EOR01152.1:1.0210594018)/0.993:0.1088061280)/1:0.3347631754)/
1:0.1254113341,((((((Spizellomyces punctatus DAOM
BR117_KNC98933.1:0.0000025026,Spizellomyces sp.
'palustris'_TPX61015.1:0.0231482824)/1:0.0634063725,Powellomyces
hirtus_TPX61525.1:0.2506604442)/0.991:0.1331288276,Blyttiomyces
helicus_RKO82769.1:0.6127530084)/0.969:0.1974370165,Blyttiomyces
helicus_RKO85343.1:0.1672603689)/0.989:0.1939106423,(Chytriomyces
confervae_TPX55570.1:0.0000021469,Chytriomyces
confervae_TPX61770.1:0.0000025951)/1:0.8181487990)/1:0.2303331311,
(Neocallimastix sp. JGI-2020a_KAG4092591.1:0.0814341488,Piromyces
finnis_ORX47301.1:0.0756598232)/1:0.5725107068)/1:0.1246115104)/1:0.1288393924,
(((Smittium mucronatum_OLY80664.1:0.0804744658,Smittium
culicis_OMJ21466.1:0.1499904228)/1:0.1697799101,((Zancudomyces
culisetae_OMH83487.1:0.5591740929,Smittium
simulii_PVU90330.1:0.1853700286)/0.757:0.0758875396,Smittium
megazygosporum_PVV05375.1:0.3429379667)/1:0.1454707978)/1:0.1731724192,Furculomy
ces boomerangus_PVU96364.1:0.2419552080)/1:0.6464099720)/0.757:0.0647900480)/
1:0.1460130820,Sphaeroforma arctica
JP610_XP_014155422.1:1.1890106490)/0.248:0.1252337460,Capsaspora owczarzaki ATCC
30864_XP_004364475.2:0.6505896210)/1:0.9930677082,Olpidium
bornovanus_KAG5458076.1:1.4694596621)/0.999:0.1876775396,Fonticula
alba_XP_009494877.1:1.9272576698)/0.227:0.0124633184,(Drosophila
melanogaster_NP_524583.1:0.9974328661,Rozella allomycis
CSF55_EPZ33448.1:1.2336586460)/0.921:0.0936663289)/1:0.0650596529,(((((((Nosema
granulosis_KAF9764107.1:0.0358618480,(Nosema bombycis
CQ1_EOB11895.1:0.0042045449,Nosema bombycis
CQ1_EOB11223.1:0.1618598588)/1:0.0762224944)/1:0.2285136103,Nosema
ceranae_KKO74715.1:0.1305056807)/0.821:0.0688255672,Nosema apis BRL
01_EQB60701.1:0.0832890296)/1:0.2567921955,((Encephalitozoon hellem ATCC
50504_AFM99344.1:0.0165115133,Encephalitozoon intestinalis ATCC
50506_ADM12569.1:0.0147824777)/1:0.0794656217,Ordospora colligata
OC4_KHN68850.1:0.2181913049)/1:0.1689836356)/1:0.2541988581,((((Enterocytozoon
bieneusi H348_EDQ31186.1:0.2305598164,Enterospora
canceri_ORD94454.1:0.2787902068)/0.416:0.0243668391,Enterocytozoon
hepatopenaei_OQS55103.1:0.3838105005)/1:0.1485373216,Hepatospora
eriocheir_ORD96532.1:0.3755821528)/0.997:0.0982851160,Vittaforma corneae ATCC
50505_ELA42927.1:0.4411857150)/1:0.1863545940)/1:0.1627172257,(((((Dictyocoela
roeselum_KAG0420198.1:0.0732590109,Dictyocoela
muelleri_KAG0438868.1:0.0413453152)/1:0.3105155539,(((Hamiltosporidium
tvaerminnensis_TBU10985.1:0.0000029753,Hamiltosporidium
magnivora_TBU00721.1:0.0088877419)/0.129:0.0000024700,Hamiltosporidium
magnivora_TBU02671.1:0.0000028829)/1:0.3117193457,(Edhazardia aedis USNM
41457_EJW05219.1:0.5643841787,(Anncaliia algerae
PRA339_KCZ79458.1:0.1208917818,Tubulinosema
ratisbonensis_RVD90575.1:0.1267149755)/1:0.1587896083)/0.75:0.0538409259)/

0.307:0.0327904037)/1:0.0922
389454,Thelohania contejeani_KAF7684466.1:0.3388138695)/1:0.0567516526,((Vavraia culicis subsp. floridensis_ELA47458.1:0.0107245004,Trachipleistophora hominis_ELQ75018.1:0.0319196533)/1:0.1783335750,Pseudoloma neurophilia_KRH93297.1:0.2814760718)/1:0.2369954199)/0.97:0.0981456932,Cucumispora dikerogammari_KAF7696489.1:1.0594070612)/0.999:0.1011859817)/1:0.1306036629,
((Nematocida sp. 1 ERTm6_KFG27164.1:0.1531954875,(Nematocida sp. ERTm5_OAG30624.1:0.0000025376,Nematocida parisii ERTm1_EIJ94865.1:0.0072947396)/1:0.1071686486)/1:0.9615297119,Nematocida displodere_OAG31748.1:0.4092190976)/1:0.3440197324)/0.912:0.1263759984,Amphiamblys sp.
WSBS2006_OIR57801.1:1.1289152077)/0.992:0.1672332729)/1:0.1178112058,Paramicrosporidium saccamoebae_PJF17429.1:0.4671941926)/1:0.1474965991,((((((Glomus cerebriforme_RIA92998.1:0.0895239974,(Rhizophagus clarus_GET04201.1:0.0560547307,Rhizophagus irregularis DAOM 181602=DAOM 197198_POG70495.1:0.1139577091)/1:0.1017738190)/1:0.3545845203,((Gigaspora margarita_KAF0546069.1:0.0000024156,Gigaspora margarita_KAF0546070.1:0.0000023034)/1:0.6075724579,((Diversispora epigaea_RHZ57132.1:0.7115037104,(Gigaspora margarita_KAF0504898.1:0.0000024668,Gigaspora margarita_KAF0504899.1:0.0000020618)/1:0.3021270757)/0.248:0.0253347530,Diversispora epigaea_RHZ61908.1:0.2353370680)/0.907:0.0391165718)/1:0.3255468759)/
1:0.3354589097,Geosiphon pyriformis_KAG9304939.1:1.0218363091)/0.998:0.0990784696,(((((((Parasitella parasitica_CEP12164.1:0.1979170552,(Mucor lusitanicus_KAF1807511.1:0.0374775807,Mucor ambiguus_GAN04987.1:0.0804125905)/1:0.0789610236)/1:0.2519944214,Choanephora cucurbitarum_OBZ80962.1:0.3460808179)/0.726:0.1015474137,Mucor saturninus_KAG2204041.1:0.6350424056)/1:0.1647196247,(Rhizopus delemar RA 99-880_EIE79639.1:0.2267858997,(Rhizopus azygosporus_RCH86815.1:0.0193650474,Rhizopus azygosporus_RCH95708.1:0.0419318310)/1:0.2280309510)/1:0.2022991166)/
1:0.3587599798,(((Apophysomyces sp. BC1015_KAG0164926.1:0.0000020940,Apophysomyces sp. BC1021_KAG0172855.1:0.0000020416)/1:0.4251743696,Apophysomyces ossiformis_KAF7730675.1:0.1989472296)/1:0.3158436714,Mucor circinatus_KAG2224305.1:0.5990773890)/1:0.2527741949)/1:0.2922801786,
((Umbelopsis isabellina_KAG2184514.1:0.2197203553,Umbelopsis vinacea_KAG2188594.1:0.1005536776)/1:0.1942222624,((((Choanephora cucurbitarum_OBZ86032.1:0.2123151551,(Mucor saturninus_KAG2207547.1:0.0431950146,Thamnidium elegans_KAG2236116.1:0.0871617163)/1:0.1153364788)/0.897:0.0396138166,(((Mucor ambiguus_GAN03643.1:0.0000022191,Mucor lusitanicus_KAF1801726.1:0.0000022170)/0.999:0.0315970634,Mucor plumbeus_KAG2212486.1:0.0406309458)/0.995:0.0213785432,Parasitella parasitica_CEP18855.1:0.1175746972)/1:0.1691104968)/0.963:0.0292096389,
(((Absidia glauca_SAM01541.1:0.2320157307,(Phycomyces blakesleeanus NRRL 1555_OAD80259.1:0.2497629116,Syncephalastrum racemosum_ORZ01446.1:0.2235706602)/0.956:0.1290160972)/0.987:0.0650938905,Mucor circinatus_KAG2216929.1:0.1975159158)/0.962:0.0230655893,(Apophysomyces ossiformis_KAF7732322.1:0.0000022922,((Apophysomyces sp. BC1015_KAG0174997.1:0.0000026873,Apophysomyces sp. BC1034_KAG0193750.1:0.0000020900)/1:0.0081822614,Apophysomyces sp. BC1021_KAG0182944.1:0.0000022659)/1:0.1734463161)/1:0.3584750511)/
0.996:0.0639470835)/0.963:0.1091488036,(Rhizopus delemar RA 99-880_EIE81616.1:0.4579009793,(Rhizopus delemar RA 99-880_EIE85356.1:0.0760356726,Rhizopus azygosporus_RCI01612.1:0.1139826217)/0.334:0.0658668670)/1:0.3792875878)/
1:0.2578991724)/0.863:0.0651798366)/1:0.1661441374,((Bifiguratus adelaidae_OZJ06257.1:0.1499420938,Endogone sp. FLAS-F59071_RUS17608.1:0.2499727997)/0.983:0.0399005468,Jimgerdemannia flammicorona_RUP51864.1:0.2982576143)/1:0.1180554191)/1:0.1121098177)/
1:0.0687842531,(((((((((((Mortierella sp. AD031_KAF9096695.1:0.0000028728,Mortierella sp.

GBA35_KAF9099114.1:0.0071183609)/1:0.1389930760,(((Mortierella sp.
14UC_KAF9114365.1:0.0395872716,Linnemannia
zychae_KAF9906770.1:0.0273926344)/1:0.2214593289,(Linnemannia
exigua_KAG0271116.1:0.0384689001,Mortierella sp.
AD032_KAG0378901.1:0.0124855633)/1:0.0878406339)/1:0.1275405792,((((Linnemannia
schmuckeri_KAF9150467.1:0.0000029781,Haplosporangium
gracile_KAF8945743.1:0.0141472202)/1:0.0142194674,Mortierella sp.
GBA39_KAF9136717.1:0.0000025425)/0.125:0.0000029584,(Mortierella
hygrophila_KAF9541433.1:0.0072658220,Linnemannia
hyalina_KAG9067849.1:0.0140920568)/0.994:0.0071083334)/0.999:0.0159998812,Linnem
annia gamsii_KAG0283216.1:0.0492004018)/1:0.0488216738)/0.504:0.0163159728)/
0.13:0.0000026881,Gamsiella
multidivaricata_KAG0353524.1:0.0752258973)/0.492:0.0136534893,(Dissophora
ornata_KAF8926721.1:0.1534500787,Mortierella
antarctica_KAF9982538.1:0.1815748739)/0.991:0.0373352049)/0.693:0.0096835389,
((Haplosporangium sp. Z 11_KAF9180486.1:0.0000021401,Mortierella
polycephala_KAG0252268.1:0.0357784815)/1:0.0242317739,Mortierella sp.
GBA30_KAG0200669.1:0.0663650674)/1:0.0241303035)/1:0.0153308374,(Mortierella sp.
AM989_KAF9113875.1:0.0519755707,(Lunasporangiospora
selenospora_KAF9579443.1:0.1844907396,Gryganskiella
cystojenkinii_KAG0047840.1:0.1180102859)/0.916:0.0365995761)/0.967:0.0082291327)
/1:0.0150741697,((Entomortierella
chlamydospora_KAG0000811.1:0.0140405545,Mortierella sp.
AD094_KAF9354871.1:0.0142144831)/1:0.0292038042,Entomortierella
beljakovae_KAF9437915.1:0.0593791215)/0.999:0.0210269330)/0.922:0.0075018098,Ent
omortierella lignicola_KAF8983088.1:0.0284085613)/1:0.0596372840,(((Mortierella
sp. NVP85_KAF9363379.1:0.3017190271,Mortierella sp.
GBA43_KAG0237784.1:0.3420879583)/0.961:0.0875943024,Modicella
reniformis_KAF9987694.1:0.3702092443)/1:0.2186077436,Dissophora
globulifera_KAG0312046.1:0.1491953668)/0.989:0.0749352842)/1:0.1373248567,
((((((Haplosporangium bisporale_KAF8947298.1:0.0000027224,Podila verticillata
NRRL 6337_KFH66657.1:0.0000025676)/0.131:0.0000021028,Haplosporangium
bisporale_KAF8923440.1:0.0000027214)/1:0.0649700471,Podila
clonocystis_KAG0011888.1:0.0000027714)/1:0.0072486617,(Podila
horticola_KAF9308213.1:0.0070541605,Podila
epicladia_KAG0100701.1:0.0000024901)/0.96:0.0069110079)/0.997:0.0204783115,Podil
a minutissima_KAG0361050.1:0.0445502418)/1:0.0453556037,Podila
humilis_KAG0348810.1:0.0593741259)/1:0.0420805140,Podila
epigama_KAF9426310.1:0.1503364417)/1:0.1134782676)/0.841:0.0587448183,
(Actinomortierella wolfii_KAG0242569.1:0.0183115058,Actinomortierella
ambigua_KAG0257137.1:0.0692055423)/1:0.1544420093)/1:0.2630660440,Gonapodya
prolifera JEL478_KXS09704.1:0.6184171524)/0.476:0.0188971768,(((Malassezia
globosa CBS 7966_EDP43515.1:0.5012340444,Ustilago maydis
521_KIS68723.1:0.3122444983)/1:0.2015129064,(Wallemia ichthyophaga EXF-
994_EOQ99772.1:0.6819903046,(Cryptococcus neoformans var. grubii
H99_AFR92850.1:0.3421018412,((((Coprinopsis cinerea
okayama7#130_EAU93170.1:0.1703583612,Amanita muscaria Koide
BX008_KIL70899.1:0.2960450855)/1:0.2100022912,Rhizoctonia
solani_QRW18941.1:0.4825553325)/0.97:0.1203774049,Puccinia graminis f. sp.
tritici CRL 75-36-700-3_EFP88202.1:1.1093693579)/0.999:0.1557791213,Nosema
bombycis CQ1_EOB11224.1:1.4628944742)/0.999:0.0969831154)/0.996:0.1557758753)/
0.913:0.0901967048)/1:0.1263891016,(((((((Aspergillus nidulans FGSC
A4_EAA65830.1:0.1177725596,Exophiala dermatitidis
NIH/UT8656_EHY55857.1:0.4051529979)/0.974:0.0486779637,(((Blumeria graminis f.
sp. triticale_CAD6504638.1:0.0833741120,(Neurospora crassa
OR74A_EAA35890.1:0.1103519993,Verticillium dahliae
VdLs.17_EGY18462.1:0.1515762345)/1:0.0609128426)/0.97:0.0253746601,Sclerotinia
sclerotiorum 1980 UF-70_EDO04299.1:0.0897946956)/1:0.0868025805,Bacidia
gigantensis_KAG8533257.1:0.2789116856)/0.915:0.0346954726)/0.424:0.0373875813,Co
ccidioides immitis RS_EAS27743.2:0.1956384245)/1:0.1990263160,Tuber
melanosporum_CAZ80285.1:0.4164320907)/0.959:0.0517384092,((Orbilia oligospora
ATCC 24927_EGX53310.1:0.0795442973,Drechslerella
brochopaga_KAF3909803.1:0.1505810293)/0.865:0.0124713464,Arthrobotrys
entomopaga_KAF3929349.1:0.0695520264)/1:0.1894065802)/1:0.3702445261,

(Pneumocystis carinii B80_KTW25774.1:0.3334332392,Neolecta irregularis DAH-3_OLL26693.1:0.3512379271)/0.519:0.0269699940)/1:0.1195243192,(Schizosaccharomyces pombe_CAB90141.1:0.1586686369,Schizosaccharomyces japonicus yFS275_EEB06365.1:0.1781867111)/1:0.3032335755)/0.888:0.0520326378,((Yarrowia lipolytica CLIB122_CAG78267.1:0.5639963878,Candida albicans SC5314_AOW30970.1:0.3847241314)/0.931:0.0752277621,Saccharomyces cerevisiae S288C_DAA07757.1:0.7284477648)/1:0.2371819126)/1:0.1095890783)/1:0.0945014538)/0.998:0.0250984915)/0.974:0.0364684143,(((Spizellomyces punctatus DAOM BR117_KND00769.1:0.0343536836,Spizellomyces sp. 'palustris'_TPX72343.1:0.0268109836)/1:0.1349227520,Powellomyces hirtus_TPX55239.1:0.3575701720)/1:0.2099951176,Chytriomyces confervae_TPX77094.1:0.6139245186)/1:0.1079110040)/1:0.0635929991,(((Neocallimastix sp. JGI-2020a_KAG4106608.1:0.0153345178,Piromyces finnis_ORX59322.1:0.0068339725)/0.664:0.0169981616,Neocallimastix sp. JGI-2020a_KAG4108015.1:0.0595499272)/1:0.2380791700,(Synchytrium microbalum_TPX30879.1:0.0816522135,Synchytrium endobioticum_TPX50643.1:0.0592898108)/1:0.2466857938)/0.981:0.0379274621)/0.82:0.0168579069)/0.395:0.0188056004,(((Allomyces macrogynus ATCC 38327_KNE59636.1:0.0084486351,Allomyces macrogynus ATCC 38327_KNE61320.1:0.0071781622)/1:0.2341155363,((Allomyces macrogynus ATCC 38327_KNE58002.1:0.0000027135,Allomyces macrogynus ATCC 38327_KNE59184.1:0.1565931337)/1:0.5093206222,Catenaria anguillulae PL171_ORZ31519.1:0.3030292123)/0.446:0.0672591158)/1:0.2643457524,Mitosporidium daphniae_KGG51257.1:0.9125193029)/0.848:0.0648581865)/0.983:0.0791530437,(((Smittium mucronatum_OLY79567.1:0.2049437536,Smittium culicis_OMJ08494.1:0.0156393439)/0.999:0.0989984207,(Zancudomyces culisetae_OMH79472.1:0.0000024746,Zancudomyces culisetae_OMH84649.1:0.0000020684)/1:0.4779463242)/0.926:0.0383243105,(Smittium megazygosporum_PVV04488.1:0.0000021513,Smittium megazygosporum_PVV04494.1:0.0000022847)/1:0.2226262398)/1:0.4055372701)/1:0.3366336293);

****
FANCU
****

(Mus musculus_NP_065595.2:0.1814647072,((Drosophila melanogaster_AAF48852.1:3.3520140751,(((((Coccidioides immitis RS_EAS34619.3:1.0613725342,Zymoseptoria tritici IPO323_EGP92510.1:1.0096480970)/1:0.4825265159,Orbilia oligospora ATCC 24927_EGX51736.1:1.1675353598)/0.993:0.2518740628,(Spizellomyces sp. 'palustris'_TPX70254.1:2.1167021261,(((Capsaspora owczarzaki ATCC 30864_XP_004363926.1:0.6256156545,Sphaeroforma arctica JP610_XP_014157974.1:0.7935598465)/0.986:0.2698077862,Salpingoeca rosetta_XP_004998933.1:0.5650831198)/1:1.1434157489,Fonticula alba_XP_009494159.1:1.6643382249)/1:0.7412036521)/0.984:0.2232927877)/0.844:0.1462243768,((Coprinopsis cinerea okayama7#130_EAU93078.1:1.2637386946,((((Postia placenta Mad-698-R_EED80433.1:0.0186530379,Postia placenta Mad-698-R_EED82378.1:0.0157953732)/1:0.6755732825,Amanita muscaria Koide BX008_KIL70722.1:1.0076014911)/0.648:0.0828333690,Serpula lacrymans var. lacrymans S7.9_EGO20492.1:1.2474851512)/1:0.2541873391,(Ramaria rubella_KAF8591892.1:0.6985560263,(Rhizoctonia solani_QRW24265.1:0.0000029803,Rhizoctonia solani_QRW25003.1:0.0057690938)/1:1.2288457946)/0.987:0.1961877165)/0.966:0.1924956826)/1:0.4803297906,Pneumocystis carinii B80_KTW28146.1:1.6768440919)/0.538:0.1643528975)/0.919:0.2225310777,((((Gigaspora margarita_KAF0505769.1:0.0137994648,Gigaspora rosea_RIB18734.1:0.0292379946)/1:0.5390287424,Glomus cerebriforme_RIA94590.1:0.7014155978)/1:0.4810457677,(Podila epigama_KAF9426470.1:1.5914501739,Umbelopsis vinacea_KAG2181576.1:1.6539457902)/0.88:0.2676308404)/1:0.2995565765,(Entomophthora muscae_KAF7755001.1:0.0093102320,Entomophthora muscae_KAF7756708.1:0.0000024222)/1:1.8827201282)/0.658:0.0843813304)/

0.995:0.3952407457)/0.88:0.3153454220,Dictyostelium discoideum_EAL62275.1:1.3949089771)/1:1.3278273417,Homo sapiens_NP_005422.1:0.1248131013);

****

FANCV

****

(Mus musculus_NP_082261.2:0.0246643377,((((((((((((((((Caenorhabditis elegans_CCD74133.1:1.7175051887,Batrachochytrium dendrobatidis JAM81_EGF82262.1:0.7543456494)/0.996:0.3940962618,(((((((Neocallimastix sp. JGI-2020a_KAG4086132.1:0.0000020828,Neocallimastix californiae_ORY74728.1:0.0000025712)/1:0.6361604781,Piromyces finnis_ORX59277.1:0.3270443978)/1:0.2199707914,(Neocallimastix sp. JGI-2020a_KAG4088559.1:0.0000028663,Neocallimastix sp. JGI-2020a_KAG4091183.1:0.0000021397)/1:0.0917469935)/0.54:0.0131036716,Piromyces finnis_ORX41952.1:0.1136341075)/0.995:0.0863012912,Anaeromyces robustus_ORX78819.1:0.1001378699)/1:0.4872656196,Monosiga brevicollis MX1_XP_001748095.1:1.7285379011)/0.787:0.1283383827,Gonapodya prolifera JEL478_KXS17755.1:0.7455256125)/0.699:0.0803600081)/1:0.2303348035,Thamnocephalis sphaerospora_RKP09537.1:0.4808126562)/0.654:0.0676188729,((((((Drosophila melanogaster_NP_649555.1:5.5712541969,Absidia repens_ORZ10880.1:0.2768464012)/0.932:0.3071292118,Hesseltinella vesiculosa_ORX54928.1:0.5150587242)/1:0.3517895574,Phycomyces blakesleeanus NRRL 1555_OAD80640.1:1.7003822625)/0.995:0.2697548928,(Umbelopsis isabellina_KAG2172044.1:0.1824534624,Umbelopsis vinacea_KAG2178466.1:0.1048442678)/1:0.5598771063)/1:0.3328003903,(Gigaspora margarita_KAF0482738.1:0.0000024292,Gigaspora rosea_RIB17192.1:0.0434481596)/1:0.6585522302)/0.936:0.1326731758,(Linderina pennispora_ORX66762.1:0.5229587599,Coemansia reversa NRRL 1564_PIA17123.1:0.2326291197)/1:0.5525122815)/0.881:0.0612920914)/0.998:0.1362097388,((((Saccharomyces cerevisiae_NP_012504.3:0.0000020937,Saccharomyces cerevisiae S288C_DAA08768.1:0.0000021014)/1:1.1598006683,(((((Blumeria graminis f. sp. triticale_CAD6500492.1:0.3579417405,(Neurospora crassa OR74A_EAA30805.3:0.1825546050,Trichoderma reesei QM6a_EGR44301.1:0.2588878987)/0.877:0.0386687420)/1:0.1807155292,(Tuber melanosporum_CAZ86428.1:0.2823333733,Orbilia oligospora ATCC 24927_EGX50711.1:0.4611218853)/1:0.1292225694)/0.544:0.0549737451,Coccidioides immitis RS_EAS34611.2:0.1680312287)/0.983:0.0836937336,Zymoseptoria tritici IPO323_EGP92551.1:0.2683432324)/1:0.3718045752,Yarrowia lipolytica CLIB122_CAG79505.1:0.6733662102)/0.439:0.1072887059)/0.828:0.0649227359,Pneumocystis carinii B80_KTW25853.1:0.4057208405)/0.999:0.1766834422,(Schizosaccharomyces pombe_CAA16846.1:0.4900132310,Schizosaccharomyces japonicus yFS275_EEB06287.1:0.2485676351)/1:0.3714752441)/1:0.3065360201)/0.999:0.1662535769,Wallemia ichthyophaga EXF-994_EOR01111.1:0.4640832037)/1:0.1667016505,(((((Coprinopsis cinerea okayama7#130_EAU93158.2:0.1731217796,Amanita muscaria Koide BX008_KIL70914.1:0.0604466623)/1:0.2620466533,Ramaria rubella_KAF8587313.1:0.3068526013)/0.966:0.1221551376,Rhizoctonia solani_QRW18480.1:0.8357197888)/1:0.2913895264,Dacryopinax primogenitus_EJU00496.1:0.4166439625)/1:0.3130261284,((Malassezia globosa CBS 7966_EDP43041.1:0.3837550325,Ustilago maydis 521_KIS68006.1:0.1900855675)/0.998:0.1810525546,Tilletiaria anomala UBC 951_KDN44518.1:0.5187300084)/1:0.5772694554)/0.871:0.1051392056)/0.817:0.1050470148,Cryptococcus neoformans var. grubii H99_AFR97843.1:0.6009851305)/1:0.5078648338,(Puccinia graminis f. sp. tritici CRL 75-36-700-3_EFP77287.2:0.4265807234,Melampsora larici-populina 98AG31_EGF98905.1:0.4883721100)/1:0.9079616392)/1:2.7474905834,((Dictyostelium discoideum_XP_640239.1:2.1240772745,(Dimargaris cristalligena_RKP39478.1:2.8585626307,Salpingoeca rosetta_XP_004991674.1:2.5950811635)/0.396:0.4221496581)/0.952:0.4010196924,Coemansia reversa NRRL 1564_PIA19360.1:2.2254717668)/0.31:0.0642084431)/0.944:0.2325992651,

(Entomophthora muscae_KAF7748258.1:1.9725614497,(((((((((((Haplosporangium gracile_KAF8937069.1:0.0705917767,Linnemannia schmuckeri_KAF9149069.1:0.0456106125)/1:0.0768265089,(((Mortierella sp. GBA39_KAF9146723.1:0.0354364013,Linnemannia hyalina_KAG9067401.1:0.0355713903)/0.717:0.0024772090,Mortierella hygrophila_KAF9550411.1:0.0504204563)/1:0.0424575413,Linnemannia elongata AG-77_OAQ35651.1:0.1060750131)/1:0.0826872990)/1:0.1572629360,((Mortierella sp. AD031_KAF9089580.1:0.0024160145,Mortierella sp. NVP41_KAG0208497.1:0.0000020625)/0.694:0.0021587472,Mortierella sp. GBA35_KAF9102020.1:0.0027044012)/1:0.4006691561)/1:0.0886158745,(Linnemannia exigua_KAG0279798.1:0.1029921168,Mortierella sp. AD032_KAG0381053.1:0.1324021478)/1:0.0844489156)/0.998:0.0371810780,Mortierella sp. 14UC_KAF9127235.1:0.1983678559)/1:0.1200096478,Linnemannia zychae_KAF9908961.1:0.1049610313)/1:0.1603846151,((((Haplosporangium bisporale_KAF8980421.1:0.0226814891,Podila verticillata NRRL 6337_KFH66166.1:0.0000021059)/1:0.0865648334,(Podila clonocystis_KAG0024522.1:0.1411631147,(Podila epicladia_KAG0099488.1:0.0500209191,Podila minutissima_KAG0350041.1:0.0887793824)/1:0.0638689136)/0.985:0.0441092363)/1:0.1237820220,Podila humilis_KAG0347013.1:0.4331824822)/1:0.3936621237,Podila epigama_KAF9430026.1:0.2187556542)/1:0.0679574193)/1:0.0609566134,((Entomortierella lignicola_KAF8961207.1:0.1298622662,Haplosporangium sp. Z 27_KAF9209550.1:0.0202088895)/1:0.0952126418,(((Mortierella sp. AD010_KAF9162264.1:0.0000022003,Mortierella sp. AD011_KAF9381938.1:0.0000025427)/1:0.0283633459,(Entomortierella beljakovae_KAF9436645.1:0.8254670799,Entomortierella chlamydospora_KAG0005271.1:0.0082580932)/0.839:0.0358890540)/1:0.1606014830,Dissophora globulifera_KAG0312591.1:0.6730665940)/0.971:0.0850834108)/0.993:0.0272896516)/1:0.1593539231,(Mortierella sp. GBA30_KAG0203037.1:0.4334694325,Lobosporangium transversale_ORZ15413.1:0.4759821047)/0.994:0.1207704419)/0.54:0.0296683823,Dissophora ornata_KAF8940991.1:0.3484528771)/0.972:0.0395296679,Gamsiella multidivaricata_KAG0369772.1:0.3999195018)/0.826:0.0511613082,(Modicella reniformis_KAF9963746.1:0.2492491201,Mortierella sp. GBA43_KAG0245552.1:0.2431716651)/0.997:0.1437987931)/1:1.5472734543)/0.749:0.2031008973)/0.931:0.1097940496,(Umbelopsis vinacea_KAG2176853.1:0.8121353040,Umbelopsis isabellina_KAG2184854.1:0.6236951519)/1:1.3879922403)/0.521:0.0410145036,(((((((((Blumeria graminis f. sp. triticale_CAD6499076.1:0.9655775731,Sclerotinia sclerotiorum 1980 UF-70_EDN98926.1:1.1530771533)/1:0.4557086780,(Neurospora crassa OR74A_EAA32701.1:0.3991412702,Trichoderma reesei QM6a_EGR51349.1:1.1041907846)/1:0.4701603948)/0.999:0.2208729718,(((Aspergillus nidulans FGSC A4_EAA64207.1:0.6609740415,Coccidioides immitis RS_EAS34951.3:0.8411810629)/1:0.3505792667,Exophiala dermatitidis NIH/UT8656_EHY54041.1:1.3351761671)/1:0.3965292203,Bacidia gigantensis_KAG8527522.1:1.0050681261)/0.902:0.2258938437)/0.985:0.2117060603,Zymoseptoria tritici IPO323_EGP84745.1:0.9014399789)/1:0.2420031991,(Orbilia oligospora ATCC 24927_EGX47145.1:0.3964179070,(Drechslerella brochopaga_KAF3908051.1:0.5243984171,Arthrobotrys entomopaga_KAF3912367.1:0.0756981325)/0.999:0.2418108117)/1:0.6813783450)/0.997:0.3113354684,Tuber melanosporum_CAZ85742.1:1.2907334913)/0.984:0.2831496428,Neolecta irregularis DAH-3_OLL25940.1:1.2634913093)/0.997:0.3580257416,Schizosaccharomyces pombe_CAL45666.1:2.5666765519)/0.992:0.3921933973,(Mitosporidium daphniae_KGG51268.1:4.6632320150,(Catenaria anguillulae PL171_ORZ29190.1:0.0173008556,Catenaria anguillulae PL171_ORZ31551.1:0.0325274329)/1:2.8557083667)/0.876:0.3963262540)/0.993:0.2005097653,((((Cryptococcus neoformans var. grubii H99_AFR98611.2:1.3926782186,Wallemia ichthyophaga EXF-994_EOR01647.1:1.5683070162)/0.995:0.4185718346,((Coprinopsis cinerea okayama7#130_EAU92778.2:0.8785279395,(Postia placenta Mad-698-R_EED79114.1:0.3376646070,((Serpula lacrymans var. lacrymans S7.9_EGO31180.1:0.2525795186,Ramaria

rubella_KAF8585840.1:0.7727317850)/0.966:0.1074048174,Amanita muscaria Koide BX008_KIL71692.1:0.3326667044)/1:0.1564012492)/0.702:0.0511164732)/ 1:0.2343828076,(Dacryopinax primogenitus_EJT99924.1:0.8034499911,Rhizoctonia solani_QRW24807.1:0.7731875551)/0.125:0.0000027645)/1:0.7185994110)/ 0.866:0.2046555936,(Tilletiaria anomala UBC 951_KDN51362.1:1.2956681303,Ustilago maydis 521_KIS70575.1:1.3916184164)/1:0.6131769191)/0.993:0.2790941183, ((Puccinia graminis f. sp. tritici CRL 75-36-700-3_EFP75114.2:0.6618288136,Melampsora larici-populina 98AG31_EGG05869.1:0.6493431683)/1:0.8091306191,Mixia osmundae IAM 14324_KEI40091.1:1.6726755514)/1:0.8997115038)/0.992:0.2527629394)/ 1:0.2413313958)/0.987:0.0766928378,Geosiphon pyriformis_KAG9301141.1:1.2684979786)/0.993:0.1484911859,((((((Spizellomyces punctatus DAOM BR117_KND00296.1:0.0000022012,(Spizellomyces punctatus DAOM BR117_KND00297.1:0.0000022981,Spizellomyces punctatus DAOM BR117_KND00298.1:0.0000023068)/0:0.0000020069)/0.965:0.0405376267,Spizellomyces sp. 'palustris'_TPX62013.1:0.0000020280)/0.999:0.2247927721,Powellomyces hirtus_TPX54800.1:0.6033951379)/1:0.3085748263,Blyttiomyces helicus_RKO90681.1:0.9441391228)/0.897:0.1264656813,((Rhizoclosmatium globosum_ORY38554.1:0.4243940704,Chytriomyces confervae_TPX73283.1:0.6881442174)/1:0.9084697135,Synchytrium endobioticum_TPX53213.1:2.6474729651)/1:0.7371926780)/1:0.5892601287, (Basidiobolus meristosporus CBS 931.73_ORY04811.1:0.6635092887,((Thamnocephalis sphaerospora_RKP08841.1:0.1750944097,Syncephalis pseudoplumigaleata_RKP24512.1:0.3749197856)/1:0.4236903223,Piptocephalis cylindrospora_RKP14443.1:2.7261513411)/0.99:0.2803331187)/0.996:0.1766073609)/ 0.251:0.0278523835,((Bifiguratus adelaidae_OZJ04328.1:1.0736909495,Endogone sp. FLAS-F59071_RUS23393.1:1.0257824398)/0.715:0.2172064824,Jimgerdemannia flammicorona_RUP45457.1:1.5436283436)/0.964:0.2235588312)/1:0.2197377495)/ 0.754:0.0595900536,((((((((Mucor ambiguus_GAN02488.1:0.1444336726,Mucor lusitanicus_KAF1802373.1:0.0767713015)/1:0.2337790437,Parasitella parasitica_CEP19692.1:0.2736162163)/0.533:0.0434343383,Mucor plumbeus_KAG2189969.1:0.3230791477)/1:0.4045957530,Thamnidium elegans_KAG2237891.1:1.1192159685)/0.975:0.0954398931,Mucor saturninus_KAG2207725.1:0.4324757384)/0.895:0.1518751839,Choanephora cucurbitarum_OBZ82130.1:0.9804713352)/0.994:0.2036708976,(Rhizopus delemar RA 99-880_EIE80471.1:0.6345807586,(Rhizopus microsporus ATCC 52813_PHZ09320.1:0.0821925369,Rhizopus azygosporus_RCH89856.1:0.0720031367)/1:0.5469074328)/0.994:0.2157227311)/ 1:0.6252530203,(Mucor circinatus_KAG2224040.1:0.8369061736,Syncephalastrum racemosum_ORY93801.1:1.2520858726)/0.974:0.2100626686)/0.959:0.2580307039, (((Absidia glauca_SAM09680.1:0.7847368450,Hesseltinella vesiculosa_ORX50812.1:1.7867516934)/0.862:0.1697963294,Absidia repens_ORZ11070.1:0.4556782986)/1:0.7987369895,Phycomyces blakesleeanus NRRL 1555_OAD77667.1:0.8832072935)/0.694:0.1169441549)/1:1.2024281402)/ 1:0.2767956177,Capsaspora owczarzaki ATCC 30864_XP_004365102.2:1.5095583297)/0.3:0.0684016433,(((Neocallimastix sp. JGI-2020a_KAG4098279.1:0.0319010157,Neocallimastix californiae_ORY61079.1:0.0622284399)/1:0.3617050803,Piromyces finnis_ORX54236.1:0.3935237986)/0.89:0.1218568706,Anaeromyces robustus_ORX79150.1:0.3092401778)/1:2.0773712372)/1:1.2330517434,Homo sapiens_sp|Q9UI95|MD2L2_HUMAN:0.0000023142);

****

FANCW.treefile

****

(Dictyostelium discoideum_XP_646004.1:1.6566557520,((((((Mus musculus_NP_666330.2:0.2578181744,Homo sapiens_NP_060594.3:0.2155815710)/1:0.7921644069,((Drosophila melanogaster_NP_648919.1:1.4551477090,Monosiga brevicollis MX1_XP_001742568.1:2.7883709460)/0.673:0.2099033769,Paramicrosporidium saccamoebae_PJF17720.1:1.9729369991)/0.18:0.0249270471)/0.91:0.1596657712,Salpingoeca rosetta_XP_004987510.1:1.0902237009)/1:0.3287827712,(((((((((((((Mucor

ambiguus_GAN09022.1:0.1079157021,Mucor
lusitanicus_KAF1800737.1:0.1357052093)/1:0.3283021739,Mucor
plumbeus_KAG2198574.1:0.3449257480)/0.733:0.0589637150,Parasitella
parasitica_CEP09515.1:0.3403651665)/1:0.6504858285,Choanephora
cucurbitarum_OBZ87122.1:0.8806212812)/0.986:0.2090670597,((Rhizopus delemar RA
99-880_EIE92427.1:0.9955686847,(Rhizopus microsporus ATCC
52813_PHZ16113.1:0.0376553926,Rhizopus
azygosporus_RCH82500.1:0.0000020931)/1:0.3200020102)/1:0.6763490774,Thamnidium
elegans_KAG2235732.1:0.6162784611)/0.969:0.1992734441)/1:0.3871407162,Phycomyces
blakesleeanus NRRL 1555_OAD68195.1:1.1109960928)/1:0.1764283583,(((Absidia
glauca_SAM03726.1:0.5912418305,Absidia
repens_ORZ14244.1:0.4449053143)/0.951:0.1729540144,Hesseltinella
vesiculosa_ORX50462.1:0.9495332207)/1:0.6133837610,Apophysomyces sp.
BC1021_KAG0182375.1:0.9576972379)/0.528:0.0517596407)/1:0.1271936128,(Mucor
circinatus_KAG2222380.1:0.9035525566,Syncephalastrum
racemosum_ORY96619.1:1.0173142652)/1:0.3187930483)/0.864:0.1072619100,
(((((Parasitella parasitica_CEP10775.1:0.4310410540,Mucor
plumbeus_KAG2201716.1:0.2882691816)/1:0.1752063456,Mucor
lusitanicus_KAF1800787.1:0.4996379082)/1:0.4978902420,Choanephora
cucurbitarum_OBZ85250.1:0.9787662828)/0.998:0.2047369018,(Mucor
saturninus_KAG2205032.1:0.6585654015,Thamnidium
elegans_KAG2230875.1:0.4096333710)/1:0.4791841295)/1:0.2210814478,(Rhizopus
delemar RA 99-880_EIE79779.1:0.6149737910,(Rhizopus microsporus ATCC
52813_PHZ15348.1:0.1766179854,(Rhizopus
azygosporus_RCH88946.1:0.0222835055,Rhizopus
azygosporus_RCH93568.1:0.0528302937)/0.943:0.0366014675)/1:0.4098572782)/
1:0.4676820401)/1:0.3655080156,(Phycomyces blakesleeanus NRRL
1555_OAD69302.1:0.9452104317,(Hesseltinella
vesiculosa_ORX59830.1:1.0873659408,Absidia
repens_ORZ22559.1:0.8488883432)/1:0.5480665645)/0.899:0.1317451184)/
1:0.1443899348)/1:0.3064454768,(Umbelopsis
isabellina_KAG2171835.1:0.3352288528,Umbelopsis
vinacea_KAG2176687.1:0.4542757239)/1:0.6713065474)/1:0.2982730822,((Basidiobolus
meristosporus CBS 931.73_ORX89763.1:0.7569864715,((Jimgerdemannia
flammicorona_RUP43916.1:0.0000027084,Jimgerdemannia
flammicorona_RUP43917.1:0.0000023242)/1:0.3992127904,Endogone sp. FLAS-
F59071_RUS21578.1:0.3598497229)/1:0.5999670498)/0.976:0.1330523577,Bifiguratus
adelaidae_OZJ05717.1:1.5269897257)/0.88:0.1395502947)/0.349:0.0113874348,
(Thamnocephalis sphaerospora_RKP08613.1:1.2854913199,Dimargaris
cristalligena_RKP38993.1:1.2935379664)/1:0.2713126566)/0.999:0.0861581980,
(((((((((Dissophora ornata_KAF8932178.1:0.2484271168,Dissophora
globulifera_KAG0330405.1:0.2953296509)/1:0.0959379355,(((Mortierella sp.
AM989_KAF9116936.1:0.2101225650,((((Mortierella sp.
AD010_KAF9169308.1:0.0024930381,Mortierella sp.
AD011_KAF9402300.1:0.0010432434)/1:0.0513985010,Entomortierella
chlamydospora_KAG0000668.1:0.0627912456)/1:0.1522346533,Mortierella sp.
AD094_KAF9353857.1:0.0321140907)/1:0.0466159229,Haplosporangium sp. Z
27_KAF9209478.1:0.3644279954)/1:0.0536882463)/0.999:0.0628129850,Entomortierella
beljakovae_KAF9435361.1:0.4953138631)/1:0.0739752419,(Gamsiella
multidivaricata_KAG0354639.1:0.2771225846,Lobosporangium
transversale_ORZ21021.1:0.5943410504)/0.128:0.0000020080)/0.925:0.0311907945)/
1:0.0559636926,(((Mortierella sp. AD031_KAF9087147.1:0.0000021681,Mortierella
sp. NVP41_KAG0207459.1:0.0016591873)/1:0.1805567217,(((Mortierella sp.
14UC_KAF9122962.1:0.1018045113,Linnemannia
zychae_KAF9904515.1:0.1274771277)/1:0.0562997037,Mortierella sp.
AD032_KAG0372457.1:0.1403854998)/1:0.0650677239,((Linnemannia
schmuckeri_KAF9142540.1:0.0834560125,((Mortierella sp.
GBA39_KAF9143925.1:0.0170039925,(Mortierella
hygrophila_KAF9540845.1:0.0253520281,Linnemannia
hyalina_KAG9067608.1:0.0164572078)/0.97:0.0068771682)/0.774:0.0112235530,Linnema
nnia elongata
AG-77_OAQ28144.1:0.1197297746)/1:0.1004824231)/1:0.0453376794,Linnemannia
gamsii_KAG0298193.1:0.1394405740)/1:0.0358499308)/1:0.0561923524)/
1:0.3254994884,(Mortierella antarctica_KAF9980232.1:0.3464808487,Mortierella sp.

GBA30_KAG0199716.1:0.1735863277)/1:0.1109334782)/1:0.0478849391)/0.78:0.02366462
39,((Haplosporangium sp. Z 767_KAF9187567.1:0.0297076613,Haplosporangium sp. Z
11_KAF9193873.1:0.0067783870)/1:0.0347730225,Mortierella
polycephala_KAG0247366.1:0.0926077460)/1:0.3602508430)/1:0.0826342118,Gryganskie
lla cystojenkinii_KAG0049119.1:0.6762398156)/1:0.0908276397,
((((((Haplosporangium bisporale_KAF8935665.1:0.0038555084,Podila verticillata
NRRL 6337_KFH65728.1:0.0051815565)/1:0.1169868162,Podila
horticola_KAF9315083.1:0.0584456713)/0.85:0.0174459887,(Podila
clonocystis_KAG0030467.1:0.1140558734,Podila
minutissima_KAG0359137.1:0.0783452518)/0:0.0000021690)/0.826:0.0536953687,Podila
clonocystis_KAG0030468.1:0.3840761926)/0.991:0.0833137436,Podila
humilis_KAG0348690.1:0.3779987297)/1:0.0801100001,Podila
epigama_KAF9431065.1:0.4163200648)/1:0.2313303405)/0.996:0.0982044782,Lunasporan
giospora selenospora_KAF9580029.1:0.7979987268)/0.438:0.0317595543,Mortierella
sp. NVP85_KAF9362343.1:0.4327714427)/0.98:0.1885937990,(Actinomortierella
wolfii_KAG0225973.1:0.2023839888,Actinomortierella
ambigua_KAG0269474.1:0.2767445814)/1:0.6347966070)/1:0.6630790455,
(Paramicrosporidium saccamoebae_PJF17718.1:1.4476440529,Capsaspora owczarzaki
ATCC 30864_XP_004365112.1:1.1930847570)/0.993:0.2174946610)/1:0.2166770419)/
1:0.1001511060,((((((Neocallimastix sp. JGI-
2020a_KAG4084586.1:0.0000021734,Neocallimastix
californiae_ORY26000.1:0.0017134498)/1:0.1772745173,(Piromyces
finnis_ORX54342.1:0.3663589158,Anaeromyces
robustus_ORX79940.1:0.2439611950)/0.729:0.0907495848)/1:1.0443935828,
((Neocallimastix sp. JGI-2020a_KAG4103155.1:0.0000023474,Neocallimastix
californiae_ORY43212.1:0.0000024394)/0.999:0.1061309701,((Piromyces
finnis_ORX46883.1:0.2135072925,Piromyces sp.
E2_OUM69378.1:0.3044889867)/1:0.3076713995,Anaeromyces
robustus_ORX78839.1:0.1776044794)/0.995:0.1013214382)/1:0.9669494231)/
1:0.3191230263,Synchytrium
microbalum_TPX30617.1:1.4679485492)/0.668:0.1004681922,Gonapodya prolifera
JEL478_KXS16823.1:1.4863729483)/0.902:0.0761971616,(Linderina
pennispora_ORX66461.1:0.5994650750,Coemansia reversa NRRL
1564_PIA14848.1:0.9131158896)/1:1.0559756145)/1:0.1596001249)/0.97:0.0897703354)
/0.458:0.0290708432,Fonticula
alba_XP_009497567.1:2.3251515917)/0.75:0.0498067575,(Allomyces macrogynus ATCC
38327_KNE56303.1:1.0091401588,Catenaria anguillulae
PL171_ORZ29884.1:1.1197886318)/1:0.8074879668)/0.9:0.0805855554,
(((Caenorhabditis elegans_NP_510498.1:1.9619103549,Sphaeroforma arctica
JP610_XP_014159207.1:1.0575501487)/1:1.3573160704,Conidiobolus coronatus NRRL
28638_KXN68948.1:1.7675501648)/0.347:0.0960130158,Entomophthora
muscae_KAF7746593.1:1.4612766976)/0.993:0.1997850086)/1:0.2772151795,
((((((Encephalitozoon intestinalis ATCC 50506_ADM11612.1:0.3018208294,
(Encephalitozoon hellem ATCC 50504_AFM98379.1:0.1890951284,Encephalitozoon
romaleae
SJ-2008_AFN83098.1:0.1798959922)/0.978:0.0694650269)/1:0.2785756236,Ordospora
colligata OC4_KHN69512.1:0.7358662347)/1:0.7448198092,((Nosema bombycis
CQ1_EOB15228.1:0.4997367032,Nosema
granulosis_KAF9761860.1:0.2722956820)/1:0.6681373915,(Nosema apis BRL
01_EQB62005.1:0.9364809155,Nosema
ceranae_KKO75512.1:0.5127463840)/1:0.7118741772)/1:0.2123223662)/0.994:0.1495164
979,Vittaforma corneae ATCC 50505_ELA41634.1:1.2260095190)/1:0.2720287540,
((Vavraia culicis subsp. floridensis_ELA47627.1:0.0782788007,Trachipleistophora
hominis_ELQ76110.1:0.2245832333)/1:1.4738528059,(Anncaliia algerae
PRA339_KCZ81870.1:0.3180980144,Tubulinosema
ratisbonensis_RVD92136.1:0.4448650824)/1:0.7578658547)/0.63:0.1106972844)/
0.993:0.1184698991,(Thelohania contejeani_KAF7684340.1:1.0753822812,
((Hamiltosporidium magnivora_TBT99925.1:0.0000026177,Hamiltosporidium
magnivora_TBU01526.1:0.0000020755)/0.386:0.0104874894,Hamiltosporidium
tvaerminnensis_TBU12622.1:0.0339102562)/1:0.9144839740)/0.963:0.1461955285)/
0.993:0.2226688555,Edhazardia aedis USNM
41457_EJW02463.1:1.3540637908)/1:0.8866676013);
