# Supplementary material for: A minimal Fanconi Anemia complex in early diverging fungi
Source: Sci Rep. 2024 Apr 30;14:9922. doi: 10.1038/s41598-024-60318-w (PMC11061109; doi:10.1038/s41598-024-60318-w)
Supplement: Supplementary file 3 — Supplementary Figures. [file 41598_2024_60318_MOESM3_ESM.pdf]

MHF1

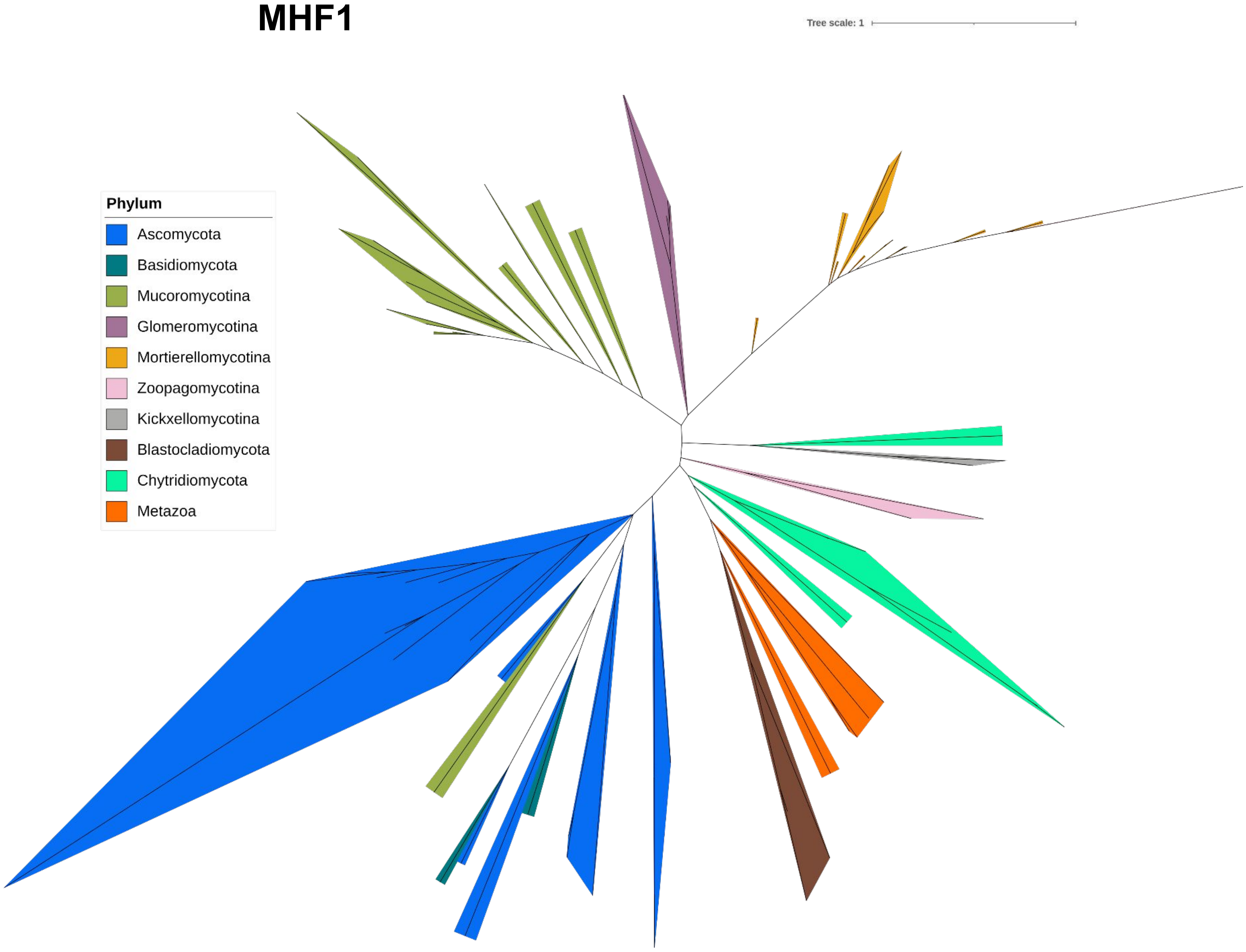
Tree scale: 1
Phylum
Ascomycota
Basidiomycota
Mucoromycotina
Glomeromycotina
Mortierellomycotina
Zoopagomycotina
Kickxellomycotina
Blastocladiomycota
Chytridiomycota
Metazoa

MHF2

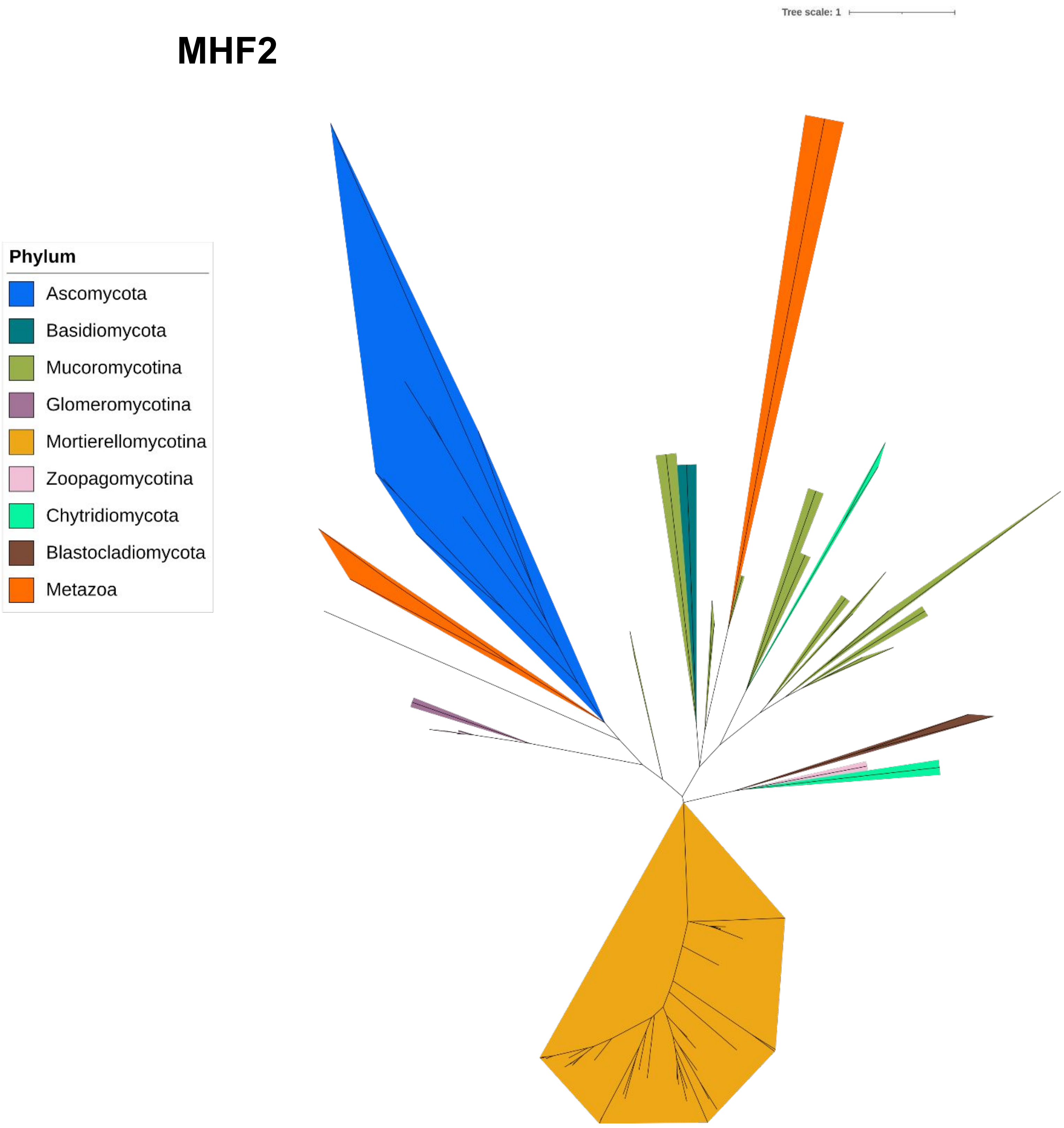
Tree scale: 1
Phylum
Ascomycota
Basidiomycota
Mucoromycotina
Glomeromycotina
Mortierellomycotina
Zoopagomycotina
Chytridiomycota
Blastocladiomycota
Metazoa

FANCA

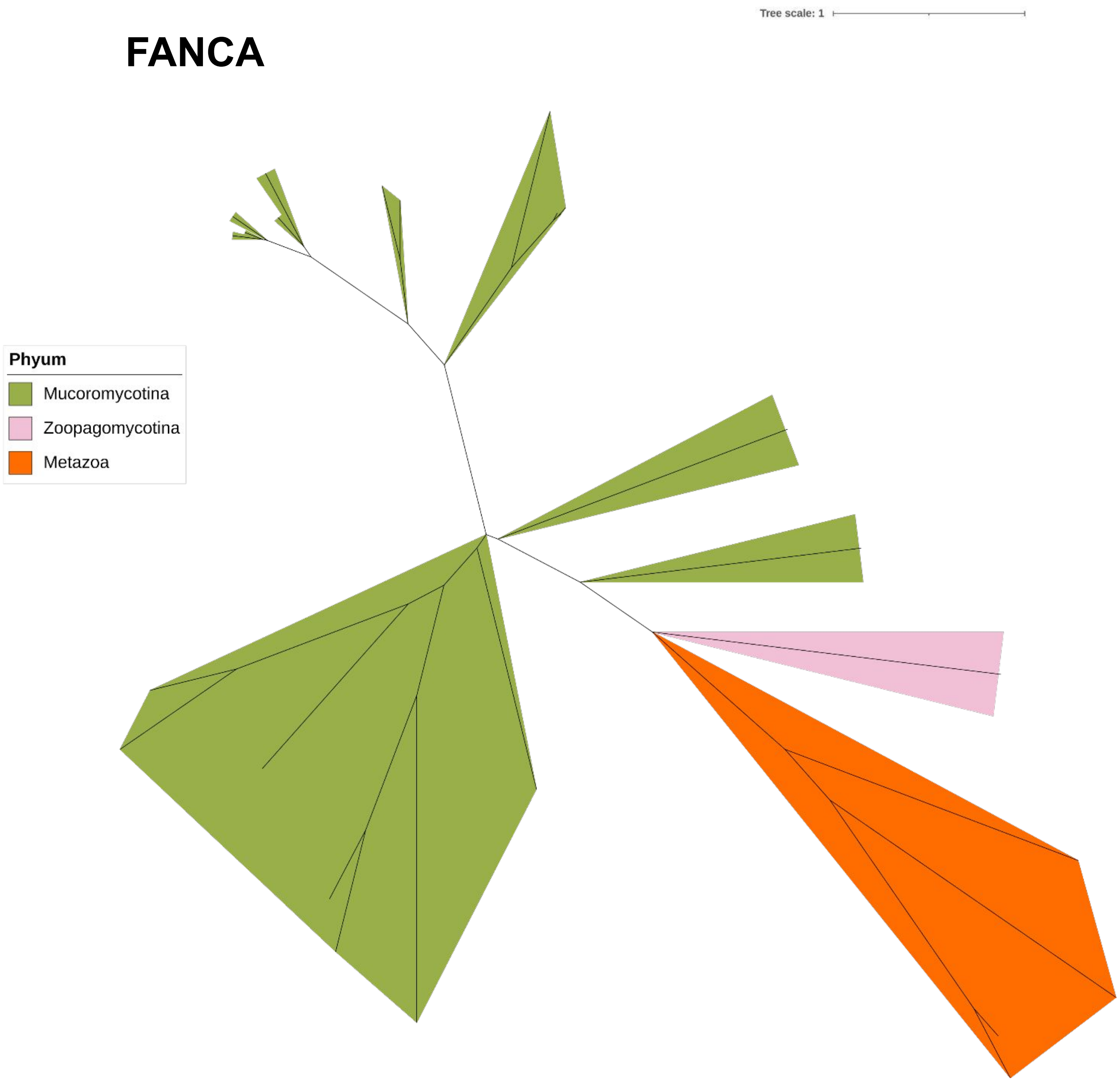
Tree scale: 1
Phyum
Mucoromycotina
Zoopagomycotina
Metazoa

FANCE

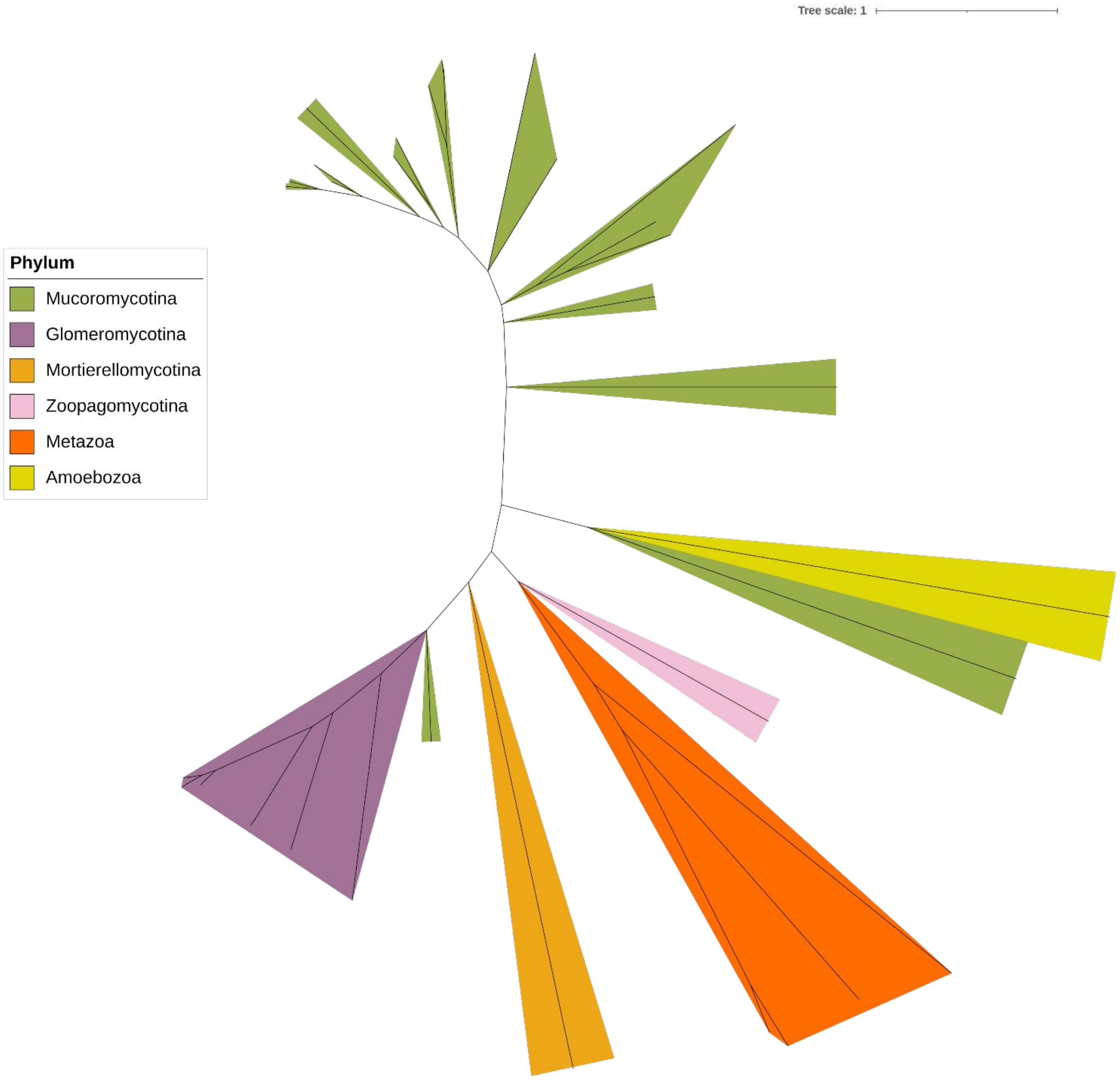
Tree scale: 1
Phylum
Mucoromycotina
Glomeromycotina
Mortierellomycotina
Zoopagomycotina
Metazoa
Amoebozoa

FANCJ

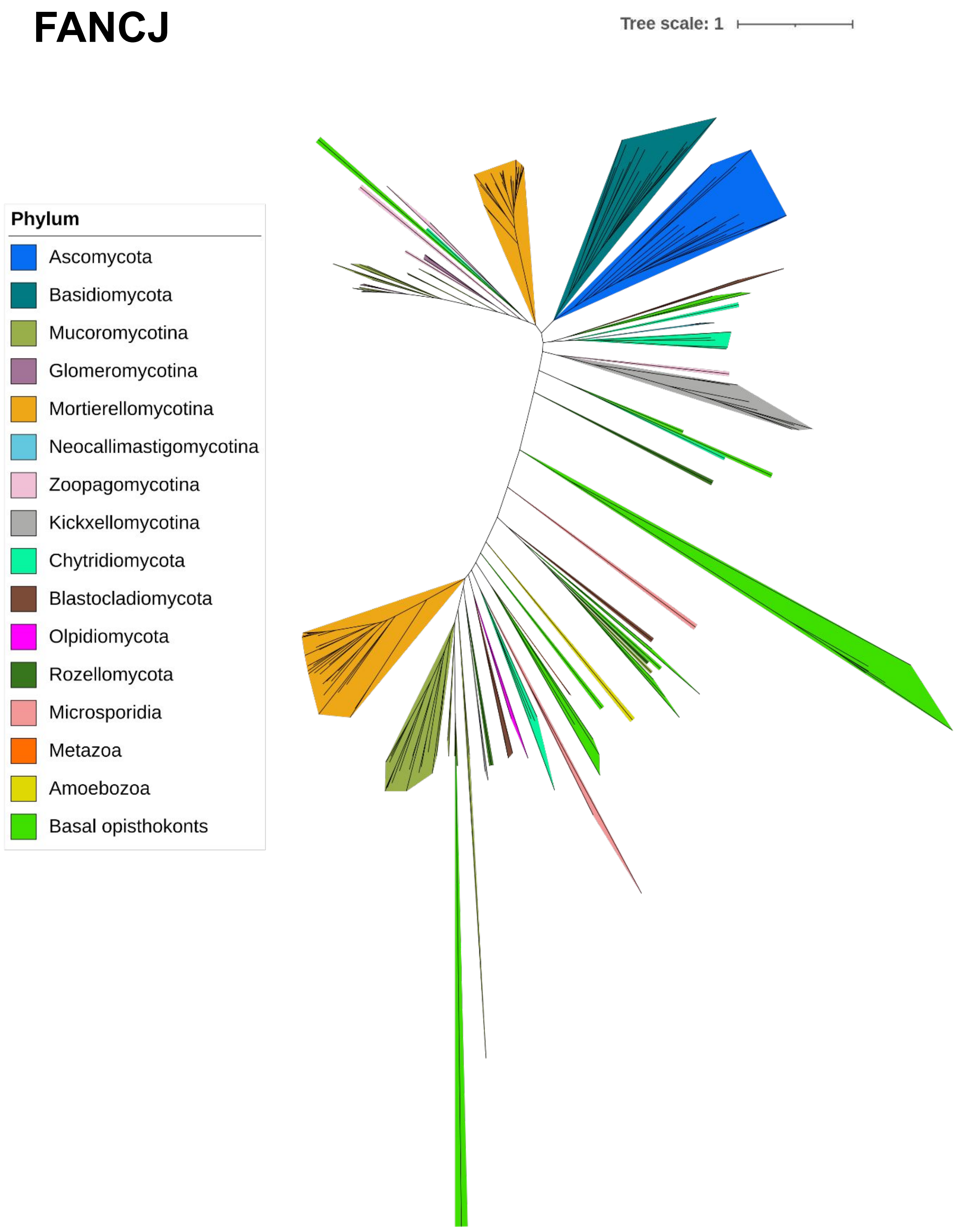

Tree scale: 1
Phylum
Ascomycota
Basidiomycota
Mucoromycotina
Glomeromycotina
Mortierellomycotina
Neocallimastigomycotina
Zoopagomycotina
Kickxellomycotina
Chytridiomycota
Blastocladiomycota
Olpidiomycota
Rozellomycota
Microsporidia
Metazoa
Amoebozoa
Basal opisthokonts

FANCL

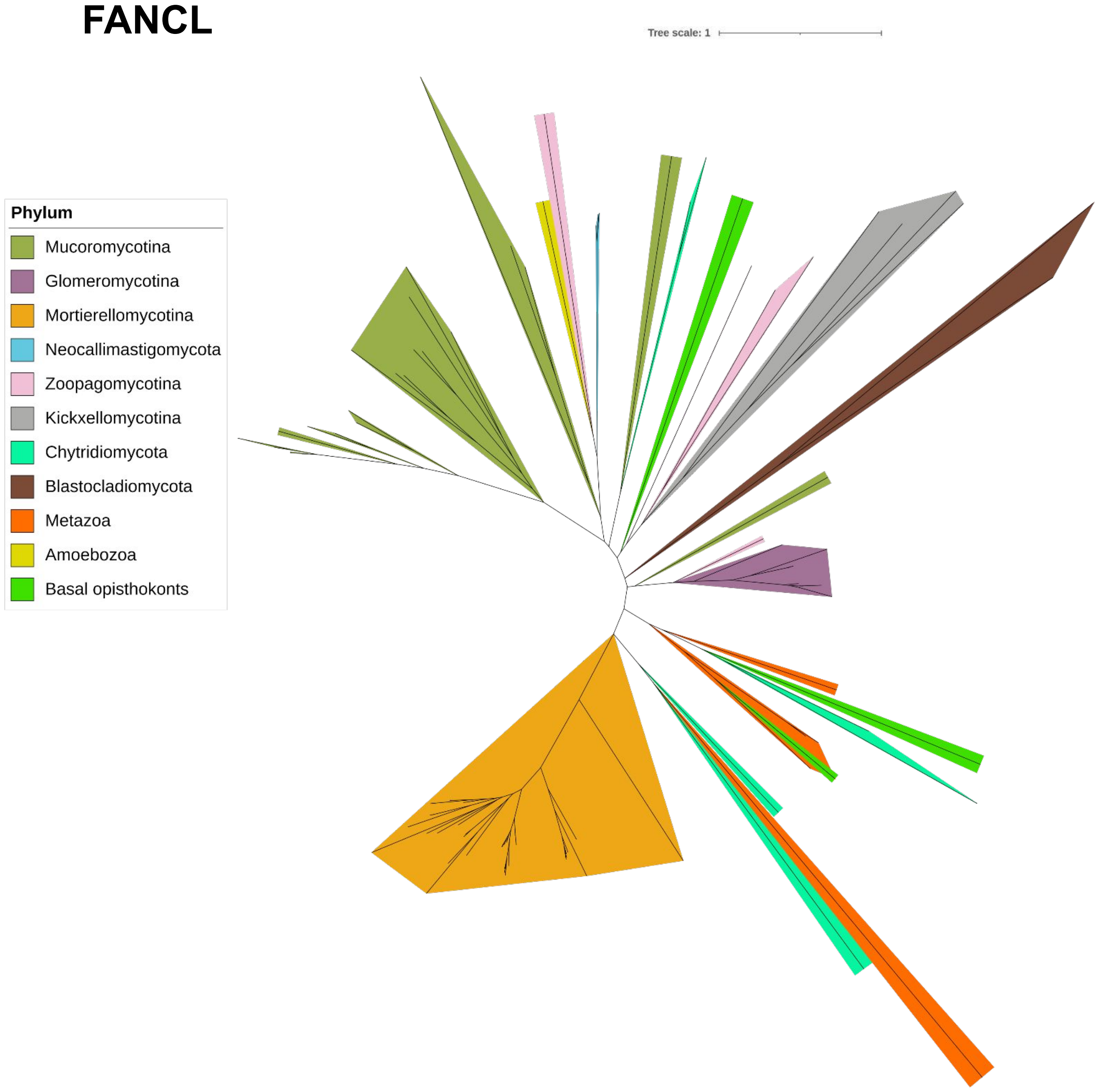

Tree scale: 1
Phylum
Mucoromycotina
Glomeromycotina
Mortierellomycotina
Neocallimastigomycota
Zoopagomycotina
Kickxellomycotina
Chytridiomycota
Blastocladiomycota
Metazoa
Amoebozoa
Basal opisthokonts

FANCM

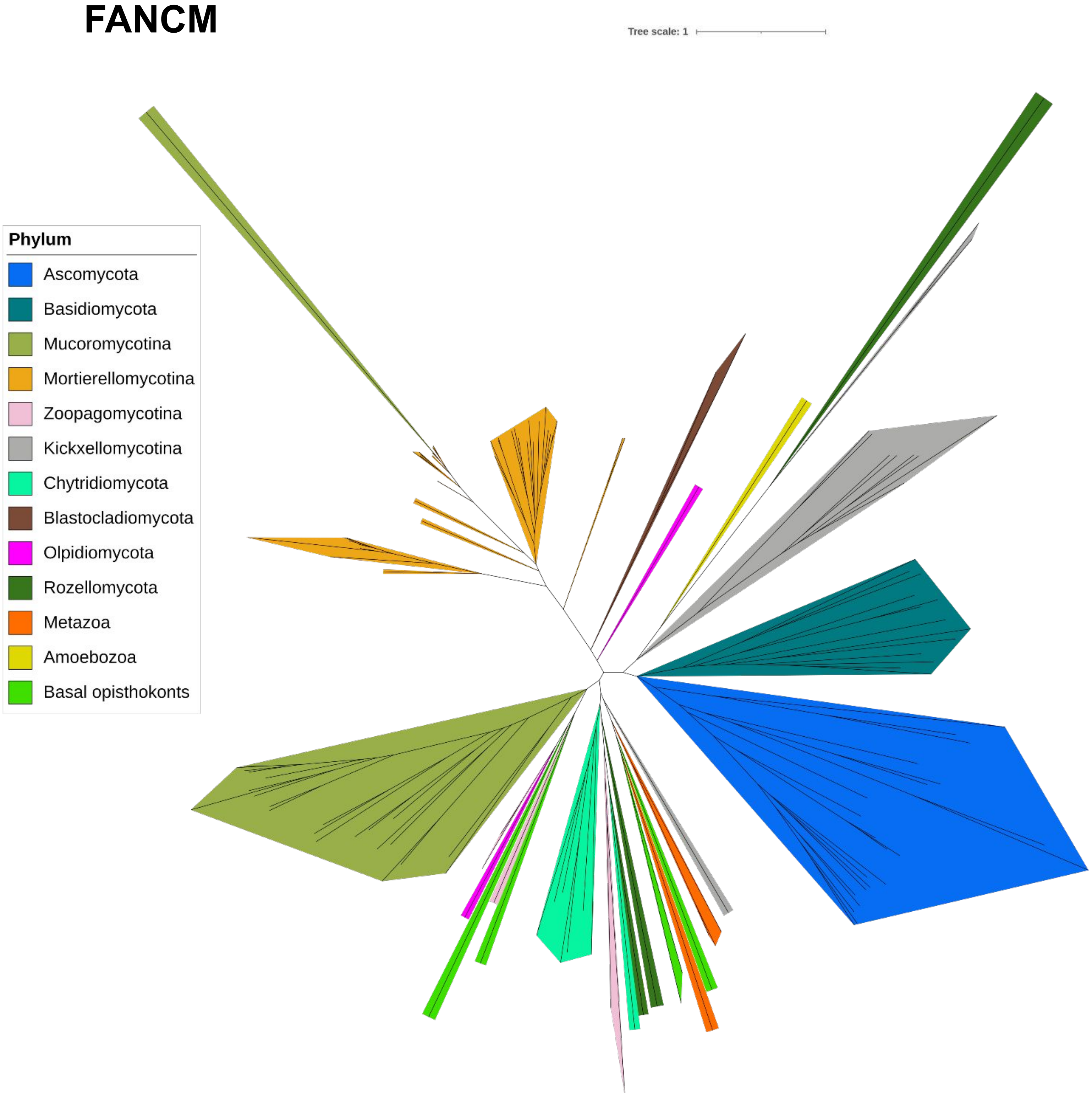

Tree scale: 1
Phylum
Ascomycota
Basidiomycota
Mucoromycotina
Mortierellomycotina
Zoopagomycotina
Kickxellomycotina
Chytridiomycota
Blastocladiomycota
Olpidiomycota
Rozellomycota
Metazoa
Amoebozoa
Basal opisthokonts

FANCO

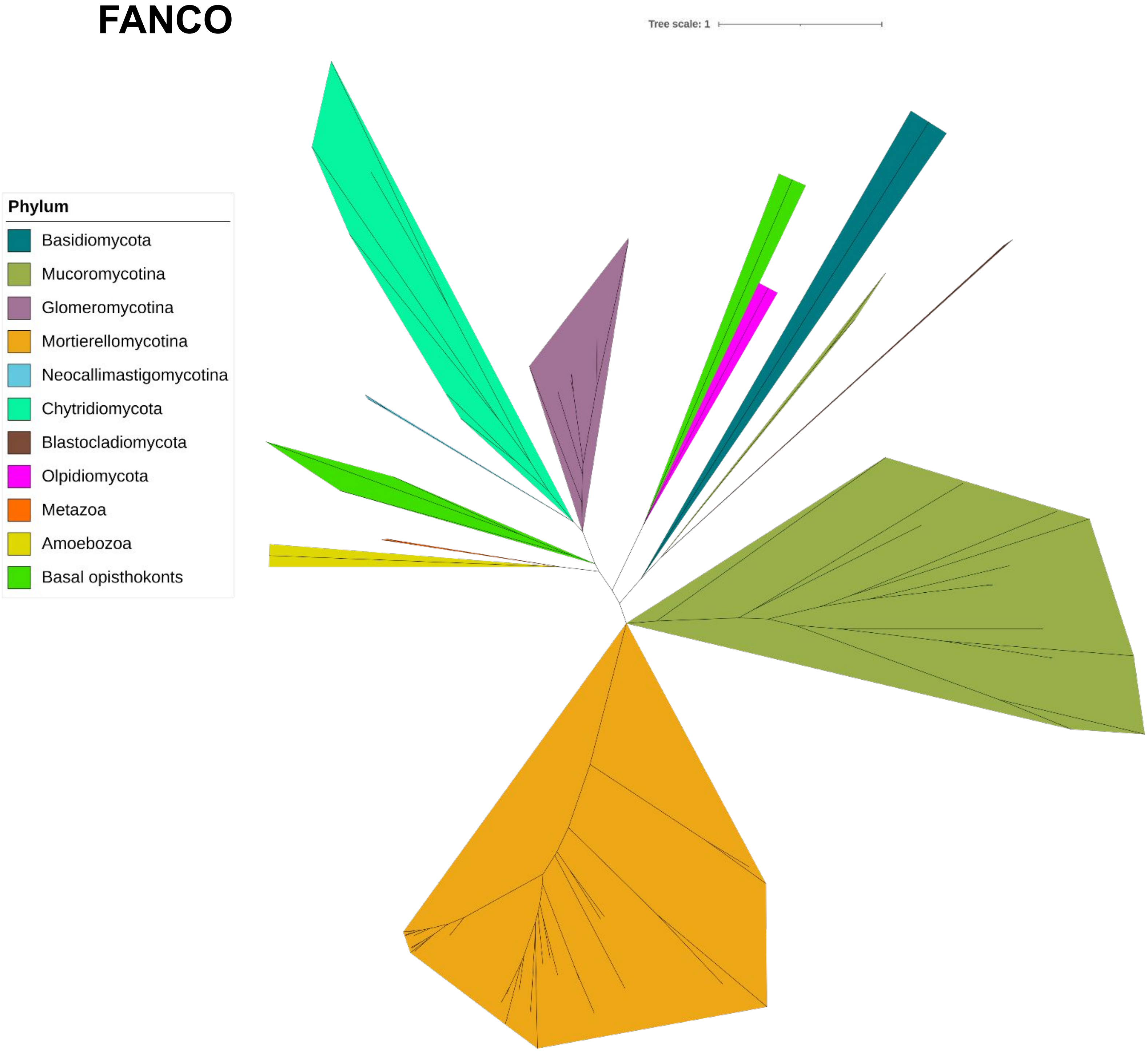

Tree scale: 1
Phylum
Basidiomycota
Mucoromycotina
Glomeromycotina
Mortierellomycotina
Neocallimastigomycotina
Chytridiomycota
Blastocladiomycota
Olpidiomycota
Metazoa
Amoebozoa
Basal opisthokonts

UBE2T

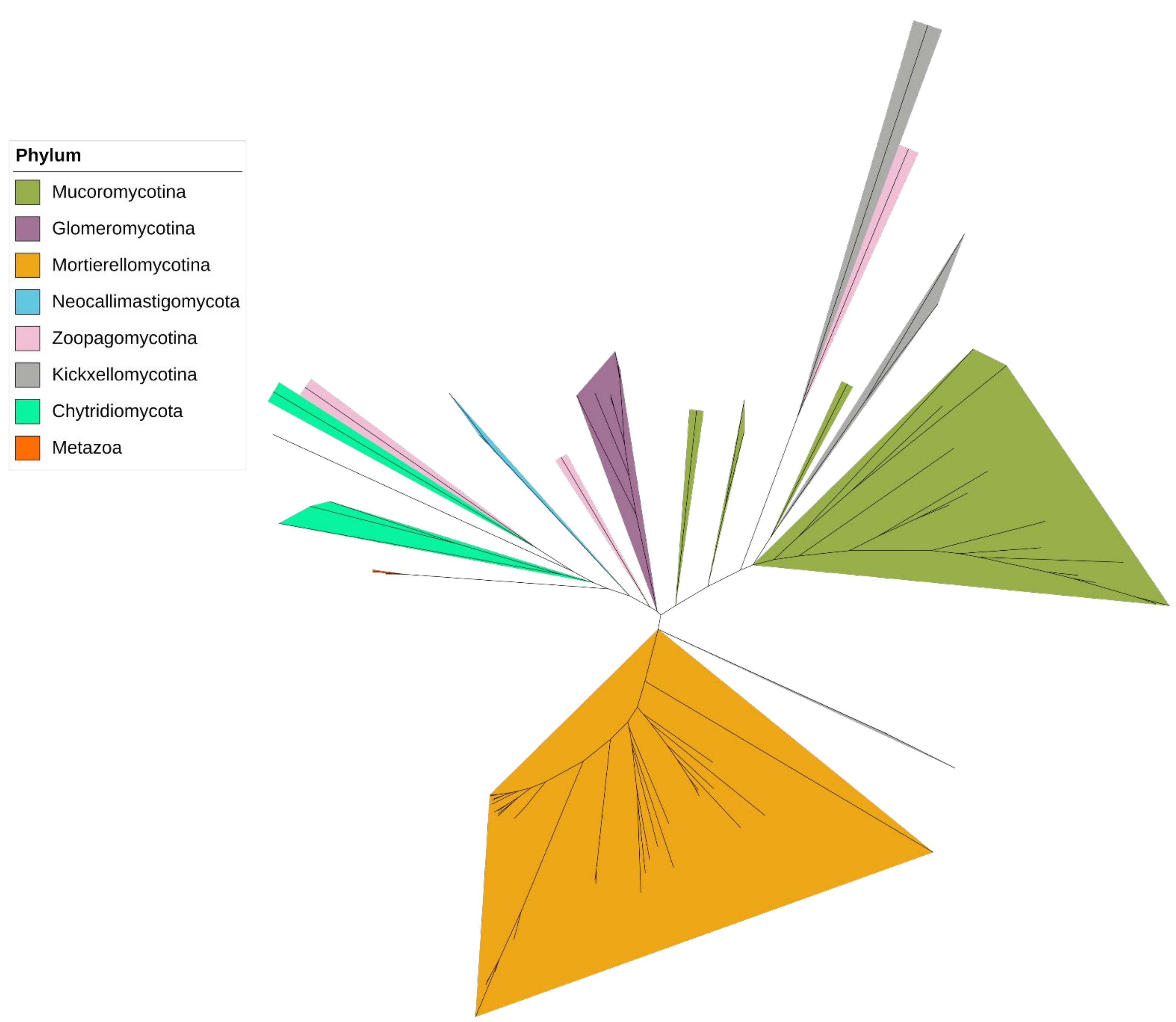
Tree scale: 1
Phylum
Mucoromycotina
Glomeromycotina
Mortierellomycotina
Neocallimastigomycota
Zoopagomycotina
Kickxellomycotina
Chytridiomycota
Metazoa

ATR

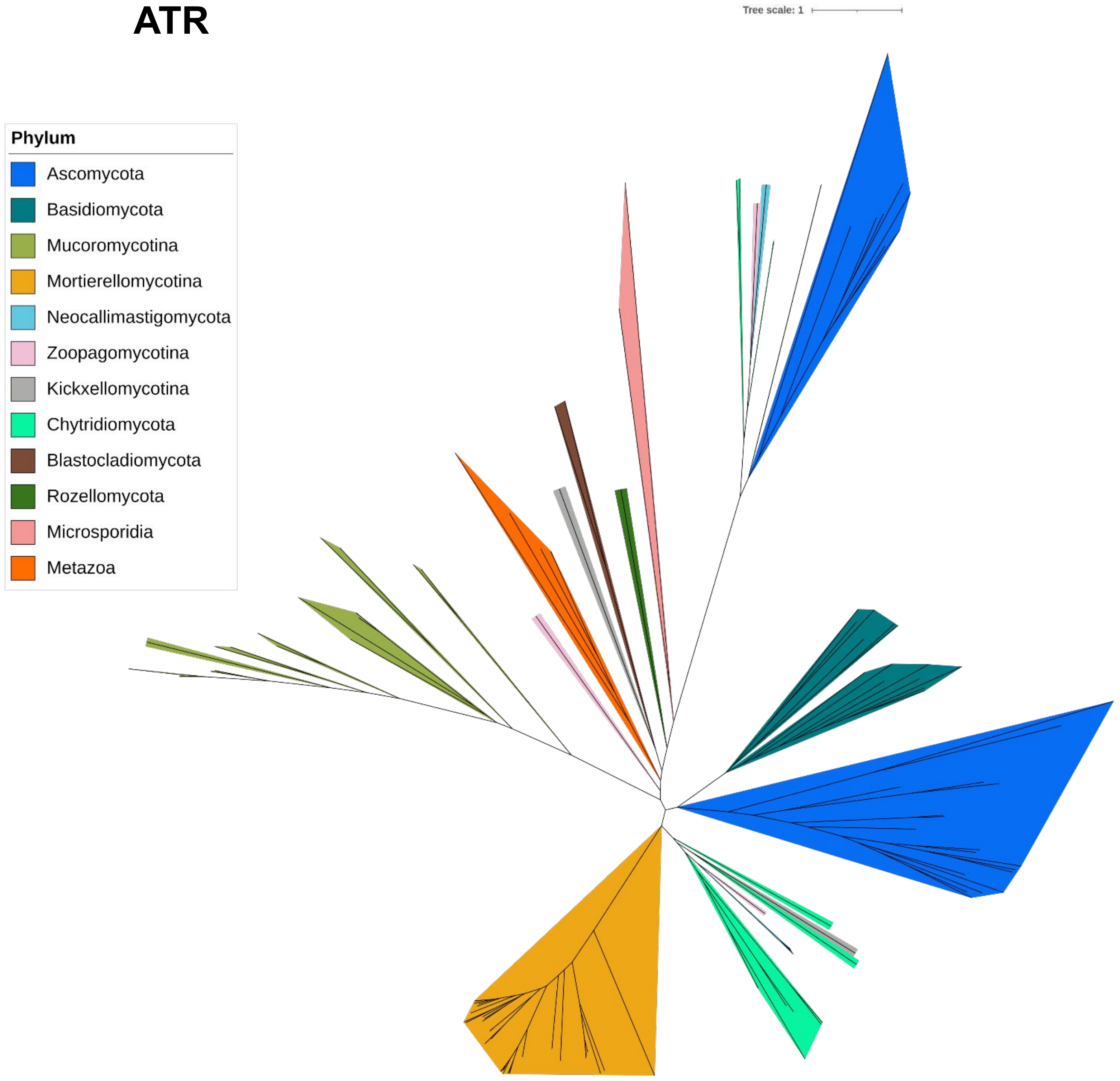
Tree scale: 1
Phylum
Ascomycota
Basidiomycota
Mucoromycotina
Mortierellomycotina
Neocallimastigomycota
Zoopagomycotina
Kickxellomycotina
Chytridiomycota
Blastocladiomycota
Rozellomycota
Microsporidia
Metazoa

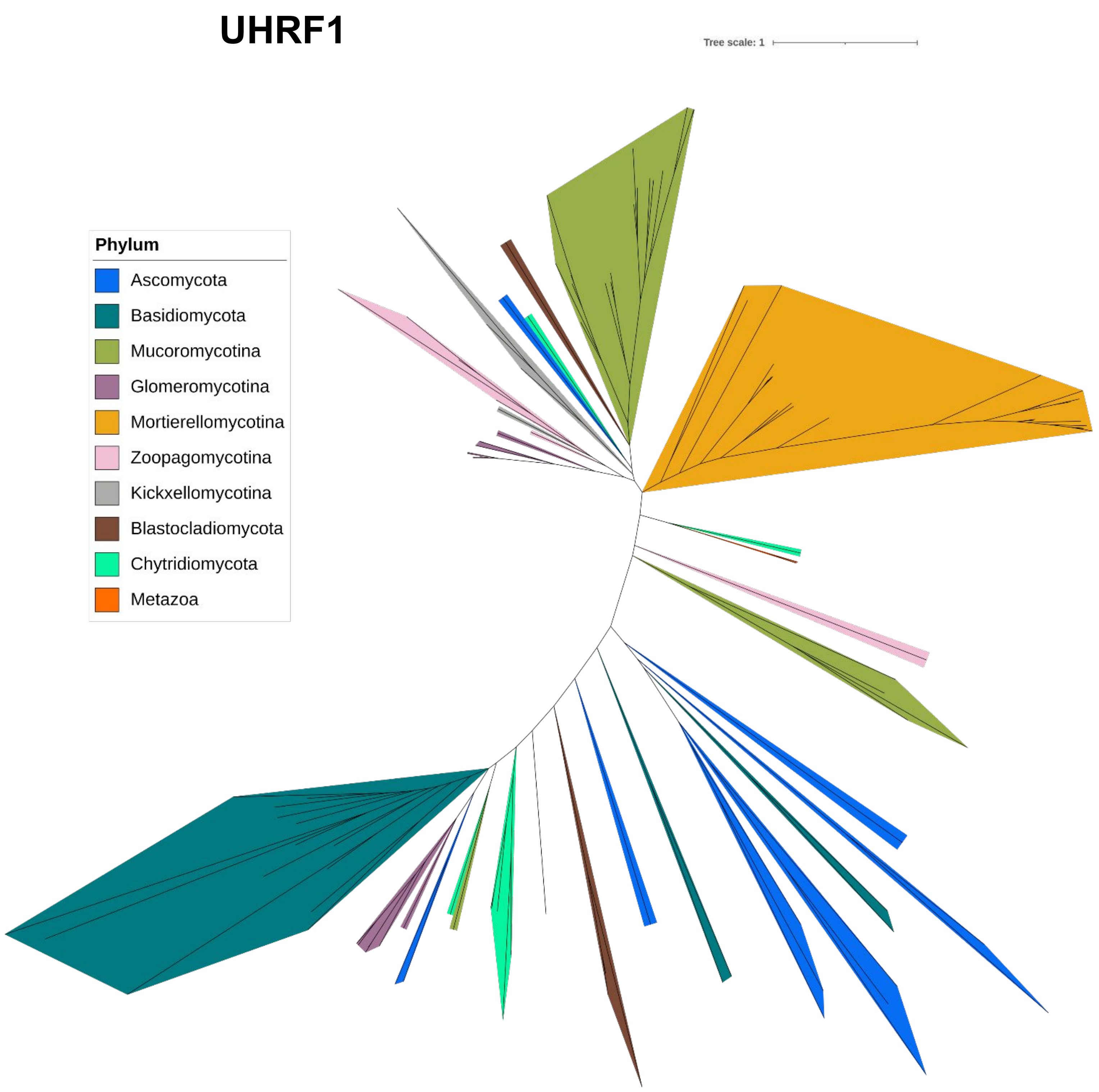
UHRF1
Tree scale: 1
Phylum
Ascomycota
Basidiomycota
Mucoromycotina
Glomeromycotina
Mortierellomycotina
Zoopagomycotina
Kickxellomycotina
Blastocladiomycota
Chytridiomycota
Metazoa

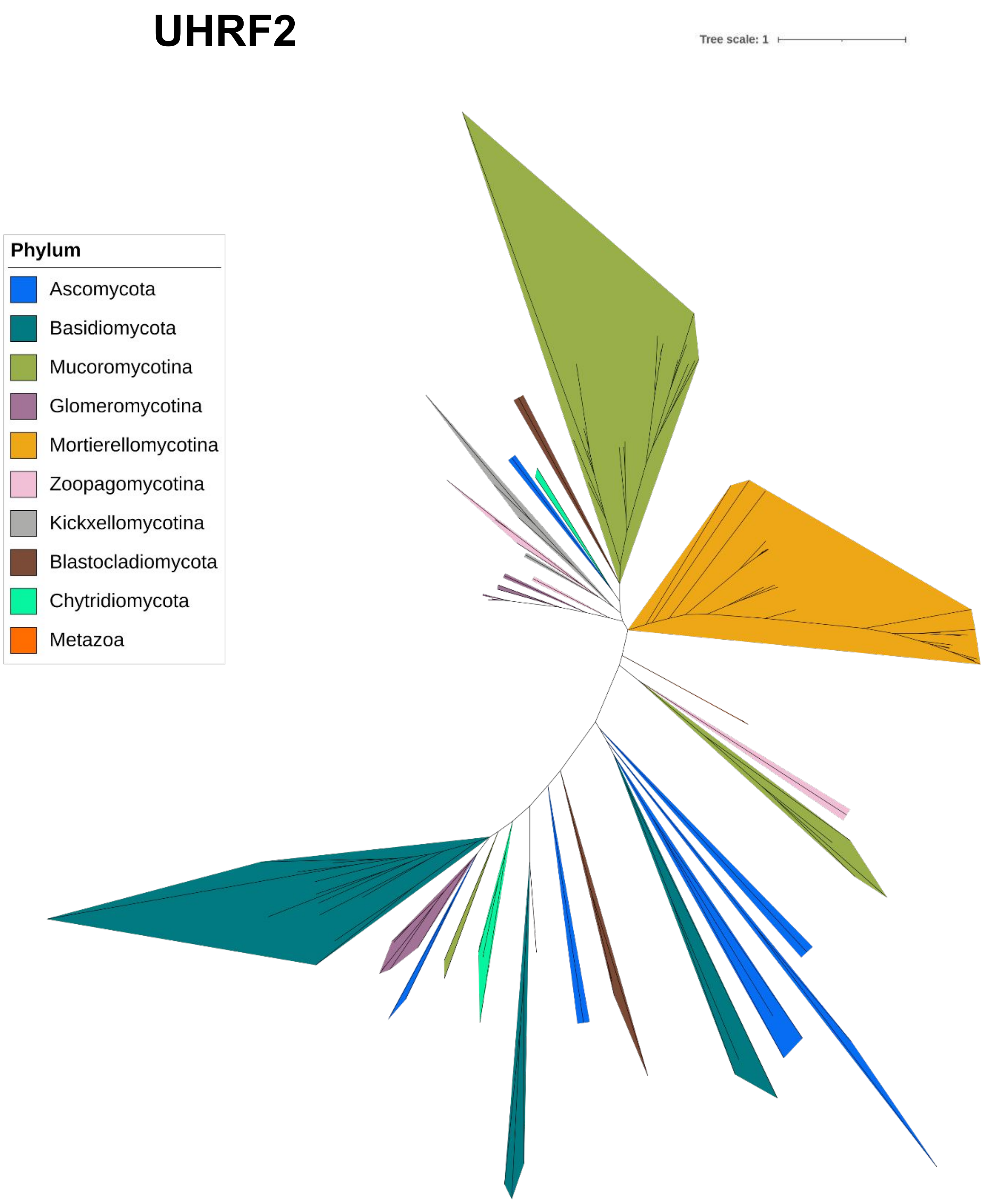
UHRF2
Tree scale: 1
Phylum
Ascomycota
Basidiomycota
Mucoromycotina
Glomeromycotina
Mortierellomycotina
Zoopagomycotina
Kickxellomycotina
Blastocladiomycota
Chytridiomycota
Metazoa

FANCD2

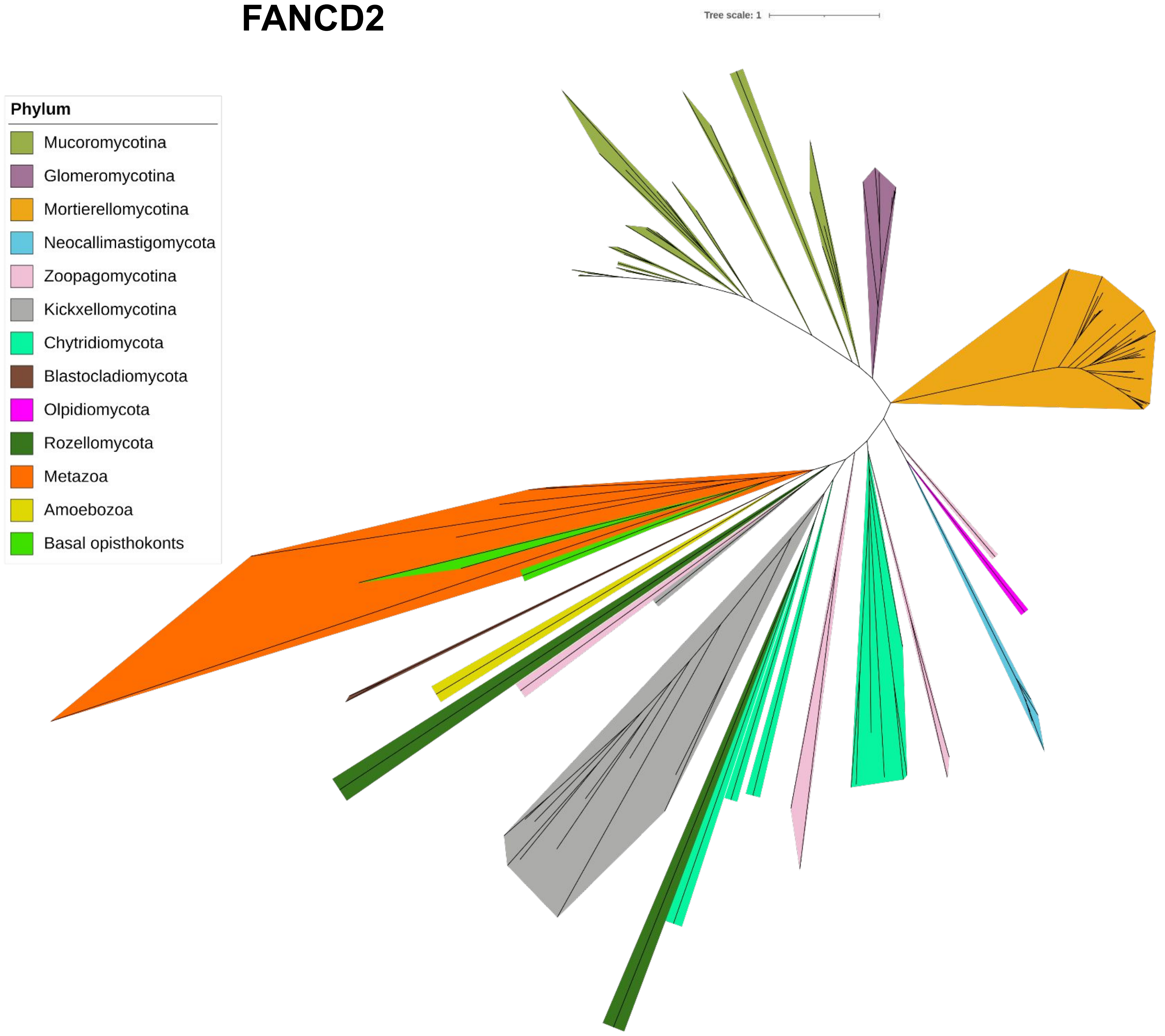

FANCI

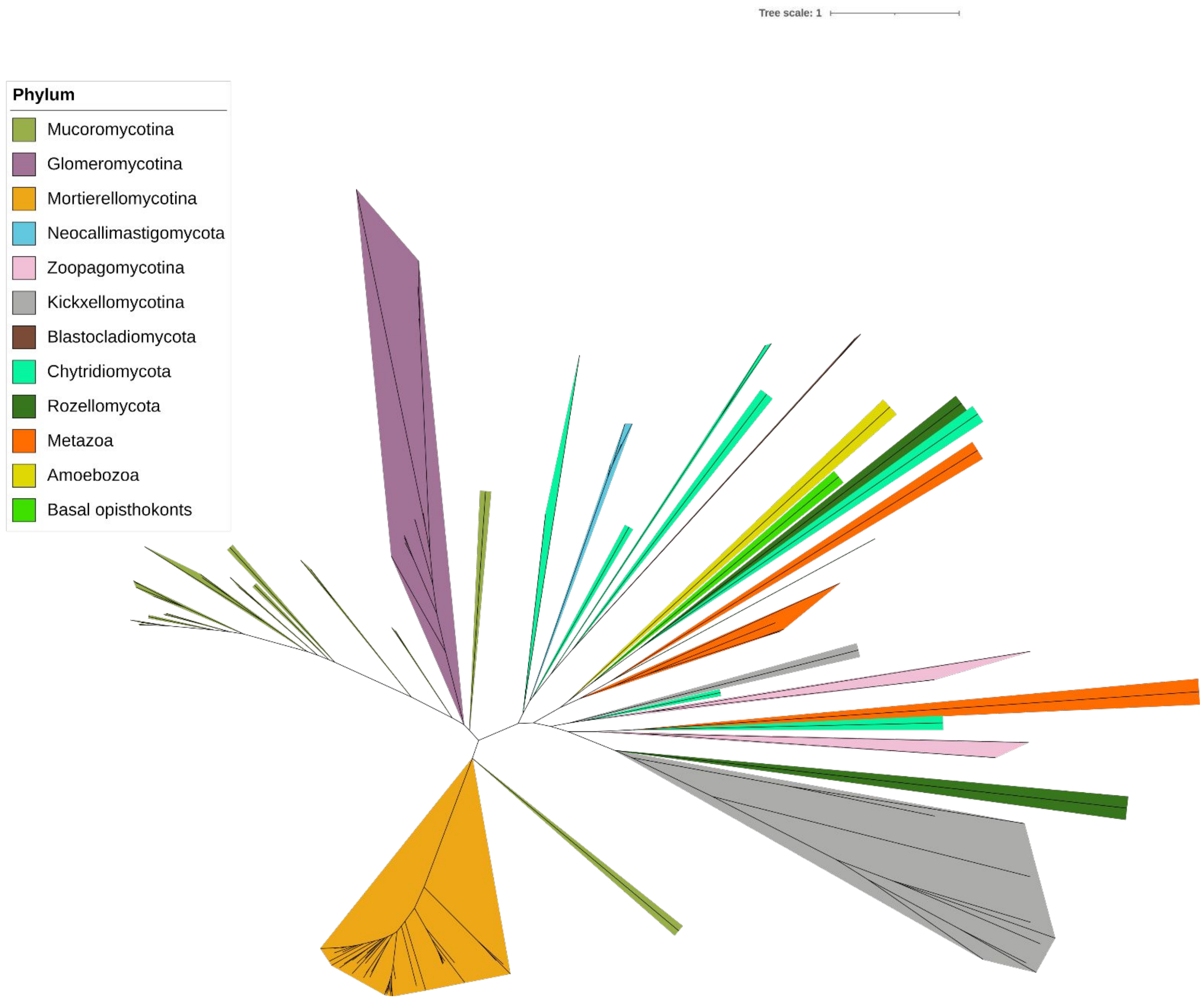

SLX1

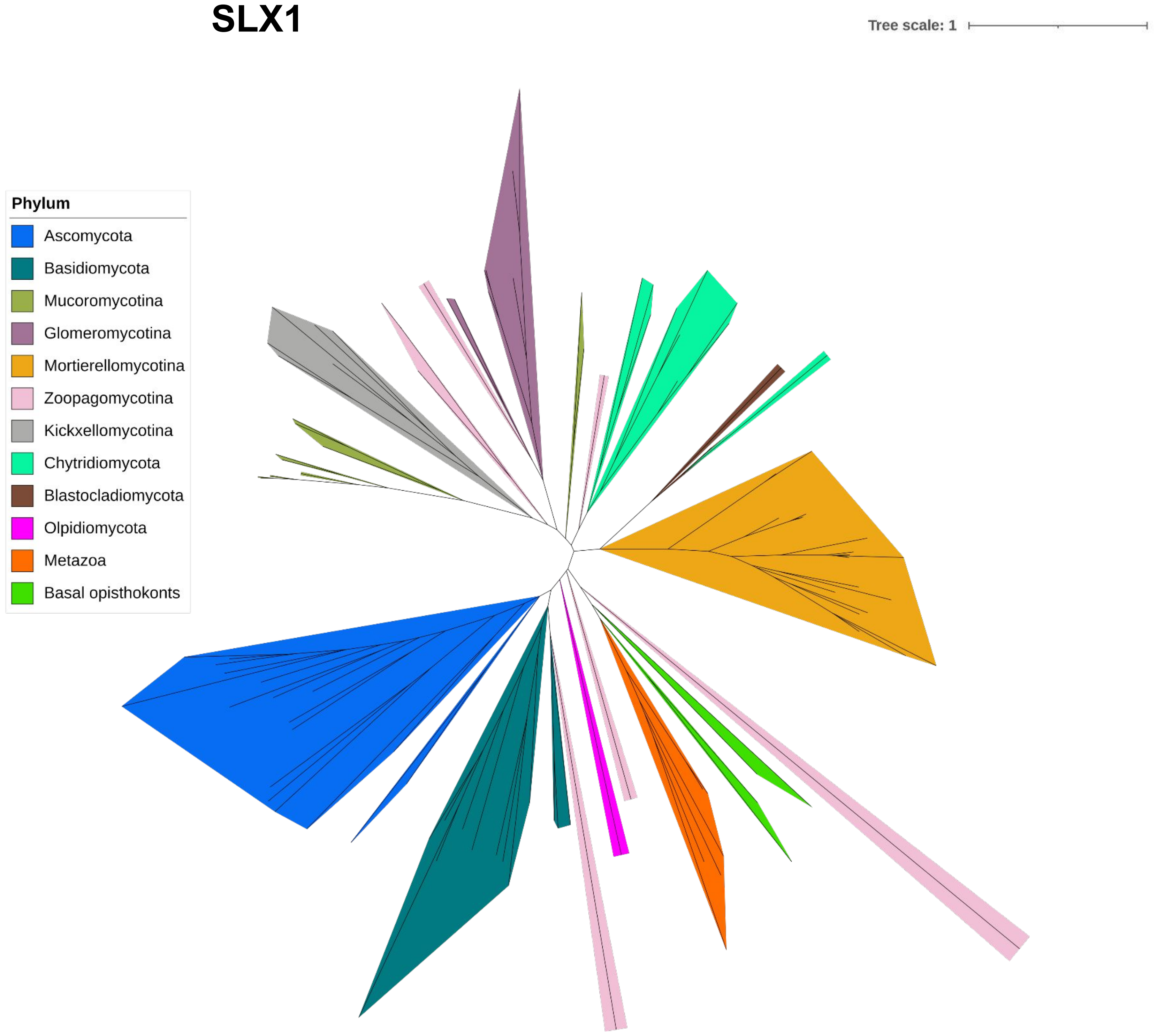
Tree scale: 1
Phylum
Ascomycota
Basidiomycota
Mucoromycotina
Glomeromycotina
Mortierellomycotina
Zoopagomycotina
Kickxellomycotina
Chytridiomycota
Blastocladiomycota
Olpidiomycota
Metazoa
Basal opisthokonts

SLX4

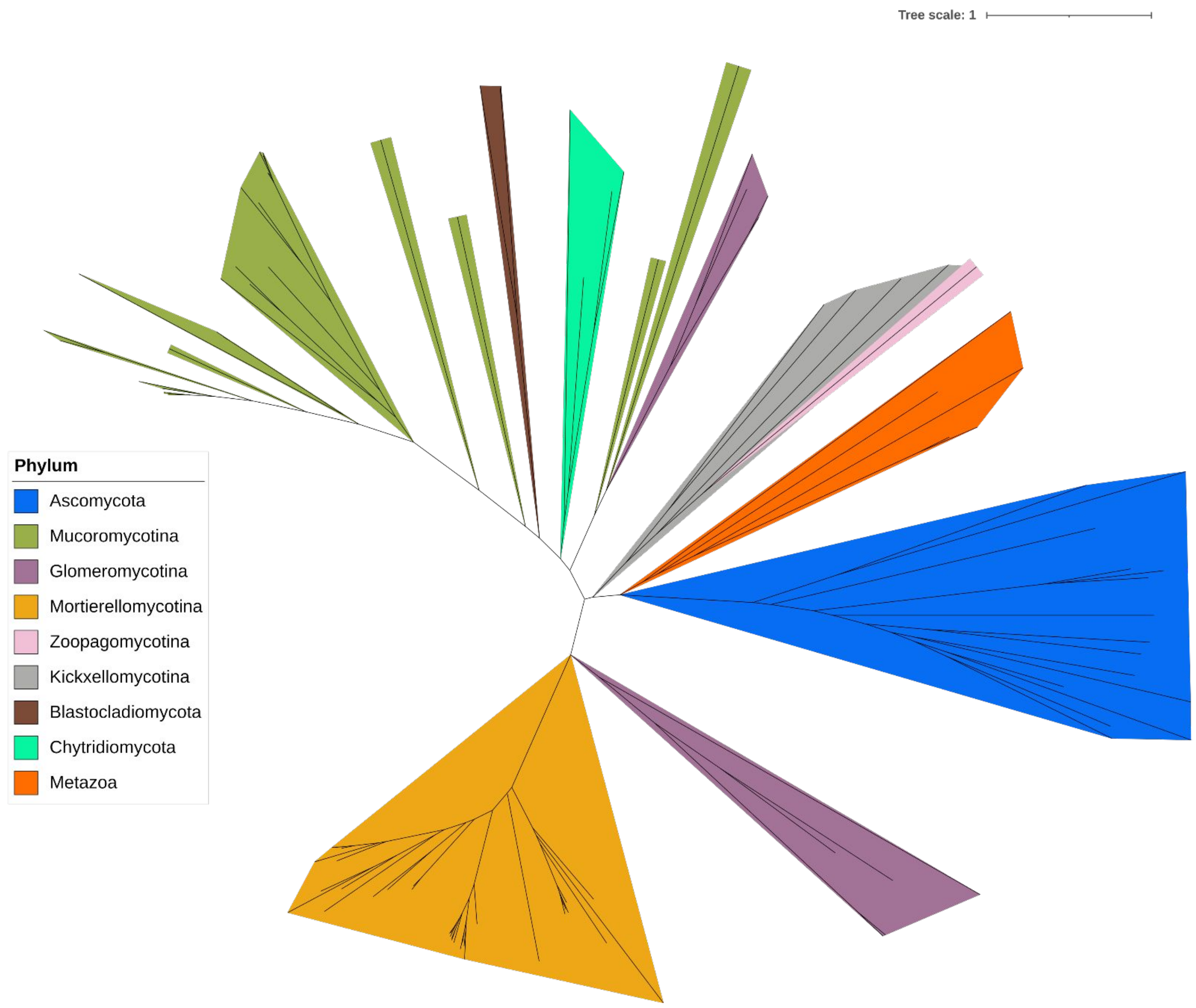
Tree scale: 1
Phylum
Ascomycota
Mucoromycotina
Glomeromycotina
Mortierellomycotina
Zoopagomycotina
Kickxellomycotina
Blastocladiomycota
Chytridiomycota
Metazoa

EME1

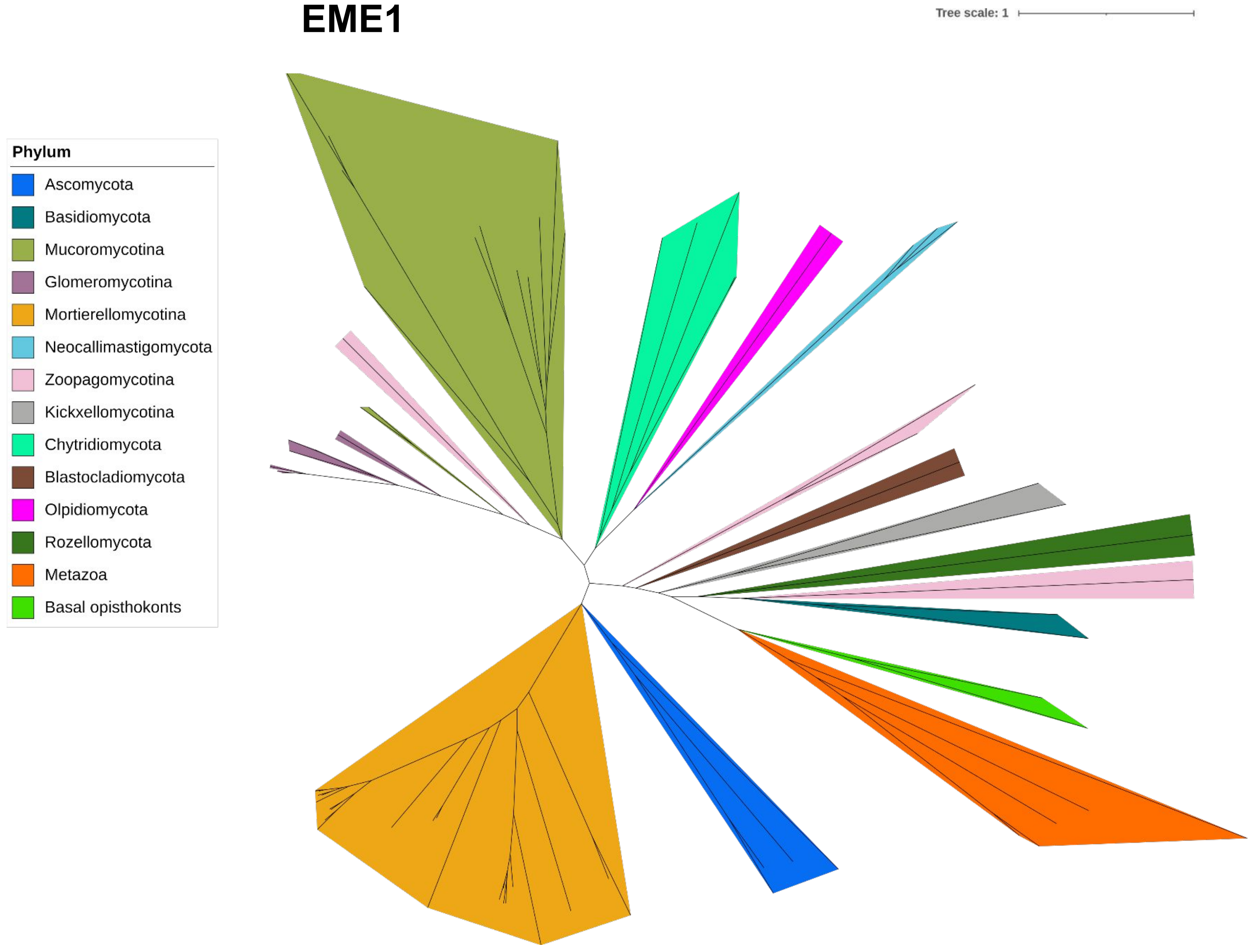
Tree scale: 1
Phylum
Ascomycota
Basidiomycota
Mucoromycotina
Glomeromycotina
Mortierellomycotina
Neocallimastigomycota
Zoopagomycotina
Kickxellomycotina
Chytridiomycota
Blastocladiomycota
Olpidiomycota
Rozellomycota
Metazoa
Basal opisthokonts

MUS81

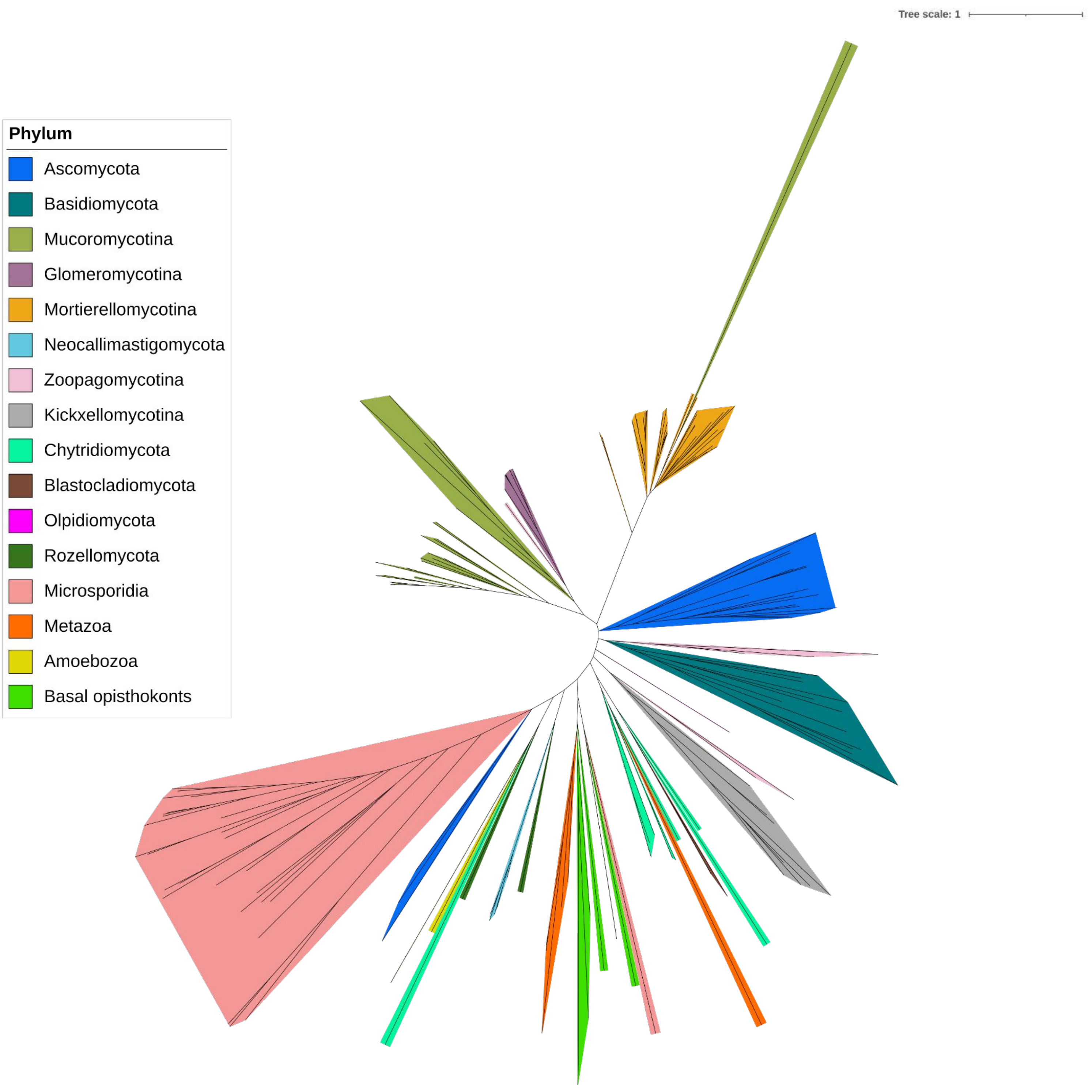
Tree scale: 1
Phylum
Ascomycota
Basidiomycota
Mucoromycotina
Glomeromycotina
Mortierellomycotina
Neocallimastigomycota
Zoopagomycotina
Kickxellomycotina
Chytridiomycota
Blastocladiomycota
Olpidiomycota
Rozellomycota
Microsporidia
Metazoa
Amoebozoa
Basal opisthokonts

XPF

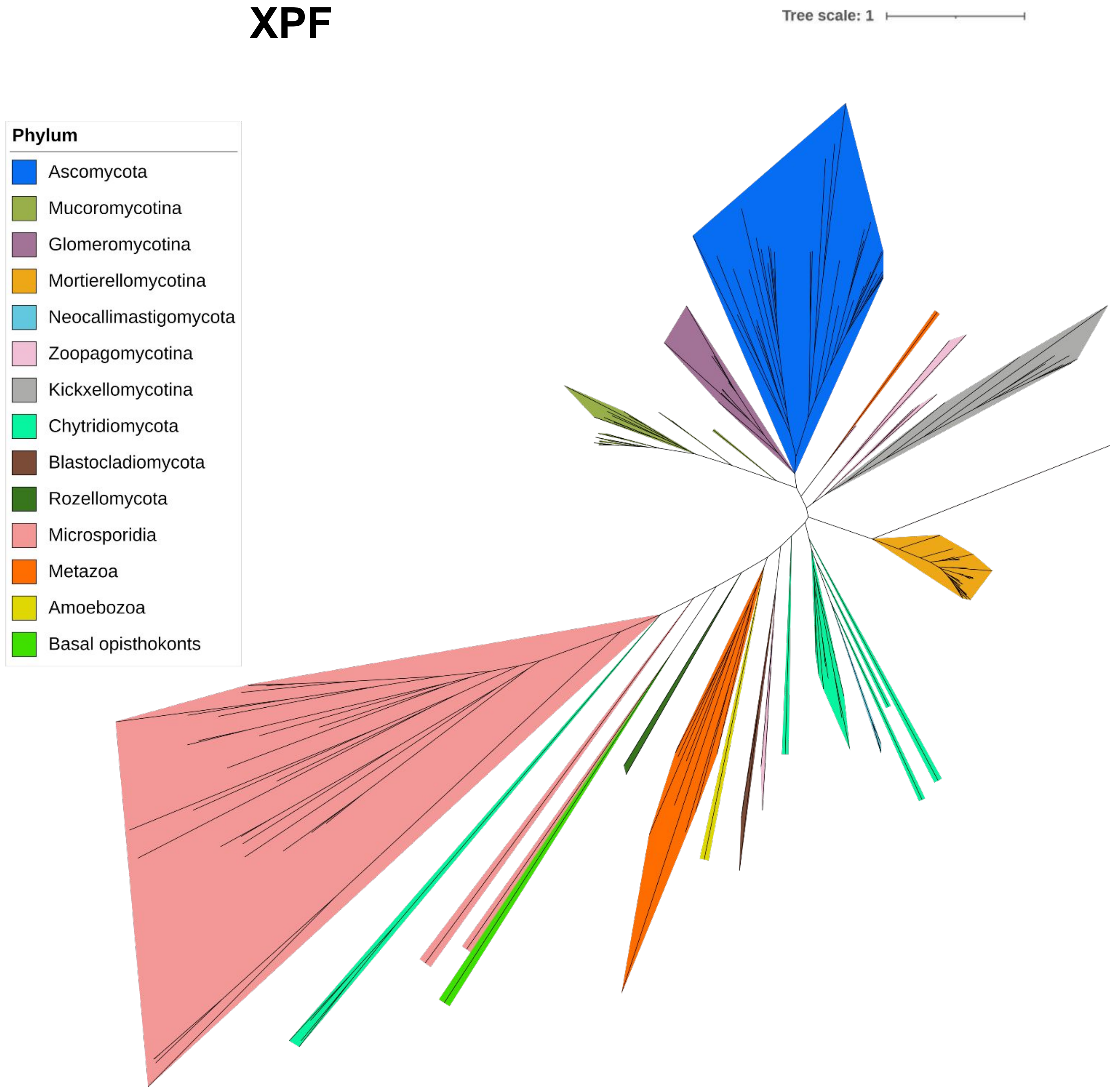

Tree scale: 1
Phylum
Ascomycota
Mucoromycotina
Glomeromycotina
Mortierellomycotina
Neocallimastigomycota
Zoopagomycotina
Kickxellomycotina
Chytridiomycota
Blastocladiomycota
Rozellomycota
Microsporidia
Metazoa
Amoebozoa
Basal opisthokonts

ERCC1

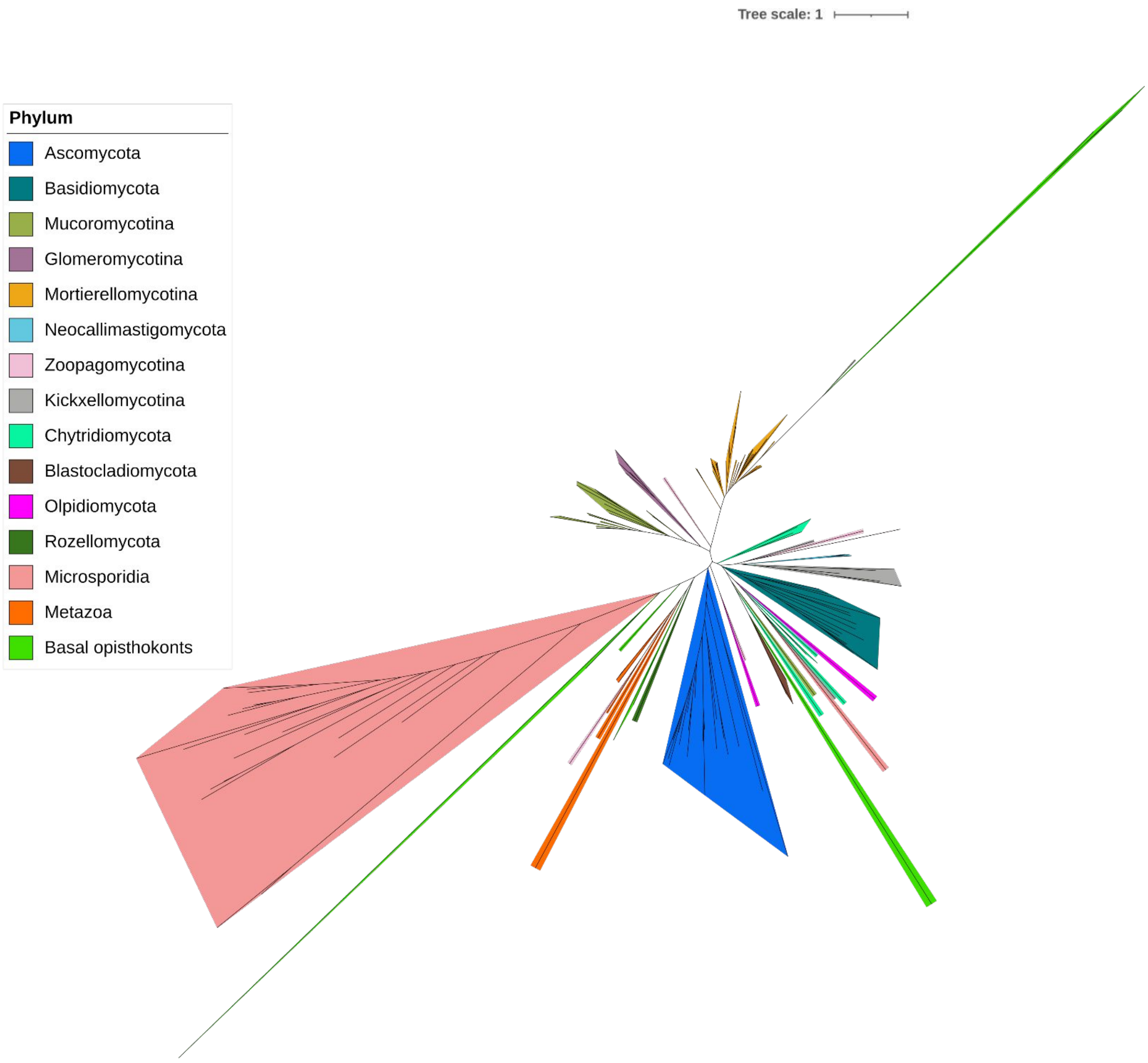

Tree scale: 1
Phylum
Ascomycota
Basidiomycota
Mucoromycotina
Glomeromycotina
Mortierellomycotina
Neocallimastigomycota
Zoopagomycotina
Kickxellomycotina
Chytridiomycota
Blastocladiomycota
Olpidiomycota
Rozellomycota
Microsporidia
Metazoa
Basal opisthokonts

FAN1

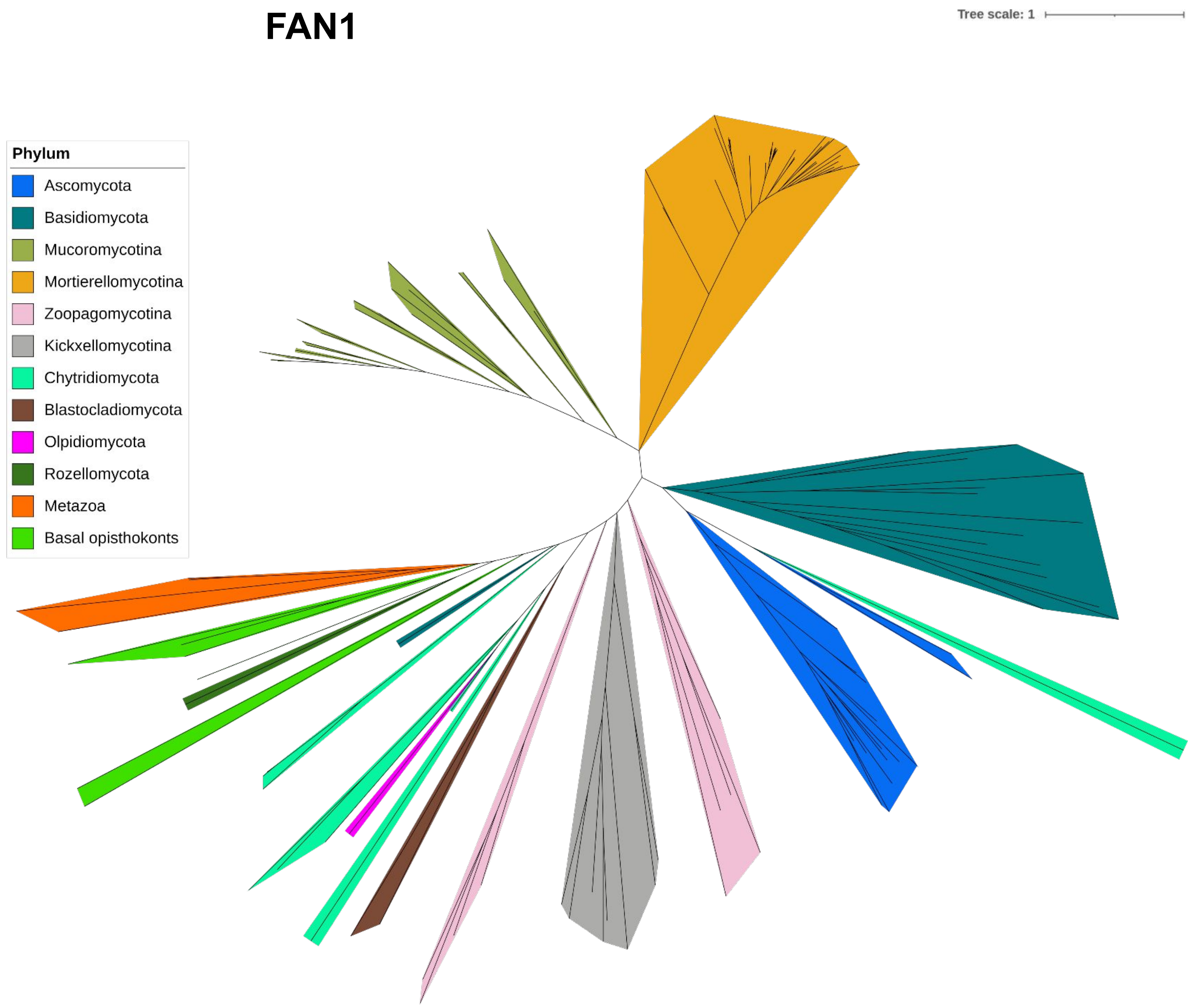
Tree scale: 1
Phylum
Ascomycota
Basidiomycota
Mucoromycotina
Mortierellomycotina
Zoopagomycotina
Kickxellomycotina
Chytridiomycota
Blastocladiomycota
Olpidiomycota
Rozellomycota
Metazoa
Basal opisthokonts

DPOLN

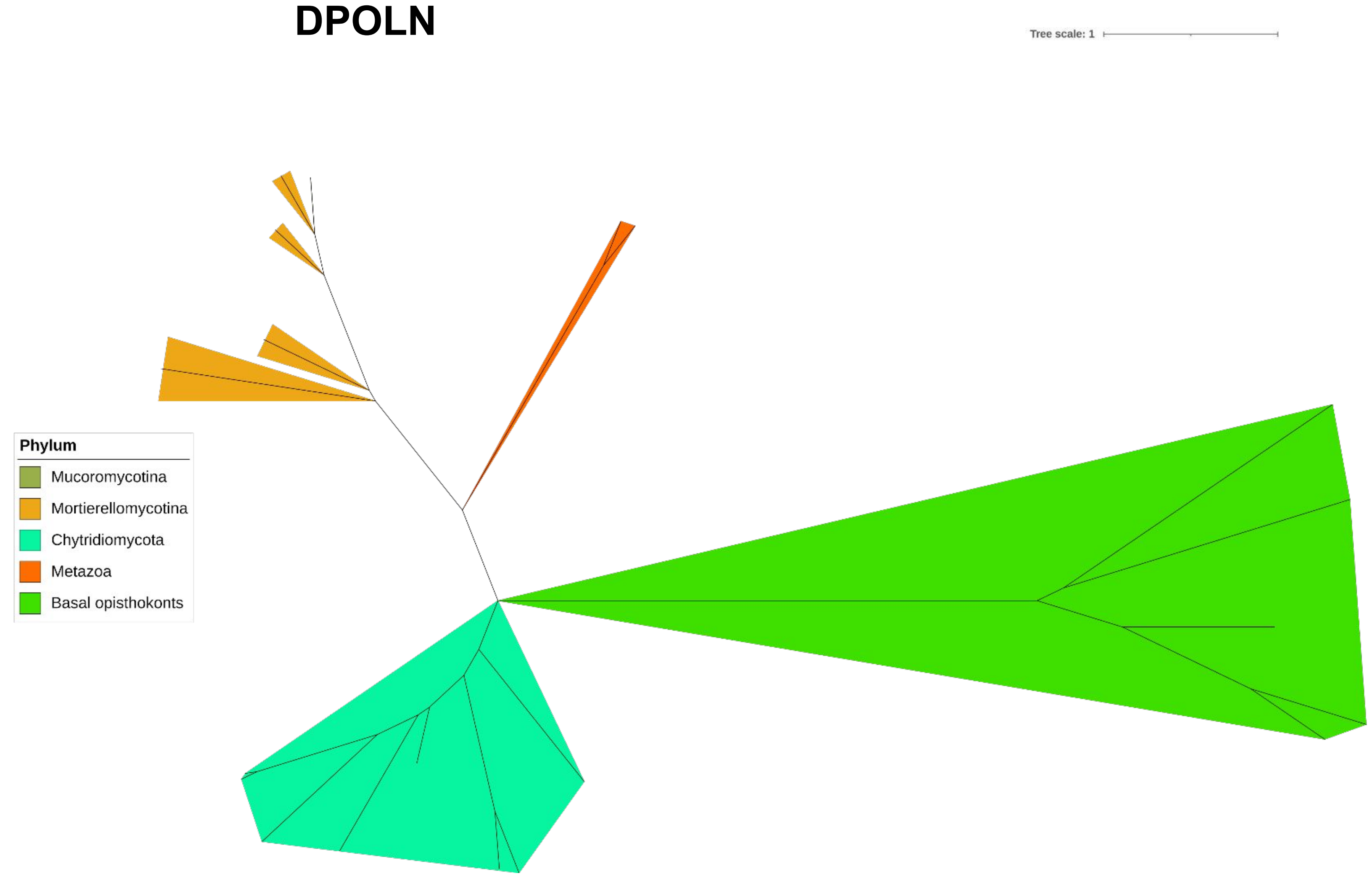
Tree scale: 1
Phylum
Mucoromycotina
Mortierellomycotina
Chytridiomycota
Metazoa
Basal opisthokonts

REV1

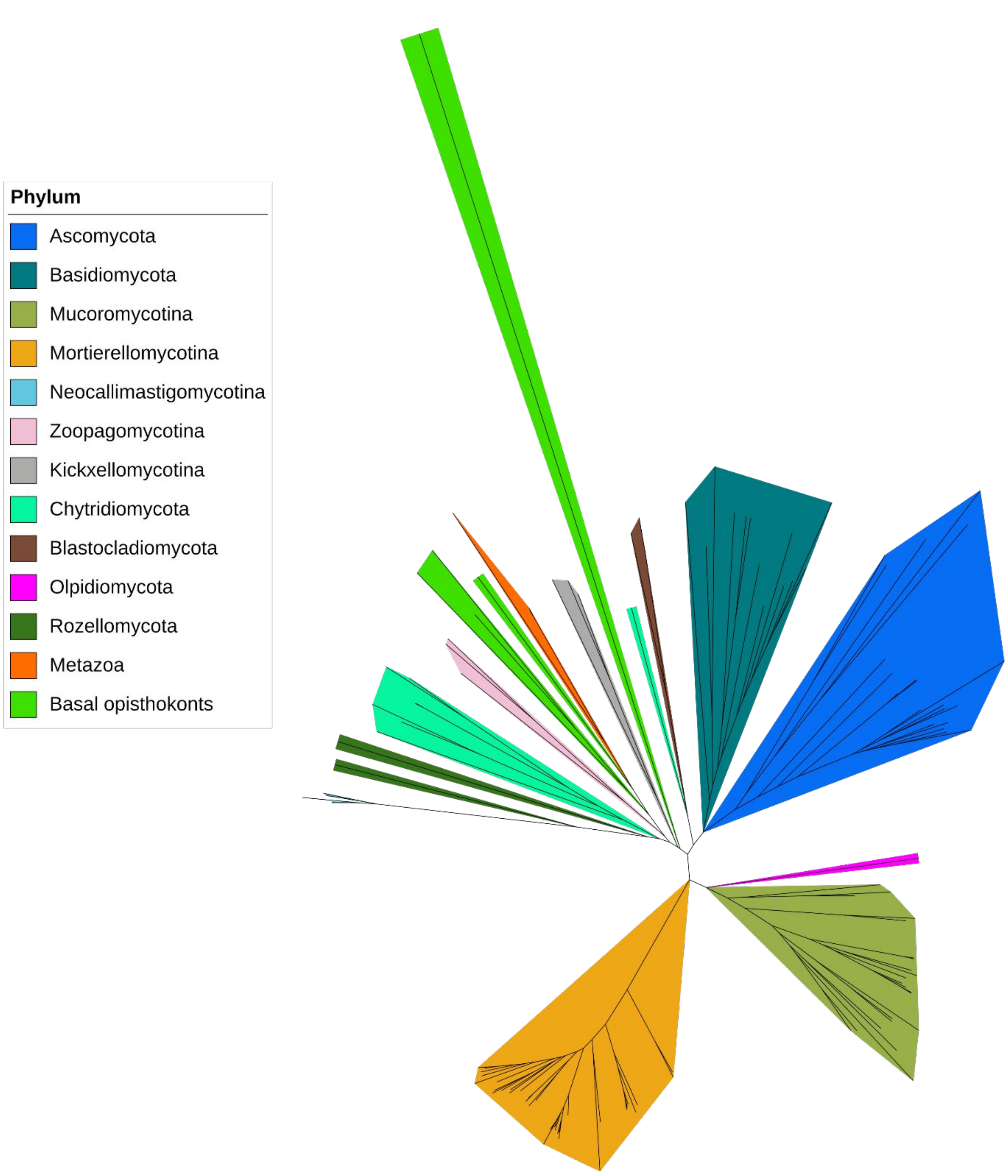

Tree scale: 1
Phylum
Ascomycota
Basidiomycota
Mucoromycotina
Mortierellomycotina
Neocallimastigomycotina
Zoopagomycotina
Kickxellomycotina
Chytridiomycota
Blastocladiomycota
Olpidiomycota
Rozellomycota
Metazoa
Basal opisthokonts

REV3

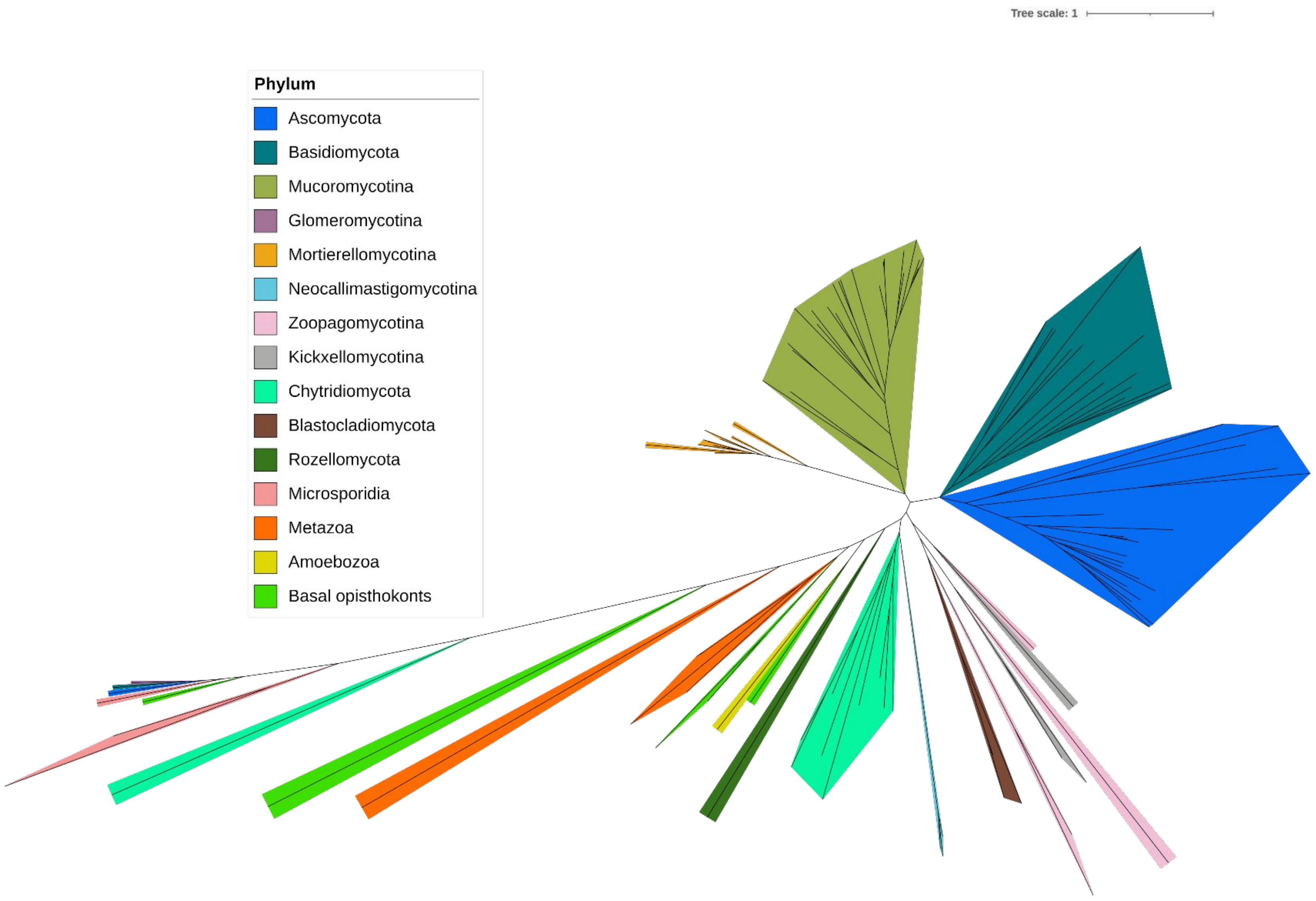

Tree scale: 1
Phylum
Ascomycota
Basidiomycota
Mucoromycotina
Glomeromycotina
Mortierellomycotina
Neocallimastigomycotina
Zoopagomycotina
Kickxellomycotina
Chytridiomycota
Blastocladiomycota
Rozellomycota
Microsporidia
Metazoa
Amoebozoa
Basal opisthokonts

REV7/FANCV

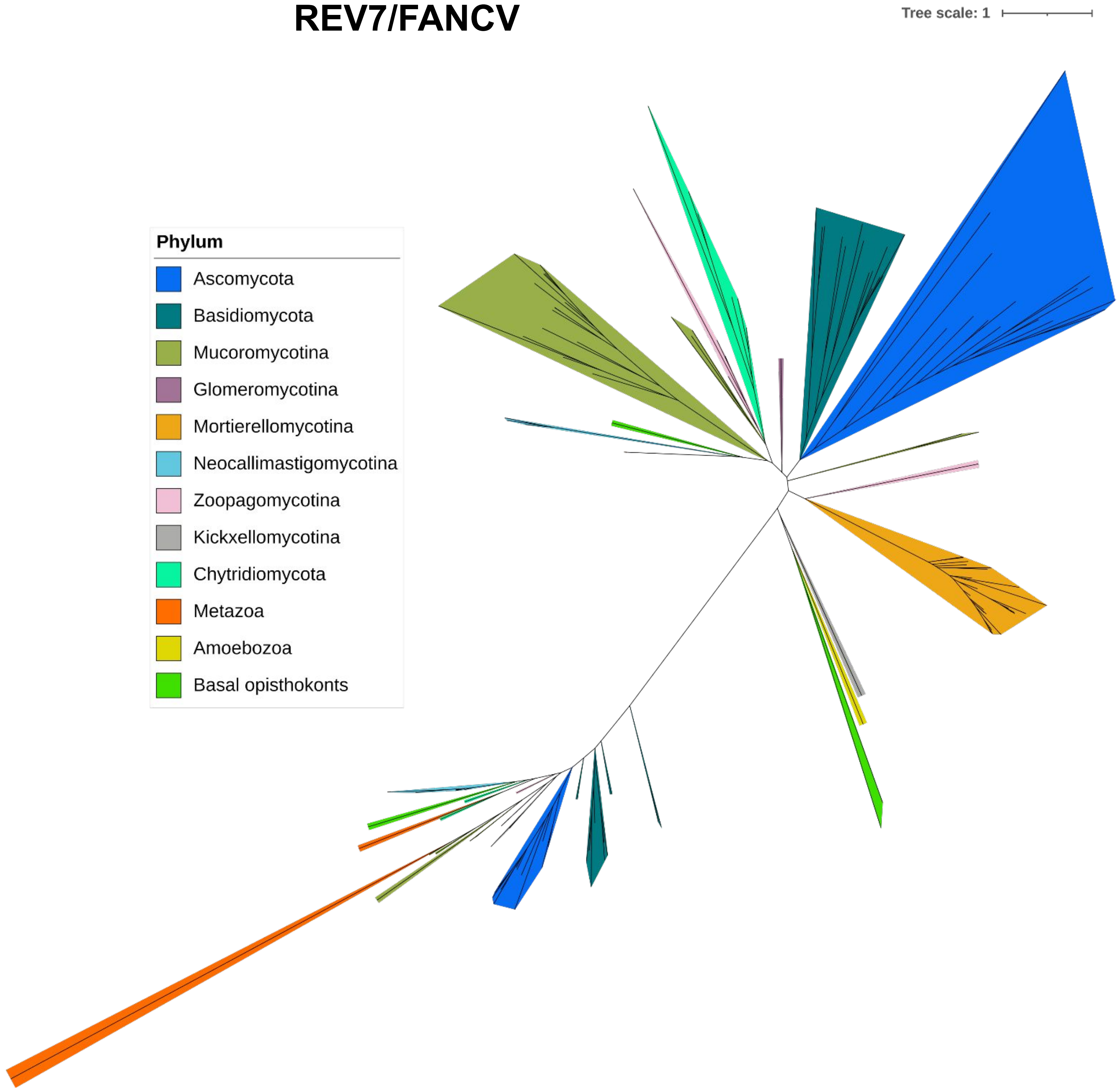

FANCW

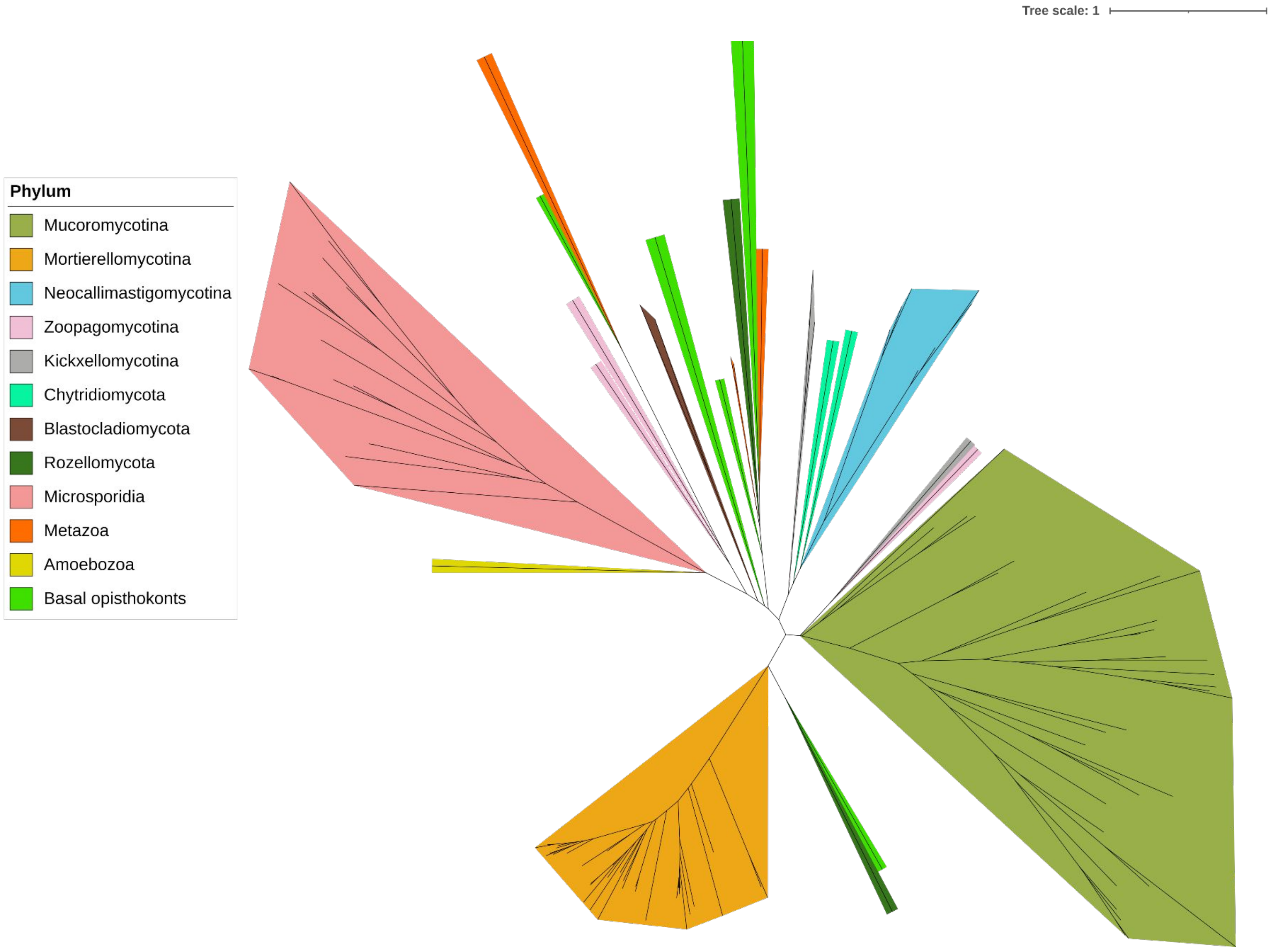

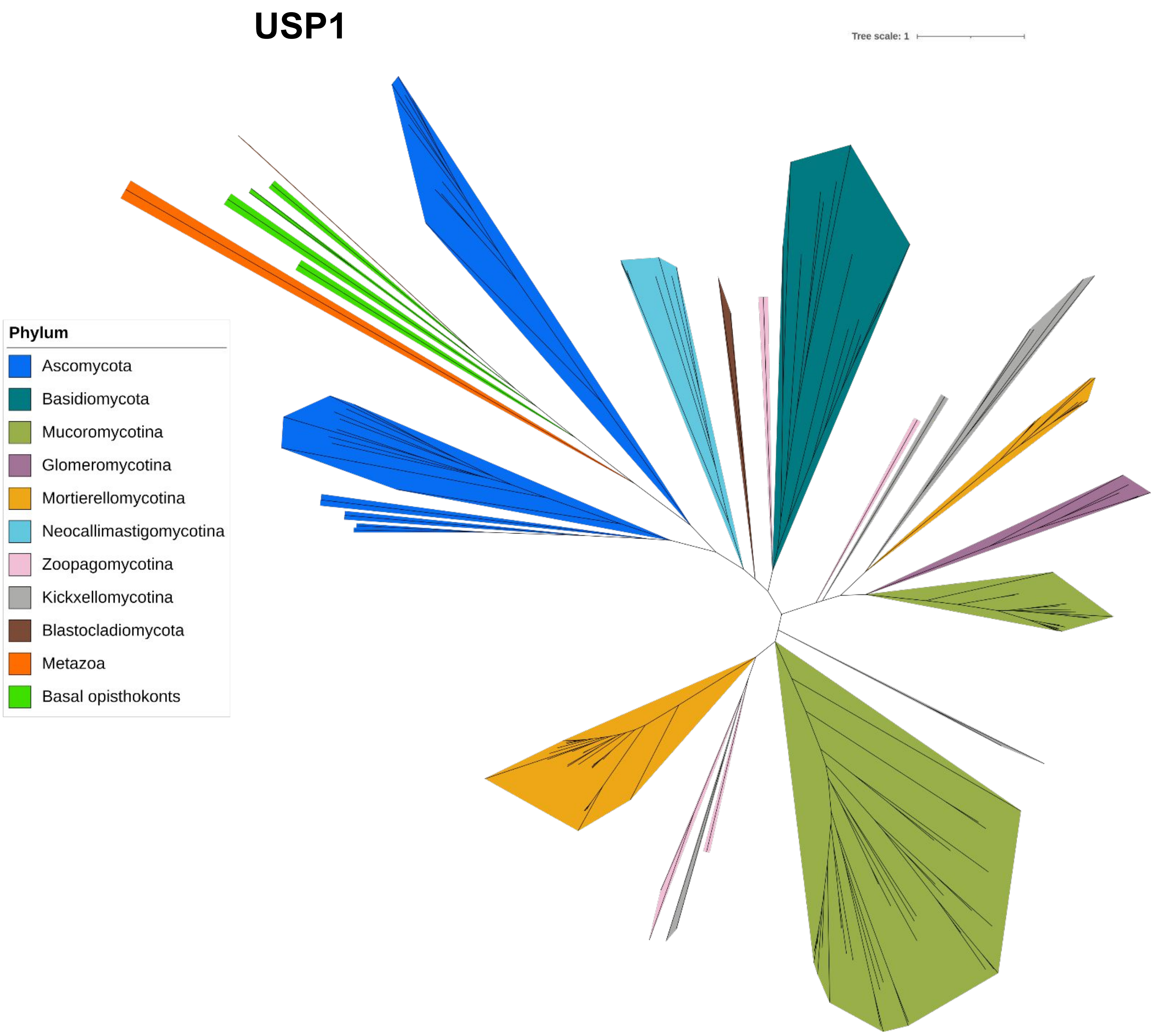
USP1
Tree scale: 1
Phylum
Ascomycota
Basidiomycota
Mucoromycotina
Glomeromycotina
Mortierellomycotina
Neocallimastigomycotina
Zoopagomycotina
Kickxellomycotina
Blastocladiomycota
Metazoa
Basal opisthokonts

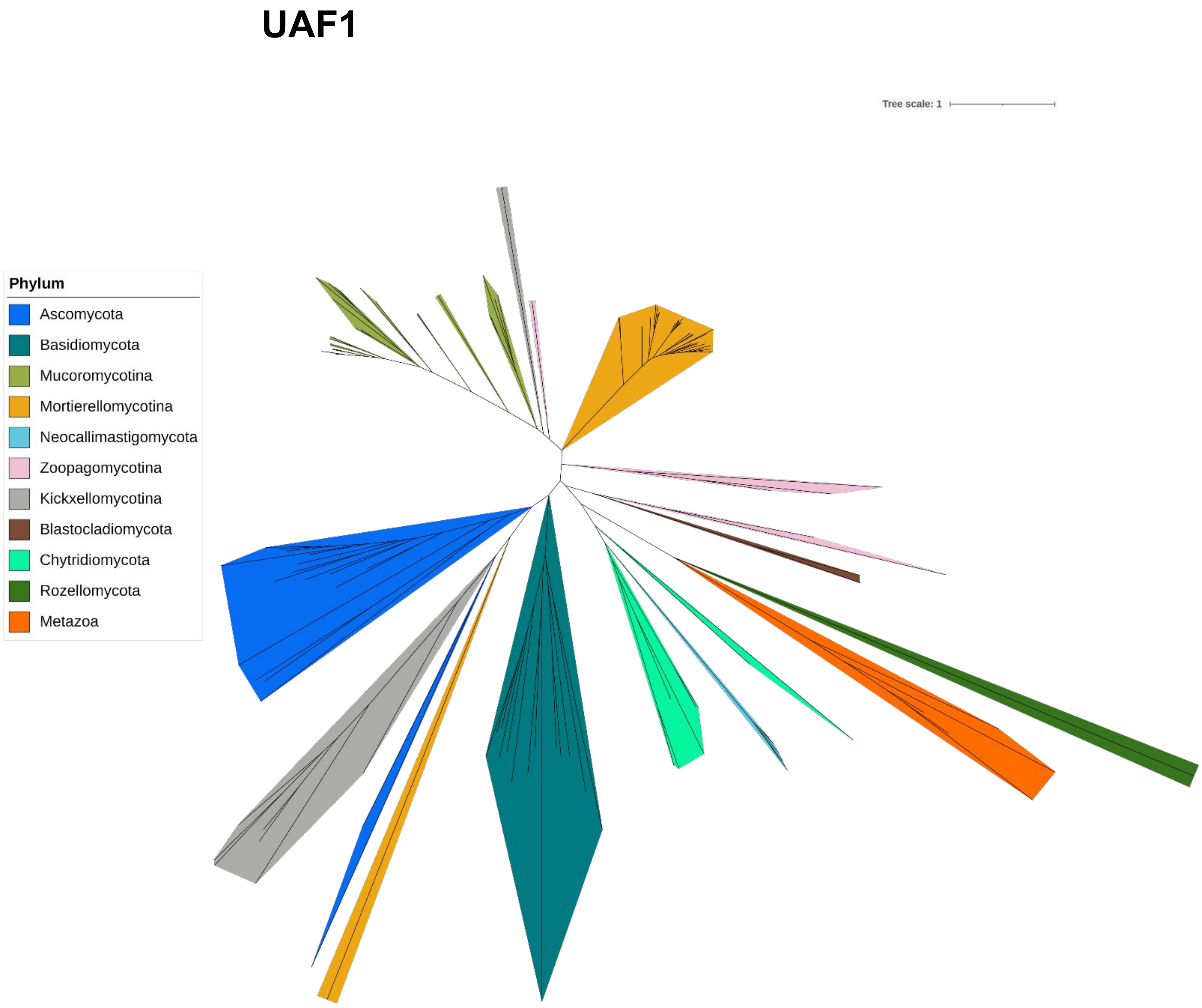
UAF1
Tree scale: 1
Phylum
Ascomycota
Basidiomycota
Mucoromycotina
Mortierellomycotina
Neocallimastigomycota
Zoopagomycotina
Kickxellomycotina
Blastocladiomycota
Chytridiomycota
Rozellomycota
Metazoa

BRCA1

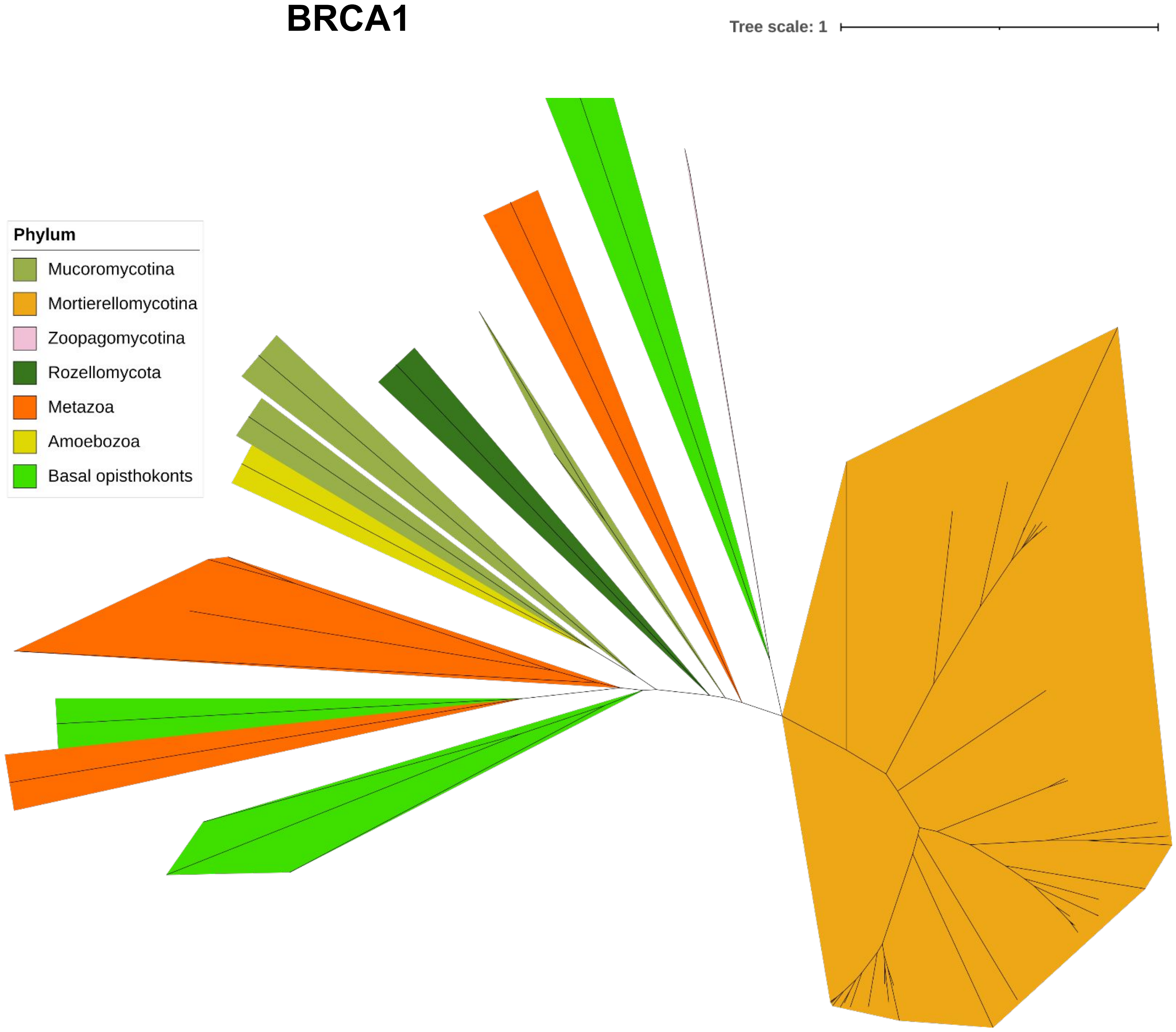

Tree scale: 1
Phylum
Mucoromycotina
Mortierellomycotina
Zoopagomycotina
Rozellomycota
Metazoa
Amoebozoa
Basal opisthokonts

BRCA2

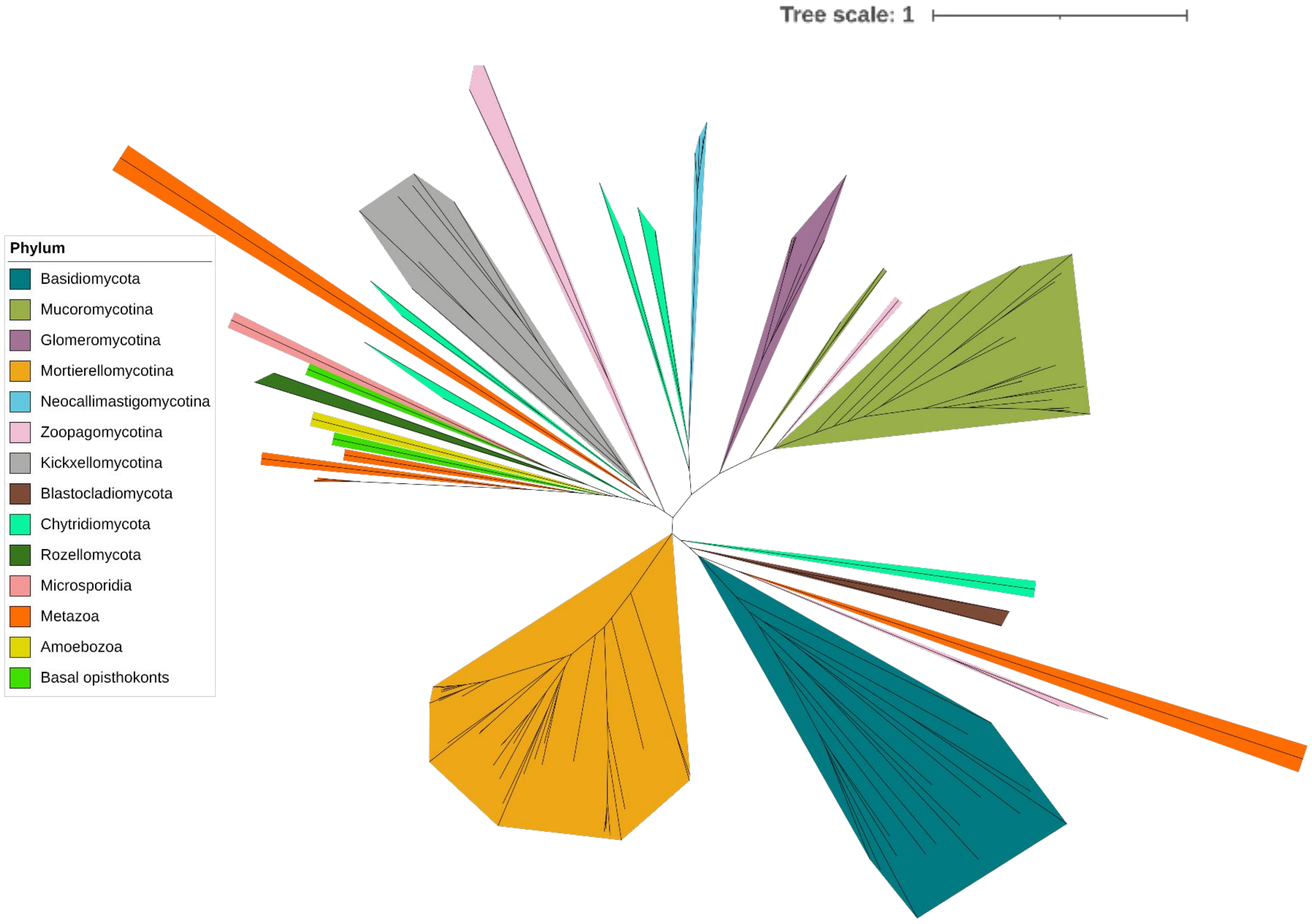

Tree scale: 1
Phylum
Basidiomycota
Mucoromycotina
Glomeromycotina
Mortierellomycotina
Neocallimastigomycotina
Zoopagomycotina
Kickxellomycotina
Blastocladiomycota
Chytridiomycota
Rozellomycota
Microsporidia
Metazoa
Amoebozoa
Basal opisthokonts

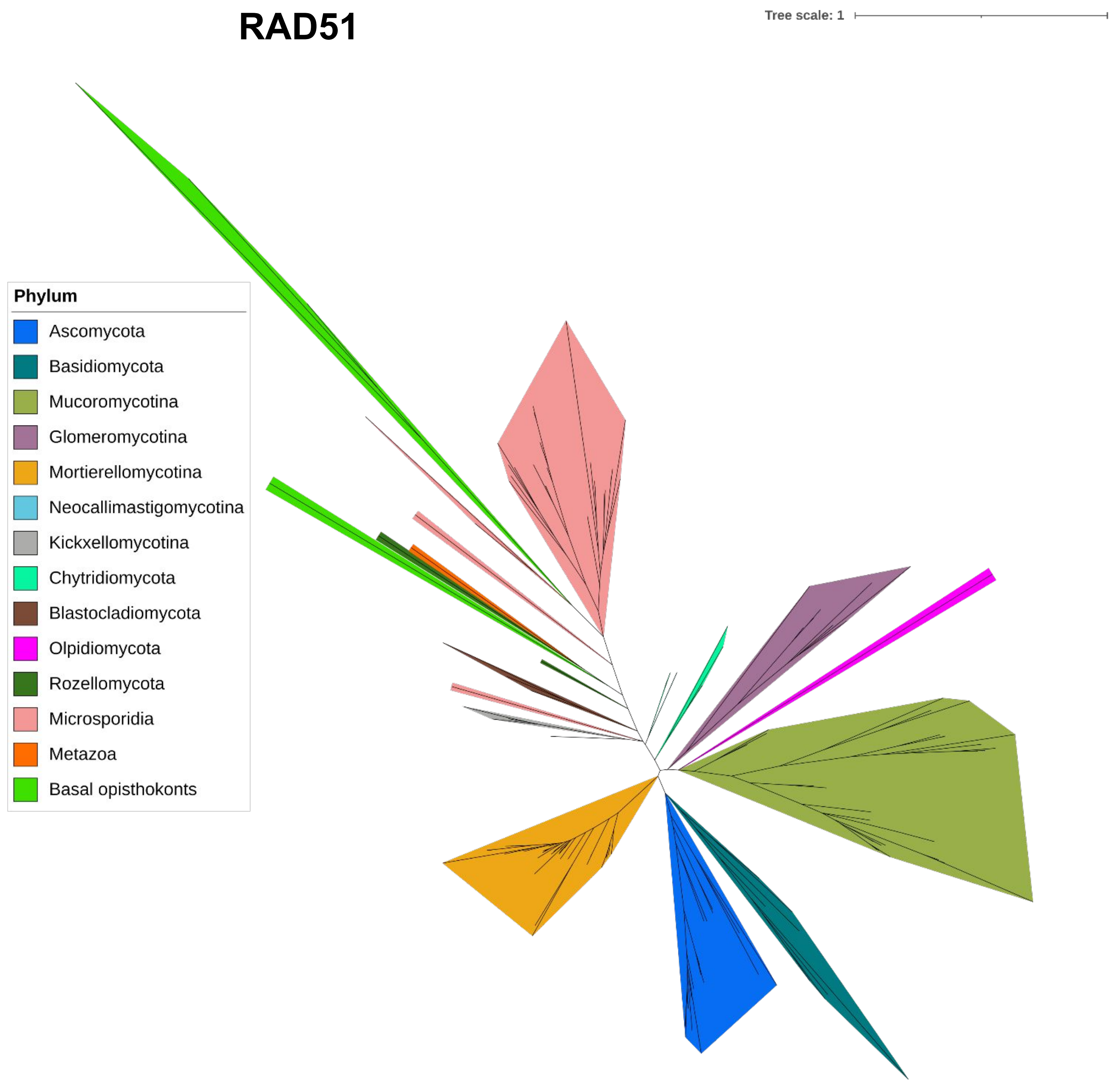
RAD51
Tree scale: 1
Phylum
Ascomycota
Basidiomycota
Mucoromycotina
Glomeromycotina
Mortierellomycotina
Neocallimastigomycotina
Kickxellomycotina
Chytridiomycota
Blastocladiomycota
Olpidiomycota
Rozellomycota
Microsporidia
Metazoa
Basal opisthokonts

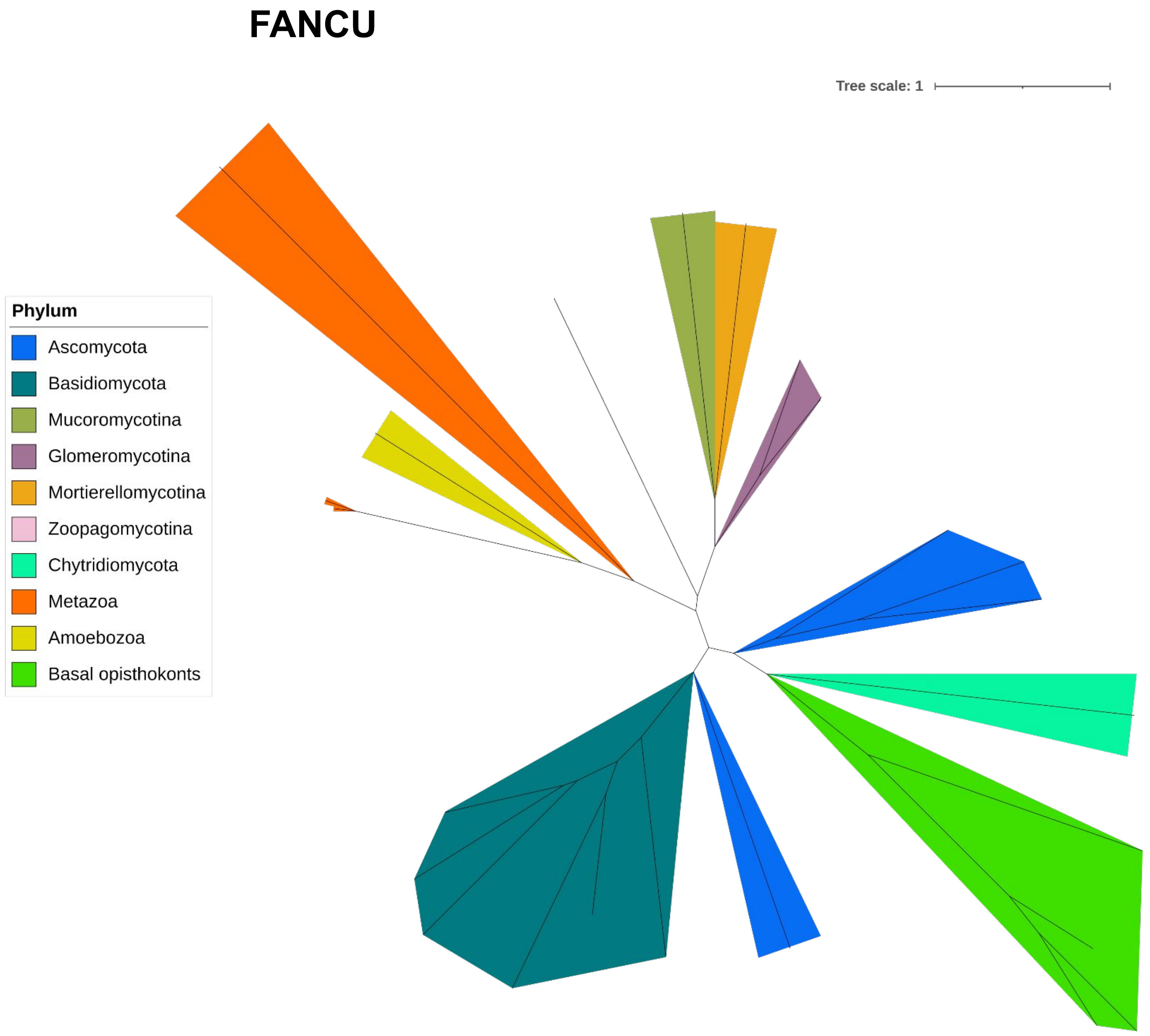
FANCU
Tree scale: 1
Phylum
Ascomycota
Basidiomycota
Mucoromycotina
Glomeromycotina
Mortierellomycotina
Zoopagomycotina
Chytridiomycota
Metazoa
Amoebozoa
Basal opisthokonts
