# Supplementary material for: A minimal Fanconi Anemia complex in early diverging fungi
Source: Sci Rep. 2024 Apr 30;14:9922. doi: 10.1038/s41598-024-60318-w (PMC11061109; doi:10.1038/s41598-024-60318-w)
Supplement: Supplementary file 4 — Supplementary Information 3. [file 41598_2024_60318_MOESM4_ESM.pdf]

A minimal Fanconi Anemia complex in Early Diverging Fungi

Drishtee Barua, Magdalena Płecha, Anna Muszewska*

Institute of Biochemistry and Biophysics, Polish Academy of Sciences, Pawińskiego 5A, 02-106 Warsaw, Poland

* corresponding author: Anna Muszewska musze@ibb.waw.pl

Supplementary Results - Taxonomic distribution comments

A minimal Fanconi Anemia complex in Early Diverging Fungi **1**
Supplementary Results - Taxonomic distribution comments 1
Activation complex proteins have been lost several times in fungal evolution 1
Core binding proteins are sparse across the fungal kingdom 2
Ubiquitination complex 2
ID complex is highly conserved in early diverging fungi 3
Endonuclease complex 3
FA downstream effectors have a patchy distribution among Fungi 3
The FA pathway closure system is conserved in Fungi 4

Activation complex proteins have been lost several times in fungal evolution

Our results show a scattered distribution of MHF1 and MHF2 proteins in *Mucoromycota*, *Blastocladiomycota, Chytridiomycota* along with *Taphrinomycotina*, *Saccharomycotina* and *Pucciniomycotina*. Remaining fungal phyla have lost one or both of the activation complex proteins. On one hand, nematodes, sponges and molluscs possess both proteins; on the other,

they seem to be absent from tested *Holozoa* representatives. Moreover, MHF1 protein was also found in the amoeba *Dictyostelium* and *Holomycota*.

Core binding proteins are sparse across the fungal kingdom

Proteins FANCJ and FANCM are conserved in all analyzed fungal isolates and the only two core FA proteins in *Dikarya*. A notable number of core binding proteins are absent from the fungal kingdom and seem to be present only in mammals and molluscs (FANCA, FANCB, FANCC, FANCF, FANCG, FAAP20, FAAP24 and FAAP100) (**Fig.2**). While FANCL has uniform conservation across EDF and basal Opisthokonts, FANCA and FANCE are present only in two EDF groups (*Glomeromycotina* and *Mucoromycotina*). Additionally, FANCO appears in selected EDF (*Mucoromycota*, *Chytridiomycota* and *Olpidiomycota*). FANCA, FANCE, FANCL and FANCO seem to have an ancient origin confirmed by their presence in molluscs, amoebozoans and *Holomycota*.

Ubiquitination complex

The ubiquitination protein UBE2T is conserved in all fungal lineages; however the ATR kinase is scattered across EDF lineages. Both UHRF1 and UHRF2 occur in *Zoopagomycota*, *Blastocladiomycota, Chytridiomycota* and *Agaricomycotina*. *Pezizomycotina*, *Pucciniomycotina* and *Mucoromycotina* retained only UHRF1 and lost UHF2. All four ubiquitination complex components are missing in basal Opisthokonts and *Olpidium*. *Rozellomycota* and *Microsporidia*, nematodes and arthropods lost UHRF1 and UHRF2. UBE2T and ATR are conserved in *Holomycota* and *Dictyostelium*.

ID complex is highly conserved in early diverging fungi

The ID proteins FANCD2 and FANCI show high conservation in the EDF groups except the absence of FANCI from *Olpidiomycota*. In contrast to the ubiquity of ID complex components among EDF, evolutionary younger *Dikarya* lost both of the components. On the other hand, they are completely absent from *Dikarya, Microsporidia and Sphaeroforma arctica*. Most of the early Opisthokonts along with *Holomycota*, however, have only one of the two proteins FANCD2. The ID complex is present in animals and amoebozoa.

Endonuclease complex

The scaffold protein SLX4 that binds to the endonucleases, is conserved in EDF and *Ascomycota*, with losses observed in *Basidiomycota*, *Olpidiomycota* and basal opisthokonts. The loss of this protein is also seen in nematodes and amoebozoans. Its partner SLX1 is conserved in fungi (except in *Wallemiomycotina*, *Neocallimastigomycota* and *Rozellomycota*) and metazoans but absent from early Opisthokonts and amoeba. The endonuclease pairs EME1-MUS81 and XPF-ERCC1 are conserved across the fungal tree, with an instance of loss of EME1 in *Kicxellomycotina* and MUS81 in *Wallemiomycotina*. Early diverging Opisthokonts lost four of the endonuclease complex elements (EME1, XPF, SLX1 and FAN1). The FAN1 nuclease was lost independently several times since it is missing from *Glomeromycotina*, *Neocallimastigomycota* and arthropods.

FA downstream effectors have a patchy distribution among Fungi

While REV3 is conserved across all lineages, REV1 and REV7 are absent in *Ustilagomycotina, Glomero- and Kickxellomycotina.* Homologues of the vertebrate DNA polymerase v DPOLN however, are found in selected EDF members of *Mucoromycotina*, *Mortierellomycotina* and *Chytridiomycota* along with *Holomycota*. FANCU shows a non-uniform distribution among fungi.

Protein PALB2 involved in HR is present only in mammals, while BRCA1 is found in all metazoans and EDFs (*Mucoromycotina*, *Mortierellomycotina*, *Entomophthoromycotina* and *Zoopagomycotina*). Interestingly, BRCA2 is conserved across all fungi with exceptions in *Ascomycota*, *Umbelopsidales* and *Zoopagomycotina*. RAD51 is conserved across the selected eukaryotic lineages, while FANCW is absent in *Dikarya* members, *Chytridiomycota* and *Glomeromycotina*. On the other hand, FANCO is also absent from members of *Ascomycota*, *Kickxellomycotina* and *Neocallimastigomycotina*.

The FA pathway closure system is conserved in Fungi

While UAF1 of the deubiquitination complex is conserved across all lineages except *Microsporidia*, the deubiquitylating enzyme USP1 is absent from *Taphrinomycotina*, *Olpidium bornovanus*, *Rozellomycota* and *Monoblepharomycota*, a subgroup of *Chytridiomycota*. USP1 is also absent in sponges.
